# Society for Immunotherapy of Cancer consensus statement on immunotherapy for the treatment of renal cell carcinoma

**DOI:** 10.1186/s40425-016-0180-7

**Published:** 2016-11-15

**Authors:** Brian I. Rini, David F. McDermott, Hans Hammers, William Bro, Ronald M. Bukowski, Bernard Faba, Jo Faba, Robert A. Figlin, Thomas Hutson, Eric Jonasch, Richard W. Joseph, Bradley C. Leibovich, Thomas Olencki, Allan J. Pantuck, David I. Quinn, Virginia Seery, Martin H. Voss, Christopher G. Wood, Laura S. Wood, Michael B. Atkins

**Affiliations:** 1 Cleveland Clinic Taussig Cancer Institute, 9500 Euclid Ave, Cleveland, OH 44195 USA; 2 Beth Israel Deaconess Medical Center, Dana-Farber/Harvard Cancer Center, 10 Highfield Circle, Milton, MA 02186 USA; 3 Johns Hopkins Sidney Kimmel Comprehensive Cancer Center, 1650 Orleans St, Baltimore, MD 21287 USA; 4 Kidney Cancer Association, PO Box 4668 #38269, Chicago, IL 60680 USA; 5 Cleveland Clinic Taussig Cancer Institute, 1 Clinic Center, Cleveland, OH 44195 USA; 6 Patient and Patient Advocate, Cleveland, USA; 7 Cedars-Sinai Medical Center, 8700 Beverly Blvd., Saperstein Critical Care Tower, 1S28, Los Angeles, CA 90048 USA; 8 Charles A. Sammons Cancer Center, Baylor University Medical Center, 3410 Worth Street, Suite 400, Dallas, TX 75254, USA; 9 M.D. Anderson Cancer Center, 1515 Holcombe Boulevard, Houston, TX 77030, USA; 10 Mayo Clinic, 4500 San Pablo Road, Jacksonville, FL 32224, USA; 11 Mayo Clinic, 200 First Street SW, Rochester, MN 55905, USA; 12 The Ohio State University Medical Center, 320 W. 10th Avenue, Columbus, OH 43210, USA; 13 UCLA Institute of Urologic Oncology, 66-124 Center for Health Sciences, Los Angeles, CA 90095, USA; 14 Kenneth J. Norris Comprehensive Cancer Center, University of Southern California, 1441 Eastlake Ave Suite 3451, Los Angeles, CA 90033, USA; 15 Memorial Sloan-Kettering Cancer Center, 1275 York Avenue, New York, NY 10065, USA; 16 Georgetown-Lombardi Comprehensive Cancer Center, 3970 Reservoir Road, NW, Research Building, Room E501, Washington, DC 20057, USA

**Keywords:** Guidelines, Immunotherapy, Renal cell carcinoma, Treatment

## Abstract

Immunotherapy has produced durable clinical benefit in patients with metastatic renal cell cancer (RCC). In the past, patients treated with interferon-alpha (IFN) and interleukin-2 (IL-2) have achieved complete responses, many of which have lasted for multiple decades. More recently, a large number of new agents have been approved for RCC, several of which attack tumor angiogenesis by inhibiting vascular endothelial growth factors (VEGF) and VEGF receptors (VEGFR), as well as tumor metabolism, inhibiting the mammalian target of rapamycin (mTOR). Additionally, a new class of immunotherapy agents, immune checkpoint inhibitors, is emerging and will play a significant role in the treatment of patients with RCC. Therefore, the Society for Immunotherapy of Cancer (SITC) convened a Task Force, which met to consider the current role of approved immunotherapy agents in RCC, to provide guidance to practicing clinicians by developing consensus recommendations and to set the stage for future immunotherapeutic developments in RCC.

**Electronic supplementary material:**

The online version of this article (doi:10.1186/s40425-016-0180-7) contains supplementary material, which is available to authorized users.

## Introduction

For more than 20 years, immunotherapy using IL-2 or IFN has been a primary treatment for patients with metastatic RCC (mRCC) [1–5]. The toxicity of high-dose (HD) IL-2 therapy, in particular, has restricted its use to patients with adequate organ function and treated at institutions experienced in the management of side effects. Multiple studies over many years have tried to identify biologic and immunologic parameters to pre-select patients for sensitivity to HD IL-2, but to date there is no biomarker for response related to the tumor itself or to the patient’s immune profile that has been prospectively validated. The most recent prospective study demonstrated that clinico/pathologic parameters such as excellent performance status and clear cell histology remain as the best predictors of HD IL-2 responsiveness [6]. Investigations have also clarified prognostic groups, and those for whom immunotherapy is not useful. The identification of biomarkers predictive of response or resistance to immunotherapy continues to be the focus of active research. Over many years of evaluation, it has become apparent that RCC is comprised of a number of different histologic subtypes, now shown to have different genomic profiles [7]. It has also been noted in multiple clinical trials and registry experiences that non-clear cell RCC is much less likely to respond to IL2 or IFN. The treatment approach for non-clear cell RCC continues to be explored with optimal subtype-specific strategies yet to be developed.

Since 2005, ten agents have been approved for the treatment of patients with metastatic clear cell RCC (which continues to be 75–85 % of mRCC). These include six agents that target the tyrosine kinase of receptors of VEGF (sorafenib, sunitinib, pazopanib, axitinib, cabozantinib, lenvatinib [in combination with everolimus]) [8–11], two that target mTOR (temsirolimus, everolimus) [12, 13], and a monoclonal antibody that binds VEGF before it engages its receptor (bevacizumab) [14, 15]. These agents have brought treatment options to greater numbers of patients with metastatic clear cell RCC. The selection of patients for different treatment options as well as the sequencing of these targeted agents relative to each other continue to be topics of clinical investigation. Despite an abundance of newer agents, there continues to be a role, albeit more limited, for cytokine-based immunotherapy. In addition, new immunotherapeutic agents are entering the clinical arena, notably nivolumab, an immune checkpoint inhibitor for programmed death 1 (PD-1) (nivolumab) [16]. Therefore, optimal sequencing becomes even more important in order to provide patients with the greatest chance of durable disease control and survival that is free from symptoms of disease or treatment.

In the era of anti-angiogenesis therapy, with agents that are available to nearly all patients with mRCC, SITC has convened a panel of RCC/immunotherapy experts to consider the current data and to provide treatment recommendations to practicing clinicians caring for patients with RCC, outlining the current and potential future role of immunotherapy for this disease.

## Methods

### Consensus statement policy

SITC has adopted a process and standards, initially outlined by the Institute of Medicine, to develop clinical practice guidelines for the use of immunotherapy [17, 18]. This paper is the result of this process in delineating guidelines for the use of immunotherapy in the treatment of renal cell cancer. SITC convened a multi-disciplinary panel of renal cancer/immunotherapy experts in October 2014 to produce an evidence-based guideline document, transparent with regard to funding as well as the reporting and management of conflicts of interest. The resulting document is designed to provide guidance only. The panel focused on drugs currently approved by the U.S. Food and Drug Administration (FDA) for the treatment of patients in the U.S. The final consensus statement and was made available to the entire SITC membership for open comment. This feedback received during the comment period were considered for the final manuscript (Additional file 1). Due to the approval of two agents and release of phase III data since the convened meeting, additional edits, approved by all authors, were also incorporated.

### Renal cancer consensus Task Force and conflicts of interest

The Task Force consisted of 17 health care providers, all specializing in the treatment of patients with RCC (12 medical oncologists, 3 urologic oncologists, and 2 oncology nurses), as well as 2 patient advocates and 1 patient (Additional file 2). The providers were particularly experienced in the management of patients with either advanced or local/regional disease. More than 80 % had experience with HD IL-2 and with anti-angiogenesis agents, and more than 75 % had experience with mTOR inhibitors. In addition, more than 85 % had experience with RCC clinical trials. Clinical trial participation among the Task Force members included studies involving anti-PD-1 (80 %), anti-PD-L1 (59 %), allogeneic bone marrow transplantation (29 %), RCC vaccines (45–50 %), and cabozantinib, a VEGF and MET inhibitor (65 %). Thus, the Task Force was a highly selected group of experts with long-standing experience in RCC treatment and clinical research and reflects the forefront of individuals conducting clinical trials with the newer agents for RCC over the past decade. Several Task Force members were also involved in the development and conduct of adjuvant clinical trials in patients with high risk RCC.

All Task Force members were required to disclose any conflicts of interest related to the treatment of RCC and the agents to be discussed during the conference. This included full financial disclosure of relationships with commercial sponsors of these agents. No commercial funding was provided for any aspect of the process, including the literature search, support of the meeting, or preparation of the manuscript.

### Literature review

The database selected for the literature review was MEDLINE. The search terms that were utilized included “kidney cancer or renal cancer and immunotherapy” with subtopics of “BMT” and “other/vaccine,” “kidney cancer or renal cancer and interferon,” “cytokine monotherapy” with subtopics “Bev/interferon” and “Peg-IFN,” “kidney cancer or renal cancer and IL2,” and “kidney cancer or renal cancer and anti-PD-1.” The literature search was supplemented with additional papers identified by the Task Force at the time of the consensus meeting. This resulted in a 290-item bibliography (Additional file 3).

The level of evidence reported in the literature was placed into one of three levels. Level A was considered the strongest supportive evidence, demonstrated by randomized, controlled trials and/or by meta-analyses as well as by long-term follow-up of prospective, uncontrolled trials in the case of HD IL-2. Level B was considered moderate evidence supported by more recent prospective, uncontrolled trials, and level C was considered weak evidence, derived from case reports and retrospective reviews.

### Task Force consensus meeting agenda

Topics discussed with respect to immunotherapy of RCC were the following: 1) the current role and place of HD IL-2 therapy; 2) the selection of patients for IL-2-based regimens and the criteria for those choices; 3) the current role of IFN and its use in conjunction with bevacizumab; 4) the identification of biomarkers of response to immunotherapy; 5) the sequencing of immunotherapy with the anti-VEGF agents; 6) the management of patients with central nervous system (CNS) metastases; 7) the potential role and sequencing of new immunotherapy agents including the PD-1/PD-L1 pathway inhibitors; 8) future opportunities and the role of immunotherapy-based combination therapies for RCC. The pre-meeting survey questions and Task Force responses are also available in full (Additional file 4).

It was agreed that the data supporting the use of IL-2 originates from older studies, rather than phase III comparative trials, and therefore, the basis for recommendations regarding this agent reflects decades of clinical experience. Because of the need for careful selection of patients for HD IL-2, historical data must be the basis for recommendations. More recently, a prospective phase II clinical trial of HD IL-2 suggested that in the current era of alternative treatment options, patients felt to be appropriate for HD IL2 treatment experience a higher response rate than in the initial reports (25 vs. 14 %) [6]. Additionally, registry data from treatment centers in the current era demonstrate enhanced activity and reduced severe toxicity for this treatment approach [19, 20].

## Consensus recomendations

### What is the role of systemic therapy for resected stage II/III renal cell cancer?

Although clinical trials of HD IL-2 and of IFN were conducted as adjuvant therapy, results did not support their use in this setting [21, 22]. VEGFR tyrosine kinase inhibitors (TKIs) sorafenib and sunitinib did not demonstrate benefit relative to placebo in the E2805 ASSURE trial [23]. However, recent preliminary data from S-TRAC indicate a relapse-free survival benefit to sunitinib over placebo in patients with resected high-risk RCC [24]. Full details regarding this trial including overall survival and relative toxicity are awaited to determine if sunitinib will be a new standard of care in this setting. Other ongoing adjuvant clinical trials awaiting results include: EVEREST (S0931, NCT01120249), a phase III comparison of everolimus versus placebo in the North American Cooperative Groups, which will complete accrual shortly and SORCE, a randomized phase III trial of one year of sorafenib versus three years of sorafenib versus observation conducted in Europe, which completed accrual and is pending analysis [25]. In addition, two industry-sponsored trials (PROTECT and ATLAS) are ongoing.

### Literature review and analysis

The earliest adjuvant trials in patients with completely resected RCC were with IFN. The North American Cooperative Groups conducted an intergroup study, enrolling from 1987–1992, in which 283 patients with pT3-4a and/or lymph node positive patients were randomized to observation or to IFN alfa-NL, administered daily for 5 days, every 3 weeks, for up to 12 cycles [22]. At a median follow-up of 10.4 years, the median survival was 7.4 years in the observation group, and 5.1 years in the IFN group (log rank *p =* 0.9). Median recurrence-free survival (RFS) was 3.0 years for the observation group and 2.2 years in the IFN group (*p =* .33). The investigators concluded that adjuvant treatment with IFN did not contribute to survival or RFS [22]. The Cytokine Working Group (CWG) conducted an adjuvant study in a mixed population of 69 resected locally advanced or metastatic patients, comparing HD IL-2 to observation [21]. Early closure was recommended after an interim analysis determined that the goal of a 30 % improvement in 2-year disease-free survival (DFS) could not be achieved with further accrual.

As stated above, a number of randomized, placebo-controlled adjuvant trials utilizing anti-VEGF agents or anti-mTOR agents are being completed and/or undergoing analysis. The first report was of ASSURE in 2015, demonstrating no difference in RFS comparing sunitinib to placebo or sorafenib to placebo [23]. Ongoing genomic studies may provide insights into differing populations among the patients in this trial. Considerable enthusiasm is developing for adjuvant trials of checkpoint inhibitors in resected RCC and such trials are in development.

### Consensus recommendations

The entire Task Force agreed that the current standard of care in the adjuvant setting is either observation or enrollment in a clinical trial based on Level A evidence for cytokines [21, 22] and Level A evidence from the ASSURE clinical trial [23]. The panel was supportive of initiation of studies utilizing PD-1 pathway blocking agents in the neoadjuvant and/or adjuvant setting and such trials are in development (Table 2). The preliminary S-TRAC data release, which occurred after the meeting, may impact both the standard of care and the control arms of future clinical trials in this setting.

### What is the role of surgery for stage IV renal cell cancer?

#### Initial assessment of a patient with mRCC

Patients with mRCC should be evaluated for histologic subtype and extent of metastatic disease, including evaluation of the CNS. In the presence of small volume metastatic disease, relative to the tumor volume in the primary site, cytoreductive nephrectomy is often recommended prior to systemic therapy [26–29]. Data suggest improved survival associated with cytoreductive nephrectomy in the cytokine era [26–29] and preliminarily also with VEGFR pathway targeted therapy [30]. If there are isolated distant metastases, these may be considered for resection as data support this approach [31, 32]. Systemic therapy is not indicated after metastasectomy in the absence of residual disease except as part of a research study. There is an ongoing cooperative group clinical trial evaluating pazopanib versus placebo in the setting of resected metastatic disease (E2810, NCT01575948).

However, if patients have a large tumor burden outside of the kidney, particularly symptomatic distant metastases, or poor performance status/co-morbidities, then initiating therapy without nephrectomy may be appropriate and should be strongly considered as part of a multi-disciplinary discussion.

#### Literature review and analysis

Early studies demonstrated improved survival in patients presenting with metastatic disease, who subsequently underwent nephrectomy and were then treated with IFN, compared in randomized trials with those only treated systemically [27–29]. Similarly, nephrectomy prior to HD IL-2 confers benefit [26]. A more recent report suggests that this benefit may be limited to selected patients, with survival being primarily improved in patients with favorable Memorial-Sloan Kettering Cancer Center (MSKCC) or Eastern Cooperative Oncology Group (ECOG) prognostic features among patients treated with VEGF-targeted therapies [30]. Several reports also describe survival benefit from resection of concurrent or recurrent metastatic disease, again in highly selected patients [31, 32].

#### Consensus recommendations

These comments were discussed as part of the general discussion and were not voted on. In general, the Task Force agreed that nephrectomy remains an important component of management of patients with mRCC based on Level A evidence for IFN and IL-2 [26–29] and Level C evidence for VEGF-targeted agents [30, 32]. The resection of oligometastases is supported by Level C evidence [31, 32]. It is unclear how novel immunotherapy may impact these surgical approaches.

#### Immunotherapy for mRCC

**Fig. 1:**
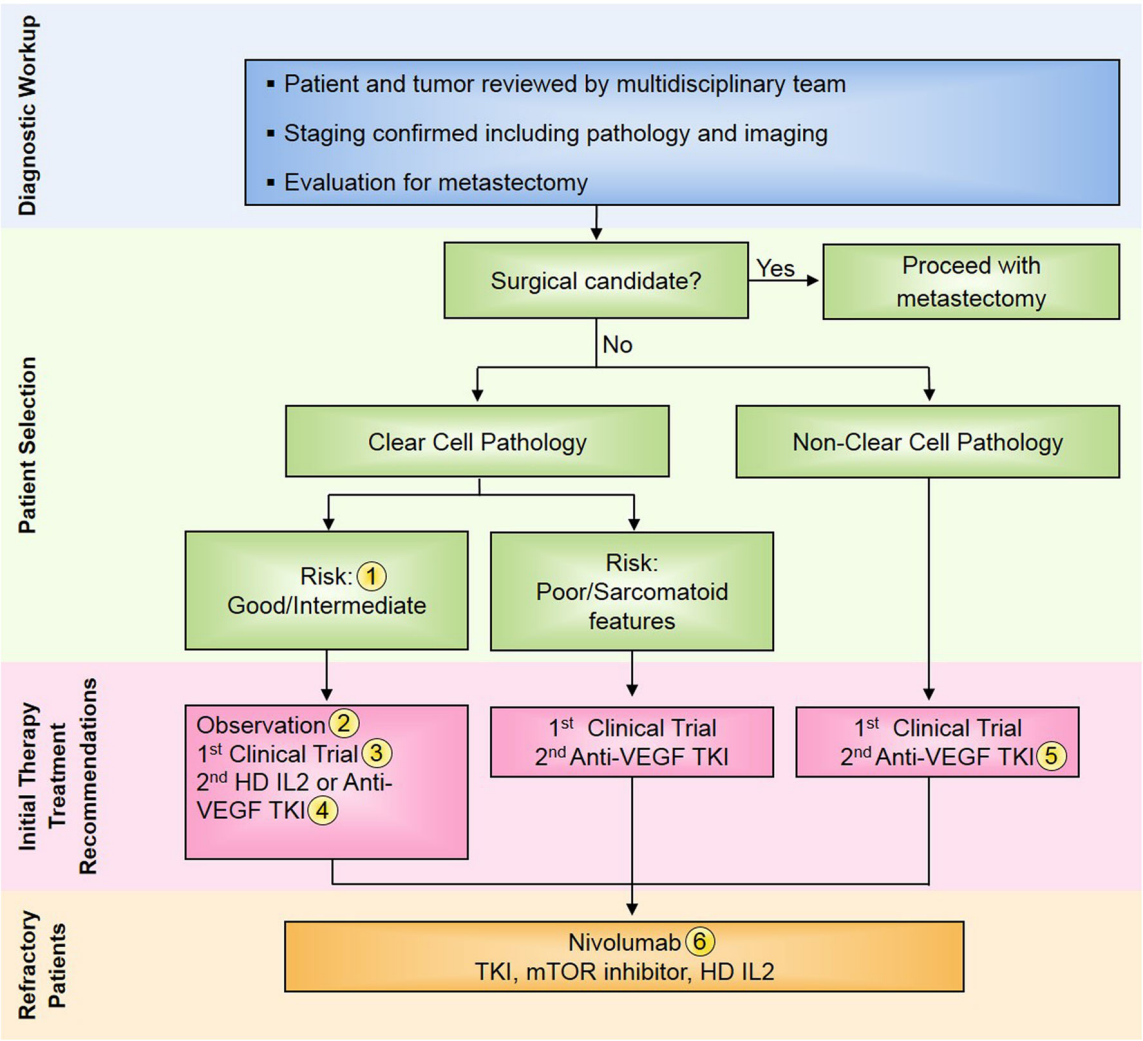
Stage IV renal cell carcinoma (RCC) immunotherapy treatment algorithm. All treatment options shown may be appropriate. The final selection of therapy should be individualized based on patient eligibility and the availability of each therapy at the treating physician’s discretion. 1) “Risk” refers to prognostic risk group per Memorial Sloan Kettering Cancer Center (MSKCC) and/or International Metastatic Renal Cell Carcinoma Database Consortium (IMDC) classification [49, 83]. 2) For patients with small-volume, indolent metastases, an initial period of observation can be considered accounting for patient age/comorbidities, patient preference, and toxicity of available therapy. 3) A clinical trial, including those that are immunotherapy-based, should be considered in all RCC patients in all lines of therapy. 4) As noted in the manuscript, HD IL2 should be considered and discussed with mRCC patients with clear cell histology and good performance status. 5) For patients with advanced non-clear cell renal cell carcinoma (RCC), if available a clinical trial is the preferred initial treatment option, including trials of checkpoint inhibitors for which limited data exists regarding efficacy in non-clear cell RCC. If unavailable, then a VEGFR tyrosine kinase inhibitor (TKI) is preferred given results from two small randomized trials showing a slight advantage over mTOR inhibitors in this setting [81, 82]. 6) Nivolumab is an appropriate initial recommendation in refractory RCC in the absence of contraindications given the overall survival benefit and tolerability. Other options (TKI, HD IL-2 and mTOR inhibitors) can be considered depending on patient performance status, comorbidities, prior therapy received and preference. Figure adapted from Kaufman et al., 2013 [18]

In the setting of residual metastatic disease, following nephrectomy, or recurrent metastatic disease, the Task Force discussed the role of first-line treatment with immunotherapy versus VEGF or mTOR targeted therapy for metastatic disease. The outcome of this discussion is outlined below and summarized in a treatment algorithm for patients with stage IV RCC (Fig. 1).

### What is the current role of HD IL-2 in the treatment of mRCC?

IL-2 is a cytokine that was initially called “T cell growth factor” [33] that activates both effector and regulatory T cells. It has shown clinical antitumor activity in preclinical models and clinical trials leading to its FDA approval in patients with advanced RCC in 1992 and melanoma in 1998 [2–4].

The FDA approval for HD IL-2 was based on the potential for a small subset of treated patients to achieve durable complete responses, which may last for decades. Therefore, centers that treat patients with mRCC frequently screen for HD IL-2 candidates prior to considering other types of agents as initial treatment. Research to develop biomarkers of responsiveness has been ongoing. However, criteria for patient selection remain clinical at this time. Many IL-2 treatment centers recommend HD IL-2 as initial treatment for patients with mRCC, depending upon the patient’s clinical condition and perceived ability to tolerate this therapy. Others recommend clinical trials since some, especially those evaluating frontline use of checkpoint inhibitors, preclude patients with prior therapy of any kind including HD IL-2.

#### Literature review and analysis

HD IL-2 was approved for treatment of mRCC in 1992, based on summarized data from 7 clinical trials consisting of 255 patients [3]. The overall response rate (ORR) was 15 % (37/255), including 17 complete (CR) and 20 partial responses (PR). Sixty percent of the PRs had more than 90 % reduction in tumor burden, and some were rendered complete responders by further surgery. The median duration of response was 54 months, including a median of 20 months for PR patients and median not reached for CR patients. The median survival for all 255 patients was 16 months [3].

Subsequent reports with data from a median of 10 years follow-up showed that 60 % of CR patients remained in complete remission. Additionally, 4 PR patients who underwent surgery of residual disease to achieve CR remained alive and disease-free at more than 65 + months [2, 4].

More recently, the CWG conducted a prospective, biomarker validation study entitled “SELECT” in which clinical and some biologic features were evaluated as potential selection factors for best response [6]. This study again demonstrated that HD IL-2 therapy yielded durable remission and prolonged survival in patients with mRCC. These results were achieved in patients considered both “poor” risk and “favorable” based on retrospectively derived criteria [34]. Clinico/pathologic criteria appeared to select for better outcome, such as clear cell histology (96 % of subjects) and prior nephrectomy (99 % of subjects), and these reflected selection prior to enrollment in the trial, based on previous clinical experience. This study demonstrated improved results compared to the historical studies postulated to be based primarily on better patient selection. One hundred and twenty eligible patients were enrolled, 70 % of them being intermediate risk based on MSKCC criteria. The independently evaluated ORR was 25 %, with 3 CRs and 27 PRs. Thirteen patients (11 %) remained progression-free at 3 years from treatment, and the median overall survival (OS) was 42.8 months [6]. Biomarkers that were evaluated and not found to be predictive of response were histologic subtype and CA-IX score by immunohistochemistry. Positive expression of PD-L1 in the tumor (18 patients) did significantly correlate with response, but this result requires validation [35].

Additional new data on outcomes with HD IL-2 treatment has been derived from single institution reports and the development of a national database registry of initially retrospective patients and now ongoing prospective collection of treatment and outcome data for HD IL-2 (PROCLAIM NCT 01415167,) [19, 20].

PROCLAIM data on the retrospective cohort of 97 patients with mRCC treated between 2007 and 2012 at 13 sites was presented in 2015. The ORR was 22 % (8 % CR and 14 % PR). The median OS was 51 months for the entire cohort. For those patients achieving CR, PR or stable disease (SD) > 6 months, the median OS has not been reached [19]. The median OS for those patients who directly progressed after IL-2 therapy was 37.9 months. There were no deaths due to IL-2 related toxicity among the 97 patients. Additionally, the median OS for those patients treated with HD IL-2 as first-line therapy was 61.8 months (*n =* 82) compared with a median OS of 15.3 months for those treated with IL-2 as second-line therapy (*n =* 15) [19]. Additional single institution data have been published, demonstrating similarly improved ORR and survival in the modern era [36].

#### Consensus recommendations

The Task Force was divided about the degree of the role that HD IL-2 has in initial treatment of patients with mRCC. The overall opinion was that appropriate patients with mRCC who have undergone nephrectomy, either in the past or as a cytoreductive intervention, should have a discussion about IL-2 and be referred to centers of excellence for further discussion when appropriate. Sixty-seven percent recommended that all such patients have a discussion regarding IL-2, whereas 33 % preferred to select the patients for that discussion. This recommendation was based on Level A evidence from long-term follow-up of multiple clinical trials [2–4] and Level B as well as C evidence from more recent prospective, uncontrolled clinical trials and clinical experience as noted in the literature review [6, 20, 25, 37].

### What are the criteria for considering IL-2 therapy?

The Task Force discussed in detail the clinical and biological criteria required for consideration of treatment with HD IL-2 (Table 1). These are specific to HD IL-2, but some criteria may also be applicable to emerging immunotherapy.

**Table 1:** Task Force criteria for HD IL-2 therapy

Criterion	Ranking^a^
Physiologic features	
Clear cell histology - no papillary or granular features	1.21
Adequate heart and lung function	3.57
Performance Status	3.71
Age (physiology ≤ 70 years)	4.64
Prior nephrectomy	4.93
Lack of CNS metastases (or treated)	5.42
Low priority	
No prior TKI use	7.27
MSKCC risk group	7.36
VLack of bone metastases	7.40
Lack of liver metastases	8.56
Lack of sarcomatoid histology	9.00
CA IX status	10.78
Other	11.00

^a^Each criterion was ranked from highest priority to lowest priority with 1 indicating the highest priority

### Literature review and analysis

Several clinical studies have demonstrated poorer outcomes of patients with non-clear cell RCC compared with those having clear cell RCC, after treatment with cytokines such as IL-2 or IFN [7, 12, 37]. A retrospective review of histology showed markedly improved outcome among patients with clear cell RCC after IL-2 therapy, compared with those with mixed histology or those with extensive granular features [37]. In this study, among patients with clear cell and favorable features (alveolar but no papillary or granular features) the ORR was 39 % (*n =* 36), and in patients with clear cell with < 50 % granular features, ORR was 19 % (*n =* 146). Among others including non-clear cell, the ORR was 6 % [37]. This report provided preliminary data that led to the evaluation of tumor histology that was prospectively evaluated in the renal SELECT clinical trial [6].

With respect to sarcomatoid differentiation, studies describe rapid clinical deterioration and report poorer outcomes overall among patients whose tumors have these features, regardless of treatment approach [38, 39]. Wu et al. described 7 patients with sarcomatoid histology, none of which responded to treatment with HD IL-2. Median survival among these patients was 13 months compared with a median survival of 39 months in 63 patients with clear cell RCC treated with HD IL-2. All were treated in the pre-VEGF pathway inhibitor era in a single institution [38].

Evaluations of potential biomarkers of response to HD IL-2 have been ongoing but have not provided guidance for specific populations who are more likely to respond. The prospective SELECT trial evaluated several biomarkers, such as tumor CA-IX expression, but this did not predict response. Further exploration of expression of PD-L1 as a biomarker of disease behavior and/or response to immunotherapy is ongoing [35]. Clinical selection by the above criteria remained the strongest predictors along with clear cell histology [6].

#### Consensus recommendations

In terms of biology, the histology of the renal tumor should be the first consideration [7, 38]. The majority of the Task Force felt that only patients with clear cell histology should be considered for HD IL-2. The Task Force discussed whether patients with tumors having sarcomatoid features should receive IL-2, and 40 % of participants would exclude such patients. Others would consider such patients, depending upon the proportion of sarcomatoid features noted and the biologic behavior of the disease (rapid or indolent). Thirteen percent would exclude patients with extensive granular features or Fuhrman grade 4 histology based on retrospective data [37]. PD-L1 expression versus a marker for aggressive RCC or a combination of both were discussed as biomarkers to predict sensitivity to IL-2 as suggested in the SELECT trial. However, this will need to be further verified [6, 35]. The level of evidence supporting the recommendations related to pathology are considered Level C, based on retrospective reviews [7, 12, 37–39] and Level B, based on a prospective, uncontrolled trial [6].

Clinical and physiologic criteria should also be evaluated prior to recommending HD IL-2. The following have long been established as criteria for patients to undergo HD IL-2 treatment: adequate heart and lung function; ECOG performance status 0–1, preferably 0; age (physiologic versus chronologic), but the upper limit for both is usually in the upper 70s; and absence of CNS metastases (or treated metastases, with no residual edema) [40, 41]. The Task Force agreed upon these and established a series of criteria and rated their level of importance (Table 1). This was based on Level A evidence from long-term follow-up in multiple trials [2–4, 40, 41].

### What is the role of immunotherapy in mRCC patients with CNS metastases?

The development of CNS metastases is not rare in mRCC and remains a criterion for exclusion from clinical trials. However, there are multiple modalities for treatment of small volume CNS lesions, including surgery and stereotactic radiation. When these modalities are successful, the previous CNS metastases do not alone preclude proceeding with systemic treatment of mRCC, including immunotherapy. The concern with HD IL-2 is the risk of increasing brain edema when administered to patients with untreated CNS metastases, and therefore, most clinicians screen for CNS involvement prior to starting HD IL-2. However, occasionally small lesions are not identified and such patients have been treated.

#### Literature review and analysis

Retrospective reports from the early decades of therapy with HD IL-2 have described treatment of patients with either treated or untreated CNS metastatic disease [42–44]. A report from University of California, Los Angeles (UCLA) described the outcome of 138 patients with mRCC who developed CNS metastases from 1989 to 2006 [42]. In this study, those with symptomatic and asymptomatic lesions as well as the total number of lesions were characterized. The results illustrated that patients with solitary lesions were less likely to develop additional CNS lesions. In addition, selected patients were able to proceed with HD IL-2 and experienced prolonged survival [42]. In this series, the median survival after diagnosis of CNS metastases was 10.7 months, and the 5-year survival was 12 %. Patients receiving HD IL-2 after CNS treatment had a response rate of 17 %.

Retrospective data from the National Cancer Institute consisted of more than 1000 patients with either melanoma or mRCC who were treated with HD IL-2 with or without other therapy from 1985 to 2000 [43]. Patients with previously treated CNS metastases (*n =* 27) had an ORR of 18.5 %, and those with no brain metastases (*n =* 1005) had an ORR of 19.8 % [43]. Two of 36 patients with untreated CNS lesions demonstrated objective response of both intracranial and extracranial disease. This report stated that there were no differences in toxicity profile or reasons for stopping IL-2 among those with CNS lesions and those without.

A third retrospective report described the management of CNS metastases in patients with mRCC, with the use of stereotactic radiation therapy (SRS) from 2000 to 2006 [44]. Among 32 patients with 71 CNS lesions, local control was achieved in 22 patients and 42 lesions. Whereas the median survival of all patients with CNS metastases was 10 months, 16 % achieved 3 year survival. In addition, these patients were able to proceed to systemic immunotherapy, including HD IL-2 and IFN [44].

Two later reports of patients with melanoma also describe the objective response of intracranial metastases to immunotherapy (HD IL-2 and adoptive cell therapy), confirming the ability of immunotherapy to induce regressions of intracranial tumors [45, 46].

#### Consensus recommendations

Given the heterogeneous and retrospective nature of the information available regarding management of CNS metastases in patients with mRCC, the Task Force felt that proceeding with HD IL-2 in this setting is individualized and relies on clinical judgment.

With respect to patients presenting with CNS metastasis, 47 % of the Task Force preferred the use of a VEGFR TKI after local treatment of the CNS disease. However, 40 % would treat the CNS lesion(s) with either surgery of stereotactic RT first, and then consider proceeding with HD IL2, if other criteria are met. The level of evidence for the recommendation for use of IL-2 was considered Level A, based on long-term follow-up [42, 43] and Level C based on short-term, retrospective data [44].

### What is the role of evaluation of risk factor prognostic categories in deciding treatment approach?

Several groups have evaluated clinical and laboratory features of patients with mRCC and have developed algorithms that define prognosis and survival. The initial report was developed retrospectively among patients treated with IFN [34], and additional retrospective studies demonstrated similar delineations of patients into favorable, intermediate (majority of mRCC) and poor risk groups [47, 48]. Subsequent evaluations have assessed risk criteria among patients treated with VEGF pathway inhibitors and have demonstrated consistent results [49]. The evaluation of such prognostic information has become useful in evaluating outcome of clinical trials by strata, as well as adding information when considering treatment options for patients.

#### Literature review and analysis

In the prospective SELECT trial, HD IL-2 produced durable remissions and prolonged survival in both good and poor risk patients according to MSKCC criteria; however, the poor risk patients were few in number [6]. Reports in clinical trials [8–12] and in the modern prognostic factor analyses with immune and targeted therapy [47–49] demonstrate the greatest treatment benefit for patients with mRCC to be among those with favorable and intermediate risk.

#### Consensus recommendations

Regarding the use of the prognostic categories that have been developed for predicting survival of patients with mRCC, the consensus of the Task Force was that these criteria are used for treatment decisions. Poor risk patients, with expected shortened survival are not considered initial candidates for HD IL-2, and the majority (53 %) would proceed with anti-VEGFR TKI, 20 % with temsirolimus, and 27 % with clinical trials, if available, in the setting of poor risk patients. These recommendations are based on Level B evidence from long-term retrospective reviews [34, 47–49] and Level B evidence from a prospective trial with IL-2 [16] as well as Level C evidence from retrospective evaluations of risk categories in studies of targeted therapies [8–15].

### What are considerations of duration of treatment with HD IL-2 and when to change therapy?

There was discussion regarding retreatment of patients following the first course of HD IL-2. Although chemotherapy treatment in oncology utilizes repetitive treatment cycles, the necessary treatment duration for immunotherapy continues to be evaluated. It is conceivable that once activation of the immune system occurs, additional treatment does not result in additional benefit.

#### Literature review and analysis

Based on the SELECT trial and PROCLAIM data, SD may be a therapeutic effect of IL-2. In SELECT, there was an ORR of 25 %, with 3 CRs and 27 PRs. The median duration of response was 20.6 months, and 13 patients progression-free at 3 years. There were 9 patients with SD lasting more than 6 months. The median OS was 43 months for all 120 patients [6]. In the retrospective and prospective PROCLAIM registry, which is still accumulating patient data, the response rate is 20 %, and the median OS has not been reached for the prospective category of patients [19]. The survival of stable patients aligned with the responders and was considerably better that that of the progressing patients [19].

#### Consensus recommendations

There were different opinions as to whether more than one course of HD IL-2 should be given to those patients who respond or are stable. In patients with responding or SD 12 weeks following HD IL-2, 80 % would give a second two week course of therapy. Thirteen percent would continue to observe, especially in patients with SD, until progression is documented, and then start another treatment. Anecdotal patients were discussed who achieved a durable CR with one course of HD IL-2. It has not been prospectively evaluated whether patients who have SD as their best response to the first course of HD IL-2 can achieve either a better response or delayed progression with additional courses of therapy. However, if no contraindication existed, the majority of the Task Force would proceed with a second course before changing treatment. The level of evidence was considered Level C, based on retrospective data and case anecdotes.

### What options are recommended at progression following HD IL-2?

For many years additional immunotherapy or clinical trials were the only treatment options. In initial exploratory clinical trials of VEGF and mTOR pathway inhibitors, most of the patients had progressed on prior immunotherapy, which did not have a negative effect on outcome [50, 51]. Therefore, data and clinical experience exist to inform the management of patients following HD IL-2. This decision clearly depends on the timing of progression (immediate vs years later), type and degree of progression, rate of progression, and previous experience with HD IL-2 treatment.

#### Literature review and analysis

Data for proceeding with additional HD IL-2 come from experience in patients for whom this had been their only option. Anecdotal experience has demonstrated subsequent responses to HD IL-2, after a hiatus of time from the initial treatment. Also, long-term follow-up data from IL-2 studies show patients with surgically completed complete responses continue to demonstrate long term remission [52]. Subsequent treatment with anti-angiogenesis agents or mTOR inhibitors has likewise demonstrated benefit in patients who progressed on cytokines [13, 50, 51]. There are limited data on activity of checkpoint pathway inhibitors following treatment with HD IL-2.

#### Consensus recommendations

There was a difference of opinion regarding options at progression, even if response to IL-2 lasted at least 6 months: 73 % would proceed to another therapy, whereas 13 % would recommend another course of HD IL-2. Another 13 % would recommend resection of residual disease if possible to remove all such disease.

In a follow-up discussion, the consensus was that patients who have major response to 2 courses of IL-2, who have residual oligometastatic disease should be managed with surgical resection of residual disease (73 %), another course of IL-2 (20 %), or switch to TKI (7 %). All data was considered anecdotal, and therefore, clinical judgment is the deciding factor at this time.

### What is the role of Low dose IL-2 or low dose IL-2 combined with IFN?

Low dose regimens have been studied in the past, including low dose intravenous (IV) administration on the same schedule as HD IL-2, low dose subcutaneous (SQ) administration, 5 days/week for indeterminate time frame, a decrescendo dosing schedule of SQ IL2, and SQ administration of both low dose IL-2 and IFN, among others. Although durable complete responders have been documented with all of these regimens, the ORR is lower than with HD IL-2 in the IV bolus dose and the SQ injection schedule [53, 54].

#### Literature review and analysis

A low dose IV regimen of IL-2 was noted to yield durable CRs in some patients, albeit in smaller numbers, and this regimen was safe in patients with organ dysfunction [53–55]. Additionally, studies have been reported in which alternate schedules of HD IL-2 have been utilized, and appear to be more tolerable with similar efficacy [56, 57]. These should be further evaluated, particularly in the context of combinations.

#### Consensus recommendations

All agreed that there is limited to no role of either low dose IL-2 regimen as a single agent treatment, with the possible exception of patients with impaired organ function based on a prospective, uncontrolled trial (Level B evidence) [55]. Level A efficacy data favoring HD IL-2 compared to low dose IL-2 was based on two randomized, comparative studies [53, 54]. Level B data on new schedules was derived from prospective, uncontrolled trials [56, 57]. Investigation of low dose regimens in conjunction with new immunotherapies is a research consideration, given that check point pathway inhibitors are being studied at much lower doses in combination than those used in the original single agent trials. Alternative schedules should also be explored in the context of combination immunotherapy or immunotherapy with targeted agents.

### What is the role of HD IL-2 as second-line therapy after anti-VEGF TKI in a patient who met eligibility criteria for HD IL-2 and was not progressing rapidly?

More commonly in the past 10 years, patients with mRCC are started on an anti-VEGF TKI and upon progression are referred for consideration of immunotherapy with HD IL-2 to an institution with such a treatment program. The Task Force was asked to consider the pros and cons of this approach in terms of optimizing treatment options for patients, as well as tolerability of this approach.

#### Literature review and analysis

Cho et al. reported a small experience in which 40 % of 15 patients treated with prior TKI treatment had unexpected cardiac toxicity upon treatment with HD IL-2 [58]. They noted that patients generally had very brief “wash out” periods after completing treatment with anti-VEGF TKIs. Lam et al. subsequently reported the successful administration of HD IL-2 after anti-VEGF TKIs have recommended doing so in the setting of a prolonged break between therapies [59]. They did, in fact, note unexpected grade 3 cardiac events in 6/40 patients who were treated after a short interval. Both reports recommend 8–12 weeks before initiating HD IL-2 therapy [58, 59].

#### Consensus recommendations

Sixty-seven percent of the Task Force felt that anti-PD-1 agents will be the preferred second-line immunotherapy in this setting, following initial anti-VEGF TKI. This is not based on comparative data with other immunotherapy, but it is based on the logistics of outpatient therapy of anti-PD-1 and less stringent eligibility criteria. This second-line position of anti-PD-1 agents is now supported by Level A data from the recently published randomized phase III trial of nivolumab versus everolimus in the second-line setting [16].

Currently, if anti-PD-1 agents are not available for use, then HD IL-2 should be considered as second-line therapy after a washout period in appropriate patients based on Level C data [58, 59]. Such patients should be evaluated carefully with a cardiac echo and show adequate cardiac function prior to initiation of IL-2 therapy.

### What is the role of HD IL-2 after investigational treatment with an anti-PD-1 agent?

Data are only now being compiled by the PROCLAIM registry for centers treating with HD IL-2 and thus no substantial data are yet available.

#### Literature review and analysis

There are no prospective studies. However, there is a single abstract reporting the outcome of patients treated with HD IL-2 after progression on anti-PD-1 checkpoint inhibitors. A small report using the PROCLAIM database and a single institution reported on 11 patients, 7 of whom had mRCC. All developed ongoing SD or response with a median follow-up of 15 months [60].

#### Consensus recommendations

There was no formal vote on this topic. However, the Task Force’s assessment was that HD IL-2 could follow anti-PD-1 agents based on their lower toxicity profile, which is associated with fewer persistent immune-related adverse events compared with other checkpoint inhibitors (e.g., anti-CTLA-4 agents).

Informally, 73 % felt that this sequence is a consideration, as the two immunotherapy approaches work by different mechanisms of immune activation and that anti-PD-1 and IL-2 could potentiate the activity of each other. Some of the Task Force members have done this successfully. The level of evidence for sequencing is currently Level C. A prospective trial of HD IL-2 following anti-PD-1 therapy was felt to be worth consideration.

#### Summary of HD IL-2 recommendation

Eligible patients (clear cell histology with adequate organ reserve, s/p nephrectomy, with few adverse risk features) should be considered for IL-2 therapy at centers with adequate experience. The utility and role of IL-2 prior to or after checkpoint inhibitors is unknown and requires further study.

### What is the role of IFN in the treatment of RCC?

IFN has been a mainstay in the treatment of RCC for more than 20 years and has been the control arm for the initial clinical trials that led to the approval of anti-VEGF and mTOR targeted therapies [1, 5, 10, 12]. IFN has anti-proliferative activity, as well as immune stimulatory activity, with activation of cellular immunity. Continued research provides insight into interactions with signaling pathways for gene transcription, apoptosis, and immune interactions with Toll-like receptors among others [61, 62]. IFN has produced CRs in patients with mRCC, both in the cytokine era and more recently, following anti-VEGF therapy [1, 5]. Nevertheless, it is a difficult drug to use because of the chronic administration as well as the severity and chronicity of side effects.

#### Literature review and analysis

IFN is currently approved in combination with bevacizumab for treatment of patients with mRCC, based on the results of two phase III trials comparing the combination to IFN alone [14, 15, 63, 64]. In these studies, the combination had a better response rate (26–31 %) compared to IFN alone (13 %) and a prolonged progression-free survival (PFS) compared with IFN (8.5–10.4 months versus 5.2–5.4 months). OS was prolonged in both arms, and approached 2 years. The lack of difference in OS between arms was thought to be in part due to subsequent therapy given to patients in both arms after progression.

A subsequent multicenter, phase II trial was conducted, built on the initial phase III bevacizumab/IFN data, taking into account the frequent dose reductions of IFN observed in those studies [64]. This study utilized a reduced dose of IFN (3 MIU 3×/week versus 9 MIU 3×/week). Compared with the data from the initial phase III trials, there was reduced IFN-related toxicity without compromising efficacy [64]. The response rate was 28 %, the median PFS was 15.3 months, and OS was 30.7 months.

There appears to be additive benefit for IFN in combination with bevacizumab, and studies of lower dose IFN appear to provide a manageable regimen in combination. There is wider IFN usage outside of North America.

#### Consensus recommendations

Most members of the Task Force do not use IFN, even in combination with bevacizumab (60 %) and even at lower IFN doses, which evolved in the randomized trials and then was formally evaluated [14, 15, 63–65]. The efficacy recommendation for single agent IFN is level A, based on prospective, randomized trials showing that anti-VEGF receptor and mTOR inhibitor targeted therapies have superior PFS compared to single agent IFN [10, 12]. The level of evidence for IFN in combination with bevacizumab being superior to IFN alone is Level A, based on two randomized, controlled clinical trials [14, 15, 63, 65]. Among the members of the Task Force, only 13 % would use IFN as a single agent.

### What is the role of PD-1 blockade (either with anti-PD-1 or anti-PD-L1)?

The PD-1 pathway is a checkpoint for immune regulation and suppression at the level of the tumor and immune cell interaction [66]. Inhibition of this pathway leads to immune activation. Agents that are under investigation include antibodies to PD-1 and PD-L1 (Table 2), and clinical trials have demonstrated anti-tumor benefit including in mRCC [67–69]. Two such agents have been approved for the treatment of melanoma (nivolumab and pembrolizumab) and more recently for non-small cell lung cancer, RCC (nivolumab), urothelial cancer (atezolizumab), Hodgkin Disease (nivolumab). Nivolumab was approved for mRCC following progression on a VEGFR targeted therapy by the FDA in 2015. This approval was based on Level A evidence in a randomized, phase III controlled trial demonstrating an OS benefit of nivolumab compared with everolimus following progression on anti-VEGFR TKI. There are no comparative data between immunotherapies at this time. The approval of PD-1 pathway blockers in RCC will necessitate further study of sequencing and combination therapy approaches in this disease, involving immunotherapies and VEGF pathway targeted therapies. Many such trials are ongoing.

**Table 2:** Select immunotherapy agents and ongoing immunotherapy clinical trials in RCC

Ongoing clinical trials for check point inhibitors			
Trial	National clinical trial identifier	Status	Disease setting
Neoadjuvant durvalumab +/− tremelimumab	NCT02762006	Recruiting	Neoadjuvant
Neoadjuvant pembro	NCT02212730	Recruiting	Neoadjuvant
Neoadjuvant nivolumab	NCT02595918	Recruiting	Neoadjuvant
Neoadjuvant nivolumab	NCT02575222	Recruiting	Neoadjuvant
Nivo vs. nivo + bev vs. nivo + ipi	NCT02210117	Recruiting	Neoadjuvant
Nivo pre and post-surgery	NCT02446860	Recruiting	Neoadjuvant/adjuvant
Phase I pembro + pazopanib	NCT02014636	Recruiting	Refractory
Phase III nivo vs. everolimus	NCT01668784	Stopped early and reported in 2015	Refractory
Nivo + sunitinib or pazopanib or ipi	NCT01472081	Active, not recruiting	Refractory
Pembro + RT	NCT02318771	Recruiting	Refractory
Phase Ib/II pembro + len in solid tumors	NCT02501096	Recruiting solid tumors including RCC	Refractory
Ongoing IL-2 based clinical trials			
Trial	National clinical trial identifier	Status	
HD IL-2 + HQ	NCT01550367	Recruiting	
IL-2 +/− SBRT	NCT02306954	Recruiting	
IL-2 +/− RT	NCT01896271	Recruiting	
PROCLAIM	NCT01415167	Registry of HD IL-2 patients	
IL2 + entinostat	NCT01038778	Ongoing, presented 2016	

*Abbreviations*: *Ipi* ipilimumab, *nivo* nivolumab, *atezo* atezolimumab, *bev* bevacizumab, *pembro* pembrolizumab, *len* lenvatinib, *HQ* hydroxychloroquine, *SBRT* stereotactic body radiation therapy, *RT* radiation therapy

#### Literature review and analysis

Current longitudinal data for anti-PD-1 agents in mRCC include a phase II trial of nivolumab, evaluating 3 different dose levels. There did not appear to be a dose response in this study, and responses were observed at all three dose levels with an ORR of 21 % and median PFS of 4 months [70]. Another report provided long-term follow-up of the expansion cohort of mRCC patients treated with nivolumab in the initial phase I study, in which 34 treatment-refractory mRCC patients were enrolled [67, 71]. The response rate was 29 % with a median response duration of 12.9 months, and there were 9 additional patients (27 %) with stable disease lasting beyond 24 weeks. The median OS of all patients was 22.4 months [71]. In a recent update, 3 and 5-year survival for this patient population was reported to be 41 and 34 % respectively [72]. This data led to a phase II trial (NCT01354431), which enrolled 167 patients with VEGR TKI refractory advanced RCC and randomized them to 3 different dose levels of nivolumab administered every 3 weeks [70]. Response rates were 20–22 % for each dose level and median OS ranged from 18 to 25 months. Updated data were recently presented at ASCO 2016. At a minimum follow-up of 38 months ORR was 21 % and the median duration of response was 22 months. In addition, the 3-year OS rate was 35 % [72].

Recently, results of the phase III clinical trial of nivolumab versus everolimus in second-line treatment of mRCC were released [16]. The study was stopped early in July 2015 because data demonstrated a median OS benefit in patients receiving nivolumab at 25 months compared with everolimus at 19 months, hazard ratio 0.73, *p =* 0.002 [16]. Additionally, the objective response rate for nivolumab was 25 % compared to 5 % for everolimus (*p <* 0.001). Median PFS was 4.6 months with nivolumab and 4.4 months with everolimus, *p =* .11. Grade 3 or 4 adverse events deemed related to treatment occurred in 19 % of nivolumab-treated patients and in 37 % of everolimus-treated patients. This study also evaluated tumor expression of PD-L1 as a potential biomarker of treatment effect, with cut-off values at ≥ 1 % and ≥ 5 %. While expression of PD-L1 correlated with poorer outcome, it did not predict better response to or survival with nivolumab compared with everolimus, as patients with both high and low PD-L1 expressing tumors appeared to benefit from nivolumab relative to everolimus [16]. This report led to FDA approval of nivolumab for mRCC as second line therapy following a VEGFR inhibitor.

A phase I study of the anti-PD-L1 agent, atezolimuzab reported increased anti-tumor activity in patients whose tumor-infiltrating lymphocytes demonstrated PD-L1 expression. Additionally, this study showed a response rate of 22 % among patients with clear cell RCC with Fuhrman grade 4 or sarcomatoid features [73]. It should be noted, however, that given the low expression of PD-L1 in RCC, 5 out of 9 responders had low PD-L1 expression, highlighting the limited value of assessment of PD-L1 expression for clinical decision making in patients with mRCC.

Combination studies of anti-CTLA-4 and anti-PD-1 have been reported in melanoma with high response rate and high toxicity rate [74, 75]. This approach is currently undergoing considerable modification in terms of dose and schedule and clinical trials of combinations are ongoing in a variety of diseases, including mRCC (Table 2). Studies of combinations of PD-1 pathway blockers and anti-VEGF pathway agents are also ongoing (Table 2 and Table 3).

**Table 3:** Ongoing phase III studies in front-line advanced/metastatic RCC

Study	Primary endpoint	Sample size	National clinical trial identifier	Start time/status
Nivolumab + ipilimumab vs. sunitinib	PFS, OS	1070 (1:1)	NCT02231749	Oct 2014/on-going/enrollment closed
Atezolizumab + bevacizumab vs. sunitinib	PFS, OS	900 (1:1)	NCT02420821	May 2015/on-going
Avelumab + axitinib vs. sunitinib	PFS,	583 (1:1)	NCT02684006	March 2016/on-going
Pembrolizumab + axitinib vs. sunitinib	PFS, OS	840 (1;1)	NCT02853331	Sept 2016
Pembrolizumab + lenvatinib or everolimus + lenvatinib vs. sunitinib	PFS	735 (1:1:1)	NCT02811861	Sept 2016

#### Consensus recommendations

At the time of the meeting, phase III studies were not yet reported and the Task Force discussed the role of PD-1 pathway blockade in mRCC in light of the available phase I and II data. There was enthusiasm for this approach as a single agent, as well as for investigation in combination with other checkpoint pathway inhibitors (anti-CTLA-4) and with activating cytokines (IL-2). The Task Force did vote on their preferred treatment for patients who have progressed on anti-VEGF TKI therapy, in the setting of a patient who had received sunitinib for one year, pazopanib for 8 months, and who remained with ECOG performance status 1. Sixty-seven percent preferred anti-PD-1 agents in clinical trials or as a commercial agent, if available. Thirteen percent would choose IL-2 in appropriate patients after TKIs, and 6.7 % would recommend either axitinib or everolimus. Due to their more favorable toxicity profiles, many patients as well as physicians would likely prefer immunotherapy with anti-PD-1 agents compared to HD IL-2.

There was considerable enthusiasm for enrolling patients into ongoing clinical trials of anti-PD-1 agents in combination therapy. This was preferred even in the first-line setting where several clinical trials are available (Table 3). The utilization of expression of PD-L1 as a biomarker of potential activity of these agents is still under investigation and not established.

Critical questions regarding checkpoint inhibitor therapy include the value (risk/benefit ratio) of combination therapy over single agent use, the ability to stop and restart therapy (i.e., the need for ongoing treatment), the development of biomarkers to select patients, and optimizing toxicity management.

### What treatment is recommended for metastatic non-clear cell RCC?

Non-clear cell RCC represents approximately 20–25 % of surgical cases of RCC, and much less of the mRCC population. Papillary RCC is the most common subtype of non-clear cell RCC, representing about 15 % of surgical series. Other subtypes include chromophobe, collecting duct, medullary, translocation, and several hereditary syndromes with unique features. Specific molecular characteristics have been identified separating the various subtypes. However, to date, except for clear cell RCC, this has not yet led to a successful treatable target.

#### Literature review and analysis

Historically, patients with non-clear cell RCC did not frequently respond to treatment with HD IL-2 [2–4]. The HD IL-2 “SELECT” trial included 5 patients with non-clear cell RCC, and none responded [6]. In an unplanned analysis of the outcome of patients in the phase III trial of temsirolimus versus IFN, patients with non-clear cell carcinoma treated with temsirolimus had a major survival advantage compared to those treated with IFN, demonstrating either the effectiveness of temsirolimus or the lack thereof of IFN in non-clear cell subtypes [12, 76].

Although expanded access trials and small studies of targeted therapy suggested some response to anti-VEGF directed therapy, large database reviews report lower response rates and poorer median survival among patients with metastatic non-clear cell RCC compared with clear cell RCC [77–79]. There is a recently opened NCI-sponsored clinical trial for patients with papillary RCC to evaluate a variety of MET-inhibitors, thus targeting a known genomic feature of some papillary RCC tumors (S1500). Whether the newer immunotherapies will have a role in non-clear cell RCC remains to be determined. A case report describes a dramatic and rapid response of a single patient with papillary RCC with sarcomatoid and rhabdoid features to nivolumab [80].

#### Consensus recommendations

The majority of the Task Force felt that HD IL-2 should be reserved for patients with clear cell renal cancer, based on Level A [6] and Level B evidence [2–4]. Data are insufficient regarding the use of checkpoint pathway inhibitors in the non-clear cell RCC population, since very few such patients were entered into the clinical trials of these agents.

There was lack of consensus on the initial treatment recommendation for patients with metastatic non-clear cell RCC. Essentially, the Task Force voted for clinical trials as initial therapy for such patients, provided new agents or approaches have strong rationale for the specific subtype. If a clinical trial is unavailable, then a VEGFR TKI is preferred given results from two small randomized trials showing a slight advantage over mTOR inhibitors in this setting [81, 82].

## Conclusions

Immunotherapy remains an established modality for the treatment of patients with mRCC and continues to produce durable responses in a subset of patients. Patient selection for HD IL2 remains based on clinical criteria. Outcome for HD IL-2 continues to be the gold standard insofar as there are durable complete remissions. The approval of nivolumab in previously treated patients with mRCC and clinical trials with nivolumab and other PD1 pathway blockers are providing new directions for immunotherapy in patients with mRCC and will likely expand the cohort of patients eligible for such therapy. It is not yet clear whether this approach will provide an increased number of responders, although the suggestion of response in patients with more aggressive tumors with anti-PD-L1 therapy is perhaps evidence that HD IL-2 and PD-1 pathway blockade may have some complementary anti-tumor efficacy. However, further research is ongoing, including exploration of combinations, dose and schedule, and potential consideration of studies in the adjuvant setting. The further development of immunotherapy in patients with RCC will provide meaningful benefit, and the goal should be durable CRs comparable to those observed with HD IL-2. Recent data showing substantial 3–5 year survival rates with nivolumab suggest that this goal may quickly become a reality.

## Additional files

Additional file 1: Comments Received during Review. (DOCX 17 kb)
Additional file 2: Task Force Roster. (DOCX 12 kb)
Additional file 3: Annotated Biobliography. (DOCX 225 kb)
Additional file 4: Task Force Pre-meeting Survey Questions. (DOCX 34 kb)

## Abbreviations

CNS: Central nervous system
CR: Complete response
CWG: Cytokine working group
DFS: Disease-free survival
ECOG: Eastern Cooperative Oncology Group
FDA: U.S. Food and Drug Administration
HD IL-2: High dose interleukin-2
IFN: Interferon-alpha
IL-2: Interleukin-2
mRCC: Metastatic RCC
MSKCC: Memorial-Sloan Kettering Cancer Center
mTOR: Mammalian target of rapamycin
ORR: Overall response rate
OS: Overall survival
PD-1: Programmed death receptor 1
PD-L1: Programmed death ligand 1
PR: Partial response
RCC: Metastatic renal cell cancer
RFS: Recurrence-free survival
SD: Stable disease
SITC: Society for immunotherapy of cancer
SQ: Subcutaneous
TKI: Tyrosine kinase inhibitor
VEGF: Vascular endothelial growth factor
VEGFR: Vascular endothelial growth factor receptor
