# Supplementary material for: Society for Immunotherapy of Cancer consensus statement on immunotherapy for the treatment of renal cell carcinoma
Source: J Immunother Cancer. 2016 Nov 15;4:81. doi: 10.1186/s40425-016-0180-7 (PMC5109802; doi:10.1186/s40425-016-0180-7)
Supplement: Additional file 1: — Comments Received during Review. (DOCX 17 kb) [file 40425_2016_180_MOESM1_ESM.docx]

| **Comment Date** | **Comment** |
| --- | --- |
| May 12, 2016 | Please consider that the role of radiation and radiosurgery in stage IV renal cell cancer is not limited to radiosurgery of brain metastases. As noted in the current NCCN guidelines, bone metastases occur in 30 to 40% of advanced renal cell CA patients. These bone metastases are typically lytic, painful, and carry risks of fracture, hypercalcemia, and cord compression (NCCN.org). A rapid course of stereotactic radiation therapy in 1 to 5 fractions can achieve rapid pain relief within 1 to 2 weeks which is durable for months, with a 3 year local progression-free survival of 88% after single fraction radiosurgery (1,2). For patients not amenable to surgical resection of the primary tumor or oligometastases, stereotactic body radiation therapy (SBRT) can provide effective yet non-invasive local control. In a metanalysis of SBRT for extracranial metastases of RCC, 389 patients had 730 targets treated, producing a local control rate of 89% and overall survival ranging from 11.7 to 22 months, depending on the sub-study (3). Thank you for your consideration of spine and body radiosurgery as a local modality that is well tolerated with low procedural risk for patients with stage IV renal cell carcinoma.  REFERENCES:  1. Spine SBRT: Hunter GK, Balagamwala EH, Koyfman SA, et al. The efficacy of external beam radiotherapy and stereotactic body radiotherapy for painful spinal metastases from renal cell carcinoma. Practical Radiation Oncology 2012;2:e95-e100.  2. Spine Radiosurgery: Zelefsky MJ, Greco C, Motzer R, et al. Tumor control outcomes after hypofractionated and single-dose stereotactic image-guided intensity-modulated radiotherapy fo r extracranial metastases from renal cell carcinoma. Int J Radiat Oncol Biol Phys 2012;82:1744-1748.  3. Extracranial SBRT: Kothari G1, Foroudi F, Gill S, Corcoran NM, Siva S. Outcomes of stereotactic radiotherapy for cranial and extracranial metastatic renal cell carcinoma: a systematic review. Acta Oncol. 2015 Feb;54(2):148-57. Epub 2014 Aug 20. |
| June 21, 2016 | Renal Cell carcinoma accounts for the majority of the diagnosed kidney cancers and its resistance to chemotherapy and radiotherapy is known. Targeted therapy has become a first line treatment in the past years, and development of additional targeted therapies and immunotherapy strategies is extremely important in the fight against RCC. - The paper successfully fulfills the Task Force’s specific aim, to compose the “Consensus Statement on Tumor Immunotherapy for the Treatment of Renal Cell Carcinoma”. Research on the treatments for renal cell carcinoma is now being extensively done, especially on the [causes](http://www.cancer.org/ssLINK/kidney-cancer-adult-what-causes), [detection](http://www.cancer.org/ssLINK/kidney-cancer-adult-detection), [diagnosis](http://www.cancer.org/ssLINK/kidney-cancer-adult-diagnosis) and [treatment](http://www.cancer.org/ssLINK/kidney-cancer-adult-treating-general-info) of kidney cancer. Four general topics on the clinical application of cancer immunotherapy: 1) patient eligibility considerations, 2) toxicity assessment and management, 3) assessment of response, and 4) therapy sequencing and combinations still lack some missing knowledge. The present “Cancer Immunotherapy Guidelines” seek to address these knowledge gaps in order to outline the current data for immunotherapy in RCC and to develop consensus recommendations as a guidance for practicing clinicians caring for patients with RCC. As kidney cancer responds usually well to various immunotherapies, [clinical trials](http://www.cancer.org/ssLINK/clinical-trials-landing) of many new immunotherapy strategies are being tested. I would recommend essential information and listing of Renal Cell Carcinoma medical research trials, actively recruiting patient volunteers as a helpful addition to the Guidelines.This work has become an evidence-based manuscript and its guidelines will certainly be an asset for practicing oncologists. Application of cancer immunotherapy strategies need careful, investigation-based guiding principles. The present Consensus Statement perfectly meets its other goal, namely to take a step further by providing consensus recommendations specifically for the use of cancer immunotherapy in the majority of kidney cancers. The Consensus Statement could develop successfully the guidelines, as a result of a complex process gathering the unbiased consensus recommendations to enhance clinical decision-making regarding cancer immunotherapy. It would be advantageous to determine the time intervals when the guidelines will be reevaluated and updated. New therapeutic achievements in the field or unforeseen complications of the therapeutics have to be considered separately. In the paragraph of “Literature Review and Analysis”/ page 9 it would be of importance to describe in more details the prospective biomarker validation project and how consideration of “poor” risk and “favourable” patients’ selection influence the therapeutic success. Some more precise description about the “non predictive” biomarkers and the exact expression rates of favourable PD-L1 positivity in the tumor might be useful. It would be important to mention some clinical experiences and scientific information concerning autoimmune side effects eventually coupled to the immunotherapeutic strategies. In total, the Cancer Immunotherapy Guidelines for the Treatment of Renal Cell Carcinoma is of high scientific and clinical value, and helps to harness new immunotherapeutic strategies for the patients’ benefit. |
