# Supplementary material for: Society for Immunotherapy of Cancer consensus statement on immunotherapy for the treatment of renal cell carcinoma
Source: J Immunother Cancer. 2016 Nov 15;4:81. doi: 10.1186/s40425-016-0180-7 (PMC5109802; doi:10.1186/s40425-016-0180-7)
Supplement: Additional file 2: — Task Force Roster. (DOCX 12 kb) [file 40425_2016_180_MOESM2_ESM.docx]

**APPENDIX I Task Force Roster**

**Steering Committee:**

Michael B. Atkins, MD Georgetown-Lombardi Comprehensive Cancer Center

Brian Rini, MD Cleveland Clinic Taussig Cancer Institute

**Task Force Participants:**

William Bro Kidney Cancer Association

Ron M. Bukowski, MD Cleveland Clinic Taussig Cancer Institute

Bernard Faba Patient Cleveland Clinic Taussig Cancer Institute

Jo Faba Patient Advocate Cleveland Clinic Taussig Cancer Institute

Robert A. Figlin, MD, FACP Cedars-Sinai Medical Center

Hans Hammers, MD, PhD Johns Hopkins Sidney Kimmel Comprehensive Cancer Center

Thomas Hutson, DO, PharmD Charles A. Sammons Cancer Center, Baylor University Medical Center

Eric Jonasch, MD M.D. Anderson Cancer Center

Richard W. Joseph, MD Mayo Clinic

Bradley Leibovich, MD, FACS Mayo Clinic

David F. McDermott, MD Beth Israel Deaconess Medical Center

Tom Olencki, DO The Ohio State University Medical Center

Allan J. Pantuck, MD UCLA Institute of Urologic Oncology

David I. Quinn, MD, PhD, MBBS, FRACP Kenneth J. Norris Comprehensive Cancer Center, University of Southern California

Virginia Seery, MSN, RN, ANP-BC Beth Israel Deaconess Medical Center

Martin H. Voss, MD Memorial Sloan-Kettering Cancer Center

Christopher G. Wood, MD M.D. Anderson Cancer Center

Laura S. Wood, RN, MSN, OCN^®^ Cleveland Clinic Taussig Cancer Institute
