# Supplementary material for: Society for Immunotherapy of Cancer consensus statement on immunotherapy for the treatment of renal cell carcinoma
Source: J Immunother Cancer. 2016 Nov 15;4:81. doi: 10.1186/s40425-016-0180-7 (PMC5109802; doi:10.1186/s40425-016-0180-7)
Supplement: Additional file 3: — Annotated Biobliography. (DOCX 225 kb) [file 40425_2016_180_MOESM3_ESM.docx]

**Cancer Immunotherapy
Guidelines
(Kidney)**

**An Annotated Bibliography
of the Literature (in order of topic)**

**Society for Immunotherapy of Cancer**

**September 24, 2014**

Table of Contents

[TOPIC: [Kidney Cancer or Renal Cancer] and Immunotherapy 4](#_Toc399347281)

[TOPIC: [Kidney Cancer or Renal Cancer] and Interferon 74](#_Toc399347282)

[TOPIC: [Kidney Cancer or Renal Cancer] and IL-2 155](#_Toc399347283)

[TOPIC: [Kidney Cancer or Renal Cancer] and Anti-PD-1 181](#_Toc399347284)

[TOPIC: [Kidney Cancer or Renal Cancer] and Anti-PD-L1 183](#_Toc399347285)

**Note: The bibliography is organized in topic order and then in alphabetical order by author. Duplicates have been removed in this bibliography.**

**Note: The bibliography is organized in topic order and then in alphabetical order by author. Duplicates have been removed in this bibliography. The search was conducted on 9/24/14 in the sequence and with the limits as follows:**

| **Kidney Cancer or Renal Cancer Immunotherapy Literature Searches Conducted September 24, 2014** | | | | | | | | | | |
| --- | --- | --- | --- | --- | --- | --- | --- | --- | --- | --- |
| **Seach Terms** | **Date Limits** | **Limits** | **Search Field Tags** | **Date Search Completed** | **Total Refs Identified** | **File Name with Dupes/File Name without Dupes** | **EndNote record numbers** | **total records found** | **total # dupes** | **Resulting # of records in bibliography** |
| **[kidney cancer OR renal cancer] AND immunotherapy** | **2004-2014** | ("kidney neoplasms"[MeSH Terms] AND "kidney neoplasms"[MeSH Terms]) AND "immunotherapy"[MeSH Terms] AND ((Clinical Trial[ptyp] OR Controlled Clinical Trial[ptyp] OR Meta-Analysis[ptyp] OR Practice Guideline[ptyp] OR Randomized Controlled Trial[ptyp]) AND ("2004/01/01"[PDAT] : "2014/12/31"[PDAT]) AND "humans"[MeSH Terms]) | MeSH Terms, Publication Type, Publication Date | 9/24/2014 | 116 | **Kidney 092414.enl/ Kidney 092414 w dupes removed.enl** | 1-116 | **116** | 0 | 116 |
| **[kidney cancer OR renal cancer] AND interferon** | **2004-2014** | ("kidney neoplasms"[MeSH Terms] AND "kidney neoplasms"[MeSH Terms]) AND "interferons"[MeSH Terms] AND ((Clinical Trial[ptyp] OR Controlled Clinical Trial[ptyp] OR Meta-Analysis[ptyp] OR Practice Guideline[ptyp] OR Randomized Controlled Trial[ptyp]) AND ("2004/01/01"[PDAT] : "2014/12/31"[PDAT]) AND "humans"[MeSH Terms]) | MeSH Terms, Publication Type, Publication Date | 9/24/2014 | 154 | **Kidney 092414.enl/ Kidney 092414 w dupes removed.enl** | 117-271 | 154 | 26 | 128 |
| **[kidney cancer OR renal cancer] AND IL-2** | **2004-2014** | ("kidney neoplasms"[MeSH Terms] AND "kidney neoplasms"[MeSH Terms]) AND "interleukin-2"[MeSH Terms] AND ((Clinical Trial[ptyp] OR Controlled Clinical Trial[ptyp] OR Meta-Analysis[ptyp] OR Practice Guideline[ptyp] OR Randomized Controlled Trial[ptyp]) AND ("2004/01/01"[PDAT] : "2014/12/31"[PDAT]) AND "humans"[MeSH Terms]) | MeSH Terms, Publication Type, Publication Date | 9/24/2014 | 111 | **Kidney 092414.enl/ Kidney 092414 w dupes removed.enl** | 271-381 | 111 | 69 | 42 |
| **[kidney cancer OR renal cancer] AND anti-PD-1** | **2004-2014** | ("kidney neoplasms"[MeSH Terms] OR "kidney neoplasms"[MeSH Terms]) AND anti-PD-1[All Fields] AND ((Clinical Trial[ptyp] OR Randomized Controlled Trial[ptyp] OR Controlled Clinical Trial[ptyp] OR Meta-Analysis[ptyp] OR Practice Guideline[ptyp]) AND ("2004/01/01"[PDAT] : "2014/12/31"[PDAT]) AND "humans"[MeSH Terms]) | MeSH Terms, Publication Type, Publication Date | 9/24/2014 | 3 | **Kidney 092414.enl/ Kidney 092414 w dupes removed.enl** | 382-384 | 3 | 0 | 3 |
| **[kidney cancer OR renal cancer] AND anti-PD-L1** | **2004-2014** | ("kidney neoplasms"[MeSH Terms] OR "kidney neoplasms"[MeSH Terms]) AND anti-PD-L1[All Fields] AND ((Clinical Trial[ptyp] OR Controlled Clinical Trial[ptyp] OR Randomized Controlled Trial[ptyp] OR Meta-Analysis[ptyp] OR Practice Guideline[ptyp]) AND ("2001/01/01"[PDAT] : "2014/12/31"[PDAT]) AND "humans"[MeSH Terms]) | MeSH Terms, Publication Type, Publication Date | 9/24/2014 | 1 | **Kidney 092414.enl/ Kidney 092414 w dupes removed.enl** | 385 | 1 | 0 | 1 |
|  |  |  |  |  |  |  | Totals | 385 | 95 | 290 |

**NOTE: IN THE BIBLIOGRAPHY, THE NUMBER IN BRACKETS IS THE RECORD NUMBER IN ENDNOTE (e.g., 62 is the record number for the first item in this bibliography). This is the correct number to use for identifying references in the manuscript during the manuscript draft stages.**

# TOPIC: [Kidney Cancer or Renal Cancer] and Immunotherapy

1 [62]. Acres, B. (2007). "Cancer immunotherapy: phase II clinical studies with TG4010 (MVA-MUC1-IL2)." J BUON **12 Suppl 1**: S71-75.

Vaccines are well known in the context of prevention of diseases caused by infectious agents. Current research is now aimed at using vaccines to manipulate the immune system to eliminate established diseases, including cancer. Several such immunotherapeutic vaccines are now in clinical trials and are beginning to show clinical benefit. TG4010 is one such vaccine. It incorporates the MUC1 antigen, which is overexpressed in the majority of cancers, into a non-propagative pox viral vector, MVA. A second gene, interleukin-2 is also incorporated into TG4010 as an immune stimulus. The vaccine has been tested in breast, kidney, prostate and lung cancers with encouraging results.

2 [28]. Amato, R. J., R. E. Hawkins, et al. (2010). "Vaccination of metastatic renal cancer patients with MVA-5T4: a randomized, double-blind, placebo-controlled phase III study." Clin Cancer Res **16**(22): 5539-5547.

PURPOSE: The TroVax Renal Immunotherapy Survival Trial was a randomized, placebo-controlled phase III study that investigated whether modified vaccinia Ankara encoding the tumor antigen 5T4 (MVA-5T4) prolonged survival of patients receiving first-line standard-of-care (SOC) treatment for metastatic renal cell cancer. EXPERIMENTAL DESIGN: Patients with metastatic clear cell renal cancer, prior nephrectomy, and good or intermediate prognosis were randomized 1:1 to receive up to 13 immunizations of MVA-5T4/placebo in combination with either sunitinib, interleukin-2 or interferon-alpha. The primary end point was overall survival. Secondary end points included progression-free survival, overall response rate, and safety. RESULTS: Seven hundred thirty-three patients were recruited (365 MVA-5T4 and 368 placebo). Treatment arms were well balanced for SOC and prognosis. No significant difference in the incidence of adverse events or serious adverse events was observed. No significant difference in overall survival was evident in the two treatment arms (median 20.1 months MVA-5T4 versus 19.2 months placebo; P = 0.55). The magnitude of the 5T4-specific antibody response induced by vaccination with MVA-5T4 was associated with enhanced patient survival. Furthermore, exploratory analyses suggested a number of pretreatment hematologic factors that could identify patients who derive significant benefit from this vaccine. CONCLUSION: MVA-5T4 in combination with SOC was well tolerated, but no difference in survival was observed in the overall study population. Exploratory analyses indicate that there may be subsets of patients who could gain significant benefit from MVA-5T4, but such results would need to be confirmed in future randomized clinical studies.

3 [6]. Amato, R. J., A. Shetty, et al. (2013). "A phase I study of folate immune therapy (EC90 vaccine administered with GPI-0100 adjuvant followed by EC17) in patients with renal cell carcinoma." J Immunother **36**(4): 268-275.

This is the first phase I, open-label study to assess the safety, pharmacokinetics, and antitumor activity of a novel immunotherapeutic regimen known as Folate Immune (EC90 vaccine administered with GPI-0100 adjuvant followed by EC17, a folate-targeted hapten immunotherapy that targets folate receptor expressing cancer cells), which is designed to convert poorly immunogenic tumors to highly immunogenic tumors in patients with metastatic renal cell carcinoma. Three to 6 patients were enrolled in each cohort. In the vaccination phase, patients were given once weekly vaccinations of 0.2 mg of EC90 plus 3.0 mg of GPI-0100 for 3-5 weeks. In the treatment phase, patients were treated with 0.031, 0.092, or 0.276 mg/kg of EC17, 5 d/wk, for weeks 3, 4, or 6. Forty-one patients were enrolled in the study of which 33 patients received >/=1 treatment of EC17. Two dose-limiting toxicities were observed including grade 4 anaphylaxis and grade 3 pancreatitis. During the vaccination phase, mild to moderate injection site reactions were the most frequently reported adverse events. During the treatment phase, transient hypersensitivity reactions were the most common adverse event. Partial response was noted in 4% (1/28) of patients, and stable disease was noted in 54% (15/28) of patients after cycle 1 and was maintained in the majority of patients entering the extension phase of the study. EC90 vaccine with GPI-0100 adjuvant followed by EC17 is safe and well tolerated. The recommended regimen for further studies is 4 weekly vaccinations with 0.2 mg of EC90 plus 3.0 mg GPI-0100 followed by treatment with 0.3 mg of EC17.

4 [45]. Amato, R. J., W. Shingler, et al. (2009). "Vaccination of renal cell cancer patients with modified vaccinia Ankara delivering the tumor antigen 5T4 (TroVax) alone or administered in combination with interferon-alpha (IFN-alpha): a phase 2 trial." J Immunother **32**(7): 765-772.

Attenuated vaccinia virus, modified vaccinia Ankara (MVA) has been engineered to deliver the tumor antigen 5T4 (TroVax). MVA-5T4 has been evaluated in an open-label phase 2 trial in metastatic renal cell cancer patients in which the vaccine was administered alone or in combination with interferon-alpha-2b (IFN-alpha). The safety, immunologic, and clinical efficacy of MVA-5T4 with or without IFN-alpha was determined. Twenty-eight patients with metastatic renal cell cancer were treated with MVA-5T4 alone (13) or plus IFN-alpha (15). The 5T4-specific cellular and humoral responses were monitored throughout the study. Clinical responses were assessed by measuring changes in tumor burden by computed tomography or magnetic resonance imaging scan. MVA-5T4 was well tolerated with no serious adverse event attributed to vaccination. Of 23 intent-to-treat patients tested for immune responses postvaccination, 22 (96%) mounted 5T4-specific antibody and/or cellular responses. One patient treated with MVA-5T4 plus IFN-alpha showed a partial response for >7 months, whereas an additional 14 patients (7 receiving MVA-5T4 plus IFN and 7 receiving MVA-5T4 alone) showed periods of disease stabilization ranging from 1.73 to 9.60 months. Median progression free survival and overall survival for all intent-to-treat patients was 3.8 months (range: 1 to 11.47 mo) and 12.1 months (range: 1 to 27 mo), respectively. MVA-5T4 administered alone or in combination with IFN-alpha was well tolerated in all patients. Despite the high frequency of 5T4-specific immune responses, it is not possible to conclude that patients are receiving clinical benefit. The results are encouraging and warrant further investigation.

5 [50]. Amato, R. J., W. Shingler, et al. (2008). "Vaccination of renal cell cancer patients with modified vaccinia ankara delivering tumor antigen 5T4 (TroVax) administered with interleukin 2: a phase II trial." Clin Cancer Res **14**(22): 7504-7510.

PURPOSE: The attenuated vaccinia virus modified vaccinia ankara (MVA) has been engineered to deliver the tumor antigen 5T4 (TroVax). TroVax has been evaluated in an open-label phase II trial in metastatic renal cell cancer patients in which the vaccine was administered in combination with interleukin-2 (IL-2). The safety, immunologic, and clinical efficacy of TroVax in combination with IL-2 was determined. EXPERIMENTAL DESIGN: Twenty-five patients with metastatic renal cell cancer were treated with TroVax plus IL-2. 5T4-specific cellular and humoral responses were monitored throughout the study. Clinical responses were assessed by measuring changes in tumor burden by computed tomography or magnetic resonance imaging scan. RESULTS: TroVax was well tolerated with no serious adverse event attributed to vaccination. Of 25 intention-to-treat patients, 21 mounted 5T4-specific antibody responses. Two patients showed a complete response for > 24 months and one a partial response for > 12 months. Six patients had disease stabilization from 6 to > 21 months. Median progression-free survival (PFS) and overall survival (OS) were > 3.37 months (range, 1.50- > 24.76) and > 12.87 months (range, 1.90- > 24.76), respectively. A statistically significant relationship was detected between the magnitude of 5T4-specific antibody responses and PFS and OS. CONCLUSION: TroVax in combination with IL-2 was safe and well tolerated in all patients. The high frequency of 5T4-specific immune responses and good clinical response rate are encouraging and warrant further investigation.

6 [51]. Arai, S., R. Meagher, et al. (2008). "Infusion of the allogeneic cell line NK-92 in patients with advanced renal cell cancer or melanoma: a phase I trial." Cytotherapy **10**(6): 625-632.

BACKGROUND: Renal cell cancer and malignant melanoma are two types of cancer that are responsive to immunotherapy. In this phase I dose-escalation study, the feasibility of large-scale expansion and safety of administering ex vivo-expanded NK-92 cells as allogeneic cellular immunotherapy in patients with refractory renal cell cancer and melanoma were determined. METHODS: Twelve patients (aged 31-74 years) were enrolled, three per cohort at cell dose levels of 1x10(8)/m(2), 3x10(8)/m(2), 1x10(9)/m(2) and 3x10(9)/m(2). One treatment course consisted of three infusions. Eleven patients had refractory metastatic renal cell cancer; one patient had refractory metastatic melanoma. RESULTS: The NK-92 cells were expanded in X-Vivo 10 serum-free media supplemented with 500 U/mL Proleukin recombinant human interleukin-2 (rhIL-2), amino acids and 2.5% human AB plasma. Final yields of approximately 1x10(9) cells/culture bag (218-250xexpansion) over 15-17 days were achievable with >or=80% viability. Infusional toxicities of NK-92 were generally mild, with only one grade 3 fever and one grade 4 hypoglycemic episode. All toxicities were transient, resolved and did not require discontinuation of treatment. One patient was alive with disease at 4 years post-NK-92 infusion. The one metastatic melanoma patient had a minor response during the study period. One other patient exhibited a mixed response. DISCUSSION: This study establishes the feasibility of large-scale expansion and safety of administering NK-92 cells as allogeneic cellular immunotherapy in advanced cancer patients and serves as a platform for future study of this novel natural killer (NK)-cell based therapy.

7 [112]. Arroyo, J. C., F. Gabilondo, et al. (2004). "Immune response induced in vitro by CD16- and CD16+ monocyte-derived dendritic cells in patients with metastatic renal cell carcinoma treated with dendritic cell vaccines." J Clin Immunol **24**(1): 86-96.

Monocyte-derived dendritic cells (mDC) are increasingly used as cancer vaccines. However, human monocytes are a heterogeneous cell population. We showed previously that DC derived from a monocyte subset expressing CD16 (16+mDC) stimulated allogeneic naive T lymphocytes to secrete higher levels of IL-4 than DC derived from regular CD14(high)CD16(-) monocytes (16-mDC). Th1-type responses have been associated with effective antitumor responses, thus the use of mDC containing 16+mDC as cancer vaccines might be disadvantageous. Here, we evaluate the primary and memory immune response elicited in vitro by 16+mDC and 16-mDC in five patients with metastatic renal cell carcinoma vaccinated with autologous mDC pulsed with tumor lysates (TuLy) and keyhole limpet hemocyanin (KLH). After therapy, three of the five patients had stable disease. Surprisingly, patients with longer survival showed the highest amount of peripheral blood CD16+ monocytes. Analysis of KLH-specific antibodies revealed high titers of IgG2 in patients with longer survival. CD4+ T lymphocyte proliferation against KLH and TuLy increased after treatment, and some patients showed an augmented rate of CD4+ T lymphocyte proliferation against KLH (3/5) and TuLy (2/3) when 16+mDC were used as antigen presenting cells (APC). Before treatment, the IFN-gamma/IL-4 ratio against TuLy and KLH was higher when using 16-mDC as APC, but after vaccination four of five patients had an increased ratio for TuLy with 16+mDC. These results suggest that the immune response elicited by 16-mDC and 16+mDC is modified when memory or naive T cells are stimulated, and 16+mDC could favor a stronger and more beneficial antitumoral Th1 memory response in vivo.

8 [101]. Artz, A. S., K. Van Besien, et al. (2005). "Long-term follow-up of nonmyeloablative allogeneic stem cell transplantation for renal cell carcinoma: The University of Chicago Experience." Bone Marrow Transplant **35**(3): 253-260.

Nonmyeloablative allogeneic stem cell transplantation (NST) has considerable activity in patients with metastatic renal cell carcinoma (RCC), although there are limited long-term follow-up data. Between February 1999 and May 2003, 18 patients with metastatic RCC underwent 19 matched-sibling NSTs after conditioning with fludarabine and cyclophosphamide with tacrolimus and mycophenolate mofetil as post-transplant immunosuppression. Among the four objective responses, all were partial and have relapsed with a median response duration of 609 days (range, 107-926). All responders are alive at a median of 41 months. Median overall survival for the entire cohort was 14 months. There were four early treatment-related deaths and one late treatment-related death. Eight patients died from progressive disease and five (28%) from treatment-related mortality. Stratifying transplant outcome as early death, intermediate (no response, no early death), or response, the combination of pre-treatment anemia and decreased performance status, was associated with adverse outcome (P = 0.015) and reduced survival (HR 5.4, 95% confidence interval of 1.4 to 21, P = 0.007). Responders demonstrated prolonged survival compared to nonresponders (P = 0.002). NST leads to durable responses in a minority of metastatic RCC patients. Appropriate patient selection is paramount. Anemia and decreased performance status may enable risk stratification.

9 [32]. Atchison, E., J. Eklund, et al. (2010). "A pilot study of denileukin diftitox (DD) in combination with high-dose interleukin-2 (IL-2) for patients with metastatic renal cell carcinoma (RCC)." J Immunother **33**(7): 716-722.

High-dose (HD) IL-2 is approved to treat renal cell carcinoma (RCC) with modest response rates and significant toxicity. Enhancement of cytotoxic T-cell activity by IL-2 is 1 mechanism of action. IL-2 also stimulates regulatory T lymphocytes (Tregs), which are associated with poor prognosis. Favorable outcomes are associated with greater rebound absolute lymphocyte count (Fumagalli 2003). DD depletes IL-2 receptor (CD25 component) expressing cells. We hypothesized that sequential therapy could complement each other; DD would deplete Tregs so IL-2 could more effectively stimulate proliferation and activity of cytotoxic T lymphocytes. Patients (n=18) received standard HD IL-2 and 1 dose of DD daily for 3 days; periodic flow cytometry and complete blood counts were performed. Group A included 3 patients to assess safety only with DD 6 mug/kg between the IL-2 courses. Group B included 9 patients at 9 mug/kg DD before the IL-2 courses. Group C included 6 patients at 9 mug/kg DD between the IL-2 courses. Efficacy using the RECIST criteria was assessed after the treatment. Fifteen patients from a study of IL-2 without DD served as controls for toxicity comparison and 13 of these for flow cytometry comparisons. No unusual toxicity was noted. For group B/C patients receiving DD, the median decline in Tregs was 56.3% from pre-DD to post-DD (P=0.013). Peak absolute lymphocyte count change from baseline was +9980/muL for group B, +4470/muL for group C, and +4720/muL for the controls (P=0.005 B vs. C). The overall response rate was 5 of 15 (33%); 3 of 9 (33%) and 2 of 6 (33%) for groups B and C, respectively, including 2 patients with sarcomatoid RCC and 1 with earlier sunitinib therapy.

10 [114]. Atzpodien, J., H. Kirchner, et al. (2004). "Interleukin-2- and interferon alfa-2a-based immunochemotherapy in advanced renal cell carcinoma: a Prospectively Randomized Trial of the German Cooperative Renal Carcinoma Chemoimmunotherapy Group (DGCIN)." J Clin Oncol **22**(7): 1188-1194.

PURPOSE: We conducted a prospectively randomized clinical trial to compare the efficacy of three outpatient therapy regimens in 341 patients with progressive metastatic renal cell carcinoma. PATIENTS AND METHODS: Patients were stratified according to known clinical predictors and were subsequently randomly assigned. Treatment arms were: arm A (n = 132), subcutaneous interferon alfa-2a (sc-IFN-alpha-2a), subcutaneous interleukin-2 (sc-IL-2), and intravenous (IV) fluorouracil; arm B (n = 146): arm A treatment combined with per oral 13-cis-retinoic acid; and arm C (n = 63), sc-IFN-alpha-2a and IV vinblastine. RESULTS: Treatment (according to the standard 8-week Hannover Atzpodien regimen) arms A, B, and C yielded objective response rates of 31%, 26%, and 20%, respectively. Arm B, but not arm A, showed a significantly improved progression-free survival (PFS) compared with arm C (P =.0248). Both arm A (median overall survival, 25 months; P =.0440) and arm B (median overall survival, 27 months; P =.0227) led to significantly improved overall survival (OS) compared with arm C (median OS, 16 months). All three sc-IFN-alpha-2a-based therapies were moderately or well tolerated. CONCLUSION: Our results established the safety and improved long-term therapeutic efficacy of sc-IL-2 plus sc-INF-alpha-2a-based outpatient immunochemotherapies, compared with sc-INF-alpha-2a/IV vinblastine.

11 [107]. Avigan, D., B. Vasir, et al. (2004). "Fusion cell vaccination of patients with metastatic breast and renal cancer induces immunological and clinical responses." Clin Cancer Res **10**(14): 4699-4708.

PURPOSE: Dendritic cells (DCs) are potent antigen-presenting cells that are uniquely capable of inducing tumor-specific immune responses. We have conducted a Phase I trial in which patients with metastatic breast and renal cancer were treated with a vaccine prepared by fusing autologous tumor and DCs. EXPERIMENTAL DESIGN: Accessible tumor tissue was disrupted into single cell suspensions. Autologous DCs were prepared from adherent peripheral blood mononuclear cells that were obtained by leukapheresis and cultured in granulocyte macrophage colony-stimulating factor, interleukin 4, and autologous plasma. Tumor cells and DCs were cocultured in the presence of polyethylene glycol to generate the fusions. Fusion cells were quantified by determining the percentage of cells that coexpress tumor and DC markers. Patients were vaccinated with fusion cells at 3-week intervals and assessed weekly for toxicity, and tumor response was assessed at 1, 3, and 6 months after completion of vaccination. RESULTS: The vaccine was generated for 32 patients. Twenty-three patients were vaccinated with 1 x 10(5) to 4 x 10(6) fusion cells. Fusion cells coexpressed tumor and DC antigens and stimulated allogeneic T-cell proliferation. There was no significant treatment-related toxicity and no clinical evidence of autoimmunity. In a subset of patients, vaccination resulted in an increased percentage of CD4 and CD8+ T cells expressing intracellular IFN-gamma in response to in vitro exposure to tumor lysate. Two patients with breast cancer exhibited disease regressions, including a near complete response of a large chest wall mass. Five patients with renal carcinoma and one patient with breast cancer had disease stabilization. CONCLUSIONS: Our findings demonstrate that fusion cell vaccination of patients with metastatic breast and renal cancer is a feasible, nontoxic approach associated with the induction of immunological and clinical antitumor responses.

12 [65]. Avigan, D. E., B. Vasir, et al. (2007). "Phase I/II study of vaccination with electrofused allogeneic dendritic cells/autologous tumor-derived cells in patients with stage IV renal cell carcinoma." J Immunother **30**(7): 749-761.

In the present study, we assessed the feasibility, toxicity, immunologic response, and clinical efficacy of vaccination with allogeneic dendritic cell (DC)/tumor fusions in patients with metastatic renal cell carcinoma (RCC). Patients with stage IV RCC with accessible tumor lesions or independent therapeutic indications for nephrectomy were eligible for enrollment. Tumors were processed into single cell suspensions and cryopreserved. DCs were generated from adherent peripheral blood mononuclear cells isolated from normal volunteers and cultured with granulocyte macrophage colony-stimulating factor, interleukin-4, and tumor necrosis factor-alpha. DCs were fused to patient derived RCC with serial electrical pulses. Patients received up to 3 vaccinations at a fixed dose of 4x10(7) to 1x10(8) cells administered at 6-week intervals. Twenty-four patients underwent vaccination. Twenty-one and 20 patients were evaluable for immunologic and clinical response, respectively. DCs demonstrated a characteristic phenotype with prominent expression of HLA class II and costimulatory molecules. A mean fusion efficiency of 20% was observed, determined by the percent of cells coexpressing DC and tumor antigens. No evidence of significant treatment related toxicity or auto-immunity was observed. Vaccination resulted in antitumor immune responses in 10/21 evaluable patients as manifested by an increase in CD4 and/or CD8 T-cell expression of interferon-gamma after ex vivo exposure to tumor lysate. Two patients demonstrated a partial clinical response by Response Evaluation Criteria in Solid Tumors criteria and 8 patients had stabilization of their disease. Vaccination of patients with RCC with allogeneic DC/tumor fusions was feasible, well tolerated, and resulted in immunologic and clinical responses in a subset of patients.

13 [16]. Baek, S., C. S. Kim, et al. (2011). "Combination therapy of renal cell carcinoma or breast cancer patients with dendritic cell vaccine and IL-2: results from a phase I/II trial." J Transl Med **9**: 178.

BACKGROUND: Ten cancer patients (Six renal cell carcinoma and four breast cancer patients) were treated in a phase I/II study with a vaccine composed of autologous dendritic cells (DCs) and IL-2 to evaluate the DC vaccine-related toxicity and antigen-specific immune alteration. METHODS: Cancer patients were treated twice with autologous CD34+ hematopoietic stem cell-derived, GM-CSF/IFN-gamma-differentiated DCs pulsed with autologous tumor lysate and KLH, by 4-week interval. Following each subcutaneous injection of therapeutic DCs, low-dose (200 MIU) IL-2 was introduced for 14 consecutive days as an immune adjuvant. To determine the DC vaccine-induced immunological alterations, the KLH-specific lymphocyte proliferation, number of IFN-gamma secreting T cells (ELISPOT assay), NK activity and the cytokine modulation were measured. RESULTS: Cultured-DCs expressing HLA-DR, CD11c, CD83, and B7.1/B7.2 produced IL-12p70. After vaccination, the patients tolerated it. Clinical response was observed in one RCC patient as stable disease. However DC-vaccine related antigen-specific immune responses including peripheral blood lymphocyte proliferation and the number of IFN-r secreting cells were induced in six patients without clear correlation with clinical responses. Also NK activity was induced significantly in six patients after vaccination. DC vaccine-related decrease of TGF-beta level or increase of IL-12p70 level and decline of CD4+CD25+ T cells were observed in three patients. However only in the RCC patient whose disease stabilized, combination of stimulatory as well as inhibitory immune alterations including induction of IFN-gamma secreting T cell with reduction of CD4+ CD25+ T cell were correlated with clinical responses. CONCLUSION: Data indicated that DC vaccine combined with IL-2 is well tolerated without major side effects. DC vaccine induced the specific immunity against introduced antigen. Combinatorial alterations of immunological parameters indicating antigen-specific immune induction along with reduction of inhibitory immunity were correlated with clinical responses in DC vaccine treated patients.

14 [82]. Barkholt, L., M. Bregni, et al. (2006). "Allogeneic haematopoietic stem cell transplantation for metastatic renal carcinoma in Europe." Ann Oncol **17**(7): 1134-1140.

BACKGROUND: An allogeneic antitumour effect has been reported for various cancers. We evaluated the experience of allogeneic haematopoietic stem cell transplantation (HSCT) for renal cell carcinoma (RCC) in 124 patients from 21 European centres. PATIENTS AND METHODS: Reduced intensity conditioning and peripheral blood stem cells from an HLA-identical sibling (n = 106), a mismatched related (n = 5), or an unrelated (n = 13) donor were used. Immunosuppression was cyclosporine alone, or combined with methotrexate or mycophenolate mofetil. Donor lymphocyte infusions (DLI) were given to 42 patients. The median follow-up was 15 (range 3-41) months. RESULTS: All but three patients engrafted. The cumulative incidence of moderate to severe, grades II-IV acute GVHD was 40% and for chronic GVHD it was 33%. Transplant-related mortality was 16% at one year. Complete (n = 4) or partial (n = 24) responses, median 150 (range 42-600) days post-transplant, were associated with time from diagnosis to HSCT, mismatched donor and acute GVHD II-IV. Factors associated with survival included chronic GVHD (hazards ratio, HR 4.12, P < 0.001), DLI (HR 3.39, P < 0.001), <3 metastatic sites (HR 2.61, P = 0.002) and a Karnofsky score >70 (HR 2.33, P = 0.03). Patients (n = 17) with chronic GVHD and given DLI had a 2-year survival of 70%. CONCLUSION: Patients with metastatic RCC, less than three metastatic locations and a Karnofsky score >70% can be considered for HSCT. Posttransplant DLI and limited chronic GVHD improved the patient survival.

15 [35]. Bellmunt, J., P. Maroto-Rey, et al. (2010). "A phase II trial of first-line sorafenib in patients with metastatic renal cell carcinoma unwilling to receive or with early intolerance to immunotherapy: SOGUG Study 06-01." Clin Transl Oncol **12**(7): 503-508.

AIMS: Our aim was to evaluate first-line treatment of metastatic renal cell carcinoma (mRCC) with sorafenib in patients unwilling to receive immunotherapy or with early intolerance to immunotherapy. PATIENTS AND METHODS: Patients had clear-cell mRCC with good or intermediate risk status, were unsuited to cytokine therapy due to preference or intolerance (based on <4 weeks prior immunotherapy) and had not received antiangiogenic agents. Patients received sorafenib 400 mg twice daily until disease progression or unacceptable toxicity. The primary endpoint was progression-free survival (PFS). RESULTS: Twenty-six evaluable patients were enrolled at six centres between March and July 2006. The most common metastatic sites were lung and bone; nine patients had one or two metastatic lesions. Median PFS was 7.5 months (95% confidence interval [CI] 5.1-17.5) and overall survival (OS) 15.4 months (95% CI 12.9-17.4). Among 21 patients evaluable for response, 19 (90.5%) experienced disease control (including one complete response; four partial responses; 14 stable disease). The majority of adverse events were grade 1-2 (87.3%). The most common were asthenia (53.0%) and diarrhoea (50.0%). CONCLUSION: In patients with mRCC who were unwilling to receive or intolerant to immunotherapy, treatment with sorafenib led to a high rate of disease control with toxicities that were generally mild and manageable. The PFS achieved in this essentially treatment-naive population compares favourably with that obtained in the randomised first-line phase II study.

16 [57]. Bennouna, J., E. Bompas, et al. (2008). "Phase-I study of Innacell gammadelta, an autologous cell-therapy product highly enriched in gamma9delta2 T lymphocytes, in combination with IL-2, in patients with metastatic renal cell carcinoma." Cancer Immunol Immunother **57**(11): 1599-1609.

PURPOSE: gamma9delta2 T lymphocytes have been shown to be directly cytotoxic against renal carcinoma cells. Lymphocytes T gammadelta can be selectively expanded in vivo with BrHPP (IPH1101, Phosphostim) and interleukin 2 (IL-2). A phase I Study was conducted in patients with metastatic renal cell carcinoma (mRCC) to determine the maximum-tolerated dose and safety of Innacell gammadelta, an autologous cell-therapy product based on gamma9delta2 T lymphocytes, in patients with mRCC. EXPERIMENTAL DESIGN: A 1-h intravenous infusion of gamma9delta2 T lymphocytes was administered alone during treatment cycle 1 and combined with a low dose of subcutaneous interleukin-2 (IL-2, 2 MIU/m2 from Day 1 to Day 7) in the two subsequent cycles (at 3-week intervals). The dose of gamma9delta2 T lymphocytes was escalated from 1 up to 8 x 10(9) cells. RESULTS: Ten patients underwent a total of 27 treatment cycles. Immunomonitoring data demonstrate that gamma9delta2 T lymphocytes are initially cleared from the blood to reappear at the end of IL-2 administration. Dose-limiting toxicity occurred in one patient at the dose of 8 x 10(9) cells (disseminated intravascular coagulation). Other treatment-related adverse events (AEs) included mainly gastrointestinal disorders and flu-like symptoms (fatigue, pyrexia, rigors). Hypotension and tachycardia also occurred, especially with co-administered IL-2. Six patients showed stabilized disease. Time to progression was 25.7 weeks. CONCLUSION: The data collected in ten patients with mRCC indicate that repeated infusions of Innacell gammadelta at different dose levels (up to 8 x 10(9) total cells), either alone or with IL-2 is well tolerated. These results are in favor of the therapeutic value of cell therapy with Innacell gammadelta for the treatment of cancers.

17 [53]. Berntsen, A., R. Trepiakas, et al. (2008). "Therapeutic dendritic cell vaccination of patients with metastatic renal cell carcinoma: a clinical phase 1/2 trial." J Immunother **31**(8): 771-780.

Therapeutic dendritic cell (DC) vaccination against cancer is a strategy aimed at activating the immune system to recognize and destroy tumor cells. In this nonrandomized phase 1/2 trial, we investigated the safety, feasibility, induction of T-cell response, and clinical response after treatment with a DC-based vaccine in patients with metastatic renal cell carcinoma. Twenty-seven patients with progressive cytokine-refractory metastatic renal cell carcinoma were vaccinated with DCs loaded with either a cocktail of survivin and telomerase peptides or tumor lysate depending on their HLA-A2 haplotype, and low-dose IL-2 was administered concomitantly. Tumor response, immune response, and serum IL-6 and YKL-40 were measured during treatment. Vaccine generation was successful in all patients and no serious adverse events were observed. None of the patients had an objective response but 13/27 patients obtained disease stabilization (SD) for more than 8 weeks. An antigen-specific immune response was demonstrated in 6/6 patients tested. Furthermore, significant alterations in serum YKL-40 and IL-6 were found during treatment. In conclusion, DC vaccination in our setting is feasible and without severe toxicity. Almost half of the patients obtained SD, and in more than 1/3 of the patients, SD persisted for more than 6 months. However, the evaluation of SD is difficult to interpret in the absence of a randomized trial and, therefore, these results should be interpreted with caution. Antigen-specific immune responses were observed in a subset of the treated patients.

18 [75]. Bleumer, I., D. M. Tiemessen, et al. (2007). "Preliminary analysis of patients with progressive renal cell carcinoma vaccinated with CA9-peptide-pulsed mature dendritic cells." J Immunother **30**(1): 116-122.

Carbonic anhydrase-IXG250/MN (CA9) is a renal cell carcinoma (RCC)-associated antigen ubiquitously expressed in the clear-cell subtype of RCC. Two CA9-derived peptides have been identified defining a cytotoxic T-lymphocyte epitope and human leukocyte antigen (HLA)-DR epitope, able to induce T-cell responses in vitro. A phase I clinical trial was performed with CA9-peptide-loaded dendritic cells (DCs) in patients with progressive, cytokine-refractory metastatic RCC to assess the safety, toxicity, and induction of CA9-specific immunity. Patients with objective progressive metastatic RCC received 5 vaccinations of mature DCs pulsed with the CA9-derived peptides and keyhole limpet hemocyanine (KLH). Peripheral blood was collected at regular intervals, delayed-type hypersensitivity (DTH) was tested at baseline and after the last vaccination, and skin biopsies of positive DTH sites were collected for immunomonitoring purposes. Patients were also monitored for clinical responses. No significant toxicity was observed. All patients developed humoral responses against KLH, and demonstrated DTH conversion. Evaluation of biopsy material suggested an increased influx of T-helper cells. In none of the immunomonitoring assays was evidence for the induction of CA9-peptide-specific immunity observed. No clinical responses were observed. The vaccination of DCs pulsed with KLH and 2 CA9-derived peptides was well tolerated. The lack of induction of CA9-peptide-specific immune responses indicates that this particular vaccine regimen is poor in inducing CA9-peptide-specific immune responses.

19 [47]. Bregni, M., M. Bernardi, et al. (2009). "Long-term follow-up of metastatic renal cancer patients undergoing reduced-intensity allografting." Bone Marrow Transplant **44**(4): 237-242.

SCT from an HLA-compatible sibling donor is an adoptive immunotherapy for cytokine-refractory, metastatic clear-cell renal cell cancer (RCC). However, the recent introduction of targeted therapy compounds has reduced the interest in this therapeutic strategy. We have reanalyzed our series with the aim to assess long-term benefit from allografting. Twenty-five RCC patients received a reduced-intensity allograft from an HLA-identical sibling donor. All patients received a thiotepa, fludarabine and CY conditioning regimen, and a cyclosporine-based GVHD prophylaxis. Best response to allograft was evaluable in 24 patients: 1 CR, 4 PR, 12 minor response/stable disease, 7 progressive disease. One-year survival was 48%, and five-year survival was 20%. At a median observation time of 65 months, five patients are alive, one in CR, one in PR and three with stable disease. By multivariate analysis, C-reactive protein value before transplant, the number of CD34 + infused cells and disease status at day +90 significantly correlated with survival. Survival of patients at favorable/intermediate-risk according to the MSKCC score that underwent allografting was better in comparison to the survival predicted by historical controls. We conclude that 20% of cytokine-refractory RCC patients are alive long-term after allografting. Transplantation is able to induce long-term disease control in a fraction of relapsed RCC patients.

20 [42]. Brignone, C., B. Escudier, et al. (2009). "A phase I pharmacokinetic and biological correlative study of IMP321, a novel MHC class II agonist, in patients with advanced renal cell carcinoma." Clin Cancer Res **15**(19): 6225-6231.

PURPOSE: To evaluate the safety, tolerability, pharmacokinetics, and pharmacodynamics of IMP321, a recombinant soluble LAG-3Ig fusion protein which agonizes MHC class II-driven dendritic cell activation. EXPERIMENTAL DESIGN: Patients with advanced renal cell carcinoma were treated with escalating doses of IMP321 s.c. Blood samples were assayed to determine plasma pharmacokinetic parameters, detect human anti-IMP321 antibody formation, and determine long-lived CD8 T cell responses. RESULTS: Twenty-one advanced renal cell carcinoma patients received 119 injections of IMP321 at doses ranging from 0.050 to 30 mg/injection s.c. biweekly for 6 injections. No clinically significant adverse events were observed. Good systemic exposure to the product was obtained following s.c. injections of doses above 6 mg. IMP321 induced both sustained CD8 T-cell activation and an increase in the percentage of long-lived effector-memory CD8 T cells in all patients at doses above 6 mg. Tumor growth was reduced and progression-free survival was better in those patients receiving higher doses (>6 mg) of IMP321: 7 of 8 evaluable patients treated at the higher doses experienced stable disease at 3 months compared with only 3 of 11 in the lower dose group (P = 0.015). CONCLUSION: The absence of toxicity and the demonstration of activity at doses above 6 mg warrant further disease-directed studies of IMP321 in combined regimens (e.g., chemoimmunotherapy).

21 [87]. Brouwers, A. H., P. F. Mulders, et al. (2005). "Lack of efficacy of two consecutive treatments of radioimmunotherapy with 131I-cG250 in patients with metastasized clear cell renal cell carcinoma." J Clin Oncol **23**(27): 6540-6548.

PURPOSE: A previous activity dose-escalation study using 131I-labeled chimeric monoclonal antibody cG250 in patients with progressive metastatic renal cell carcinoma (RCC) resulted in occasional therapeutic responses. The present study was designed to determine the safety and therapeutic efficacy of two sequential high-dose treatments with 131I-cG250. PATIENTS AND METHODS: Patients (n = 29) with progressive metastatic RCC received a low dose of (131)I-cG250 for assessment of preferential targeting of metastatic lesions, followed by the first radioimmunotherapy (RIT) with 2220 MBq/m2 131I-cG250 (n = 27) 1 week later. If no grade 4 hematologic toxicity was observed, a second low-dose 131I-cG250 (n = 20) was given 3 months later. When blood clearance was not accelerated, a second RIT of 131I-cG250 was administered at an activity-dose of 1110 MBq/m2 (n = 3) or 1665 MBq/m2 (n = 16). Patients were monitored weekly for toxicity, and tumor size was evaluated by computed tomography once every 3 months intervals. RESULTS: The maximum-tolerated dose (MTD) of the second RIT was 1,665 MBq/m2 because of dose-limiting hematological toxicity. Based on an intention-to-treat analysis, after two RIT treatments, the disease stabilized in five of 29 patients, whereas it remained progressive in 14 of 29 patients. Two patients received no RIT, and eight of 29 received only one 131I-cG250 RIT because of grade 4 hematologic toxicity, formation of human antichimeric antibodies, or disease progression. CONCLUSION: In patients with progressive end-stage RCC, the MTD of the second treatment was 75% of the MTD of the first RIT. In the majority of patients, two cycles of 131I-cG250 could be safely administered without severe toxicity. No objective responses were observed, but occasionally two RIT doses resulted in stabilization of previously progressive disease.

22 [40]. Buchner, A., H. Pohla, et al. (2010). "Phase 1 trial of allogeneic gene-modified tumor cell vaccine RCC-26/CD80/IL-2 in patients with metastatic renal cell carcinoma." Hum Gene Ther **21**(3): 285-297.

Preclinical studies showed that the allogeneic tumor cell line RCC-26 displayed natural immunogenic potential that was enhanced through expression of CD80 costimulatory molecules and secretion of interleukin-2. Here we report the study of RCC-26/CD80/IL-2 cells in a phase 1 vaccine trial of renal cell carcinoma patients with metastatic disease (mRCC). Fifteen patients of the HLA-A*0201 allotype, with at least one metastatic lesion, were included. Irradiated vaccine cells were applied in increasing doses of 2.5, 10, and 40 x 10(6) cells over 22 weeks. Primary study parameters included safety and toxicity. Sequential blood samples were analyzed by interferon-gamma enzyme-linked immunospot assays to detect tumor antigen-associated (TAA) effector cells. The vaccine was well tolerated and the designated vaccination course was completed in 9 of 15 patients. Neither vaccine-induced autoimmunity nor systemic side effects were observed. Delayed-type hypersensitivity skin reactions were detected in 11 of 12 evaluated patients and were particularly strong in patients with prolonged survival. In parallel, vaccine-induced immune responses against vaccine or overexpressed TAA were detected in 9 of 12 evaluated patients. No tumor regressions occurred according to RECIST (Response Evaluation Criteria in Solid Tumors) criteria; however, median time to progression was 5.3 months and median survival was 15.6 months, indicating substantial disease stabilization. We conclude that vaccine use was safe and feasible in mRCC. Clinical benefits were limited in these patients with advanced disease; however, immune monitoring revealed vaccine-induced responses against multiple TAAs in the majority of study participants. These results suggest that this vaccine could be useful in combination therapies and/or minimal residual disease.

23 [33]. Buti, S., S. Lazzarelli, et al. (2010). "Dose-finding trial of a combined regimen with bevacizumab, immunotherapy, and chemotherapy in patients with metastatic renal cell cancer: An Italian Oncology Group for Clinical Research (GOIRC) study." J Immunother **33**(7): 735-741.

The aim of this study was to look for the maximum tolerated dose (MTD) of gemcitabine and 5-fluorouracil in a new regimen also containing the antiangiogenic bevacizumab and immunotherapy (IT) for the treatment of metastatic renal cell cancer. The primary objective of this multicenter dose-finding study was to establish the MTD of chemotherapy (CT) in combination with fixed doses of IT and bevacizumab. The secondary objective was to assess the combination's activity. Five escalated dose levels of CT with intravenous gemcitabine and 5-fluorouracil (days 1 and 8 every 28 d), were associated together with intravenous bevacizumab (10 mg/kg on days 1 and 15 every 28 d), subcutaneous interleukin-2 (1 MIU/m(2) bid on days 8, 9, 15, 16, and 1 MIU/m(2)/d on days 10-12 and 17-19), and interferon-alpha-2a (3 MIU on days 10, 12, 17, 19). Of the 27 enrolled patients, 59% had been pretreated. The MTD was not reached. The highest CT dose studied was gemcitabine 1000 mg/m(2) and 5-fluorouracil 600 mg/m(2). More frequent grade 3 to 4 toxicities included neutropenia (63%), thrombocytopenia (33%), and fever (26%). The response rate was 33% according to the Response Evaluation Criteria in Solid Tumors. This is the first study that explored the feasibility and safety of combined bevacizumab, IT, and CT in metastatic renal cell cancer. The activity of this regimen is interesting and its efficacy warrants further trials.

24 [20]. Cao, S., Y. L. Wang, et al. (2011). "Efficacy of large doses of IL-2-activated human leukocyte antigen haploidentical peripheral blood stem cells on refractory metastatic renal cell carcinoma." Cancer Biother Radiopharm **26**(4): 503-510.

Traditional immunotherapy for patients with refractory metastatic renal cell carcinoma (RCC) is limited because the tumors themselves induce immunosuppression. The aim of this article was to evaluate the clinical efficacy of the infusion of a high dose of interleukin (IL)-2-activated allogeneic haploidentical peripheral blood stem cells (haplo-PBSCs) in patients with advanced intractable RCC. Ten advanced RCC patients and their haploidentical relatives, who were haplo-PBSC donors, were enrolled in this study. All patients accepted one cycle of activated haplo-PBSCs. The clinical and immunologic responses were evaluated. A range from 2.3 to 5.5x10(10) of activated haplo-PBSCs were harvested after exposure to recombinant human IL-2 (rhIL-2), along with a significant increase in the proportion of natural killer cells and activated lymphocytes (CD69+ and CD25+). Enhanced cytotoxicity of haplo-PBSCs for RCC was also observed. After treatment, 2 (2/10) cases of partial remission, 6 (6/10) cases of stable disease, and 2 (2/10) cases of progressive disease were identified in these 10 patients. The median progression-free survival of the 10 patients was 5.5 months (3-14 months). The adoptive transfusion of IL-2-activated haplo-PBSCs can induce sustained antitumor effects for advanced intractable RCC patients who have had no response to conventional immunotherapy.

25 [97]. Coppin, C., F. Porzsolt, et al. (2005). "Immunotherapy for advanced renal cell cancer." Cochrane Database Syst Rev(1): CD001425.

BACKGROUND: The course of advanced renal cell carcinoma is extremely variable, ranging from spontaneous remission to disease progression refractory to chemotherapy. Immunotherapy has held promise of improved outcomes based on uncontrolled studies and randomized controlled trials generally limited by small size and low power. OBJECTIVES: To evaluate immunotherapy for advanced renal cell carcinoma by comparing: (1) high dose interleukin-2 to other options and (2) interferon-alfa to other options. The primary outcome of interest was overall survival at one year, with remission as the main secondary outcome of interest. SEARCH STRATEGY: A systematic search of the CENTRAL, MEDLINE, and EMBASE databases was conducted for the period 1966 through end of December 2003. Handsearches were made of the proceedings of the periodic meetings of the American Urologic Association, the American Society of Clinical Oncology, ECCO - the European Cancer Conference, and the European Society of Medical Oncology for the period 1995 to June 2004. SELECTION CRITERIA: Randomized controlled trials that selected (or stratified) patients with advanced renal cell carcinoma, utilized an immunotherapeutic agent in at least one study arm, and reported remission or survival by allocation. Fifty-three identified studies involving 6117 patients were eligible and all but one reported remission; 32 of these studies reported the one-year survival outcome. DATA COLLECTION AND ANALYSIS: Two reviewers independently abstracted each article by following a prospectively designed protocol. Dichotomous outcomes for treatment remission (partial plus complete) and for deaths at one year were used for the main comparisons. Survival hazard ratios were also used for studies of interferon-alfa versus controls, and for two randomized studies of the value of initial nephrectomy prior to interferon-alfa in fit patients with metastases detected at the time of diagnosis. MAIN RESULTS: Combined data for a variety of immunotherapies gave an overall chance of partial or complete remission of only 12.9% (99 study arms), compared to 2.5% in 10 non-immunotherapy control arms, and 4.3% in two placebo arms. Twenty-eight percent of these remissions were designated as complete (data from 45 studies). Median survival averaged 13.3 months (range by arm, 6 to 27+ months). The difference in remission rate between arms was poorly correlated with the difference in median survival so that remission rate is not a good surrogate or intermediate outcome for survival for advanced renal cancer. We were unable to identify any published randomized study of high-dose interleukin-2 versus a non-immunotherapy control, or of high-dose interleukin-2 versus interferon-alfa reporting survival. It has been established that reduced dose interleukin-2 given by intravenous bolus or by subcutaneous injection provides equivalent survival to high dose interleukin-2 with less toxicity. Results from four studies (644 patients) indicate that interferon-alfa is superior to controls (OR for death at one year = 0.56, 95% confidence interval 0.40 to 0.77). Using the method of Parmar 1998, the pooled overall hazard ratio for death was 0.74 (95% confidence interval 0.63 to 0.88). The weighted average median improvement in survival was 3.8 months. T he optimal dose and duration of interferon-alfa remains to be elucidated. The addition of a variety of enhancers, including lower dose intravenous or subcutaneous interleukin-2, has failed to improve survival compared to interferon-alfa alone. Two recent randomized studies have examined the role of initial nephrectomy prior to interferon-alfa therapy in highly selected fit patients with metastases at diagnosis and minimal symptoms: despite minimal improvement in the chance of remission, both studies of up-front nephrectomy improved median survival by 4.8 months over interferon-alfa alone. Recent studies have been examining anti-angiogenesis agents. A landmark study of bevacizumab, an anti-vascular endothelial growth factor antibody, was associated with significant prolongation of the time to progression of disease when given at high dose compared to low-dose or placebo therapy though frequency of remissions or survival were not improved. AUTHORS' CONCLUSIONS: interferon-alfa provides a modest survival benefit compared to other commonly used treatments and should be considered for the control arm of future studies of systemic agents. In fit patients with metastases at diagnosis and minimal symptoms, nephrectomy followed by interferon-alfa gives the best survival strategy for fully validated therapies. The need for more effective specific therapy for this condition is apparent.

26 [95]. Correale, P., S. Marsili, et al. (2005). "Immunotherapy of renal cell carcinoma with granulocyte macrophage colony stimulating factor and very low dose interleukin-2." Oncol Rep **13**(4): 751-756.

We have performed a translational phase II trial testing an original immunotherapy schedule based on the monthly subcutaneous (s.c.) administration of hrGM-CSF (days 1 through 5) and very low dose hrIL-2 (days 6 through 15) in 19 patients with metastatic renal cell carcinoma. Bone pain, first dose reaction to GM-CSF, asthenia and fever were the most common side effects. A partial response, and a disease stabilization were respectively observed in 4 and 11 cases, with a rate of objective response and a disease control rate respectively of 21% and 79%. We recorded a time to progression of 9 months and a 2- and 3-year survival respectively of 42% (8/19 patients) and 26% (5/19 patients). Our results suggest that this GM-CSF/hrIL-2 combination is active and well tolerated in patients with renal cell carcinoma and deserves to be investigated in larger comparative trials.

27 [83]. Culine, S., F. Iborra, et al. (2006). "Subcutaneous interleukin-2 and interferon-alpha in metastatic renal cell carcinoma: results of a French regional experience in Languedoc." Am J Clin Oncol **29**(2): 148-152.

OBJECTIVES: To assess the efficacy and toxicity of an immunotherapy regimen combining subcutaneous (SC) interleukin-2 (IL-2) and interferon-alpha (IFN) in patients with metastatic renal cell carcinoma (MRCC). METHODS: The present study included 86 patients with MRCC. Data on treatment toxicity and efficacy (responses rates and overall survival) were collected on a hospital database. Treatment consisted of 6-week cycles repeated every 2 months for a maximum of 3 cycles. Each cycle included SC IL-2 20 x 10 MIU/m2 3 times/wk on weeks 1 and 4; 5 x 10 MIU/m2 3 times/wk on weeks 2, 3, 5, and 6, in combination with IFN 6 x 10 MIU/m2 once weekly on weeks 1 and 4; and 3 times/wk on weeks 2, 3, 5, and 6. RESULTS: Seventy (82%) and 71 (83%) patients received more than 80% of the planned doses of IL-2 and IFN during the first cycle, respectively. Ten patients had to stop therapy before the end of the first cycle because of excessive toxicity (7 patients) or rapidly progressive disease (3 patients). Only 17 (28%) proceeded to the second cycle. Main toxicities included fever and asthenia in 86 (100%) patients, nausea/emesis in 83 (96%) patients, skin disorders in 69 (80%) patients, hypotension in 56 (65%) patients, and diarrhea in 50 (58%) patients. Sixty-seven (78%) patients developed at least one episode of grade 3 toxicity. Objective responses were observed in 13 patients, including 4 complete and 9 partial responses (15%; 95% confidence interval, 9.5-20.5%). After a median follow-up of 45 months, the median time to progression was 4 months (range, 1-41) and the median survival was 14 months (range, 1-89). CONCLUSIONS: Only a small subset of patients with MRCC is likely to benefit from treatment with IL-2 and IFN. As toxicity is significant, the refinement of predictive variables for sensitivity to immunotherapy is mandatory.

28 [105]. Divgi, C. R., J. A. O'Donoghue, et al. (2004). "Phase I clinical trial with fractionated radioimmunotherapy using 131I-labeled chimeric G250 in metastatic renal cancer." J Nucl Med **45**(8): 1412-1421.

This trial was performed to determine the maximum tolerated whole-body radiation-absorbed dose of fractionated (131)I-cG250. METHODS: This was a phase 1 dose escalation trial. Dose escalation refers here to the escalation of average whole-body absorbed dose. Fifteen patients with measurable metastatic renal cancer were studied. For each treatment cycle, patients initially received a "scout" administration consisting of 5 mg of cG250 antibody labeled with 185 MBq (5 mCi) of (131)I. Whole-body and serum activity was measured for 1 wk, and a simple pharmacokinetic model was fitted to the measured data. The pharmacokinetic model was used to calculate the required activities, administered in a fractionated pattern with 2-3 d between fractions, projected to deliver the prescribed whole-body absorbed dose. The initial cohort of 3 patients was prescribed an average whole-body absorbed dose of 0.50 Gy. In subsequent cohorts this was increased in 0.25-Gy increments. The first fraction in each cycle was 1,110 MBq (30 mCi) of (131)I conjugated to 5 mg of antibody. Subsequent fractions consisted of variable activities depending on the patient-specific whole-body clearance rates and the times between fractions. Patients without evidence of disease progression were retreated after recovery from toxicity if there was no evidence of altered pharmacokinetics or serum human antichimeric antibody titers, for a total of no more than 3 treatments. RESULTS: For the initial treatment course, the pharmacokinetics of the scout dose accurately predicted the pharmacokinetics of fractionated (131)I-cG250 therapy. In 2 patients, altered clearance accurately predicted development of human antichimeric antibody. Targeting to known disease >or= 2 cm in diameter was noted in all patients. Dose-limiting toxicity was hematopoietic, and the maximum tolerated dose per cycle was 0.75 Gy. CONCLUSION: Measurements of whole-body and serum clearance of cG250 antibody can be used to accurately predict the clearance of subsequent administrations, thus enabling rational treatment planning. An additional practical benefit of real-time pharmacokinetic monitoring is that therapy can be altered dynamically to reduce toxic side effects. However, there was no evidence for fractionation-induced sparing of the hematopoietic system in this study.

29 [109]. Donskov, F., K. M. Bennedsgaard, et al. (2004). "Leukocyte orchestration in blood and tumour tissue following interleukin-2 based immunotherapy in metastatic renal cell carcinoma." Cancer Immunol Immunother **53**(8): 729-739.

With the objective of evaluating leukocyte orchestration in situ, serial blood samples and tumour tissue core needle biopsies were obtained at baseline and repeated after 1 month of therapy, among 49 consecutive single-institution patients with metastatic renal cell carcinoma (mRCC). Patients were treated with outpatient low-dose subcutaneous interleukin 2 (IL-2) and interferon alpha (IFN-alpha) alone (n = 23) or in combination with histamine dihydrochloride (n = 26). Objective responses were achieved in ten of 49 patients (20%) with an overall median survival of 14 months and an estimated 1- to 4-year survival rate of 57, 35, 24 and 22%, respectively. Toxicity was mild to moderate with no treatment-related deaths. High numbers of blood monocytes and neutrophils were significantly correlated to short survival. By contrast, high numbers of intratumoural CD3+, CD4+, CD8+ and CD57+ lymphocytes were positively correlated to objective response and/or long-term survival. Intratumoural lymphocytes showed low zeta expression, whereas blood lymphocytes showed almost normal levels of zeta expression. Neutrophils, the most frequent peripheral blood leukocyte subset, were scarce within the tumour tissue. Intratumoural eosinophils were not observed. In progressing patients, both the absolute number and the relative composition of leukocyte subsets in blood and tumour tissue remained unaffected by cytokine therapy. However, in responding patients, cytokine therapy was followed by an absolute and relative increase in T cells in blood as well as tumour tissue, an absolute and relative reduction in neutrophils in peripheral blood and a relative reduction of intratumoural macrophages. Histamine did not influence levels of intratumoural or blood leukocyte numbers, zeta-chain expression or cytotoxicity. In conclusion, the present regimen of outpatient low-dose subcutaneous IL-2 and IFN-alpha in mRCC should attract interest based on response, survival and toxicity. In responding patients, cytokine therapy was followed by substantial changes in the blood and tumour tissue leukocyte composition, correlated to response and survival. No discernable differences in immunologic parameters studied could be detected between histamine- and nonhistamine-treated patients.

30 [24]. Draube, A., N. Klein-Gonzalez, et al. (2011). "Dendritic cell based tumor vaccination in prostate and renal cell cancer: a systematic review and meta-analysis." PLoS One **6**(4): e18801.

BACKGROUND: More than 200 clinical trials have been performed using dendritic cells (DC) as cellular adjuvants in cancer. Yet the key question whether there is a link between immune and clinical response remains unanswered. Prostate and renal cell cancer (RCC) have been extensively studied for DC-based immunotherapeutic interventions and were therefore chosen to address the above question by means of a systematic review and meta-analysis. METHODOLOGY/PRINCIPAL FINDINGS: Data was obtained after a systematic literature search from clinical trials that enrolled at least 6 patients. Individual patient data meta-analysis was performed by means of conditional logistic regression grouped by study. Twenty nine trials involving a total of 906 patients were identified in prostate cancer (17) and RCC (12). Objective response rates were 7.7% in prostate cancer and 12.7% in RCC. The combined percentages of objective responses and stable diseases (SD) amounted to a clinical benefit rate (CBR) of 54% in prostate cancer and 48% in RCC. Meta-analysis of individual patient data (n = 403) revealed the cellular immune response to have a significant influence on CBR, both in prostate cancer (OR 10.6, 95% CI 2.5-44.1) and in RCC (OR 8.4, 95% CI 1.3-53.0). Furthermore, DC dose was found to have a significant influence on CBR in both entities. Finally, for the larger cohort of prostate cancer patients, an influence of DC maturity and DC subtype (density enriched versus monocyte derived DC) as well as access to draining lymph nodes on clinical outcome could be demonstrated. CONCLUSIONS/SIGNIFICANCE: As a 'proof of principle' a statistically significant effect of DC-mediated cellular immune response and of DC dose on CBR could be demonstrated. Further findings concerning vaccine composition, quality control, and the effect of DC maturation status are relevant for the immunological development of DC-based vaccines.

31 [66]. Escudier, B., T. K. Choueiri, et al. (2007). "Prognostic factors of metastatic renal cell carcinoma after failure of immunotherapy: new paradigm from a large phase III trial with shark cartilage extract AE 941." J Urol **178**(5): 1901-1905.

PURPOSE: We analyzed prognostic factors, described survival and generated a prognostic model in patients with metastatic renal cell carcinoma in whom immunotherapy failed and who were potentially eligible for novel agents. MATERIALS AND METHODS: An analysis of the relationship between clinical features and survival was performed in 300 patients with advanced renal cell carcinoma in whom immunotherapy had failed and who were subsequently treated as part of a single, phase III clinical trial with the anti-angiogenic agent Neovastat (shark cartilage extract AE 941). Clinical features were first examined univariately and a stepwise modeling approach based on Cox proportional hazard regression was then performed to generate a multivariate model. RESULTS: Median and progression-free survival (prognostic factors) for the whole cohort was 12.6 and 2 months, respectively. Prognostic features associated with shorter survival on multivariate analysis were the number of metastatic sites (greater than 1), time from nephrectomy to metastatic disease (less than 2 years), high alkaline phosphatase, abnormal corrected serum Ca and high lactate dehydrogenase (greater than 1.5 x the upper limit of normal). Four prognostic subgroups were identified by counting the number of adverse prognostic factors. Median survival in patients with zero adverse prognostic factors was 15.6 months compared to 11.7 months in patients with 1, 8.5 months in patients with 2 and 3.5 months in patients with 3 or more. CONCLUSIONS: We identified 4 risk groups to predict survival in previously treated patients with renal cell carcinoma. This model was based on data from what is to our knowledge the largest experience in this population. It should be used in clinical trial design, risk stratification and patient counseling.

32 [29]. Finkelstein, S. E., T. Carey, et al. (2010). "Changes in dendritic cell phenotype after a new high-dose weekly schedule of interleukin-2 therapy for kidney cancer and melanoma." J Immunother **33**(8): 817-827.

High-dose intravenous interleukin-2 (IL-2) therapy (14 doses/course, 2 courses/cycle) for metastatic melanoma or kidney cancer induces infrequent, although major responses. In this trial, we evaluated a new schedule (dose of 600,000 IU/kg, 8 h between doses, 5 doses/course, 4 courses at weekly intervals/cycle) of high-dose IL-2, in which we inserted more planned breaks while maintaining high cumulative dose delivery, and investigated the relationship between dendritic cells (DC) and response to treatment. Target dose delivery was attained: median IL-2 cumulative dose per patient was 11.4 and 10.8 million units/kg (cycles 1 and 2, respectively). Major responses were observed in patients with kidney cancer (n=20; 3 complete and 2 partial responses) and melanoma (n=16; 1 partial response). Adverse events appeared comparable with those typically associated with high-dose IL-2. From this data set, we introduce the hypothesis-generating observation that patients who had more favorable outcomes had high pretreatment DC-to-myeloid-derived suppressor cell (MDSC) ratios, similar to the ratio observed in healthy individuals. However, even in patients with the most favorable outcome, after treatment, there were IL-2-induced changes in the DC-to-MDSC ratio, specifically increases in MDSCs. This modified IL-2 schedule is a feasible option, with a more uniform dose delivery over the treatment cycle, a similar toxicity profile, and observed complete, durable response in patients with renal cancer. Pretreatment assessment of DC phenotypic or maturational status may be a starting point to predicting response to high-dose IL-2 cytokine immunotherapy in patients with melanoma and kidney cancer.

33 [7]. Florcken, A., J. Kopp, et al. (2013). "Allogeneic partially HLA-matched dendritic cells pulsed with autologous tumor cell lysate as a vaccine in metastatic renal cell cancer: a clinical phase I/II study." Hum Vaccin Immunother **9**(6): 1217-1227.

Multi-kinase inhibitors have been established for the treatment of advanced renal cell cancer, but long-term results are still disappointing and immunotherapeutic approaches remain an interesting experimental option particularly in patients with a low tumor burden. DC are crucial for antigen-specific MHC-restricted T cell immunity. Furthermore, allogeneic HLA-molecules pose a strong immunogenic signal and may help to induce tumor-specific T cell responses. In this phase I/II trial, 7 patients with histologically confirmed progressive metastatic RCC were immunized repetitively with 1 x 10 (7) allogeneic partially HLA-matched DC pulsed with autologous tumor lysate following a schedule of 8 vaccinations over 20 weeks. Patients also received 3 Mio IE IL-2 s.c. once daily starting in week 4. Primary endpoints of the study were feasibility and safety. Secondary endpoints were immunological and clinical responses. Vaccination was feasible and safe with no severe toxicity being observed. No objective response could be documented. However, while all patients had documented progress at study entry, 29% of the patients showed SD throughout the study with a mean TTP of 24.6 weeks (range 5 to 96 weeks). In 3/7 patients, TH1-polarized immune responses against RCC-associated antigens were observed. In one patient showing a minimal clinical response and a TTP of 96 weeks, clonally proliferated T cells against yet undefined antigens were induced by the vaccine. Vaccination with tumor antigen loaded DC remains an interesting experimental approach, but should rather be applied in the situation of minimal residual disease after systemic therapy. Additional depletion of regulatory cells might be a promising strategy.

34 [74]. Gez, E., R. Rubinov, et al. (2007). "Immuno-chemotherapy in metastatic renal cell carcinoma: long-term results from the rambam and linn medical centers, Haifa, Israel." J Chemother **19**(1): 79-84.

Nephrectomy, immuno-chemotherapy and resection of residual disease have been the treatment of choice for patients with metastatic renal cell carcinoma during the past decades. The aim of this study was to report the long-term results of this treatment approach. Sixty-two patients with metastatic renal cell carcinoma participated in a Phase II study. At diagnosis, 32 patients had localized disease, 30 had metastatic disease and 53 underwent nephrectomy. Metastatic sites were lungs, lymph nodes, bones and liver. Immuno-chemotherapy consisted of: interleukin-2, interferon alpha, 5-fluorouracil and vinblastine. All patients were evaluated for toxicity and response to treatment. CR was achieved in 4 patients and PR in 14. Seven patients, with maximum response to immuno-chemotherapy underwent resection of residual tumor and reached CR. Therefore, CR was achieved in 11 patients (18%) with a median survival of +67 months. Flu-like symptoms were the common side effects. Performance status and histology type significantly affected survival. Nephrectomy, immuno-chemotherapy and resection of residual disease are recommended for patients with metastatic renal cell carcinoma.

35 [21]. Gilles, R., L. F. de Geus-Oei, et al. (2013). "Immunotherapy response evaluation with (18)F-FDG-PET in patients with advanced stage renal cell carcinoma." World J Urol **31**(4): 841-846.

BACKGROUND: CT imaging is widely used for response evaluation of immunotherapy in patients with advanced stage renal cell carcinoma (RCC). However, this kind of treatment may not immediately be cytoreductive, although the treatment is successful. This poses new demands on imaging modalities. Positron emission tomography (PET) using (18)F-fluorodeoxyglucose (FDG) proved to be useful in monitoring the effect of several antitumour treatments. We investigated the potential of FDG-PET for the evaluation of response to immunotherapy. METHODS: In seven patients with metastasized RCC, who were treated with either interferon-alpha (IFN-alpha) monotherapy or a combination of IFN-alpha, interleukin-2 and 5-fluorouracil, FDG-PET was performed prior and after 5 and 9 weeks of treatment. Quantitative changes of glucose metabolic rate (MRGlu) were compared with changes in tumour size on CT imaging using Response Evaluation Criteria in Solid Tumors (RECIST) and to survival and progression-free survival. RESULTS: No consistent changes in MRGlu were observed within different response groups. And no correlation with CT imaging, neither with survival or progression-free survival, was found. CONCLUSION: In contrast to the positive results reported on (chemo) therapy response evaluation with FDG-PET in different malignancies, this imaging modality appears not useful in response monitoring of immunotherapeutic modalities in RCC.

36 [36]. Gore, M. E., C. L. Griffin, et al. (2010). "Interferon alfa-2a versus combination therapy with interferon alfa-2a, interleukin-2, and fluorouracil in patients with untreated metastatic renal cell carcinoma (MRC RE04/EORTC GU 30012): an open-label randomised trial." Lancet **375**(9715): 641-648.

BACKGROUND: In metastatic renal cell carcinoma combinations of interferon alfa-2a, interleukin-2, and fluorouracil produce higher response rates and longer progression-free survival than do single agents. We aimed to compare overall survival in patients receiving combination treatment or interferon alfa-2a. METHODS: RE04/30012 was an open-label randomised trial undertaken in 50 centres across eight countries. 1006 treatment-naive patients diagnosed with advanced metastatic renal cell carcinoma were randomly allocated (1 to 1) by minimisation to receive interferon alfa-2a alone or combination therapy with interferon alfa-2a, interleukin-2, and fluorouracil. Treatment was not masked. The primary endpoint was overall survival. Treatment groups were compared with a non-stratified log-rank test. Analysis was by intention to treat. This study is registered, number ISRCTN 46518965. FINDINGS: 502 patients were randomly assigned to receive interferon alfa-2a and 504 to receive combined treatment. Median follow-up was 37.2 months (24.8-52.3). Median overall survival was 18.8 months (17.0-23.2) for patients receiving interferon alfa-2a versus 18.6 months (16.5-20.6) for those receiving combination therapy. Overall survival did not differ between the two groups (hazard ratio 1.05 [95% CI 0.90-1.21], p=0.55; absolute difference 0.3% (-5.1 to 5.6) at 1 year and 2.7% (-8.2 to 2.9) at 3 years). Serious adverse events were reported in 113 (23%) patients receiving interferon alfa-2a and 131 (26%) of those receiving combined treatment. INTERPRETATION: Although combination therapy does not improve overall or progression-free survival compared with interferon alfa-2a alone, immunotherapy might still have a role because it can produce remissions that are of clinically relevant length in some patients. Identification of patients who will benefit from immunotherapy is crucial. FUNDING: UK Medical Research Council.

37 [115]. Harlin, H., A. S. Artz, et al. (2004). "Clinical responses following nonmyeloablative allogeneic stem cell transplantation for renal cell carcinoma are associated with expansion of CD8+ IFN-gamma-producing T cells." Bone Marrow Transplant **33**(5): 491-497.

Nonmyeloablative allogeneic stem cell transplantation (NST) is thought to be an immunologic therapy in which donor T cells mediate a graft-versus-tumor effect. We recently reported the clinical outcome of a phase II trial of NST in metastatic renal cell carcinoma (RCC). However, the immune response correlates of clinical activity remain unknown. We now describe the analysis of T-cell subsets and T-cell cytokine-producing potential for those patients evaluable for immune monitoring. The incidence of graft-versus-host disease (GVHD) correlated with clinical outcome, with all responders exhibiting chronic GVHD. Following initial tapering of immunosuppression, an increase in the total numbers of CD8+ T cells but not CD4+ T cells was observed among responders compared to nonresponders. In addition, a greater ratio of CD8+ to CD4+ T cells producing IFN-gamma and IL-2 was seen in clinical responders at the time when clinical responses were first detected (day 180 after transplantation). Our results support the hypothesis that the antitumor effects of NST may be mediated by IFN-gamma-producing CD8+ T cells, and indicate that isolation of putative tumor antigen-specific T cells, ideally, should be pursued around day +180.

38 [27]. Harrop, R., W. Shingler, et al. (2010). "Cross-trial analysis of immunologic and clinical data resulting from phase I and II trials of MVA-5T4 (TroVax) in colorectal, renal, and prostate cancer patients." J Immunother **33**(9): 999-1005.

The attenuated vaccinia virus MVA has been engineered to deliver the tumor antigen 5T4 (MVA-5T4; TroVax), a surface glycoprotein expressed by most solid tumors. MVA-5T4 has been tested in 2 phase I/II and 7 phase II clinical trials in colorectal (4 trials), renal (4 trials), and prostate (1 trial) advanced cancer patients. Data have been collated from all 9 studies and used to investigate the magnitude and kinetics of 5T4-specific antibody responses after vaccination and to identify potential associations between the immune response and patient survival. Antibody responses specific for the 5T4 tumor antigen and the MVA viral vector were quantified in plasma samples taken from cancer patients before and after the treatment with MVA-5T4. Immunologic and survival data were analyzed using proportional hazards regression adjusting for age and gender. Both survival and immunologic response data were available for 189 patients with colorectal (n=73), renal (n=89), and prostate (n=27) cancer. Before the treatment with MVA-5T4, 5T4-specific antibody levels were significantly elevated in cancer patients compared with healthy donors. After MVA-5T4 administration, 5T4-specific antibody responses increased significantly and peaked after 3 to 4 vaccinations. Exploratory analyses showed significant associations between 5T4 antibody responses and overall survival across all 9 trials and in patients with colorectal cancer. The 5T4-specific antibodies were present at higher levels in cancer patients compared with healthy donors and increased significantly after treatment with MVA-5T4. Although the studies were uncontrolled, there were encouraging signs of activity which is associated with the magnitude of 5T4-specific antibody responses.

39 [89]. Holtl, L., R. Ramoner, et al. (2005). "Allogeneic dendritic cell vaccination against metastatic renal cell carcinoma with or without cyclophosphamide." Cancer Immunol Immunother **54**(7): 663-670.

In this phase I/II study, we evaluated the feasibility, safety and efficacy of allogeneic dendritic cells (DCs) with or without cyclophosphamide in the treatment of patients with metastatic renal cell carcinoma (RCC). Immunomagnetic beads were used to isolate CD14(+) monocytes from healthy donor leukapheresis products, and CD83(+) antigen-pulsed monocyte-derived DCs (moDCs) loaded with tumor lysate and keyhole limpet hemocyanin (KLH) were generated. Twelve patients were treated with allogeneic moDCs alone, while ten patients also received cyclophosphamide on days 4 and 3 prior to vaccination. Of the 22 patients enrolled, 20 received full treatment consisting of at least three vaccinations at monthly intervals. Two mixed responses with substantial tumor regression were observed. In 3 patients, disease stabilization occurred, in 13 patients disease progressed and 4 patients were lost to follow-up. Overall, immune responses against KLH and tumor lysate were weak or absent; however, the strongest increases in antigen-independent and KLH-specific responses were observed in the 2 patients with mixed responses. In addition, 1 of them showed a substantial increase in oncofetal antigen (OFA)-specific IFN-gamma production. Importantly, the 2 mixed responders and 1 patient with stable disease belonged to the cyclophosphamide group. Median overall survival in the cyclophosphamide group was 23.2 and 20.3 months in the group that received allogeneic moDCs alone. Allogeneic immunotherapy with moDCs is feasible and well tolerated. However, the immunogenicity of allogeneic moDCs is clearly less pronounced than that of autologous moDC immunotherapy. Cyclophosphamide may have the capacity to augment DC-induced antitumor immunity.

40 [37]. Ismail-Zade, R. S., E. A. Zhavrid, et al. (2010). "[Use of LAK-cells and systemic chemotherapy with hyperthermia in the management of chemo-resistant tumors]." Vopr Onkol **56**(6): 681-686.

Tentative results of LAK-cell and whole-body hyperthermia (WBH) were evaluated in 19 children with advanced chemorefractory tumors. LAK-cells were obtained by extracorporeal incubation of peripheral blood lymphocytes: a germ-cell rhabdomyosarcoma was detected in 4, Askin's tumor--2--2, renal cell carcinoma--2 and miscellaneous--7. Autologous LAK-cells were infused twice: on completion of WBH as body temperature fell to as low as (+) 40 deg. C and on day after WBH. The latter was well tolerated. Complete or partial response to thermochemobiotherapy was reported in 8 patients. Overall 5-year survival was 43% (median follow-up--12.6 months).

41 [113]. Jocham, D., A. Richter, et al. (2004). "Adjuvant autologous renal tumour cell vaccine and risk of tumour progression in patients with renal-cell carcinoma after radical nephrectomy: phase III, randomised controlled trial." Lancet **363**(9409): 594-599.

BACKGROUND: Organ-confined renal-cell carcinoma is associated with tumour progression in up to 50% of patients after radical nephrectomy. At present, no effective adjuvant treatment is established. We aimed to investigate the effect of an autologous renal tumour cell vaccine on risk of tumour progression in patients with stage pT2-3b pN0-3 M0 renal-cell carcinoma. METHODS: Between January, 1997, and September, 1998, 558 patients with a renal tumour scheduled for radical nephrectomy were enrolled at 55 institutions in Germany. Before surgery, all patients were centrally randomised to receive autologous renal tumour cell vaccine (six intradermal applications at 4-week intervals postoperatively; vaccine group) or no adjuvant treatment (control group). The primary endpoint of the trial was to reduce the risk of tumour progression, defined as progression or death. All patients were assessed after standardised diagnostic investigations at 6-month intervals for a minimum of 4.5 years. FINDINGS: By preoperative and postoperative inclusion criteria, 379 patients were assessable for the intention-to-treat analysis. At 5-year and 70-month follow-up, the hazard ratios for tumour progression were 1.58 (95% CI 1.05-2.37) and 1.59 (1.07-2.36), respectively, in favour of the vaccine group (p=0.0204, log-rank test). 5-year and 70-month progression-free survival rates were 77.4% and 72%, respectively, in the vaccine group and 67.8% and 59.3%, respectively, in the control group. The vaccine was well tolerated, with only 12 adverse events associated with the treatment. INTERPRETATION: Adjuvant treatment with autologous renal tumour cell vaccine in patients with renal-cell carcinoma after radical nephrectomy seems to be beneficial and can be considered in patients undergoing radical nephrectomy due to organ-confined renal-cell carcinoma of more than 2.5 cm in diameter.

42 [63]. Kim, J. H., Y. Lee, et al. (2007). "Phase I/II study of immunotherapy using autologous tumor lysate-pulsed dendritic cells in patients with metastatic renal cell carcinoma." Clin Immunol **125**(3): 257-267.

This phase I/II study was conducted to evaluate the feasibility, safety and efficacy of immunotherapy using tumor lysate (TL)-pulsed dendritic cells (DC) in patients with metastatic renal cell carcinoma (RCC). DC were generated by culturing peripheral blood mononuclear cells in the presence of GM-CSF and IL-4 and were pulsed with autologous TL and keyhole limpet hemocyanin (KLH). Maturation of DC was induced by a combined treatment of CD40 ligand, IFN and monocyte-conditioned medium. The patients were administered two cycles of TL-pulsed DCs vaccination, each of which comprised of four doses injected subcutaneously at biweekly intervals. Nine patients were included. The immunotherapy was well tolerated without severe side effects. One patient achieved an objective partial response (PR). Five patients showed stable disease (SD), and the remaining three had progressive disease (PD). With a median follow-up of 17.5 months, the median time to progression was 5.2 months and the median overall survival was 29 months. In the antigen-specific lymphocyte proliferation assay, eight patients showed a proliferative response, which tended to be stronger in patients with SD or PR than in patients with PD. The ELISPOT assay was performed in two patients and indicated that one patient with PR showed a much stronger response than another with PD. Our results suggest that TL-pulsed DC immunotherapy in combination with nephrectomy affect the natural course of RCC and are associated with clinical benefits for patients with metastatic diseases.

43 [25]. Kobayashi, H., Y. Tanaka, et al. (2011). "Phase I/II study of adoptive transfer of gammadelta T cells in combination with zoledronic acid and IL-2 to patients with advanced renal cell carcinoma." Cancer Immunol Immunother **60**(8): 1075-1084.

Human Vgamma2 Vdelta2-bearing T cells have recently received much attention in cancer immunotherapy. In this study, we conducted a phase I/II clinical trial of the adoptive transfer of gammadelta T cells to patients with advanced renal cell carcinoma. Eleven patients who had undergone nephrectomy and had lung metastasis were enrolled. Peripheral blood gammadelta T cells obtained from the patients were stimulated ex vivo with 2-methyl-3-butenyl-1-pyrophosphate (2M3B1PP), a synthetic pyrophosphomonoester antigen, and transferred in combination with zoledronic acid (Zol) and teceleukin (recombinant human interleukin-2). Expanded gammadelta T cells exhibited potent cytotoxic activity against tumor cells in vitro, and the proportion of peripheral blood gammadelta T cells among CD3(+) cells typically peaked three to 5 days after transfer. Tumor doubling time was prolonged in all 11 patients, and the best overall responses were 1 CR, 5 SD, and 5 PD, as defined based on Response Evaluation Criteria in Solid Tumors (RECIST). Although ten patients developed adverse reactions of grade >/=3, they were likely to have been the result of the concomitant infusion of Zol and IL-2, and most symptoms swiftly reverted to normal during the course of treatment. In conclusion, this clinical trial demonstrated that our regimen for the adoptive transfer of gammadelta T cells in combination with Zol and IL-2 was well tolerated and that objective clinical responses could be achieved in some patients with advanced renal cell carcinoma.

44 [77]. Kobayashi, H., Y. Tanaka, et al. (2007). "Safety profile and anti-tumor effects of adoptive immunotherapy using gamma-delta T cells against advanced renal cell carcinoma: a pilot study." Cancer Immunol Immunother **56**(4): 469-476.

PURPOSE: Although various types of immunotherapy have been used to improve the prognosis of patients with advanced renal cell carcinoma (RCC), adoptive immunotherapy using gamma-delta (gammadelta) T cells has not yet been tried. In this study, we designed a pilot study of adoptive immunotherapy using in vitro activated gammadelta T cells against advanced RCC to evaluate the safety profile and possible anti-tumor effects of this study. EXPERIMENTAL DESIGN: Patients with advanced RCC after radical nephrectomy were administered via intravenous infusion in vitro-activated autologous gammadelta T cells every week or every 2 weeks, 6-12 times, with 70 JRU of teceleukin. Adverse events, anti-tumor effects and immunomonitoring were assessed. The anti-tumor effects were evaluated according to tumor doubling time (DT) by computed tomography (CT) and immunomonitoring was performed by flow cytometric analysis. RESULTS: Seven advanced RCC patients were entered in this study. The most common adverse events were fever, general fatigue and elevation of hepatobiliary enzymes, but no severe adverse events were seen. Prolongation of tumor DT was seen in three out of five patients; these three patients showed an increase in the number of gammadelta T cells in peripheral blood and also a high response to the antigen in vitro. CONCLUSIONS: The results indicated that adoptive immunotherapy using in vitro-activated autologous gammadelta T cells was well tolerated and induced anti-tumor effects.

45 [59]. Kobayashi, M., K. Nakano, et al. (2008). "[A pilot clinical trial of gemcitabine and capecitabine chemotherapy for the treatment of advanced renal cell carcinoma failing immunotherapy]." Gan To Kagaku Ryoho **35**(2): 277-279.

We have treated 8 patients with metastatic renal cell carcinoma for whom prior immunotherapy proved uneffective. The patients consisted of 6 males and 2 females, with a median age of 68 years. One patient had a primary site in place and 5 patients had multiple organs involvement. The chemotherapy consisted of gemcitabine 1,000 mg/m(2) div at day 1, 8 and capecitabine 1,657 mg/m(2)/day p.o. at day 1-14, followed by a week rest period. Then, the regimen was repeated every 3 weeks. No treatment response was observed. There were two SD, three SD to PD, and two PD after a median of 3 cycles of treatment. The median time to disease progression was 9.1 months, and the one-year progression- free survival rate was 83%. Adverse effects were observed in 3 cases (37.5%), however, treatment was well tolerated. Despite the limited anti-tumor efficacy, it would be meaningful that the treatment brought disease stabilization to the majority of this group of patients.

46 [34]. Kraemer, M., S. Hauser, et al. (2010). "Long-term survival of patients with metastatic renal cell carcinoma treated with pulsed dendritic cells." Anticancer Res **30**(6): 2081-2086.

AIM: This study aimed to determine the long-term survival of 30 dendritic cell (DC) vaccinated patients with metastatic renal cell cancer. PATIENTS AND METHODS: Patients were treated with a therapeutic vaccination of pulsed DCs in one of three clinical phase I/II trials during the years 1999 - 2003. RESULTS: Patients were followed up until 191 months (mean 65 days after DC therapy), when all patients had died. Total response to treatment was 40% with partial remission in 3%, mixed response in 7% and stable disease in 30%. The progressive disease rate was 60%. Long-term survival ranged from 3 to 191 months, with a mean survival of 59 months. Interestingly, patients who were treated previously with another form of immunotherapy showed a significantly improved probability of surviving. Mean long-term survival from the beginning of DC therapy was 21 months (1 to 75 months). CONCLUSION: Patients treated with DC vaccination seem to have a benefit in long-term survival.

47 [38]. Kutikov, A., R. G. Uzzo, et al. (2010). "Use of systemic therapy and factors affecting survival for patients undergoing cytoreductive nephrectomy." BJU Int **106**(2): 218-223.

OBJECTIVE: To present a multi-institutional experience evaluating the use of systemic therapy in patients undergoing cytoreductive nephrectomy (CN), as prospective randomized trials showed a survival benefit for CN in patients with metastatic renal cell carcinoma treated with immunotherapy, and these data have been extrapolated to support CN in the era of targeted therapy, but the likelihood that patients with metastatic kidney cancer who undergo CN will receive systemic treatment afterward remains poorly defined. PATIENTS AND METHODS: In all, 141 patients who underwent CN between 1990 and 2008 were identified from our Institutional Kidney Cancer Registries. Kaplan-Meier analyses and Cox regression models were used to assess the effect of clinicopathological and perioperative variables on patients' subsequent receipt of systemic therapy, and survival after CN. RESULTS: Overall, 98 of the 141 patients (69.5%) received postoperative systemic treatment, at a median (range) of 2.5 (0.1-61.5) months after CN. In this group, 52 (53%) patients received immunotherapy, 34 (35%) targeted agents, and 12 (12%) other regimens. By contrast, 43 patients (31%) did not receive systemic therapy, because of rapid disease progression (13, 30%), decision for surveillance by medical oncology (nine, 21%), patient refusal (10, 23%), perioperative death (eight, 19%), and unknown reasons in three (7%). The median (range) survival after CN was 16.7 (0-120) months. The risk of death after surgery correlated with the number of metastatic sites (P = 0.012) and symptoms (P = 0.001) at presentation, poor performance status (P = 0.001), high tumour grade (P = 0.006), and presence of sarcomatoid features (P < 0.024). CONCLUSION: Nearly a third of patients undergoing CN did not receive systemic treatment. While some were electively observed or declined therapy, others did not receive treatment due to rapidly progressive disease. Further investigation is warranted to identify those patients at highest risk of rapid postoperative disease progression who might benefit instead from an initial approach to treatment with systemic therapy.

48 [102]. Lamers, C. H., S. Sleijfer, et al. (2004). "Adoptive immuno-gene therapy of cancer with single chain antibody [scFv(Ig)] gene modified T lymphocytes." J Biol Regul Homeost Agents **18**(2): 134-140.

Adoptive transfer of antigen-specific T cells has recently shown therapeutic successes in the treatment of viral infections and tumors. T cells specific for the antigen of interest can be generated in vitro, and adoptively transferred back to provide patients with large numbers of immune-competent T cells. Adoptive T cell therapy, however, is a patient-tailored treatment that unfortunately is not universally applicable to treat viral infections and tumors. We and others have demonstrated that the transfer of genes encoding antigen-specific receptors into T cells (i.e., genetic retargeting) represents an attractive alternative to induce antigen-specific immunity. Currently, we evaluate this concept in a clinical protocol to treat patients with metastatic renal cell cancer (RCC) using autologous RCC-specific gene-modified T lymphocytes.

49 [23]. Lang, J. M., M. R. Kaikobad, et al. (2011). "Pilot trial of interleukin-2 and zoledronic acid to augment gammadelta T cells as treatment for patients with refractory renal cell carcinoma." Cancer Immunol Immunother **60**(10): 1447-1460.

Prior to the advent of VEGF-targeted therapies, renal cell carcinoma (RCC) was among the few solid tumors shown to respond to cytokine-based therapies such as interleukin-2 (IL-2) and interferon alpha. Previous work has shown that aminobisphosphonates, including zoledronic acid (ZA), are capable of activating human Vgamma9 Vdelta2 T cells in vitro, and these cells can be further expanded with IL-2. Moreover, these Vgamma9 Vdelta2 T cells have cytolytic activity in vitro to multiple human tumor cell lines. In the current report, we have conducted a pilot trial in patients with metastatic RCC, evaluating different doses of ZA in combination with low-dose IL-2 to determine whether combining these agents can promote in vivo proliferation of Vgamma9 Vdelta2 T cells and elicit an antitumor response. In 12 patients evaluated, no objective clinical responses were observed by RECIST criteria; however, two patients experienced prolonged stable disease. A modest increase in Vgamma9 Vdelta2 T-cell frequency could be detected by Day 8 of therapy in four of the nine patients who received at least one cycle of therapy, but not to the magnitude anticipated from preclinical models. Repeated administration of IL-2 and ZA resulted in both a diminished in vivo percentage of Vgamma9 Vdelta2 T cells as well as impaired expansion in vitro after the first cycle of therapy. These results suggest that repeated administration of IL-2 and ZA, at the doses and schedules used in this trial, may actually inhibit the proliferative capacity of Vgamma9 Vdelta2 T cell in patients with metastatic RCC.

50 [43]. Lemoine, F. M., M. Cherai, et al. (2009). "Massive expansion of regulatory T-cells following interleukin 2 treatment during a phase I-II dendritic cell-based immunotherapy of metastatic renal cancer." Int J Oncol **35**(3): 569-581.

Cytotoxic chemotherapy is ineffective in metastatic renal cancer. However, systemic administration of interleukin 2 (IL-2) or infusion of dendritic cells (DCs) loaded with tumor extracts can lead to some response rates with concomitant survival improvements. We report the results of a phase I-II pilot study combining DCs and IL-2 where six patients were included. DCs were derived from bone marrow CD34+ cells and loaded with autologous tumor extracts. CD34-DC vaccines were infused subcutaneously at day 45, 52, 59, 90 and 120 following surgery in combination with IL-2, that was subsequently administrated after the 3rd and 4th DC vaccinations. Preparation of tumor extracts and CD34-DCs were satisfactory in all patients but one. Due to rapid tumor progression, one patient was excluded before vaccination. In the 4 remaining patients, two received 3 vaccinations, while the 2 others received 5 vaccinations and the full IL-2 treatment. No adverse effect due to the vaccinations was observed. A specific immune response against autologous tumor cells was observed in the 2 patients who completed the treatment. Interestingly, these 2 patients had a more prolonged survival than the patients receiving 3 vaccinations. Importantly, a transient and massive increase of circulating natural regulatory T-cells (nTregs) was evidenced in 3 patients following IL-2 administration. Overall, the use of CD34-DC vaccines is feasible, safe and non-toxic. A specific anti-tumor immune response can be detected. However, our data highlights that IL-2 is a potent inducer of nTregs in vivo and as such may have a negative impact on cancer immunotherapy.

51 [14]. Liu, L., W. Zhang, et al. (2012). "Randomized study of autologous cytokine-induced killer cell immunotherapy in metastatic renal carcinoma." Clin Cancer Res **18**(6): 1751-1759.

PURPOSE: The therapeutic benefit of the cytokine-induced killer (CIK) cells was unknown in the renal cell carcinoma (RCC). This prospectively randomized study was conducted to evaluate the effects of autologous CIK cell immunotherapy in patients with metastatic clear cell RCCs. EXPERIMENTAL DESIGN: From June 2005 to June 2008, 148 patients with metastatic clear cell RCC were randomized to autologous CIK cell immunotherapy (arm 1, n = 74), or interleukin-2 treatment combination with IFN-alpha-2a (arm 2, n = 74). The primary endpoint was overall survival (OS) and secondary endpoint was progression-free survival (PFS) evaluated by Kaplan-Meier analyses and treatment HRs with the Cox proportional hazards model. RESULTS: The 3-year PFS and OS in arm 1 were 18% and 61%, as compared with 12% and 23% in arm 2 (P = 0.031 and <0.001, respectively). The median PFS and OS in arm 1 were significantly longer than those in arm 2 (PFS, 12 vs. 8 months, P = 0.024; OS, 46 vs. 19 months, P < 0.001). Multivariate analyses indicated that the cycle count of CIK cell immunotherapy as a continuous variable was significantly associated with prolonged PFS [HR = 0.88; 95% confidence interval (CI), 0.84-0.93; P < 0.001] and OS (HR = 0.58; 95% CI, 0.48-0.69; P < 0.001) in arm 1. CONCLUSION: The data suggested that CIK cell immunotherapy could improve the prognosis of metastatic clear cell RCC, and increased cycle count of CIK cell treatment could further enhance the beneficial effects.

52 [110]. Lummen, G., M. Schenck, et al. (2004). "[Inhaled immunotherapy for pulmonary metastases of renal cell cancer]." Urologe A **43**(4): 457-461.

Studies on immunotherapy with inhaled interleukin-2 (IL-2) for the treatment of pulmonary metastases in renal cell carcinoma patients have indicated objective response rates of 11%. The aim of the present study was to evaluate efficacy, toxicity, and quality of life during inhaled immunotherapy with IL-2. Patients with pulmonary metastases of renal cell carcinoma were treated with interferon-alpha (IFN-alpha) 3 x 10(6) IU/m(2) s.c. on days 1, 3, and 5 and inhaled twice a day 9 x 10(6) IU IL-2 on days 1-5. Treatment continued for 4 weeks and after a 2-week rest a second cycle was given. Patients who responded received two additional cycles. Quality of life was assessed according a self-administered quality of life questionnaire (QLQ-C30) before, during, and after therapy. Of 23 treated patients, 21 could be evaluated concerning response rate and toxicity [16 men, 5 women; median age: 60 years (38-72 years)]. Sixteen patients had pulmonary metastases only and five patients additionally had bone or liver metastasis or local recurrence. One patient (5%) developed a partial remission for 4 months and ten patients (47.5%) showed a stable disease for a median time of 6 months (2-24 months). The median follow-up was 9 months (3-26 months). Ten patients (47.5%) developed progressive disease. Maximal toxicity was mild and grade III-IV toxicity (WHO) was not observed. The patients' quality of life did not change significantly at any time during therapy. Inhaled immunotherapy is a treatment option with little toxicity, but achieved only a few objective responses. Whether or not it influences overall survival could not be answered in this study.

53 [9]. Maroto, J. P., X. G. del Muro, et al. (2013). "Phase II trial of sequential subcutaneous interleukin-2 plus interferon alpha followed by sorafenib in renal cell carcinoma (RCC)." Clin Transl Oncol **15**(9): 698-704.

PURPOSE: Immunotherapy (IL-2 and INF-alpha) was the treatment of choice for advanced renal cell carcinoma (RCC) until antiangiogenic therapy with tyrosin kinase inhibitors was developed in the early 2000s. This clinical trial explored the efficacy and toxicity of sequential treatment of IL-2 plus INF-alpha followed by sorafenib. METHODS: Eligibility criteria included measurable, non-resectable, histologically confirmed predominantly clear cell RCC, no prior systemic treatment, and ECOG PS 0-2. The treatment regimen was a 6-week cycle of subcutaneous IL-2 at 9 x 10(6) IU on days 1-6 of weeks 1, 2, 4 and 5 plus s.c. INF-alpha at 6 x 10(6) IU on days 1, 3 and 5 of weeks 1-6. Responders received 6 additional weeks of this regimen. All patients received oral sorafenib (400 mg bid) after immunotherapy until disease progression. The primary endpoint was progression-free survival. RESULTS: Forty-one patients were enrolled, median age 57 years. ECOG was 0/1 in 17/20 patients, 35 patients had prior nephrectomy and 18 patients pure clear cell cancer. Median PFS was 7.4 months (95 % CI 6.5-13.1) and OS was 16.6 months (95 % CI not reached). In 36 patients evaluable for response, ORR was 44.4 % and control rate was 94.4 %. Most adverse events (AEs) were Grade 1 or 2 toxicities (84.7 %). During immunotherapy the most common AEs were pyrexia (82.9 %), asthenia (56.1 %) and anorexia (46.3 %), whereas during sorafenib were diarrhoea (48.8 %) and hand-foot syndrome (46.3 %). CONCLUSIONS: A sequential regimen of IL-2 and INF-alpha followed by sorafenib showed effectiveness and manageable toxicity in patients with advanced RCC.

54 [85]. Maroto, J. P., R. Martino, et al. (2006). "[Allogeneic transplantation of peripheral blood hematopoietic stem-cells with low intensity conditioning for the treatment of a metastatic clear cell carcinoma. Preliminary results after two years of follow-up]." Arch Esp Urol **59**(1): 44-48.

OBJECTIVES: To evaluate the toxicity and outcomes of the allogeneic transplantation of peripheral blood hematopoietic stem-cells with low intensity conditioning (mini-alo Bone Marrow transplantation-BMT) in metastatic renal cell carcinoma refractory to at least one line of systemic treatment. METHODS: From 30 patients submitted to the Sant Pau's Hospital for immunotherapy between 1/2001 and 1/2003, six patients finally underwent mini-alo BMT within a clinical trial. Conditioning: Fludarabine: 30 minute i.v. infusion of 13 mg/m2 on days -9, -8, -7, -6 and -5. Busulfan: 1 mg/kg (real weight) q 6 hours, PO on days -6, -5, and -4 (4 doses per day on days -6 and -5, and 2 doses only on day -4, total number of doses 10). RESULTS: Two patients achieved partial response, one patient stabilized disease, and two patients had progression on days +30 and +60. Therefore, 2/3 patients surviving more than six months achieved partial response. One patient died on day +30 after acute hepatic graft-versus-host disease. Complete chimerism was demonstrated in all patients. CONCLUSIONS: The mimni-alo BMT is feasible in patients with metastatic renal cell carcinoma and provides objective responses, although its efficacy should be confirmed by phase III clinical trials.

55 [78]. Masucci, G. V., E. Mansson-Brahme, et al. (2006). "Alternating chemo-immunotherapy with temozolomide and low-dose interleukin-2 in patients with metastatic melanoma." Melanoma Res **16**(4): 357-363.

Temozolomide is a rapidly absorbed chemotherapeutic agent, achieving significant central nervous system penetration. Previous clinical trials suggested that temozolomide in sequence with low-dose recombinant human interleukin-2 might be an efficacious and relatively non-toxic chemo-immunotherapeutic treatment, which may synergistically eliminate tumours. The primary objective was to determine the safety and tolerance of temozolomide administered orally 200 mg/m days 1-5, in sequential combination with subcutaneous injections of 4.5x10 IU recombinant human interleukin-2 on days 8-11, 15-18 and 22-25 in patients with measurable, progressive metastatic malignant melanoma without radiological signs of central nervous system metastases. The secondary objectives were to determine tumour response and time to progression. Twenty-seven patients were included, of which four were non-evaluable for response. Twenty-three patients tolerated the regimen with side effects below grade 3 according to the World Health Organization (WHO) scale. Three patients suspended the treatment because of WHO grade 3 side effects already during the first 3 days of the first course of temozolomide. Seven patients showed no tumour progression during the first four treatment cycles. Two patients had complete responses, three partial responses and two stable disease at the end of the four cycles defined by the protocol and they continued the treatment until signs of relapse or a maximum of 21 courses. Five of these patients are still alive. Thrombocytopenia was significantly more pronounced in patients with objective response and stable disease than in non-responders to therapy. The median time to progression for all patients was 3.1 months and for responding and stable disease patients was 15 months. Five of 23 treated patients (22%) developed brain metastases during follow-up. Temozolomide in combination with recombinant human interleukin-2 is a well-tolerated regimen for outpatient treatment and the bio-chemotherapy combination induced durable clinical responses. Thrombocytopenia might be a positive predictive factor for response to therapy.

56 [72]. Matsumoto, A., K. Haraguchi, et al. (2007). "Immunotherapy against metastatic renal cell carcinoma with mature dendritic cells." Int J Urol **14**(4): 277-283.

OBJECTIVE: We performed a clinical trial of immunotherapy using autologous mature dendritic cells (DC) pulsed with autologous tumor lysate, for patients with metastatic renal cell carcinoma (RCC). METHODS: Patients with refractory metastatic RCC were enrolled in the study. All of them received interferon (IFN)-alpha treatment after nephrectomy and were followed over 3 months prior to this study. Autologous monocyte-derived immature DC were pulsed with lysate from autologous primary tumor as the antigen and keyhole limpet hemocyanin (KLH) as immunomodulator, and cultured in the presence of tumor necrosis factor (TNF)-alpha, interleukin (IL)-1beta, and prostaglandin (PG)E2 to generate mature DC. Mature DC were injected intradermally near bilateral inguinal lymph nodes of the patients. A delayed-type hypersensitivity (DTH) test and enzyme-linked immunospot (ELISPOT) assay were performed to evaluate the immunological response. After 4 months from first injection, the clinical effect was evaluated by diagnostic imaging. RESULTS: The treatments were well tolerated without significant toxicity by the patients who were an average of 65.7 years old and had multiple metastases in the lung and other organs. One of the two patients developed a positive DTH reaction to tumor lysate and the other patient only to KLH. The patient with a positive DTH reaction to tumor lysate had stable disease in the clinical evaluation. CONCLUSIONS: We confirmed the safety of DC therapy in this clinical trial. The DTH test revealed that the DC therapy induced immunological response to RCC. On the other hand, it was necessary to reconsider the patient selection criteria.

57 [3]. Melichar, B., S. Bracarda, et al. (2013). "A multinational phase II trial of bevacizumab with low-dose interferon-alpha2a as first-line treatment of metastatic renal cell carcinoma: BEVLiN." Ann Oncol **24**(9): 2396-2402.

BACKGROUND: Avastin and Roferon in Renal Cell Carcinoma (AVOREN) demonstrated efficacy for bevacizumab plus interferon-alpha2a (IFN; 9 MIU tiw) in first-line metastatic renal cell carcinoma (mRCC). We evaluated bevacizumab with low-dose IFN in mRCC to determine whether clinical benefit could be maintained with reduced toxicity. METHODS: BEVLiN was an open-label, single-arm, multinational, phase II trial. Nephrectomized patients with treatment-naive, clear cell mRCC and favourable/intermediate Memorial Sloan-Kettering Cancer Center scores received bevacizumab (10 mg/kg every 2 weeks) and IFN (3 MIU thrice weekly) until disease progression. Descriptive comparisons with AVOREN patients having favourable/intermediate MSKCC scores treated with bevacizumab plus IFN (9 MIU) were made. Primary end points were grade >/=3 IFN-associated adverse events (AEs) and progression-free survival (PFS). All grade >/=3 AEs and bevacizumab/IFN-related grade 1-2 AEs occurring from first administration until 28 days after last treatment were reported. RESULTS: A total of 146 patients were treated; the median follow-up was 29.4 months. Any-grade and grade >/=3 IFN-associated AEs occurred in 53.4% and 10.3% of patients, respectively. The median PFS and overall survival were 15.3 [95% confidence interval (CI): 11.7-18.0] and 30.7 months (95% CI: 25.7-not reached), respectively. The ORR was 28.8%. CONCLUSIONS: Compared with a historical control AVOREN subgroup, low-dose IFN with bevacizumab resulted in a reduction in incidence rates of IFN-related AEs, without compromising efficacy [NCT00796757].

58 [116]. Meller, B., C. Frohn, et al. (2004). "Monitoring of a new approach of immunotherapy with allogenic (111)In-labelled NK cells in patients with renal cell carcinoma." Eur J Nucl Med Mol Imaging **31**(3): 403-407.

The transfusion of allogenic, in vitro expanded natural killer cells (NKC) is a novel therapy option in oncology. To date, however, the biodistribution and kinetics of allogenic NKC have not been investigated. Therefore, in this study three patients with renal cell carcinoma received 3-7 x 10(8) NKC labelled with indium-111 oxine with a tenfold excess of unlabelled cells during NKC therapy. Whole-body scintigrams were obtained (0.5-144 h) in the anterior and posterior views. Scintigrams were analysed using a region of interest technique, and single-photon emission tomography (SPET) studies of the abdomen were performed. Results were compared to those obtained with polymerase chain reaction (PCR) of the peripheral blood (determination of foreign DNA, nested PCR, limit of detection 0.01%). Shortly after transfusion of NKC, more than 50% of the activity was accumulated in the lungs. We observed redistribution effects from lungs to liver, spleen and bone marrow. No significant loss of activity could be detected. In two of four large metastases, tracer accumulation could be proven by SPET. As confirmed by scintigrams and PCR, the fraction of circulating transfused cells was low at all times. Long-term activity retention might be caused either by survival of the allogenic cells, as confirmed by PCR (up to 3 days p.i.), or by phagocytosis of labelled cellular fragments. However, PCR data and uptake in metastases indicated long survival of a portion of allogenic NKC. Such long survival and low retention of the cells in the lung are requirements for an effective immunotherapeutic approach.

59 [68]. Messina, G., P. Lissoni, et al. (2007). "Efficacy of IL-2 immunotherapy in metastatic renal cell carcinoma in relation to the psychic profile as evaluated using the Rorschach test." Anticancer Res **27**(4C): 2985-2988.

BACKGROUND: Despite the well-documented importance of the psycho-emotional status in modulating the anticancer immunity, at present no study has been performed to analyse the influence of the psychological condition on the efficacy of IL-2 cancer immunotherapy. Previous clinical studies have already suggested that the evidence of anxiety may negatively affect the therapeutic efficacy of IL-2 immunotherapy of cancer. Moreover, previous psycho-oncological investigations showed that the suppression of sexual pleasure and sexual identity would represent one of the most frequent psychological profiles in cancer patients. On this basis, a study was planned in an attempt to evaluate relations existing between psychological status, analysed using the Rorschach test and efficacy of IL-2 immunotherapy in the treatment of metastatic renal cell cancer patients. PATIENTS AND METHODS: The study included 30 consecutive metastatic RCC patients. IL-2 was injected s.c. at a dose of 3 million IU twice/day 5 days/week for 4 consecutive weeks, corresponding to one complete immunotherapeutic cycle, followed by a second cycle after a 21-day rest period. RESULTS: A complete response (CR) was achieved in only 1/30 (3%) patients; a partial response (PR) was obtained in 6/30 (20%) patients. The tumor response rate (CR +PR) was 7/30 (23%) patients. The performance of a psychological analysis was accepted by 24/30 (80%) patients. A normal sexual identity was present in 7/24 (29%) patients. The tumor response rate achieved in patients with sexual identity was significantly higher compared to these who had no sexual identity or who refused the psychological investigation (p<0.05 and p<0.01, respectively). In the same way, the increase in mean lymphocyte number obtained in patients with sexual identity was significantly higher compared to that found in the other two groups of patients. CONCLUSION: This study demonstrated that the psychological status prior to treatment may be associated with the clinical response to IL-2 cancer immunotherapy.

60 [98]. Miller, J. S., Y. Soignier, et al. (2005). "Successful adoptive transfer and in vivo expansion of human haploidentical NK cells in patients with cancer." Blood **105**(8): 3051-3057.

We previously demonstrated that autologous natural killer (NK)-cell therapy after hematopoietic cell transplantation (HCT) is safe but does not provide an antitumor effect. We hypothesize that this is due to a lack of NK-cell inhibitory receptor mismatching with autologous tumor cells, which may be overcome by allogeneic NK-cell infusions. Here, we test haploidentical, related-donor NK-cell infusions in a nontransplantation setting to determine safety and in vivo NK-cell expansion. Two lower intensity outpatient immune suppressive regimens were tested: (1) low-dose cyclophosphamide and methylprednisolone and (2) fludarabine. A higher intensity inpatient regimen of high-dose cyclophosphamide and fludarabine (Hi-Cy/Flu) was tested in patients with poor-prognosis acute myeloid leukemia (AML). All patients received subcutaneous interleukin 2 (IL-2) after infusions. Patients who received lower intensity regimens showed transient persistence but no in vivo expansion of donor cells. In contrast, infusions after the more intense Hi-Cy/Flu resulted in a marked rise in endogenous IL-15, expansion of donor NK cells, and induction of complete hematologic remission in 5 of 19 poor-prognosis patients with AML. These findings suggest that haploidentical NK cells can persist and expand in vivo and may have a role in the treatment of selected malignancies used alone or as an adjunct to HCT.

61 [18]. Montagna, D., I. Turin, et al. (2012). "Feasibility and safety of adoptive immunotherapy with ex vivo-generated autologous, cytotoxic T lymphocytes in patients with solid tumor." Cytotherapy **14**(1): 80-90.

BACKGROUND AIMS: Adoptive T-cell therapy with tumor-specific T cells has emerged as a potentially useful approach for treating patients with advanced malignancies. We have demonstrated previously the feasibility of obtaining large numbers of autologous anti-tumor-specific cytotoxic T lymphocytes (CTL) generated by stimulation of patients' peripheral blood mononuclear cells with dendritic cells pulsed with apoptotic tumor cells. Methods. Six patients with progressing metastatic solid tumors (one renal cell carcinoma, two ovarian cancers, two extraosseous peripheral neuroectodermal tumors, one soft tissue sarcoma) not eligible for conventional therapies were treated with adoptive immunotherapy. Anti-tumor CTL, proven to be reactive in vitro against patient tumor cells, but not against normal cells, were infused following lymphodepleting chemotherapy administered to favor T-cell proliferation in vivo. RESULTS: Patients received a median of nine CTL infusions (range 2-19). The median number of CTL administered per infusion was 11 x 10(8) (range 1-55 x 10(8)). No patient experienced acute or late adverse events related to CTL infusion, even when large numbers of cells were given. Post-infusion laboratory investigations demonstrated an increase in the frequency of circulating anti-tumor T-cells and, in patients with a longer follow-up receiving two CTL infusions/year, a stabilization of these values. CONCLUSIONS: Our study demonstrates that autologous ex vivo-generated anti-tumor CTL can be administered safely in patients with advanced solid tumors and can improve the immunologic reactivity of recipients against tumor. These preliminary results provide a rationale for evaluating the clinical efficacy of this immunotherapeutic approach in phase I/II studies.

62 [49]. Montie, J. E., P. E. Clark, et al. (2009). "Bladder cancer." J Natl Compr Canc Netw **7**(1): 8-39.

63 [22]. Mulder, S. F., J. F. Jacobs, et al. (2011). "Cancer patients treated with sunitinib or sorafenib have sufficient antibody and cellular immune responses to warrant influenza vaccination." Clin Cancer Res **17**(13): 4541-4549.

PURPOSE: The tyrosine kinase inhibitors sorafenib and sunitinib have efficacy in several types of cancer. Recent studies indicate that these agents affect the immune system. The way it affects the immune response to influenza vaccination is unknown. The aim of this study was to elucidate the specific immune response to seasonal flu vaccination in cancer patients treated with sunitinib or sorafenib. PATIENTS AND METHODS: Sunitinib- or sorafenib-treated cancer patients were vaccinated against seasonal influenza with an inactivated vaccine. Healthy controls and patients with metastatic renal cell cancer (mRCC) without systemic treatment (nontreated mRCC controls) were included for comparison. Antibody responses were measured at baseline, day 8, and day 22 by a standard hemagglutination inhibition assay and cellular T-cell responses at baseline and day 8 by proliferation assay and secretion of cytokines. RESULTS: Forty subjects were enrolled: 16 patients treated with sunitinib, 6 patients with sorafenib, 7 nontreated mRCC controls, and 11 healthy controls. All patients treated with sunitinib and sorafenib developed seroprotection rates comparable with controls. Functional T-cell reactivity was observed in all groups, except for patients treated with sorafenib who showed a decreased proliferation rate and IFN-gamma/IL-2 production and increased IL-10 compared with healthy controls. CONCLUSION: We conclude that influenza vaccination should be recommended to cancer patients treated with sunitinib or sorafenib.

64 [46]. Olioso, P., R. Giancola, et al. (2009). "Immunotherapy with cytokine induced killer cells in solid and hematopoietic tumours: a pilot clinical trial." Hematol Oncol **27**(3): 130-139.

BACKGROUND AND OBJECTIVES: CIK cells are a novel population of efficient immune effector cells with high antitumour activity mainly due to the high proliferation of CD3(+)CD56(+) cells, so may play a role in the development of new forms of adoptive cellular immunotherapy. We started a pilot clinical trial with autologous CIK cells in patients with refractory lymphoma and metastatic solid tumours. This study was aimed at determining the feasibility of generating a sufficient number of CIK cells in heavily pretreated patients and at assessing treatment toxicity. DESIGN AND METHODS: CIK cells were generated from peripheral blood mononuclear cells (MNC) and incubated in the presence of IFN-gamma followed by OKT3 and IL-2. Treatment schedule consisted of three cycles of CIK cells infusions at an interval of 3 weeks. RESULTS: At present 12 patients were enrolled: 6 advanced lymphomas, 5 metastatic kidney carcinoma and 1 hepatocellular carcinoma (HCC). The median number of transferred cells per patient was 28 x 10(9) (range, 6-61). Protocol adherence was excellent and the toxicity profile was favourable. After CIK cells infusion, the absolute median count of lymphocytes, CD3(+), CD8(+) and CD3(+)CD56(+) cells significantly increased in patient's peripheral blood. Clinical outcome appeared promising: three patients had complete response (CR) and two patients had stabilization of disease with a median follow-up of 33 months (range, 9-44). INTERPRETATIONS AND CONCLUSIONS: These preliminary data showed that adoptive immunotherapy with CIK cells is a safe therapy with some suggestion of efficacy that significantly enhances immune functions increasing absolute numbers of effector cells without side effects. If confirmed in larger scale studies, these promising results may have a favourable impact on conventional treatment strategy of malignancies.

65 [106]. Pandha, H. S., R. J. John, et al. (2004). "Dendritic cell immunotherapy for urological cancers using cryopreserved allogeneic tumour lysate-pulsed cells: a phase I/II study." BJU Int **94**(3): 412-418.

OBJECTIVE: To assess the feasibility, toxicity and immunogenicity of dendritic cell (DC)-based immunotherapy in patients with advanced urological cancers. PATIENTS AND METHODS: Patients with hormone-refractory prostate cancer (11) and metastatic renal cell carcinoma (five) received 1-3 x 10(6) intradermal allogeneic tumour lystate-pulsed DCs fortnightly for six vaccinations then monthly until disease progression. Intradermal keyhole limpet haemocyanin was injected near the DCs as the adjuvant. DC vaccine was prepared from buffy coats, then lysate-pulsed, cryopreserved in aliquots, and tested for phenotypic expression and activity in an allogeneic mixed lymphocyte reaction before clinical use. RESULTS: There was no evidence of significant toxicity from vaccine or adjuvant. Delayed-type hypersensitivity skin testing and biopsy revealed a cellular infiltrate to intradermal re-challenge to tumour lysate and adjuvant in almost all patients. In addition, there was increased expression of T helper type 1 cytokines, interferon-gamma-expressing T cell by ELISPOT analysis, but also interleukin-10 in a few patients. Vaccination resulted in a reduction in the level of prostate-specific antigen (PSA) in one patient, a reduction in PSA velocity in a further man and an increased PSA doubling time in six. Two of five patients with renal cell carcinoma had stabilization of disease. CONCLUSION: The cryopreservation and repeated administration of DC vaccine was feasible and not toxic. There was evidence of induction of both humoral and cellular immunity to vaccine and adjuvant in most patients. The use of sequential aliquots of identical cryopreserved vaccine will ensure quality control and greatly facilitate future clinical studies in terms of consistency of vaccine administered and the provision of primed DCs for in vitro assessment of response.

66 [19]. Parkhurst, M. R., J. P. Riley, et al. (2011). "Adoptive transfer of autologous natural killer cells leads to high levels of circulating natural killer cells but does not mediate tumor regression." Clin Cancer Res **17**(19): 6287-6297.

PURPOSE: Adoptive transfer of tumor-infiltrating lymphocytes (TIL) can mediate regression of metastatic melanoma. However, many patients with cancer are ineligible for such treatment because their TIL do not expand sufficiently or because their tumors have lost expression of antigens and/or MHC molecules. Natural killer (NK) cells are large granular lymphocytes that lyse tumor cells in a non-MHC-restricted manner. Therefore, we initiated in a clinical trial to evaluate the efficacy of adoptively transferred autologous NK cells to treat patients with cancers who were ineligible for treatment with TIL. EXPERIMENTAL DESIGN: Patients with metastatic melanoma or renal cell carcinoma were treated with adoptively transferred in vitro activated autologous NK cells after the patients received a lymphodepleting but nonmyeloablative chemotherapy regimen. Clinical responses and persistence of the adoptively transferred cells were evaluated. RESULTS: Eight patients were treated with an average of 4.7 x 10(10) (+/- 2.1 x 10(10)) NK cells. The infused cells exhibited high levels of lytic activity in vitro. Although no clinical responses were observed, the adoptively transferred NK cells seemed to persist in the peripheral circulation of patients for at least one week posttransfer and, in some patients, for several months. However, the persistent NK cells in the circulation expressed significantly lower levels of the key activating receptor NKG2D and could not lyse tumor cell targets in vitro unless reactivated with IL-2. CONCLUSIONS: The persistent NK cells could mediate antibody-dependent cell-mediated cytotoxicity without cytokine reactivation in vitro, which suggests that coupling adoptive NK cell transfer with monoclonal antibody administration deserves evaluation.

67 [41]. Passalacqua, R., C. Buzio, et al. (2010). "Phase III, randomised, multicentre trial of maintenance immunotherapy with low-dose interleukin-2 and interferon-alpha for metastatic renal cell cancer." Cancer Immunol Immunother **59**(4): 553-561.

This is the first phase III randomised trial to evaluate maintenance immunotherapy in metastatic renal cell cancer (mRCC). Patients were randomised to receive treatment with a 4-week cycle of subcutaneous low doses IL-2 + IFN in months 1, 3 and 5, and then every 3 months until the first documented disease progression (arm A, suspension), or the same regimen, with chronic maintenance of immunotherapy, regardless of tumour response, until death or intolerable toxicity (arm B, maintenance). The primary endpoint was overall survival (OS); secondary endpoints were time from first progression to death (TFPTD) and tolerability. One hundred and eighty-three patients were enrolled between January 1998 and November 2003. After a median follow-up of 53.9 months, response rate, median OS and median TFPTD were 14.7% (6.3% CR) versus 11.3% (5.5% CR), 14 versus 14 months, 6 versus 5 months, in arms A and B, respectively with no significant differences between the groups. Cox regression analysis showed that the use of chemotherapy after first progression (HR 0.54; 95% CI 0.35-0.86; p = 0.008), PS = 0 (HR 0.53; 95% CI 0.35-0.81; p = 0.001) and female gender (HR 0.63; 95% CI 0.41-0.98; p = 0.038) were significantly associated with a longer TFPTD; treatment arm was not significant (HR 0.88; 95% CI 0.60-1.31; p = 0.54). Toxicity was mainly limited to WHO grades 1 or 2. Chronic maintenance immunotherapy after disease progression is feasible, but does not significantly increase OS or the TFPTD.

68 [61]. Patel, P. M., S. Sim, et al. (2008). "An evaluation of a preparation of Mycobacterium vaccae (SRL172) as an immunotherapeutic agent in renal cancer." Eur J Cancer **44**(2): 216-223.

Two studies were carried out to evaluate heat-killed Mycobacterium vaccae SRL172 as an immunotherapeutic agent for patients with metastatic, post-nephrectomy, renal cell carcinoma. In the first study, 60 patients in France and the UK received injections of SRL172, and their survival was compared with that of historical controls who had been treated either with biological response modifiers (IL-2, IFN-alpha) or chemotherapy. In the second study, 36 patients were randomised to receive treatment with IL-2 alone or IL-2 plus SRL172. Survival and adverse events related to the treatments were assessed and compared between treatment groups. The first study showed that those treated with SRL172 alone survived equally as long as those receiving IL-2 or IFN-alpha and both treatment groups survived longer than those on chemotherapy (p<0.001), a result supported by Cox's proportional hazards regression analysis. The second study, stopped early due to drug supply issues, showed that the addition of SRL172 to IL-2 made no difference to survival compared to IL-2 alone, in the limited numbers treated. Adverse events occurring in those receiving SRL172 in the first study were mild and in the second study those receiving IL-2 alone had significantly more adverse events than those receiving SRL172 plus IL-2 (p<0.001). It is concluded that SRL172 may have activity in metastatic renal cancer and has very low toxicity, making it worthy of further study.

69 [30]. Paz-Ares, L., J. G. del Muro, et al. (2010). "A cost-effectiveness analysis of sunitinib in patients with metastatic renal cell carcinoma intolerant to or experiencing disease progression on immunotherapy: perspective of the Spanish National Health System." J Clin Pharm Ther **35**(4): 429-438.

AIM: To investigate the cost-effectiveness of sunitinib (50 mg/day, schedule 4/2) vs. best supportive care (BSC) in patients with cytokine-refractory metastatic renal cell carcinoma (mRCC), from the perspective of the Spanish National Health Service. MATERIAL AND METHODS: A Markov model compared the cost-effectiveness (taking into account drugs; medical visits; laboratory tests; X-rays; terminal care; adverse event management) of sunitinib and BSC across three disease states: no progression, survival with progression and death from mRCC or other causes. RESULTS: The monthly incremental cost-effectiveness ratio (ICER) values for sunitinib treatment were euro6073/progression-free survival month, euro25,199/life years and euro34,196/quality-adjusted life years (QALY) gained. In 95% of cases, the ICER/QALY values were below the accepted euro45,000/QALY threshold. Efficacy and cost of sunitinib had the greatest impact on cost-effectiveness. CONCLUSION: Sunitinib has a good cost-effectiveness profile in mRCC. The cost per life year and QALY gained is affordable according to current effectiveness thresholds in developed countries.

70 [17]. Pedersen, A. E., A. Stryhn, et al. (2011). "Wildtype p53-specific antibody and T-cell responses in cancer patients." J Immunother **34**(9): 629-640.

Mutation in the p53 gene based on single amino acid substitutions is a frequent event in human cancer. Accumulated mutant p53 protein is released to antigen presenting cells of the immune system and anti-p53 immune responses even against wt p53 is induced and observed in a number of human cancer patients. Detection of antibodies against wt p53 protein has been used as a diagnostic and prognostic marker and discovery of new T-cell epitopes has enabled design of cancer vaccination protocols with promising results. Here, we identified wt p53-specific antibodies in various cancer patients and identified a broad range of responses against wt p53 protein and 15-mer peptides using a novel print array technology. Likewise, using bioinformatic tools in silico, we identified CD8 T-cell specificity or reactivity against HLA-A*02:01 binding peptides wt p53(65-73), wt p53(187-197), and wt p53(264-272) in breast cancer patients and against HLA-A*01:01 binding peptide wt p53(226-234) and HLA-B*07:02 binding peptide wt p53(74-82) in renal cell cancer and breast cancer patients, respectively. Finally, we analyzed antibody and T-cell responses against wt p53 15-mer peptides in patients with metastatic renal cell carcinoma who were alive with no evidence of disease after a follow-up period of minimum 5 years after treatment with IL-2 +/- IFN-alpha +/- histamine containing immunotherapy to identify novel epitopes for use in immunotherapy and for potential response biomarkers. However, none of the wt p53 reactivity observed justified use of 15-mer or was related to survival in this rare patient population.

71 [69]. Peres, E., M. H. Abidi, et al. (2007). "Reduced intensity transplantation for metastatic renal cell cancer with 2-year follow-up." J Immunother **30**(5): 562-566.

Metastatic renal cell carcinoma (RCC) is a disease that is resistant to conventional systemic therapy. Nonmyeloablative allogeneic stem cell transplant has activity in patients with metastatic RCC. This approach has been used in related donor transplantation but there are limited data on outcomes in the setting of unrelated donor (URD) transplantation. This phase II trial assessed the efficacy, safety, and responses in 16 patients, 10 related and 6 URD transplants after a reduced intensity conditioning regimen and stem cell transplant as a treatment for metastatic RCC. Sixteen patients received a conditioning consisting of either fludarabine, cyclophosphamide (n=11) or fludarabine, total body irradiation (n=5) followed by transplantation from an HLA-matched sibling donor or a unrelated HLA-donor. Cyclosporine and mycophenolate mofetil were administered as posttransplant immunosuppression. Patients were monitored for engraftment by short tandem repeat for myeloid and lymphoid lineages and clinical response was assessed by serial imaging. All patients achieved donor chimerism, 7 patients developed acute, grades 2 to 3, graft-versus-host disease. Chronic graft-versus-host disease occurred in 6 patients and transplant-related mortality was 12%. Of the 10 related donors, 1 obtained a complete response, 3 had a partial response, and 3 had stable disease. In the 6 patients who underwent URD transplant, 1 obtained a complete response and 1 patient had stable disease. These results suggest that similar outcomes are possible where either related or URD were used as the stem cell source in reduced intensity stem cell transplant for metastatic RCC.

72 [70]. Petrioli, R., L. Paolelli, et al. (2007). "Capecitabine as third-line treatment in patients with metastatic renal cell carcinoma after failing immunotherapy." Anticancer Drugs **18**(7): 817-820.

The aim of this study was to evaluate the activity and toxicity of capecitabine as third-line treatment in patients with advanced renal cell carcinoma for whom immunotherapy had failed. Twenty-one patients with metastatic clear renal cell carcinoma were enrolled. Capecitabine was administered orally twice daily at a dosage of 2500 mg/m(2) for 14 days, followed by 7 days of rest. The median number of administered cycles was five (1-13). One patient (4.8%) achieved a remission after eight treatment cycles. Stable disease was observed in nine patients (42.8%), whereas 11 progressed (52.4%). The estimated median time to progression was 3.6 months (confidence interval: 1.4 to 5.2). The estimated median overall survival was 7.2 months (confidence interval: 4.6 to 8.8). The regimen was well tolerated and no unexpected toxic effects were observed. Capecitabine as third-line treatment showed a favourable toxicity profile, but exhibited low activity in patients with advanced renal cell carcinoma after failing immunotherapy.

73 [96]. Pizza, G., C. De Vinci, et al. (2004). "Allogeneic gene-modified tumour cells in metastatic kidney cancer. Report II." Folia Biol (Praha) **50**(6): 175-183.

In a limited study, comprising only ten patients, we have previously reported that allogeneic irradiated RCC-cell-line cells, engineered to produce IL-2 (ACHN-IL-2), admixed with autologous metastatic formalin-treated tumour cells were used to vaccinate MRCC patients in progression of disease and also receiving IL-2 immunotherapy. The cells, admixed to autologous TC, were administered subcutaneously. We now report an extended study on thirty patients and one hundred thirty-one controls. Patients received 4-20 injections (mean 10 +/- 4), containing an average of 92 x 10(6) +/- 45 x 10(6) ACHN-IL-2 transfected cells (a minimum of 25 x 10(6), and a maximum of 200 x 10(6)). Autologous TC, admixed to allogeneic, were also administered by 4-16 s.c. injections (mean 7 +/- 3), i.e. a total of 12 x 10(6)-160 x 10(6) cells. Vaccination was administered during 73-1451 (307 +/- 316) days, and the follow-up continued for 1122 +/- 1240 days (106-5137). Throughout this period, the patients continued receiving the previously set immunotherapy treatment. No adverse side effects related to the treatment were noticed. One complete and four partial tumour responses were observed, as well as nine cases of stable disease. Thirteen patients died in the treated group (43%) and 63 (44%) in the control group. Responding patients resumed progression in 4-11 months and died 18 and 36 months after beginning the vaccine therapy. The Gehan Wilcoxon's test showed a significantly (P < 0.01) better survival in the vaccinated patients compared to that of the controls. Thus, we confirm, in an increased number of patients and an extensive follow-up, that our vaccination protocol is safe, devoid of adverse side effects, and promising.

74 [10]. Pohla, H., A. Buchner, et al. (2012). "High immune response rates and decreased frequencies of regulatory T cells in metastatic renal cell carcinoma patients after tumor cell vaccination." Mol Med **18**: 1499-1508.

Our previously reported phase I clinical trial with the allogeneic gene-modified tumor cell line RCC-26/CD80/IL-2 showed that vaccination was well tolerated and feasible in metastatic renal cell carcinoma (RCC) patients. Substantial disease stabilization was observed in most patients despite a high tumor burden at study entry. To investigate alterations in immune responses that might contribute to this effect, we performed an extended immune monitoring that included analysis of reactivity against multiple antigens, cytokine/chemokine changes in serum and determination of the frequencies of immune suppressor cell populations, including natural regulatory T cells (nTregs) and myeloid-derived suppressor cell subsets (MDSCs). An overall immune response capacity to virus-derived control peptides was present in 100% of patients before vaccination. Vaccine-induced immune responses to tumor-associated antigens occurred in 75% of patients, demonstrating the potent immune stimulatory capacity of this generic vaccine. Furthermore, some patients reacted to peptide epitopes of antigens not expressed by the vaccine, showing that epitope-spreading occurred in vivo. Frequencies of nTregs and MDSCs were comparable to healthy donors at the beginning of study. A significant decrease of nTregs was detected after vaccination (p = 0.012). High immune response rates, decreased frequencies of nTregs and a mixed T helper 1/T helper 2 (T(H)1/T(H)2)-like cytokine pattern support the applicability of this RCC generic vaccine for use in combination therapies.

75 [48]. Polcari, A. J., A. Gorbonos, et al. (2009). "The role of cytoreductive nephrectomy in the era of molecular targeted therapy." Int J Urol **16**(3): 227-233.

While the widespread use of imaging has resulted in an increasing number of incidentally detected renal cancers, up to one third of patients present with metastatic disease and a significant number of those with clinically localized disease subsequently develop metastasis. The prognosis for patients with metastatic disease has traditionally been poor, with a 2-year survival of only 10 to 20%. However, over the past decade a number of developments have enhanced the treatment of these patients. Phase III trials have demonstrated a significant improvement in overall survival for well-selected patients undergoing cytoreductive nephrectomy prior to immunotherapy. Meanwhile, the recent introduction of molecular targeted agents has resulted in improved response rates and tolerability compared with immunotherapy, and has prompted a re-evaluation of the role and timing of surgery in patients with advanced disease. This review examines the role of surgical therapy for patients with metastatic disease in the new era of molecular targeted therapy.

76 [108]. Porta, C., M. Zimatore, et al. (2004). "Gemcitabine and oxaliplatin in the treatment of patients with immunotherapy-resistant advanced renal cell carcinoma: final results of a single-institution Phase II study." Cancer **100**(10): 2132-2138.

BACKGROUND: Currently, there is no standard treatment for patients with advanced renal cell carcinoma (RCC) who do not experience a response to first-line immunotherapy. In the current Phase II study, the authors explored the antitumor activity of a combination of gemcitabine and oxaliplatin (L-OHP) in this setting. METHODS: Forty-two patients with RCC who had progressive disease following immunotherapy received gemcitabine (1000 mg/m2 intravenously on Days 1 and 8 every 21 days) and L-OHP (90 mg/m2 intravenously on Day 1 every 21 days) for a minimum of 2 cycles before responses were evaluated. Responses to treatment and toxicity were recorded according to the Response Evaluation Criteria in Solid Tumors and the National Cancer Institute Common Toxicity Criteria, respectively. RESULTS: No complete responses were recorded; however, 6 patients experienced a partial response (14.28%; 95% confidence interval, 5.43-28.5%), 11 patients (26.19%) had temporary stable disease as a best response, and the remaining 25 patients (59.52%) experienced progression despite receiving treatment. The median time to disease progression was 2.5 months (mean, 3.86 months; range, 1.5-11.0 months), whereas the median overall survival was 9.5 months (mean, 10.46 months; range, 4.0-22.5 months). With regard to toxicity, treatment generally was well tolerated, with only one episode of Grade 4 toxicity and expected episodes of Grade 3 toxicity, including myelosuppression and neuropathy. CONCLUSIONS: The current results suggest that the combination of gemcitabine and L-OHP possesses a certain level of activity and an acceptable toxicity profile in patients with immunotherapy-resistant advanced RCC.

77 [99]. Putz, T., R. Ramoner, et al. (2004). "Monitoring of CD4+ and CD8+ T-cell responses after dendritic cell-based immunotherapy using CFSE dye dilution analysis." J Clin Immunol **24**(6): 653-663.

CFSE dye dilution analysis and [3H] thymidine incorporation were used side by side to assess proliferative responses of peripheral blood mononuclear cells (PBMCs) after vaccination of renal cell carcinoma patients (n=6) with antigen-loaded dendritic cells. Immune responses against the control antigen keyhole limpet hemocyanin (KLH) were induced in all patients. While [3H] thymidine incorporation revealed a 4 to 977-fold increase in KLH-induced proliferation (mean: 209-fold), CFSE-labeling experiments demonstrated that the KLH-responsive population of postvaccination PBMCs represented 7-53% (mean: 23%). Combining CFSE-labeling with T-cell subset analysis confirmed the presence of CD4+ KLH-reactive T cells but also revealed a substantial population of CD8+ KLH-reactive T cells in one patient as well as minor populations of CD8+ KLH-reactive T cells in three other patients. Our data indicate that CFSE dye dilution analysis is a valuable tool for immune monitoring after dendritic cell vaccination.

78 [94]. Quan, W., Jr., M. Ramirez, et al. (2005). "Administration of high-dose continuous infusion interleukin-2 to patients age 70 or over." Cancer Biother Radiopharm **20**(1): 11-15.

High-dose bolus or continuous infusion interleukin-2-based therapy can cause capillary leak syndrome. Significant cardiovascular/hemodynamic events, including myocardial infarction, hypotension, pulmonary edema, and cardiac arrhythmia, have been described with such therapy. Concern over the toxicity of highdose interleukin-2 (IL-2) therapy has led to some clinicians excluding patients 70 years of age or over. We have treated 15 patients 70 years of age or over having an Eastern Conference Oncology Group (ECOG) performance status of 0 or 1, with therapy based on continuous infusion IL-2 18 MIU/sq m/24 hours for 72 hours. All patients underwent a pretreatment evaluation of cardiac status with a low-level stress or adenosine stress test. Cycles were typically repeated every 3 weeks for 4 cycles, then every 3-4 weeks thereafter. Patients were treated by oncology nurses in either the stem cell transplant (intermediate unit) or the oncology inpatient unit. Patient characteristics were: median age, 72 years (range, 70-83 years); tumor types: melanoma (10), kidney cancer (5); most common sites of disease: lung (11), lymph nodes (6), subcutaneous (3), liver (2); prior therapy included: none (8), outpatient IL-2 (5), other immunotherapy (4). Median number of cycles received: 3 (1-10). Most common toxicities were: fever, rigors, nausea, emesis, hypophosphatemia, and hypomagnesemia. Three patients required the use of dopamine for blood pressure support. Two patients declined further therapy. There were no treatment-related deaths. No patients required endotracheal intubation or transfer to an intensive care unit. One complete and 8 partial responses (60% response rate) have been seen. Responding sites include the lung, lymph node, intact kidney primary, and liver. Median survival has not been reached at over 14 months (range 3+-26+ months). Patients who are 70 years of age and older with an ECOG performance status of 0 or 1 are able to tolerate high-dose continuous infusion IL-2-based therapy and may respond to such treatment.

79 [76]. Quan, W. D., Jr., M. Vinogradov, et al. (2006). "Continuous infusion interleukin-2 and famotidine in metastatic kidney cancer." Cancer Biother Radiopharm **21**(5): 515-519.

Infusional interleukin-2 (IL-2) is able to elicit lymphokine-activated killer cell (LAK) cytotoxicity against kidney cancer in vitro and in vivo. Famotidine may be able to augment LAK cytotoxicity against neoplastic cells. Fifteen (15) patients were treated with continuous-infusion IL-2 (9-18 MIU/m2/24 hours) for 72 hours and famotidine 20 mg intravenously twice per day. Cycles were repeated every 3 weeks. These patients had a median age of 60 years (range, 29-72), had a median performance status of 1 (range, 0-1), and had metastatic sites, including lung, bone, lymph node, and liver. The most common toxicities of this regimen were hypophosphatemia, fever, nausea/emesis, rigors, elevated creatinine, and hypomagnesemia. One (1) complete and 6 partial responses have been seen (47% response rate). The median duration of response is 9 months. The median survival for all patients is 20 months. Five (5) patients are alive at a median of 36+ months. This combination of infusional IL-2 with famotidine is active in metastatic kidney cancer.

80 [4]. Rasmussen, S., F. Donskov, et al. (2013). "Carbon anhydrase IX specific immune responses in patients with metastatic renal cell carcinoma potentially cured by interleukin-2 based immunotherapy." Immunopharmacol Immunotoxicol **35**(4): 487-496.

The majority of clear-cell renal cell carcinomas (ccRCC) show high and homogeneous expression levels of the tumor associated antigen (TAA) carbonic anhydrase IX (CAIX), and treatment with interleukin-2 (IL-2) based immunotherapy can lead to cure in patients with metastatic renal cell carcinoma (mRCC). However, the involvement of CAIX specific CD8+ T cells and/or NK cells in the tumor eradication is unknown. We investigated T cell and antibody reactivity against overlapping 15-mer CAIX-peptides as well as HLA haplotype frequency and NK cell cytotoxicity in 11 patients with no evidence of disease (NED) following treatment with IL-2 based immunotherapy, and thus potentially cured. Immune reactivity in these patients was compared with samples from patients with dramatic tumor response obtained immediately at the cessation of therapy, samples from patients that experienced progressive disease during treatment and samples from healthy controls. We observed more focused but only weak and not consistent CAIX specific T-cells in the late observation and early observation response groups compared with the healthy control group. An increased frequency of the class II alleles HLA-DRB4 01:01, HLA-DPB 01:01 and HLA-DPB 03:01 was noted in the NED patients. In contrast, NK cytotoxicity was low even in the late observation response group as compared with controls. In particular, a HLA-B*40:01 restricted CD8+ T cell response recognizing the CAIX- derived peptide SEEEGSLKL was identified. This may have interest in future cancer vaccines, but more studies are needed to elucidate the immunological mechanisms of action in potentially cured patients treated with an immunotherapeutic agent.

81 [73]. Recchia, F., G. Saggio, et al. (2007). "Multicenter phase II study of chemo-immunotherapy in the treatment of metastatic renal cell carcinoma." J Immunother **30**(4): 448-454.

The purpose of this study was to evaluate the potential efficacy of a chemo-immunotherapy regimen for the treatment of metastatic renal cell carcinoma (MRCC). Forty-one patients with progressing MRCC and with a median age of 63 years were recruited. Planned treatment consisted of 6 courses of capecitabine 1000 mg/m twice daily on days 1 to 14 every 4 weeks, pegylated alpha-interferon 2b 50 microg every week, interleukin-2 1.8 M IU subcutaneously, and oral 13-cis-retinoic acid 0.5 mg/kg, all given 5 days/wk, 3 weeks of each month. After 6 courses of concomitant biochemotherapy, biotherapy was continued in patients who had a clinical benefit. The primary end point was response; secondary end points were the evaluation of the immunologic parameters, toxicity, progression-free, and overall survival. The treatment was well-tolerated. Grade 3 and 4 neutropenia and thrombocytopenia occurred in 5% and 7% of patients, respectively. The overall response rate in the 41 evaluable patients was 53.6% (95% confidence interval 37%-69%). Median progression-free and overall survivals were 14.7 and 27.8 months, respectively. A sustained improvement in all evaluated immunologic parameters was observed in the 36 patients treated with maintenance biotherapy. Six cycles of biochemotherapy, being followed by maintenance immunotherapy is well-tolerated and shows significant activity in patients with MRCC.

82 [86]. Ribas, A., L. H. Camacho, et al. (2005). "Antitumor activity in melanoma and anti-self responses in a phase I trial with the anti-cytotoxic T lymphocyte-associated antigen 4 monoclonal antibody CP-675,206." J Clin Oncol **23**(35): 8968-8977.

PURPOSE: Cytotoxic T lymphocyte-associated antigen 4 (CTLA4) blockade with CP-675,206, a fully human anti-CTLA4 monoclonal antibody, may break peripheral immunologic tolerance leading to effective immune responses to cancer in humans. A phase I trial was conducted to test the safety of CP-675,206. PATIENTS AND METHODS: Thirty-nine patients with solid malignancies (melanoma, n = 34; renal cell, n = 4; colon, n = 1) received an intravenous (IV) infusion of CP-675,206 at seven dose levels. The primary objective was to determine the maximum-tolerated dose and the recommended phase II dose. RESULTS: Dose-limiting toxicities and autoimmune phenomena included diarrhea, dermatitis, vitiligo, panhypopituitarism and hyperthyroidism. Two patients experienced complete responses (maintained for 34+ and 25+ months), and there were two partial responses (26+ and 25+ months) among 29 patients with measurable melanoma. There have been no relapses thus far after objective response to therapy. Four other patients had stable disease at end of study evaluation (16, 7, 7, and 4 months). Additionally, five patients had extended periods without disease progression (36+, 35+, 26+, 24+, and 23+ months) after local treatment of progressive metastases. Longer systemic exposure to CP-675,206 achieved in higher dose cohorts predicted for a higher probability of response. CONCLUSION: CP-675,206 can be administered safely to humans as a single IV dose up to 15 mg/kg, resulting in breaking of peripheral immune tolerance to self-tissues and antitumor activity in melanoma.

83 [80]. Rini, B. I., S. Halabi, et al. (2006). "Adoptive immunotherapy by allogeneic stem cell transplantation for metastatic renal cell carcinoma: a CALGB intergroup phase II study." Biol Blood Marrow Transplant **12**(7): 778-785.

A graft-versus-tumor effect through nonmyeloablative allogeneic stem cell transplantation (N-SCT) in metastatic renal cell carcinoma (RCC) has been reported. An Intergroup phase II trial was undertaken to define further the feasibility, toxicity and efficacy of this approach in a multi-institutional setting, Patients with cytokine-refractory, metastatic RCC were treated with N-SCT. The conditioning regimen was fludarabine 30 mg . m(-2) . d(-1) on day (d) -7 through d -3 and cyclophosphamide 60 mg . kg(-1) . d(-1) on d -4 and d -3. Patients received 2-8 x 10(6) CD34+ cells/kg of granulocyte colony-stimulating factor mobilized stem cells from a 6/6 HLA-matched sibling donor. Immunosuppression after transplantation included tacrolimus and methotrexate. Twenty-two patients were enrolled at 14 institutions. Greater than 90% donor T-cell chimerism was observed in 17 of 19 evaluable patients (89%) by d +120. No objective response was observed. Acute graft-versus-host disease (GVHD) was observed in 11 patients (50%). Chronic GVHD was reported in 5 patients (23%). There was 1 patient death from liver failure secondary to chronic GVHD. Regimen-related mortality was 2 of 22 (9%; liver failure, sepsis). Median survival time was 5.5 months (95% confidence interval, 3.9-12.0 months) and the median time to progression was 3.0 months (95% confidence interval, 2.3-4.2 months). N-SCT for metastatic RCC is feasible in a multi-institutional setting. Adequate donor T-cell engraftment was achieved in most patients before disease progression. A graft-versus-tumor effect was not observed in this study despite acute and chronic GVHD, thus highlighting the need for further understanding of this approach. Allogeneic SCT remains investigational in RCC.

84 [31]. Rossi, J. F., S. Negrier, et al. (2010). "A phase I/II study of siltuximab (CNTO 328), an anti-interleukin-6 monoclonal antibody, in metastatic renal cell cancer." Br J Cancer **103**(8): 1154-1162.

BACKGROUND: Serum interleukin (IL)-6 levels correlate with disease outcomes in renal cell carcinoma (RCC) patients. Siltuximab, a chimeric, murine-human mAb against IL-6, was evaluated in a three-part phase I/II study in patients with progressive metastatic RCC. METHODS: In part 1, 11 patients received 1, 3, 6, or 12mgkg-(1) at weeks 1, 4 and q2w x 2 thereafter; in part 2, 37 patients randomly received 3 or 6 mgkg-(1) q3w x 4; in part 3, 20 low-risk patients received 6mgkg-(1) q2w x 6. Modified WHO response criteria were assessed at weeks 7, 11, the 6-week follow-up, and when clinically indicated. RESULTS: Siltuximab was well tolerated overall, with no maximum tolerated dose or immune response observed. In all, 5 out of 11, 17 out of 37, and 9 out of 20 patients in parts 1, 2, and 3, respectively, received extended treatment beyond 4-6 initial infusions. In part 2, stable disease (SD) (>/=11weeks) or better was achieved by 11 out of 17 (65%) 3 mgkg-(1) treated patients (one partial response (PR) ~8 months, 10 SD) and 10 out of 20 (50%) 6mgkg-(1) treated patients (10 SD). In part 3, documented complete or PR was not observed, but 13 out of 20 (65%) patients achieved SD. CONCLUSION: Siltuximab stabilised disease in >50% of progressive metastatic RCC patients. One PR was observed. Given the favourable safety profile of siltuximab and poor correlation of tumour shrinkage with clinical benefit demonstrated for other non-cytotoxic therapies, further evaluation of dose-escalation strategies and/or combination therapy may be considered for patients with RCC.

85 [91]. Rzepecki, P., J. Zolnierek, et al. (2005). "Allogeneic non-myeloablative hematopoietic stem cell transplantation for treatment of metastatic renal cell carcinoma -- single center experience." Neoplasma **52**(3): 238-242.

We evaluated the efficacy of allogeneic non-myeloablative stem cell transplantation (NST) in patients with metastatic renal cell carcinoma (RCC). A total of 5 patients received blood stem cells from HLA identical siblings. Conditioning consisted of: cyclophosphamide 60 mg/kg/d, days -7 to -6 and fludarabine 25 mg/m2/d for consecutive days [days -5, -4, -3, -2, -1]. The median CD34+ cell dose was 3.34 million/kg. Immunosuppression consisted of cyclosporine A and methotrexate. Among all, four patients achieved full donor chimerism with a median of 89 days. One patient rejected the graft and received the second transplantation. Grade II-III acute GVHD occured in 3 patients. None of patients achieved complete or partial response and there were only two mixed responses. All patients died due to cancer progression. There were no transplant-related deaths. Summarising, NST regimen allows allogeneic engraftment with low treatment related mortality in this high-risk population of patients. Acute and chronic GVHD are the major morbidities. Progression is common after NST in unselected patients with advanced RCC. However, regression of some metastases suggests that the graft versus tumor effect may occur after this type of treatment. At present such a procedure should be considered as an experimental approach.

86 [67]. Salot, S., C. Laplace, et al. (2007). "Large scale expansion of gamma 9 delta 2 T lymphocytes: Innacell gamma delta cell therapy product." J Immunol Methods **326**(1-2): 63-75.

gamma9delta2 T lymphocytes are non-conventional lymphocytes presenting a direct cytotoxic effect against a broad range of tumour targets. These cells also secrete inflammatory cytokines that can boost the other components of the immune system. In contrast to conventional CD8(+) T cells, the cytotoxic effect of gamma9delta2 T lymphocytes does not depend on the expression of major histocompatibility complex molecules by target tumour cells. INNACELL gammadeltatrade mark is a cell therapy product obtained by ex vivo amplification of mononuclear cells. The stimulation is achieved by a specific synthetic agonist of gamma9delta2 T lymphocytes, bromohydrin pyrophosphate (BrHPP). After a single stimulation with BrHPP, gamma9delta2 T lymphocytes are expanded for 2 weeks in a closed system in culture medium with interleukin-2 (IL-2). On day 15, cells are washed and harvested in 4% human serum albumin. In this manufacturing process, the total cell population is expanded by approximately 10-fold and gamma9delta2 T lymphocytes undergo a specific 1000-fold expansion, corresponding to a gamma9delta2 T lymphocyte enrichment of more than 70% at the end of the culture. This manufacturing process is much simpler than most current cellular therapy approaches using conventional CD8(+) T-cell lines or clones: there is no final or initial separation, no purification step and no use of feeder cells; the specific T-cell receptor-mediated signal provided by BrHPP is sufficient to trigger the IL-2-dependent expansion of the gamma9delta2 subset, which then becomes predominant in the cell culture in large amounts.

87 [26]. Scherr, A. J., J. P. Lima, et al. (2011). "Adjuvant therapy for locally advanced renal cell cancer: a systematic review with meta-analysis." BMC Cancer **11**: 115.

BACKGROUND: Many adjuvant trials have been undertaken in an attempt to reduce the risk of recurrence among patients who undergo surgical resection for locally advanced renal cancer. However, no clear benefit has been identified to date. This systematic review was conducted to examine the exact role of adjuvant therapy in renal cancer setting. METHODS: Randomized controlled trials were searched comparing adjuvant therapy (chemotherapy, vaccine, immunotherapy, biochemotherapy) versus no active treatment after surgery among renal cell cancer patients. Outcomes were overall survival (OS), disease-free survival (DFS), and severe toxicities. Risk ratios (RR), hazard ratios (HR) and 95% confidence intervals were calculated using a fixed-effects meta-analysis. Heterogeneity was measured by I2. Different strategies of adjuvant treatment were evaluated separately. RESULTS: Ten studies (2,609 patients) were included. Adjuvant therapy provided no benefits in terms of OS (HR 1.07; 95%CI 0.89 to 1.28; P = 0.48 I2 = 0%) or DFS (HR 1.03; 95%CI 0.87 to 1.21; P = 0.77 I2 = 15%) when compared to no treatment. No subgroup analysis (immunotherapy, vaccines, biochemotherapy and hormone therapy) had relevant results. Toxicity evaluation depicted a significantly higher frequency of serious adverse events in the adjuvant group. CONCLUSIONS: This analysis provided no support for the hypothesis that the agents studied provide any clinical benefit for renal cancer patients although they increase the risk of toxic effects. Randomized trials are underway to test targeted therapies, which might open a new therapeutic frontier. Until these trials yield results, no adjuvant therapy can be recommended for patients who undergo surgical resection for renal cell cancer.

88 [104]. Schiller, G. J., R. Malone, et al. (2004). "Re: allogenic transplantation after nonmyelosuppressive conditioning--the effect of single-agent pentostatin." Biol Blood Marrow Transplant **10**(8): 576-577.

89 [93]. Schrader, A. J., A. Heidenreich, et al. (2005). "Application of thalidomide/interleukin-2 in immunochemotherapy-refractory metastatic renal cell carcinoma." Anticancer Drugs **16**(5): 581-585.

Thalidomide has been reported to yield anti-tumor activity in advanced renal cell carcinoma (RCC). We evaluated safety and efficacy of a combination therapy comprising interleukin (IL)-2 and thalidomide in patients with metastatic RCC refractory to both immuno- and chemotherapy. Twelve patients with progressive metastatic RCC who had failed prior treatment with immunochemotherapy and desired further active therapy were enrolled in this study. Oral thalidomide was started at 200 mg/day and escalated after 2 days to 400 mg/day at week 0. IL-2 at 7 MIU/m was given by s.c. injection, starting at week 1, days 1-5, weeks 1-4, with rest from IL-2 at weeks 5 and 6. Response was assessed every two therapy cycles. Ten patients were evaluable for response. There was no objective response; four patients showed stable disease for 14+, 11+, 10+ and 9 months, respectively. Toxicities were predominantly grade I-II, and included somnolence and constipation, as well as flu-like symptoms associated with IL-2. However, one patient developed serious constipation which led to a paralytic ileus and discontinuation of treatment. Another patient left the study after 7 weeks due to increasing disorientation/confusion. Eight patients required IL-2 dose reduction. Time on therapy ranged from 3 to 44 weeks (median 20 weeks). Median overall survival was 12+ months. At present, all patients have discontinued treatment. We conclude that outpatient administration of thalidomide/IL-2 is feasible in patients with heavily pretreated and progressive RCC who desire further active treatment. However, toxicity and costs are considerable, and clinical benefit is uncertain. Therefore, thalidomide/IL-2 might not represent a promising therapeutic approach for this subgroup of patients.

90 [44]. Schwaab, T., A. Schwarzer, et al. (2009). "Clinical and immunologic effects of intranodal autologous tumor lysate-dendritic cell vaccine with Aldesleukin (Interleukin 2) and IFN-{alpha}2a therapy in metastatic renal cell carcinoma patients." Clin Cancer Res **15**(15): 4986-4992.

PURPOSE: To evaluate the clinical and immunologic outcomes of DC (dendritic cell) vaccine with interleukin (IL)-2 and IFN-alpha 2a in metastatic renal cell carcinoma patients. EXPERIMENTAL DESIGN: Eighteen consented and eligible patients were treated. Peripheral blood monocytes were cultured ex vivo into mature DCs and loaded with autologous tumor lysate. Treatment consisted of five cycles of intranodal vaccination of DCs (1 x 10(7) cells/1 mL Lactated Ringer's solution), 5-day continuous i.v. infusion of IL-2 (18MiU/m2), and three s.c. injections of IFN-alpha 2a (6MiU) every other day. Response Evaluation Criteria in Solid Tumors criteria were used for disease assessment. Correlative immunologic end points included peripheral blood lymphocyte cell phenotype and function as well as peripheral blood anti-renal cell carcinoma antibody and cytokine levels. RESULTS: All patients received between two and five treatment cycles. Toxicities consisted of known and expected cytokine side effects. Overall objective clinical response rate was 50% with three complete responses. Median time to progression for all patients was 8 months, and median survival has not been reached (median follow up of 37+ months). Treatment-related changes in correlative immunologic end points were noted and the level of circulating CD4(+) T regulatory cells had a strong association with outcome. Pre-IP-10 serum levels approached significance for predicting outcome. CONCLUSIONS: The clinical and immunologic responses observed in this trial suggest an interaction between DC vaccination and cytokine therapy. Our data support the hypothesis that modulation of inflammatory, regulatory, and angiogenic pathways are necessary to optimize therapeutic benefit in renal cell carcinoma patients. Further exploration of this approach is warranted.

91 [84]. Shaughnessy, P., W. Alexander, et al. (2006). "Phase I and pharmacokinetic study of once-daily dosing of intravenously administered busulfan in the setting of a reduced-intensity preparative regimen and allogeneic hematopoietic stem cell transplantation as immunotherapy for renal cell carcinoma." Mil Med **171**(2): 161-165.

We performed a Phase I and pharmacokinetic study of once-daily, intravenously administered busulfan in the setting of a reduced-intensity preparative regimen and matched sibling donor allogeneic stem cell transplantation for treatment of metastatic renal cell carcinoma. Seven male patients with metastatic renal cell carcinoma received intravenously administered busulfan at 3.2 mg/kg once daily on day -10 and day -9, fludarabine at 30 mg/m2 on day -7 through day -2, and equine antithymocyte globulin at 15 mg/kg per day on day -5 through day -2. The mean area under the plasma concentration-time curve (AUC) and the half-life of the first dose of intravenously administered busulfan were 6,253 microM x minute (range, 5,036-7,482 microM x minute) and 3.37 hours (range, 2.54-4.00 hours), respectively. The AUC was higher than predicted from extrapolation of AUC data for the same total dose of intravenously administered busulfan divided into four doses daily. Patients experienced greater than expected regimen-related toxicity for a reduced-intensity preparative regimen, and the study was stopped. In conclusion, this preparative regimen was associated with unacceptable regimen-related toxicity among patients with metastatic renal cell carcinoma.

92 [2]. Soerensen, A. V., F. Donskov, et al. (2014). "Improved overall survival after implementation of targeted therapy for patients with metastatic renal cell carcinoma: results from the Danish Renal Cancer Group (DARENCA) study-2." Eur J Cancer **50**(3): 553-562.

AIM: To evaluate the implementation of targeted therapy on overall survival (OS) in a complete national cohort of patients with metastatic renal cell carcinoma (mRCC). METHODS: All Danish patients with mRCC referred for first line treatment with immunotherapy, TKIs or mTOR-inhibitors between 2006 and 2010 were included. Baseline and outcome data were collected retrospectively. Prognostics factors were identified using log-rank tests and Cox proportional hazard model. Differences in distributions were tested with the Chi-square test. RESULTS: 1049 patients were referred; 744 patients received first line treatment. From 2006 to 2010 we observed a significant increase in the number of referred patients; a significant increase in treated patients (64% versus 75%, P=0.0188); a significant increase in first line targeted therapy (22% versus 75%, P<0.0001); a significant increase in second line treatment (20% versus 40%, P=0.0104), a significant increased median OS (11.5 versus 17.2 months, P=0.0435) whereas survival for untreated patients remained unchanged. Multivariate analysis validated known prognostic factors. Moreover, treatment start years 2008 (HR 0.74, 95% CI, 0.55-0.99; P=0.0415), 2009 (HR 0.72, 95% CI, 0.54-0.96; P=0.0277) and 2010 (HR 0.63, 95% CI, 0.47-0.86; P=0.0035) compared to 2006, and more than two treatment lines received for patients with performance status 0-1 (HR 0.76, 95% CI, 0.58-0.99; P=0.0397) and performance status 2-3 (HR 0.19, 95% CI, 0.06-0.60; P=0.0051) were significantly associated with longer OS. CONCLUSION: This retrospective study documents that the implementation of targeted therapy has resulted in significantly improved treatment rates and overall survival in a complete national cohort of treated mRCC patients.

93 [39]. Soleimani, A., A. Berntsen, et al. (2009). "Immune responses in patients with metastatic renal cell carcinoma treated with dendritic cells pulsed with tumor lysate." Scand J Immunol **70**(5): 481-489.

Patients with metastatic renal cell carcinoma (mRCC) have a limited life expectancy but still a subset of these patients develop immune and clinical responses after immunotherapy including dendritic cell (DC) vaccination. In a recently published phase I/II trials, fourteen HLA-A2 negative patients with progressive mRCC were vaccinated with autologous DC pulsed with allogeneic tumour lysate. Low-dose IL-2 administered subcutaneously was given concomitantly. In this study, we analysed lysate specific proliferation of PBMCs from these patients together with the TH1/TH2 balance of the responding T cells. Also, serum concentrations of IL-10, IL-12, IL-15, IL-17 and IL-18 from these patients and additional thirteen HLA-A2 positive mRCC patients treated with autologous DC pulsed with survivin and telomerase peptides were analysed during vaccination to identify systemic immune responses and potential response biomarkers. In HLA-A2 negative mRCC patients a spontaneous predominance of TH1 secreting tumour lysate specific T cells was observed prior to vaccination in patients attaining stable disease (SD) during treatment whereas patients with continued progressive disease (PD) had a mixed TH1/TH2 response. The TH1/TH2 balance was unchanged during vaccination also when tumour lysate specific T cell responses increased. An increase in IL-12, IL-17 and IL-18 serum concentrations was observed during vaccination but no difference between patients with SD and PD was observed. IL-10 or IL-15 was not measurable in serum.

94 [90]. Spanknebel, K., K. Y. Cheung, et al. (2005). "Initial clinical response predicts outcome and is associated with dose schedule in metastatic melanoma and renal cell carcinoma patients treated with high-dose interleukin 2." Ann Surg Oncol **12**(5): 381-390.

BACKGROUND: High-dose interleukin (IL)-2 is an effective agent for the treatment of metastatic malignant melanoma and renal cell carcinoma. This study evaluated the outcomes of patients receiving two commonly used intravenous IL-2 schedules that have never been directly compared. METHODS: Forty-seven metastatic malignant melanoma and renal cell carcinoma patients were identified from a prospective database who underwent high-dose IL-2 therapy (720,000 or 600,000 IU/kg) during 1999 to 2003. Disease-specific survival (DSS) was calculated by the Kaplan-Meier method with the log-rank test on an intention-to-treat basis. Multivariate Cox regression analysis of prognostic variables associated with outcome was performed. Factors associated with initial response and prevention of disease progression were determined. RESULTS: Objective response (5 partial and 5 mixed) or disease stabilization was noted in 9 (20%) and 10 (22%), respectively, of 46 assessable patients after 1 course of therapy. Four patients (22%) achieved disease-free status after the third course of IL-2 (n = 1) or surgical resection of confined metastatic disease (n = 3). At 19.1 months' median follow-up, factors associated with improved DSS included an initial clinical response to IL-2 therapy (P < .001) and a higher administered dose (P = .04). Patients who received 720,000 IU/kg were more likely to experience an initial major objective response (P = .03) and disease stabilization (P = 0.03) independent of the tumor treated. Objective response early in the course of therapy was the only independent predictor of tumor-related mortality (P = .004). CONCLUSIONS: The initial clinical response to IL-2 therapy is an independent predictor of improved outcome associated with DSS and the 720,000 IU/kg dose. These results support further prospective trials with increased IL-2 dose schedules in a larger cohort of patients.

95 [58]. Spreafico, F., G. Bisogno, et al. (2008). "Treatment of high-risk relapsed Wilms tumor with dose-intensive chemotherapy, marrow-ablative chemotherapy, and autologous hematopoietic stem cell support: experience by the Italian Association of Pediatric Hematology and Oncology." Pediatr Blood Cancer **51**(1): 23-28.

BACKGROUND: We evaluated an intensified chemotherapy strategy in children with Wilms tumor who relapsed with high-risk features. PROCEDURES: From January 2001 to June 2006, we treated 20 consecutive children with reinduction chemotherapy (using ifosfamide/carboplatin/etoposide in 15/20 cases), with (n = 15) or without (n = 5) subsequent high-dose chemotherapy and hematopoietic stem cell support, surgery where feasible, and radiation therapy. The median time to relapse was 10 months after nephrectomy. All but two children initially received doxorubicin as first-line therapy. RESULTS: All patients were assessed for outcome: 13 are currently alive, 12 of them in remission a median 25 months since their relapse, one with progressing tumor. The treatment was unsuccessful in eight children: the disease progressed during reinduction in three, and relapsed in five. There was one toxic death. All transplanted patients engrafted to a neutrophil count >0.5 x 10(3)/microl after a median 11 days, and to an unsustained platelet count >25,000/microl after a median of 13 days. Three-year disease-free and overall survival rates were 56 +/- 12% and 55 +/- 13%, respectively. Neither recurrence within 12 months of nephrectomy nor extra-lung recurrence negatively affected outcome. A survival advantage was demonstrated in patients without disease evidence prior to transplant. CONCLUSION: A disease-free survival rate nearing 50% is a realistic target in children with high-risk recurrent Wilms tumor. The benefit of autologous hematopoietic stem cell transplantation for consolidation deserves to be investigated in a randomized, controlled study.

96 [12]. Stillebroer, A. B., O. C. Boerman, et al. (2013). "Phase 1 radioimmunotherapy study with lutetium 177-labeled anti-carbonic anhydrase IX monoclonal antibody girentuximab in patients with advanced renal cell carcinoma." Eur Urol **64**(3): 478-485.

BACKGROUND: Patients with metastatic clear cell renal cell carcinoma (ccRCC) have a dismal prognosis. Therefore, new and less toxic treatments are needed. OBJECTIVE: We determined the maximum tolerated dose (MTD) and potential therapeutic efficacy of multiple infusions of lutetium 177 ((177)Lu)-girentuximab (cG250) on various dose levels in a phase 1 trial in patients with progressive metastasized ccRCC. DESIGN, SETTING, AND PARTICIPANTS: In this uncontrolled case series in 23 patients with progressive ccRCC metastases, cG250 accumulation was verified by diagnostic indium 111-cG250 imaging. Patients then received a high-activity dose of (177)Lu-cG250. INTERVENTION: Groups of three patients received (177)Lu-cG250, starting at a dose level of 1110 MBq/m(2)(177)Lu-cG250, with dose increments of 370 MBq/m(2) per group. In the absence of persistent toxicity, progressive disease, and accelerated blood clearance, patients were eligible for retreatment after 3 mo with 75% of the previous activity dose. Patients could receive a total of three treatment cycles. OUTCOME MEASUREMENTS AND STATISTICAL ANALYSIS: Determination of the MTD was the primary and therapeutic efficacy was the secondary outcome measurement of the study. RESULTS AND LIMITATIONS: The MTD was 2405 MBq/m(2) because higher doses resulted in dose-limiting myelotoxicity. Some patients received second (13 of 23 [56%]) and third (4 of 23 [17%]) treatment cycles. Most patients (17 of 23 [74%]) demonstrated stable disease 3 mo after the first treatment, and one patient showed a partial response that lasted for 9 mo. Mean growth of target tumor lesions was reduced from 40.4% (95% confidence interval [CI], +/- 17.0) during the last 3 mo before study entry to 5.5% (95% CI, +/- 5.3; p<0.001) at 3 mo after the first treatment cycle. No major nonhematologic side effects were observed. CONCLUSIONS: (177)Lu-cG250 radioimmunotherapy in metastatic ccRCC patients is well tolerated at an activity dose level as high as 2405 MBq/m(2) (MTD). Radioimmunotherapy with (177)Lu-cG250 may stabilize previously progressive metastatic ccRCC.

97 [15]. Stillebroer, A. B., C. M. Zegers, et al. (2012). "Dosimetric analysis of 177Lu-cG250 radioimmunotherapy in renal cell carcinoma patients: correlation with myelotoxicity and pretherapeutic absorbed dose predictions based on 111In-cG250 imaging." J Nucl Med **53**(1): 82-89.

This study aimed to estimate the radiation absorbed doses to normal tissues and tumor lesions during radioimmunotherapy with (177)Lu-cG250. Serial planar scintigrams after injection of (111)In-cG250 or (177)Lu-cG250 in patients with metastasized renal cell carcinoma were analyzed quantitatively. The estimated radiation doses were correlated with observed hematologic toxicity. In addition, the accuracy of the predicted therapeutic absorbed doses, based on diagnostic (111)In-cG250 data, were determined. METHODS: Twenty patients received a diagnostic tracer activity of (111)In-cG250 (185 MBq), followed by radioimmunotherapy with (177)Lu-cG250. The administered activity of (177)Lu-cG250 was escalated by entering 3 patients at each activity level starting at 1,110 MBq/m(2), with increments of 370 MBq/m(2). After each diagnostic and therapeutic administration, whole-body scintigraphic images and pharmacokinetic data were acquired. Hematologic toxicity was graded using the Common Toxicity Criteria, version 3.0. Diagnostic (111)In-cG250 data were used to simulate (177)Lu and (90)Y data by correcting for the difference in physical decay. Absorbed doses were calculated for the whole body, red marrow, organs, and tumor metastases for the therapeutic (177)Lu-cG250, simulated (177)Lu-cG250, and simulated (90)Y-cG250 data. RESULTS: Observed hematologic toxicity, especially platelet toxicity, correlated significantly with the administered activity (r = 0.85), whole-body absorbed dose (r = 0.65), and red marrow dose (r = 0.62 and 0.75). An inverse relationship between the mass and absorbed dose of the tumor lesions was observed. Calculated mean absorbed doses were similar for the simulated and measured (177)Lu-cG250 data. Absorbed doses (whole body and red marrow) based on the simulated (177)Lu-cG250 data correlated with the observed platelet toxicity (r = 0.65 and 0.82). The tumor-to-red marrow dose ratio was higher for radioimmunotherapy with (177)Lu-cG250 than for radioimmunotherapy with (90)Y-cG250, indicating that (177)Lu has a wider therapeutic window for radioimmunotherapy with cG250 than (90)Y. CONCLUSION: In patients with metastasized renal cell carcinoma, hematologic toxicity after treatment with (177)Lu-cG250 can be predicted on the basis of administered activity and whole-body and red marrow-absorbed dose. Diagnostic (111)In-cG250 data can be used to accurately predict absorbed doses and myelotoxicity of radioimmunotherapy with (177)Lu-cG250. These estimations indicate that in these patients, higher radiation doses can be guided to the tumors with (177)Lu-cG250 than with (90)Y-cG250.

98 [5]. Tang, X., T. Liu, et al. (2013). "Adoptive cellular immunotherapy in metastatic renal cell carcinoma: a systematic review and meta-analysis." PLoS One **8**(5): e62847.

PURPOSE: Metastatic renal cell carcinoma (mRCC), as one of the most immunogenic tumors has been the focus of adoptive cellular immunotherapy (ACI), but the effects of ACI on objective response and survival in patients with mRCC are still controversial. Therefore, a systematic review and meta-analysis was performed to address this issue. METHODS: A search was conducted in the PubMed database for randomized clinical trials (RCTs) with ACI in mRCC. All included articles in this study were assessed according to the selection criteria and were divided into two groups: ACI versus no ACI. Outcomes were toxicity, objective response, 1-, 3- and 5-year survival. Risk ratio (RR) and 95% confidence intervals (CI) were calculated using a fixed-effects meta-analysis. Heterogeneity was measured by value of I(2) or P. RESULTS: 4 studies (469 patients) were included. Most of ACI-related adverse reactions were grade 1 or 2 and reversible. ACI provided significant benefit in terms of objective response (RR = 1.65; 95% CI, 1.15 to 2.38; P = 0.007, I(2) = 49%), 1-year survival (RR = 1.30; 95% CI, 1.12 to 1.52; P = 0.0008, I(2) = 0%), 3-year survival (RR = 2.76; 95% CI, 1.85 to 4.14; P<0.00001, I(2) = 46%) and 5-year survival (RR = 2.42; 95% CI, 1.21 to 4.83; P = 0.01, I(2) = 28%). CONCLUSIONS: ACI may be a safe and effective treatment for improving objective response, 1-, 3- and 5-year survival in patients with mRCC. Besides, five obstacles for ACI, including high degree of personalization, unsuitable WHO/RECIST response criteria, inadequate identification of tumor-associated antigens (TAAs), lack of effective combination treatments and less attention paid to the quality of ACI products, should be overcome during the successful development of more potent ACI for cancer in the future.

99 [103]. Tani, K., M. Azuma, et al. (2004). "Phase I study of autologous tumor vaccines transduced with the GM-CSF gene in four patients with stage IV renal cell cancer in Japan: clinical and immunological findings." Mol Ther **10**(4): 799-816.

We produced lethally irradiated retrovirally GM-CSF-transduced autologous renal tumor cell vaccines (GVAX) from six Japanese patients with stage IV renal cell cancer (RCC). Four patients received GVAX ranging from 1.4 x 10(8) to 3.7 x 10(8) cells on 6-17 occasions. Throughout a total of 48 vaccinations, there were no severe adverse events. After vaccination, DTH skin tests became positive to autologous RCC (auto-RCC) in all patients. The vaccination sites showed significant infiltration by CD4(+) T cells, eosinophils, and HLA-DR-positive cells. The kinetic analyses of cellular immune responses using peripheral blood lymphocytes revealed an enhanced proliferative response against auto-RCC in four patients, and cytotoxicity against auto-RCC was augmented in three patients. T cell receptor beta-chain analysis revealed oligoclonal expansion of T cells in the peripheral blood, skin biopsy specimens from DTH sites, and tumors. Western blot analysis demonstrated the induction of a humoral immune response against auto-RCC. Two of the four patients are currently alive 58 and 40 months after the initial vaccination with low-dose interleukin-2. Our results suggest that GVAX substantially enhanced the antitumor cellular and humoral immune responses, which might have contributed to the relatively long survival times of our patients in the present study.

100 [54]. Tannir, N. M., P. F. Thall, et al. (2008). "A phase II trial of gemcitabine plus capecitabine for metastatic renal cell cancer previously treated with immunotherapy and targeted agents." J Urol **180**(3): 867-872; discussion 872.

PURPOSE: We assessed the clinical activity and safety of gemcitabine plus capecitabine in patients with metastatic renal cell cancer previously treated with immunotherapy. MATERIALS AND METHODS: In this phase II trial patients received 1,000 mg/m(2) gemcitabine intravenously on days 1, 8 and 15, plus 830 mg/m(2) capecitabine orally twice daily on days 1 to 21 of 28-day cycles. The primary end point was progression-free survival time. Secondary end points included overall survival time, objective response rate and toxicity. RESULTS: Of 84 patients enrolled 83 were evaluable for response and toxicity. A total of 65 patients had intermediate or poor risk prognosis. Median progression-free survival and overall survival were 4.6 (95% CI 3.7-7.3) and 17.9 months (95% CI 13.2-23.6), respectively. There were 6 partial responses and 1 complete response (objective response rate 8.4% [95% CI 3.5-16.6]). Two patients remain in unmaintained remission close to 3 years from the initiation of gemcitabine plus capecitabine treatment. On multivariate analysis more than 3 disease sites were significantly associated with shorter progression-free survival and patients with thrombocytosis, more than 3 disease sites or anemia had a significantly increased risk of death. Adverse events occurring at least once in more than 5% of patients included grade 3 or greater neutropenia (83%), grade 2 or greater hand-foot syndrome (13%), grade 3 or greater thrombocytopenia (12%), grade 3 or greater thromboembolic events (8%), grade 3 or greater fatigue (8%) and grade 2 or greater mucositis (6%). CONCLUSIONS: At the doses and schedule tested gemcitabine plus capecitabine demonstrated modest clinical activity in metastatic renal cell cancer after cytokine failure and produced significant neutropenia. A modified gemcitabine plus capecitabine regimen may be evaluated in patients with metastatic renal cell cancer after failure of approved targeted therapies.

101 [64]. Thistlethwaite, F. C., E. Elkord, et al. (2008). "Adoptive transfer of T(reg) depleted autologous T cells in advanced renal cell carcinoma." Cancer Immunol Immunother **57**(5): 623-634.

PURPOSE: CD4(+)CD25(+) regulatory T (T(reg)) cells are present in increased numbers in patients with advanced cancer and CD25(+) T cell depletion potentiates tumour immunity in animal models. The aim of this study was to assess the feasibility and safety of adoptive transfer of CD25(+) depleted autologous T cells in patients with advanced renal cell carcinoma and to examine resulting changes in lymphocyte subsets. PATIENTS AND METHODS: Six patients with advanced renal cell carcinoma underwent leukapheresis followed by conditioning chemotherapy with cyclophosphamide and fludarabine. The autologous leukapheresis product was depleted of CD25(+) cells using CliniMACS System then re-infused into the patient. RESULTS: Efficient CD25(+) depletion from all leukapheresis products was achieved and 0.55-5.87 x 10(7)/kg CD3(+) cells were re-infused. Chemotherapy related haematological toxicity was observed, but blood counts recovered in all patients allowing discharge after a mean inpatient stay of 21 days. One patient subsequently developed a rapidly progressive neurological syndrome. A transient reduction in CD25(+) subset was noted in the peripheral blood of 5 out of 6 patients with evidence of increased T cell responses to PHA in 4 out of 6 patients. One patient showed increased specific proliferative responses to the tumour associated antigen h5T4 coinciding with the nadir of T(reg) cells. CONCLUSIONS: Given the transient nature of the reduction in CD25(+) subset and the observed toxicity there is a need to explore further strategies to improve the safety and efficacy of this approach. Nevertheless, the results provide proof of concept in potentiation of tumour antigen T cell responses when T(reg) cell levels are depleted.

102 [56]. Thompson, J. A., B. D. Curti, et al. (2008). "Phase I study of recombinant interleukin-21 in patients with metastatic melanoma and renal cell carcinoma." J Clin Oncol **26**(12): 2034-2039.

PURPOSE: A phase I study of patients with metastatic malignant melanoma (MM) and renal cell carcinoma (RCC) evaluated the safety and maximum tolerated dose (MTD), pharmacokinetics, pharmacodynamics, and preliminary antitumor activity of recombinant human interleukin-21 (rIL-21). PATIENTS AND METHODS: Patients who had one or fewer prior systemic treatments for metastatic MM or RCC were treated with rIL-21 administered for two 5-day cycles on days 1 through 5 and 15 through 19 of a treatment course; rIL-21 was administered by rapid intravenous infusion in an outpatient setting. Cohorts of patients received doses ranging from 3 to 100 microg/kg/dose, and an expanded cohort was treated at the MTD. Patients with stable disease (SD) or better could receive additional treatment cycles. RESULTS: Forty-three patients were treated (24 MM; 19 RCC), including 28 in the expanded cohort. Dose-limiting toxicities consisted primarily of transient grade 3 laboratory abnormalities. The MTD was estimated to be 30 microg/kg. The most common adverse events included flu-like symptoms, pruritus, and rash. Twelve patients received up to five additional two-cycle courses of treatment without cumulative toxicity, except for one patient with reversible grade 4 hepatotoxicity. Serum concentrations of rIL-21 increased in a dose-proportional manner. Dose-dependent increases in soluble CD25 reflected lymphocyte activation. Antitumor activity was observed in both MM (one complete response and 11 SD) and RCC (four partial responses, 13 SD). CONCLUSION: Outpatient therapy with rIL-21 at 30 microg/kg was well tolerated, had dose-dependent pharmacokinetics and pharmacodynamics, and was associated with antitumor activity in patients with MM and RCC.

103 [100]. Tykodi, S. S., E. H. Warren, et al. (2004). "Allogeneic hematopoietic cell transplantation for metastatic renal cell carcinoma after nonmyeloablative conditioning: toxicity, clinical response, and immunological response to minor histocompatibility antigens." Clin Cancer Res **10**(23): 7799-7811.

PURPOSE: This phase I trial assessed the safety, efficacy, and immunologic responses to minor histocompatibility antigens following nonmyeloablative allogeneic hematopoietic cell transplantation as treatment for metastatic renal cell carcinoma. EXPERIMENTAL DESIGN: Eight patients received conditioning with fludarabine and low-dose total body irradiation followed by hematopoietic cell transplantation from an HLA-matched sibling donor. Cyclosporine and mycophenolate mofetil were administered as posttransplant immunosuppression. Patients were monitored for donor engraftment of myeloid and lymphoid cells, for clinical response by serial imaging, and for immunologic response by in vitro isolation of donor-derived CD8(+) CTLs recognizing recipient minor histocompatibility (H) antigens. RESULTS: All patients achieved initial mixed hematopoietic chimerism with two patients rejecting their graft and recovering host hematopoiesis. Four patients developed acute, grade 2 to 3, graft-versus-host disease and four patients developed extensive chronic graft-versus-host disease. Five patients had progressive disease, two patients had stable disease, and one patient experienced a partial response after receiving donor lymphocyte infusions and IFN-alpha. CD8(+) CTL clones recognizing minor H antigens were isolated from five patients studied. Clones from three patients with a partial response or stable disease recognized antigens expressed on renal cell carcinoma tumor cells. CONCLUSIONS: Treatment of metastatic renal cell carcinoma with allogeneic hematopoietic cell transplantation after nonmyeloablative conditioning with fludarabine/total body irradiation is feasible and may induce tumor regression or stabilization in some patients. CD8(+) CTL-recognizing minor H antigens on tumor cells can be isolated posttransplant and could contribute to the graft-versus-tumor effect. Such antigens may represent therapeutic targets for posttransplant vaccination or adoptive T-cell therapy to augment the antitumor effects of allogeneic hematopoietic cell transplantation.

104 [55]. Van Gool, A. R., R. Verkerk, et al. (2008). "Plasma activity of prolyl endopeptidase in relation to psychopathology during immunotherapy with IFN-alpha in patients with renal cell carcinoma." J Interferon Cytokine Res **28**(5): 283-286.

Abnormal activity in peripheral blood of the cytosolic enzyme prolyl endopeptidase (PEP, EC 3.4.21.26, post prolyl cleaving enzyme, prolyl oligopeptidase) has been found in patients with a variety of psychiatric disorders, most consistently in mood disorders. Mood disturbance is a well-known side effect of immunotherapy with interferon-alpha (IFN-alpha). Earlier, we documented a decrease in serum PEP activity in the first 4 weeks of treatment with IFN-alpha. In 24 patients (16 men, 8 women, median age 60.5 years, range 47-72 years) with metastatic renal cell carcinoma (RCC), psychiatric assessment and blood sampling were performed before and at 4 and 8 weeks and at 6 months after initiation of treatment with IFN-alpha. No episodes of depression were observed, and the sum score and the scores on the subscales for depression and hostility of the Symptom Check List-90 (SCL-90) did not change during follow-up, whereas the anxiety scores were somewhat lower at 4 and 8 weeks compared with baseline. No change in plasma PEP activity and no relationships between change in psychiatric parameters and change in plasma PEP activity were found. As more subtle relationships between PEP activity and psychiatric status could have easily been obscured, a role for PEP in the pathophysiology of IFN-alpha-induced mood disturbance can neither be confirmed nor excluded.

105 [88]. Verra, N., D. de Jong, et al. (2005). "Infiltration of activated dendritic cells and T cells in renal cell carcinoma following combined cytokine immunotherapy." Eur Urol **48**(3): 527-533.

OBJECTIVES: In a phase I study the feasibility, toxicity and immunological effects of peri-operative cytokine immunotherapy of renal cell carcinoma were studied. Main goals were to determine the maximal tolerable dose and detailed in situ analysis of tumor infiltrates. METHODS: Fifteen patients with renal cell carcinoma, undergoing nephrectomy, received subcutaneous immunotherapy, consisting of low-dose IL-2, IFNalpha and GM-CSF, from day -3 prior, until day +5 following surgery in a dose escalation study. Infiltrates from resected tumor tissues from patients undergoing immunotherapy or control patients that underwent nephrectomy only, were examined using quantitative immunohistological analysis and 3-color immunofluorescence staining and confocal laser scanning microscope analysis. RESULTS: Toxicity was limited and the maximal tolerable dose was established. In peripheral blood an increase was found in total lymphocytes, (activated) T cells, NK cells and monocytes. Quantitative immunohistological analysis of tumor infiltrates showed enhanced numbers of CD3+ T cells, S100+ DC, CD83+ DC and IL-2 receptor positive cells (4-fold, 2-fold, 10-fold and 20-fold, respectively, compared to controls). In treated patients preferential invasion was observed of TNFalpha positive CD8+ T cells and DC, positive for DC-SIGN (CD209), CD83, CD80, IL-12 and the DC specific chemokine, DC-CK1 (CCL18). CONCLUSIONS: These findings show increased infiltration of activated, mature DC and functionally active CD8+ T cells in renal tumors, which may suggest clinical potential of cytokine immunotherapy.

106 [13]. Walter, S., T. Weinschenk, et al. (2012). "Multipeptide immune response to cancer vaccine IMA901 after single-dose cyclophosphamide associates with longer patient survival." Nat Med **18**(8): 1254-1261.

IMA901 is the first therapeutic vaccine for renal cell cancer (RCC) consisting of multiple tumor-associated peptides (TUMAPs) confirmed to be naturally presented in human cancer tissue. We treated a total of 96 human leukocyte antigen A (HLA-A)*02(+) subjects with advanced RCC with IMA901 in two consecutive studies. In the phase 1 study, the T cell responses of the patients to multiple TUMAPs were associated with better disease control and lower numbers of prevaccine forkhead box P3 (FOXP3)(+) regulatory T (T(reg)) cells. The randomized phase 2 trial showed that a single dose of cyclophosphamide reduced the number of T(reg) cells and confirmed that immune responses to multiple TUMAPs were associated with longer overall survival. Furthermore, among six predefined populations of myeloid-derived suppressor cells, two were prognostic for overall survival, and among over 300 serum biomarkers, we identified apolipoprotein A-I (APOA1) and chemokine (C-C motif) ligand 17 (CCL17) as being predictive for both immune response to IMA901 and overall survival. A randomized phase 3 study to determine the clinical benefit of treatment with IMA901 is ongoing.

107 [111]. Wang, H. J., H. Wang, et al. (2004). "[Immunotherapy of metastatic renal cell carcinoma: report of 28 cases]." Zhonghua Wai Ke Za Zhi **42**(4): 205-206.

OBJECTIVE: To determine the efficacy of IL-2, IFN and Furtulon in patients with metastatic renal cell carcinoma. METHODS: During the induction phase of the treatment of 28 patients, which lasted 3 months, IL-2 and IFN were administered subcutaneously three times a week at doses of 5 - 20 MU/m(2) and 6 - 9 MU/m(2), Furtulon was administered at doses of 800 - 1,200 mg daily by oral during 28 days a month. RESULTS: The response rate was 46.4%, including 4 complete response (CR), 9 presented with partial response (PR). CONCLUSION: The three-drugs combination described in this study demonstrates activity. Based on the present data, combined biochemotherapy may be a promising new approach to the therapy of the metastatic renal cell carcinoma.

108 [92]. Wierecky, J., M. Mueller, et al. (2006). "Dendritic cell-based cancer immunotherapy targeting MUC-1." Cancer Immunol Immunother **55**(1): 63-67.

Vaccination therapy using dendritic cells (DC) as antigen presenting cells (APC) has shown significant promise in laboratory and animal studies as a potential treatment for malignant diseases. Pulsing of autologous DCs with tumor-associated antigens (TAA) is a method often used for antigen delivery and choice of suitable antigens plays an important role in designing an effective vaccine. We identified two HLA-A2 binding novel 9-mer peptides of the TAA MUC1, which is overexpressed on various hematological and epithelial malignancies. Cytotoxic T cells generated after pulsing DC with these peptides were able to induce lysis of tumor cells expressing MUC1 in an antigen-specific and HLA-restricted fashion. Within two clinical studies, we demonstrated that vaccination of patients with advanced cancer using DCs pulsed with MUC1 derived peptides is well tolerated without serious side effects and can induce immunological responses. Of 20 patients with metastatic renal cell carcinoma, 6 patients showed regression of metastases with 3 objective responses (1 CR, 2 PR). Furthermore, we found that in patients responding to treatment T cell responses for antigens not used for treatment occurred suggesting that antigen spreading in vivo might be a possible mechanism of mediating antitumor effects. These results demonstrate that immunotherapy in patients with advanced malignancies using autologous DCs pulsed with MUC1 derived peptides can induce immunological and clinical responses. However, further clinical studies are needed to identify the most potent treatment regimen that can consistently mediate an antitumor immune response in vivo.

109 [81]. Wierecky, J., M. R. Muller, et al. (2006). "Immunologic and clinical responses after vaccinations with peptide-pulsed dendritic cells in metastatic renal cancer patients." Cancer Res **66**(11): 5910-5918.

A phase I trial was conducted to evaluate the feasibility, safety, and efficacy of a dendritic cell-based vaccination in patients with metastatic renal cell carcinoma (RCC). Autologous mature dendritic cells derived from peripheral blood monocytes were pulsed with the HLA-A2-binding MUC1 peptides (M1.1 and M1.2). For the activation of CD4(+) T-helper lymphocytes, dendritic cells were further incubated with the PAN-DR-binding peptide PADRE. Dendritic cell vaccinations were done s.c. every 2 weeks for four times and repeated monthly until tumor progression. After five dendritic cell injections, patients additionally received three injections weekly of low-dose interleukin-2 (1 million IE/m(2)). The induction of vaccine-induced T-cell responses was monitored using enzyme-linked immunospot and Cr release assays. Twenty patients were included. The treatment was well tolerated with no severe side effects. In six patients, regression of the metastatic sites was induced after vaccinations with three patients achieving an objective response (one complete response, two partial responses, two mixed responses, and one stable disease). Additional four patients were stable during the treatment for up to 14 months. MUC1 peptide-specific T-cell responses in vivo were detected in the peripheral blood mononuclear cells of the six patients with objective responses. Interestingly, in patients responding to the treatment, T-cell responses to antigens not used for vaccinations, such as adipophilin, telomerase, or oncofetal antigen, could be detected, indicating that epitope spreading might occur. This study shows that MUC1 peptide-pulsed dendritic cells can induce clinical and immunologic responses in patients with metastatic RCC.

110 [52]. Willems, E., F. Baron, et al. (2009). "Non-myeloablative transplantation with CD8-depleted or unmanipulated peripheral blood stem cells: a phase II randomized trial." Leukemia **23**(3): 608-610.

111 [71]. Yun, T., K. W. Lee, et al. (2007). "Non-myeloablative allogeneic stem cell transplantation for metastatic renal cell carcinoma." Clin Transplant **21**(3): 337-343.

Between 1999 and 2004, 11 patients with metastatic renal cell carcinoma (RCC) underwent non-myeloablative stem cell transplantation (NST) with conditioning using fludarabine-based regimens in two institutions of Korea. Among 11 patients, only one patient showed partial response (response rate: 9%), three showed stable disease, and six progressive disease. Three patients developed acute graft-versus-host disease (GVHD), and among them, one developed grade III acute GVHD which caused early death at day 60 after transplantation, and this patient showed partial response at day 30. Six patients developed chronic GVHD, three limited, and three extensive GVHD, respectively. Survival after one yr was 18% in transplanted patients. Median overall survival for entire cohort was 4.3 months. Eight patients died from progressive disease and three (27%) from treatment-related mortality. Only one patient survived 51.2 months after NST with slowly progressive disease. This patient received donor lymphocyte infusion three times after NST and achieved complete donor chimerism. NST does not lead to durable response and prolonged overall survival in the majority of patients with RCC in our series.

112 [11]. Zhan, H. L., X. Gao, et al. (2012). "A randomized controlled trial of postoperative tumor lysate-pulsed dendritic cells and cytokine-induced killer cells immunotherapy in patients with localized and locally advanced renal cell carcinoma." Chin Med J (Engl) **125**(21): 3771-3777.

BACKGROUND: It remains a challenge to inhibit the local recurrence or distant metastasis of localized or locally advanced renal cell carcinoma (RCC) after surgical resection. We investigated the feasibility, safety and efficacy of immunotherapy using autologous tumor lysate (TL)-pulsed dendritic cells (DCs) and cytokine-induced killer (CIK) cells in patients with localized or locally advanced RCC. METHODS: From January 2001 to July 2009, we collected 137 patients that met the selection criteria and randomly divided them into three groups. After surgery, immunotherapy with TL-pulsed DCs-CIK cells (DC-CIK group) and interferon (IFN)-alpha (IFN-alpha group) was performed in 46 patients, respectively. The other 45 patients received no postoperative adjuvant therapy (the control group). The changes in the numbers of T lymphocyte subsets, including CD4(+)CD25(high) regulatory T cells (Treg), were determined before the operation and after immunotherapy. The overall survival was compared among the three groups. RESULTS: An increase of the CD4(+)/CD8(+) ratio and a decrease of CD4(+)CD25(high) cells were observed after TL-pulsed DC-CIK cells or IFN-a immunotherapy. All patients tolerated the TL-pulsed DC-CIK cells immunotherapy very well, and side effects in the DC-CIK group were less than in the IFN-alpha group. The metastasis and recurrence rates were significantly decreased after TL-pulsed DC-CIK cells or IFN-alpha immunotherapy compared with the control group (P < 0.01). The Log-rank test showed that the overall survival rates were significantly higher in the DC-CIK group and IFN-alpha group than that in the control group (P < 0.01), but there was no difference between the DC-CIK group and IFN-alpha group (P > 0.05). CONCLUSION: Postoperative immunotherapy with TL-pulsed DC-CIK cells may prevent recurrence/metastasis and increase the overall survival rate after surgery in localized or locally advanced RCC.

113 [1]. Zhang, Y., J. Wang, et al. (2013). "Autologous CIK cell immunotherapy in patients with renal cell carcinoma after radical nephrectomy." Clin Dev Immunol **2013**: 195691.

OBJECTIVE: To evaluate the efficacy of autologous cytokine-induced killer (CIK) cells in patients with renal cell carcinoma (RCC). METHODS: 20 patients diagnosed with TNM stage I or II RCC were randomly divided into two groups, a CIK cell treatment group and a control group. The endpoint was progression-free survival (PFS) evaluated by Kaplan-Meier analyses. RESULTS: CD3(+), CD3(+)/CD8(+), CD3(+)/CD4(+), and CD3(+)/CD56(+) levels increased after CIK cell culture (P < 0.01). The median PFS in CIK cell treatment group was significantly longer than that in control group (PFS, 32.2 months versus 21.6 months; log-rank, P = 0.032), all patients were alive during the course of followup, and there are no statistically significant differences between two groups in OS (log-rank, P = 0.214). Grade III or greater adverse events were not observed. CONCLUSIONS: CIK cells treatment could prolong survival in patients with RCC after radical nephrectomy and showed acceptable curative effect with potential enhancement of cellular immune function. This trial is registered with Clinicaltrials.gov NCT01799083.

114 [8]. Zudaire Bergera, J. J., A. Rincon Mayans, et al. (2013). "[Renal cell carcinoma molecular biology. Prognostic and therapeutic usefulness]." Arch Esp Urol **66**(1): 23-32.

Renal cell adenocarcinoma requires different therapeutic pathways because it is one of the most therapy-resistant tumors, on the other hand it is biologically one of the most attractive tumors. Its pathological classification has a genetic base. There is an anomaly of the Von Hippel Lindau gene in 80% of adenocarcinomas, being this fact determinant to know the biological characteristics of tumor initiation and development, as well as the identification of factors susceptible to be used as therapeutic targets. Since 2005 a group of molecules have been used in the treatment of metastatic adenocarcinomas and, even though therapeutic results are significant but not clinically relevant yet, we are sure they are a key way for more efficient future developments. The present study tries to make a tour on the research of the biological anomalies in renal adenocarcinoma with special emphasis in the Von HippelLindau gene.

115 [79]. Zustovich, F., G. Cartei, et al. (2006). "A phase II study of gemcitabine and immunotherapy in renal cancer: preliminary results and review of the literature." Ann Oncol **17 Suppl 5**: v133-136.

116 [60]. Zustovich, F., G. Cartei, et al. (2007). "A phase II study of gemcitabine at fixed infusion rate of 10 mg/m2/min with or without immunotherapy in advanced renal cancer." Anticancer Res **27**(6C): 4461-4464.

BACKGROUND: Advanced renal cancer remains a challenge for oncologists since no treatment other than surgery has demonstrated a clear survival advantage. PATIENTS AND METHODS: Gemcitabine was given to suitable patients at a fixed infusion rate of 10 mg/m2/min. Eighteen patients received concomitant immunotherapy, mostly low doses of interleukin 2 (IL2). RESULTS: Thirty patients were enrolled. The overall response rate was 14% (22% in the subset of patients treated with both chemotherapy and immunotherapy) with a median progression-free survival time of 4.1 + months. Toxicity was not mild, mostly fatigue, nausea and anaemia, even though not life threatening. CONCLUSION: Gemcitabine at the fixed infusion rate of 10 mg/m2/min with concomitant low doses of IL2 could be useful in the palliative treatment of symptomatic patients with renal carcinoma progressing after tyrosine kinases inhibitor.

# TOPIC: [Kidney Cancer or Renal Cancer] and Interferon

1 [244]. Aass, N., P. H. De Mulder, et al. (2005). "Randomized phase II/III trial of interferon Alfa-2a with and without 13-cis-retinoic acid in patients with progressive metastatic renal cell Carcinoma: the European Organisation for Research and Treatment of Cancer Genito-Urinary Tract Cancer Group (EORTC 30951)." J Clin Oncol **23**(18): 4172-4178.

PURPOSE: A randomized phase II/III trial was conducted to determine whether combination treatment with 13-cis-retinoic acid (13-CRA) plus interferon alfa-2a (IFN-alpha-2a) was superior to IFN-alpha-2a alone in patients with progressive metastatic renal cell carcinoma. PATIENTS AND METHODS: Three hundred twenty patients were randomly assigned to treatment with IFN-alpha-2a plus 13-CRA or to IFN-alpha-2a alone. IFN-alpha-2a was given daily subcutaneously, starting at a dose of 3 million units (MU). The dose was escalated every 7 days from 3 to 9 MU by increments of 3 MU. Patients randomly assigned to combination therapy received oral 13-CRA 1 mg/kg/d plus IFN-alpha-2a. RESULTS: Median time to progression was 5.1 months for patients treated with the combination and 3.4 months for patients on IFN-alpha-2a alone (P = .008). Progression-free survival rates at 6 months were 43% for patients receiving combined therapy and 30% for patients on IFN-alpha-2a, and at 12 months, 27% and 17%, respectively. Median overall survival was 17.3 months for patients on IFN-alpha-2a and 13-CRA, and 13.2 months for patients treated with IFN-alpha-2a (P = .048). Twenty-two percent of the patients receiving the combination stopped treatment due to toxicity, as compared with 16% on IFN-alpha-2a. CONCLUSION: Progression-free and overall survival for patients with progressive metastatic renal cell carcinoma treated with IFN-alpha-2a plus 13-CRA were significantly longer compared with patients on IFN-alpha-2a alone (P = .007 and P = .048, respectively). Improvement in efficacy in the combination arm was accompanied by increased, though not serious, toxicity.

2 [230]. Adamson, P. C., K. K. Matthay, et al. (2007). "A phase 2 trial of all-trans-retinoic acid in combination with interferon-alpha2a in children with recurrent neuroblastoma or Wilms tumor: A Pediatric Oncology Branch, NCI and Children's Oncology Group Study." Pediatr Blood Cancer **49**(5): 661-665.

BACKGROUND: The combination of the antiproliferative and differentiation-inducing effects of retinoids together with the antiproliferative, immunostimulatory, and differentiation-potentiating effects of interferon-alpha (IFN-alpha) were the basis for the development of this combination in pediatric patients with refractory neuroblastoma or Wilms tumor. PROCEDURE: A phase 2 trial of all-trans-retinoic acid (ATRA), administered orally at a dose of 90 mg/m(2)/day in three divided doses for 3 consecutive days per week, and IFN-alpha2a, administered subcutaneously daily at a dose of 3 x 10(6) U/m(2)/day for 5 consecutive days per week, in 4 week cycles was performed. A two-stage design was used for each disease stratum. RESULTS: Seventeen patients (16 evaluable) with neuroblastoma, median age 9 years, and 15 patients (14 evaluable) with Wilms tumor, median age 6 years, were enrolled. Overall, the combination was well tolerated, with headache being the most common toxicity observed. There were no complete or partial responses. The median number of cycles administered was 1 (range 1-9). Four patients with neuroblastoma had stable disease for 12 or more weeks. CONCLUSIONS: The combination of ATRA and IFN-alpha2a was inactive in children with relapsed or refractory neuroblastoma and Wilms tumor. The lack of activity with this combination in children with refractory neuroblastoma is similar to the disappointing phase 2 results of single agent 13-cis-retinoic-acid (13cRA) and does not support further development of ATRA for children with relapsed neuroblastoma.

3 [122]. Aitchison, M., C. A. Bray, et al. (2014). "Adjuvant 5-flurouracil, alpha-interferon and interleukin-2 versus observation in patients at high risk of recurrence after nephrectomy for renal cell carcinoma: results of a phase III randomised European Organisation for Research and Treatment of Cancer (Genito-Urinary Cancers Group)/National Cancer Research Institute trial." Eur J Cancer **50**(1): 70-77.

BACKGROUND: The purpose of this trial was to compare adjuvant 5-flurouracil, alpha-interferon and interleukin-2 to observation in patients at high risk of recurrence after nephrectomy for renal cell carcinoma (RCC) in terms of disease free survival, overall survival and quality of life (QoL). PATIENTS AND METHODS: Patients 8weeks post nephrectomy for RCC, without macroscopic residual disease, with stage T3b-c,T4 or any pT and pN1 or pN2 or positive microscopic margins or microscopic vascular invasion, and no metastases were randomised to receive adjuvant treatment or observation. QoL was assessed by European Organisation for Research and Treatment of Cancer (EORTC) Quality of Life Questionnaire-30 (QLQC-30). Treatment delivery and toxicity were monitored. The trial was designed to detect an increase in 3year disease free survival (DFS) from 50% on observation to 65% on treatment (hazard ratio (HR)=0.63) with 90% power and two-sided alpha=0.05. RESULTS: From 1998 to 2007, 309 patients were randomised (155 to observation; 154 to treatment). 35% did not complete the treatment, primarily due to toxicity (92% of patients experienced grade 2, 41% grade 3). Statistically significant differences between the arms in QoL parameters at 2months disappeared by 6months although there was suggestion of a persistent deficit in fatigue and physical function. Median follow-up was 7years (maximum 12.1years). 182 patients relapsed or died. DFS at 3years was 50% with observation and 61% with treatment (HR 0.84, 95% confidence interval (CI) 0.63-1.12, p=0.233). 124 patients died. Overall survival (OS) at 5years was 63% with observation and 70% with treatment (HR 0.87, 95% CI 0.61-1.23, p=0.428). CONCLUSIONS: The treatment is associated with significant toxicity. There is no statistically significant benefit for the regimen in terms of disease free or overall survival.

4 [160]. Akaza, H., K. Kawai, et al. (2010). "Successful outcomes using combination therapy of interleukin-2 and interferon-alpha for renal cell carcinoma patients with lung metastasis." Jpn J Clin Oncol **40**(7): 684-689.

OBJECTIVE: In our previous study, a combination therapy of interleukin-2 and interferon-alpha was found to be more effective than monotherapy, especially for lung metastasis. In order to determine the genetic markers of those who positively responded, a multi-institutional open study was conducted on the patients with lung metastasis. In this paper, the clinical response to our combination therapy is reported. METHODS: Untreated patients with lung metastasis were enrolled in this study. Patients received interleukin-2 (0.7 x 10(6) U/day) and interferon-alpha (6 x 10(6) IU/day): interleukin-2, 5 days a week and interferon-alpha, 3 days a week for the first 8 weeks, and then both interleukin-2 and interferon-alpha, 2 or 3 days a week for 16 additional weeks. RESULTS: Forty-two patients were able to be evaluated for response. The overall positive response rate was 35.7% (15 of 42) including 2 patients with complete response. Progression-free patients were observed more frequently in patients with lung metastasis only (80.6%) than those with lung plus other organ metastasis (54.5%). Tumor shrinkage was observed in 81.0% (34 of 42) of patients. Progression-free survival rate at 200 days was 63.6%. Toxicities observed were primarily flu-like symptoms due to the cytokines and were typical of those observed with each single agent. CONCLUSIONS: Combination therapy of interleukin-2 and interferon-alpha was confirmed to be effective for renal cell carcinoma patients with lung metastasis. Identification of genetic markers is now ongoing with the tissue samples from this trial.

5 [144]. Akaza, H., T. Tsukamoto, et al. (2011). "Combined immunotherapy with low-dose IL-2 plus IFN-alpha for metastatic renal cell carcinoma: survival benefit for selected patients with lung metastasis and serum sodium level." Jpn J Clin Oncol **41**(8): 1023-1030.

OBJECTIVE: To clarify the survival benefit of immunotherapy for renal cell carcinoma patients with lung metastasis using low-dose interleukin-2 plus interferon-alpha, we examined survival outcomes and factors associated with prognosis. METHODS: This was a multicenter prospective study. Nephrectomized renal cell carcinoma patients with lung metastasis were treated with interleukin-2 (0.7 x 10(6) unit, 5 days a week) and interferon-alpha (6 x 10(6) IU, 3 days a week) for the first 8 weeks, and then with both interleukin-2 and interferon-alpha, 2 or 3 days a week for 16 additional weeks. RESULTS: Median follow-up period for 42 patients was 28.3 months (range: 4.2-43.8). Two-year overall survival rate was 82% and the probability of 3 year survival rate was 71%. Median progression-free survival was 10.4 months. While no difference was found in survival among patients assessed as complete response, partial response and no change, survival of patients assessed as NC or better was significantly better than those assessed as progressive disease (P < 0.0001). Furthermore, multivariate analyses identified pre-treatment serum sodium (P = 0.004) as an independent prognostic factor. The sodium level was also statistically associated with tumor response (p = 0.035). Patients with normal sodium level survived significantly longer (P = 0.0005) than those with low sodium level showing median survival of 12.2 months. CONCLUSIONS: Combination immunotherapy with low-dose interleukin-2 plus interferon-alpha showed survival benefit for patients with lung metastasis whose tumor responded as no change or better. This combination immunotherapy could be beneficial for patients selected by metastatic organ and their pre-treatment serum sodium level.

6 [226]. Akaza, H., T. Tsukamoto, et al. (2006). "A low-dose combination therapy of interleukin-2 and interferon-alpha is effective for lung metastasis of renal cell carcinoma: a multicenter open study." Int J Clin Oncol **11**(6): 434-440.

BACKGROUND: To confirm the usefulness of a combination therapy of interleukin-2 (IL-2) and interferon-alpha (IFN-alpha) against metastatic renal cell carcinoma, the recommended dose of IFN-alpha to use in combination with low-dose IL-2 was determined (phase 1). Efficacy and safety at this dose was evaluated (phase 2). METHODS: In phase 1, the dose of IL-2 was fixed at 0.7 x 10(6) Japan reference unit (JRU)/person for 5 days a week. The dose of IFN-alpha was increased from 3 x 10(6) IU for 3 days a week (level I) to 6 x 10(6) IU for 3 days a week (level II) and to 6 x 10(6) IU for 5 days a week (level III). RESULTS: In phase 1, 10 patients were registered, with 9 (3 at each level) able to be evaluated. Because grade 3 and grade 4 neutropenia were observed at level III in 1 patient each, level II was found to be the recommended regimen. The response rate in phase 1 was 44.4% (4/9). In phase 2, 46 patients were registered, with a response rate in 37 patients, classified as per protocol set (PPS), of 21.6% (8/37). Toxicities observed were primarily flu-like symptoms due to cytokines, and gastrointestinal symptoms. Leukocyte abnormalities were observed, but they were milder and tolerable. CONCLUSION: In the 46 patients evaluated in phase 1 and phase 2, the response rate was 26.1% (12/46), being highest in 38.7% (12/31) of those who were nephrectomized, and with only lung metastases.

7 [256]. Alatrash, G., T. E. Hutson, et al. (2004). "Clinical and immunologic effects of subcutaneously administered interleukin-12 and interferon alfa-2b: phase I trial of patients with metastatic renal cell carcinoma or malignant melanoma." J Clin Oncol **22**(14): 2891-2900.

PURPOSE: Interleukin-12 (IL-12) and interferon alfa-2b (IFN-alpha-2b) are pleiotropic cytokines with activity in renal cell carcinoma (RCC) and malignant melanoma (MM) as single agents. Preclinical studies suggest concurrent administration may have synergistic antitumor effects. We conducted a phase I trial of concurrent subcutaneous (SC) administration of IL-12 and IFN-alpha-2b in patients with metastatic RCC or MM to determine toxicity, maximum-tolerated dose, preliminary efficacy, and effects on chemokine/cytokine gene expression in peripheral blood mononuclear cells (PBMCs). PATIENTS AND METHODS: Cohorts of three to six patients were treated with escalating doses of IL-12 (dose I, 100 ng/kg; dose II, 300 ng/kg; dose III, 500 ng/kg; dose IV, 500 ng/kg SC) given twice weekly and IFN-alpha-2b (dose I, 1.0 MU/m(2); dose II, 1.0 MU/m(2); dose III, 1.0 MU/m(2); dose IV, 3.0 MU/m(2) SC) three times weekly in 4-week cycles. Effects on gene expression were assessed by reverse transcriptase polymerase chain reaction. RESULTS: Twenty-six patients (19 with RCC, seven with MM) were accrued at dose levels I (n = 3), II (n = 3), III (n = 13), and IV (n = 7). Dose-limiting toxicity included grades 3 and 4 hepatotoxicity and neutropenia/leukopenia. Patients received a median of three cycles of treatment. Two patients with RCC and one patient with MM had partial responses. Median survival was 13.8 months. Reverse transcriptase polymerase chain reaction on PBMCs revealed induction of IP-10, Mig, B7.1 (CD80), interleukin-5, and interferon gamma in selected patients. CONCLUSION: Concurrent SC administration of IL-12 and IFN-alpha-2b is possible at the dose levels utilized. Recommended doses for phase II trials are 500 ng/kg IL-12 and 1.0 MU/m(2) IFN-alpha-2b. Consistent induction of IP-10 and Mig, as well as variable induction of B7.1, interleukin-5, and interferon gamma expression was noted in PBMCs.

8 [196]. Amato, R. J., J. Jac, et al. (2008). "Interferon-alpha in combination with either imatinib (Gleevec) or gefitinib (Iressa) in metastatic renal cell carcinoma: a phase II trial." Anticancer Drugs **19**(5): 527-533.

Treatments for metastatic renal cell carcinoma (MRCC) are limited. RCCs frequently overexpress epithelial growth factor receptor and express c-Kit and platelet-derived growth factor receptor-beta. Combination of interferon with tyrosine kinase inhibitors of epithelial growth factor receptor [gefitinib (Iressa)] or c-Kit and platelet-derived growth factor receptor-beta [imatinib (Gleevec)] was evaluated for efficacy and safety. Patients with MRCC received 12-week cycles of interferon [3 million units (MU) subcutaneously thrice in week 1 and 6 MU thrice weekly thereafter] and either gefitinib (500 mg daily) or imatinib (600 mg daily). The gefitinib/imatinib dose was reduced as needed owing to toxicity. The primary endpoint was objective tumor response. Secondary endpoints were time to tumor progression, overall survival, and safety. Seventeen patients were enrolled. Most had clear cell [36% (6/17)] or papillary [36% (6/17)] tumors. Most (n=14) were treated on the gefitinib arm, including two patients who crossed over from the imatinib arm after experiencing disease progression. Objective tumor responses were evaluable in 14 patients (82%). Of these 14, partial responses occurred in three (21%), stable disease in seven (50%), and progressive disease in four (29%). The most frequent treatment-related adverse events were skin rash, flu-like symptoms, and fatigue (both treatment arms); diarrhea (gefitinib arm only); and thrombocytopenia and leukopenia (imatinib arm only). Median time to tumor progression (range) for patients on the gefitinib arm only was 4.27 (1.13-15.97) months and median overall survival (range) was 11.42+ (1.13-29.07+) months. Combination of gefitinib with interferon safely delays progression of refractory MRCC. Further studies in this setting are warranted.

9 [215]. Amato, R. J. and M. Khan (2008). "A phase I clinical trial of low-dose interferon-alpha-2A, thalidomide plus gemcitabine and capecitabine for patients with progressive metastatic renal cell carcinoma." Cancer Chemother Pharmacol **61**(6): 1069-1073.

BACKGROUND: We have conducted a phase I trial to determine the maximum tolerated dose of gemcitabine in combination with interferon, thalidomide and capecitabine. METHODS: Patients received oral capecitabine 1,000 mg/m(2 )per day, divided in 2 daily doses, 2 weeks on, 1 week off; subcutaneous interferon-alpha 1 mIU twice a day without an interruption; daily oral thalidomide 200 mg/day for the first 7 days, then escalated to 400 mg/day without an interruption. Gemcitabine was given by intravenous administration over 30 min on day 1, week 1 and day 8, week 2. Initial dose level of gemcitabine was 400 mg/m(2). The dose of gemcitabine was the phase I variable. One cycle was 3 weeks. RESULTS AND DISCUSSION: We treated 12 patients, 6 patients were entered at a dose level of 0 (gemcitabine 400 mg/m(2)) and 6 patients entered at a dose level-1 (gemcitabine 200 mg/m(2)). Eight of 12 patients completed at least 12 weeks of therapy. Three partial responses and two stable disease were observed. The remaining patients had progressive disease. Non-hematologic toxicity was either grade 1 or 2. Hematologic toxicity at dose level 0 consisted of 3 patients with grade 3/4 neutropenia, and 1 patient with grade 3 thrombocytopenia. At dose level-1 grade 1/2 neutropenia was observed. CONCLUSIONS: The completion of our phase I experience determined our maximum tolerated dose to be dose level-1. The phase II trial is currently being proposed for patients with rapidly growing clear cell, other histologies that may contain sarcomatoid elements or collecting duct tumor.

10 [195]. Amato, R. J. and T. Mohammad (2008). "Interferon-alpha plus capecitabine and thalidomide in patients with metastatic renal cell cancer." J Exp Ther Oncol **7**(1): 41-47.

Thirty patients with progressive metastatic renal cell carcinoma were treated. Eleven patients received 2 or more prior systemic therapies; 2 had a complete response, 7 had a partial response, and 11 had stable disease. The complete responders are off therapy and remain without disease recurrence. The median duration of response was 3.8 months (range 1 - 48+ months). Therapy was well tolerated; predominant toxicities were fatigue, paraesthesias and hand/foot syndrome. The data suggest that the combination of interferon-alpha, thalidomide and capecitabine has anti-tumor activity in previously treated patients with progressive metastatic renal cell carcinoma. A prospective investigation of this combination warranted.

11 [243]. Amato, R. J. and A. Rawat (2006). "Interferon-alpha plus capecitabine and thalidomide in patients with metastatic renal cell carcinoma: a pilot study." Invest New Drugs **24**(3): 171-175.

PURPOSE: To assess the activity and toxicity of interferon-alpha (IFN-alpha), capecitabine, and thalidomide in patients with metastatic renal cell carcinoma (MRCC). PATIENTS AND METHODS: Twenty-seven patients were enrolled in a pilot study to receive oral capecitabine 1,900 mg/m2/day in 2 daily doses, 2 weeks on, l week off; daily subcutaneous IFN-alpha 1 mIU without interruption; and daily oral thalidomide 200 mg/day for the first seven days, then escalated to 400 mg/day without interruption. Dosages were reduced for toxicity as necessary. RESULTS: Two patients discontinued treatment during the first week of the study, leaving 25 patients evaluable. There were 5 (20%) partial responses (PRs), 1 (4%) minor response (MR), 6 (24%) cases of stable disease (SD) > or = 6 months, and 13 (52%) cases of progressive disease (PD). The interval from first response to disease progression varied from 0-23 months: 17 patients progressed in 0-6 months; 4 progressed in 7-12 months; and 4 progressed in 12-24 months. Median survival was > 22 months, 14 months, and 1 month, respectively, for patients with PR, SD, and PD. Grade 3/4 toxicities consisted of hand-foot syndrome, neuropathy, fatigue, anemia, and deep venous thrombosis were common. CONCLUSION: This study demonstrates antitumor activity of combination IFN-alpha/capecitabine/thalidomide in MRCC. The 20% PR rate was notable, as the patient population had advanced disease and inferior performance status. Treatment was generally well tolerated, and further research is warranted to explore the efficacy of this combination for treating MRCC.

12 [131]. Armstrong, A. J., D. J. George, et al. (2012). "Serum lactate dehydrogenase predicts for overall survival benefit in patients with metastatic renal cell carcinoma treated with inhibition of mammalian target of rapamycin." J Clin Oncol **30**(27): 3402-3407.

PURPOSE: Lactate dehydrogenase (LDH) is an enzyme involved in anaerobic glycolysis and regulated by the phosphatidylinositol 3-kinase/Akt/mammalian target of rapamycin (mTOR)-containing complex 1 (PI3K/Akt/TORC1) pathway as well as tumor hypoxia/necrosis. High serum LDH levels are associated with poor prognosis in patients with cancer, including renal cell carcinoma (RCC). We tested whether serum LDH is prognostic and has predictive value in patients with metastatic RCC receiving an mTOR inhibitor. PATIENTS AND METHODS: We evaluated pretreatment and post-treatment serum LDH in 404 poor-risk patients with RCC treated with the TORC1 inhibitor temsirolimus or interferon alfa in an international phase III randomized trial. The proportional hazards model was used to test for the prognostic and predictive association of LDH in predicting overall survival (OS). RESULTS: Mean baseline serum normalized LDH was 1.23 times the upper limit of normal (ULN; range, 0.05 to 28.5 x ULN). The multivariable hazard ratio for death was 2.81 (95% CI, 2.01 to 3.94; P < .001) for patients with LDH more than 1 x ULN versus patients with LDH </= 1 x ULN. The LDH-treatment interaction term was statistically significant for OS (P = .016). Among 140 patients with LDH above the ULN, OS was significantly improved with temsirolimus (6.9 v 4.2 months; P < .002). Among 264 patients with normal LDH, OS was not significantly improved with temsirolimus as compared with interferon therapy (11.7 v 10.4 months; P = .514). CONCLUSION: Serum LDH is a prognostic and a predictive biomarker for the survival benefit conferred by TORC1 inhibition in poor-risk RCC. Further investigation of the predictive role of LDH as a measure of benefit with PI3K/TORC1 pathway inhibition in other RCC risk groups and other tumor types is warranted.

13 [229]. Atzpodien, J., H. Kirchner, et al. (2006). "Interleukin-2/interferon-alpha2a/13-retinoic acid-based chemoimmunotherapy in advanced renal cell carcinoma: results of a prospectively randomised trial of the German Cooperative Renal Carcinoma Chemoimmunotherapy Group (DGCIN)." Br J Cancer **95**(4): 463-469.

We performed a prospectively randomised clinical trial to compare the efficacy of four subcutaneous interleukin-2-(sc-IL-2) and sc interferon-alpha2a (sc-IFN-alpha2a)-based outpatient regimens in 379 patients with progressive metastatic renal cell carcinoma. Patients with lung metastases, an erythrocyte sedimentation rate < or =70 mm h(-1) and neutrophil counts < or =6000 microl(-1) (group I) were randomised to arm A: sc-IL-2, sc-IFN-alpha2a, peroral 13-cis-retinoic acid (po-13cRA) (n=78), or arm B: arm A plus inhaled-IL-2 (n=65). All others (group II) were randomised to arm C: arm A plus intravenous 5-fluorouracil (iv-5-FU) (n=116), or arm D: arm A plus po-Capecitabine (n=120). Median overall survival (OS) was 22 months (arm A; 3-year OS: 29.7%) and 18 months (arm B; 3-year OS: 29.2%) in group I, and 18 months (arm C; 3-year OS: 25.7%) and 16 months (arm D; 3-year OS: 32.6%) in group II. There were no statistically significant differences in OS, progression-free survival, and objective response between arms A and B, and between arms C and D, respectively. Given the known therapeutic efficacy of sc-IL-2/sc-INF-alpha2a/po-13cRA-based outpatient chemoimmunotherapies, our results did not establish survival advantages in favour of po-Capecitabine vs iv-5-FU, and in favour of short-term inhaled-IL-2 in patients with advanced renal cell carcinoma receiving systemic cytokines.

14 [247]. Atzpodien, J., E. Schmitt, et al. (2005). "Adjuvant treatment with interleukin-2- and interferon-alpha2a-based chemoimmunotherapy in renal cell carcinoma post tumour nephrectomy: results of a prospectively randomised trial of the German Cooperative Renal Carcinoma Chemoimmunotherapy Group (DGCIN)." Br J Cancer **92**(5): 843-846.

We conducted a prospectively randomised clinical trial to investigate the role of adjuvant outpatient immunochemotherapy administered postoperatively in high-risk patients with renal cell carcinoma. In total, 203 renal carcinoma patients' status post radical tumour nephrectomy were stratified into three risk groups: patients with tumour extending into renal vein/vena cava or invading beyond Gerota's fascia (pT3b/c pN0 or pT4pN0), patients with locoregional lymph node infiltration (pN+), and patients after complete resection of tumour relapse or solitary metastasis (R0). Patients were randomised to undergo either (A) 8 weeks of outpatient subcutaneous interleukin-2 (sc-rIL-2), subcutaneous interferon-alpha2a (sc-rIFN-alpha2a), and intravenous 5-fluorouracil (iv-5-FU) according to the standard Atzpodien regimen (Atzpodien et al, 2004) or (B) observation. Two-, 5-, and 8-year survival rates were 81, 58, and 58% in the treatment arm, and 91, 76, and 66% in the observation arm (log rank P=0.0278), with a median follow-up of 4.3 years. Two, 5-, and 8-year relapse-free survival rates were calculated at 54, 42, and 39% in the treatment arm, and at 62, 49, and 49% in the observation arm (log rank P=0.2398). Stage-adapted subanalyses revealed no survival advantages of treatment over observation, as well. Our results established that there was no relapse-free survival benefit and the overall survival was inferior with an adjuvant 8-week-outpatient sc-rIL-2/sc-rIFN-alpha2a/iv-5-FU-based immunochemotherapy compared to observation in high-risk renal cell carcinoma patients following radical tumour nephrectomy.

15 [262]. Bacik, J., M. Mazumdar, et al. (2004). "The functional assessment of cancer therapy-BRM (FACT-BRM): a new tool for the assessment of quality of life in patients treated with biologic response modifiers." Qual Life Res **13**(1): 137-154.

PURPOSE: This paper reports on the development and validation of two biologic response modifier (BRM) subscales for use with the Functional Assessment of Cancer Therapy-General (FACT-G) quality of life (QOL) questionnaire. METHODS: Using the FACT-G as a base, 17 additional questions related to symptoms common to interferon and retinoid therapy were developed. Data collected at baseline (n = 191) and week 2 (n = 168) in a randomized trial of interferon +/- 13-cis-retinoic acid in advanced renal cell carcinoma patients were used to validate this measure. RESULTS: Using a combined empirical and conceptual approach, the 17 questions were reduced to 13 questions consisting of two subscales: 'BRM-physical' (7 items; baseline coefficient alpha(alpha) = 0.70; week-2 alpha = 0.75) and 'BRM-mental' (6 items; baseline alpha = 0.79; week-2 alpha = 0.78). Internal consistency of the trial outcome index (TOI) combining physical well-being, functional well-being and the BRM subscales, was 0.91 for baseline assessments and 0.92 for week 2. Discriminant validity was demonstrated for the TOI by its ability to differentiate among prognostic risk groups, and for the total FACT-G, TOI and total FACT-BRM scores by their ability to distinguish between groups differing in performance, response and toxicity status. CONCLUSIONS: The 'BRM-physical' and 'BRM-mental' subscales can be combined with the FACT-G to form the 'FACT BRM' scale, useful for measuring QOL in cancer patients who are receiving treatment with biologic response modifiers.

16 [201]. Bannink, M., W. H. Kruit, et al. (2008). "Interferon-alpha in oncology patients: fewer psychiatric side effects than anticipated." Psychosomatics **49**(1): 56-63.

Interferon-alpha (IFN-alpha) treatment in both oncological and hepatological settings is associated with depression. If IFN-alpha treatment induces depression in high numbers, it could serve as a model for studying the pathophysiology of depression, in general. The authors therefore studied 43 oncology patients treated with standard or pegylated IFN-alpha with baseline psychiatric assessment and at regular time-points in the first 6 months of treatment. Apart from a severe depression because of brain metastases, authors observed only two clinically relevant depressive states. Contrary to findings in most of the literature, most depressive episodes in this study were self-limiting and short-lasting and were associated with either episodes of flu-like symptoms common at the start of the treatment or with concurrent psychosocial events. In the group as a whole, scores on both observer-based and self-report rating scales did not show clinically relevant changes. The results of this study indicate that IFN-alpha treatment is not suitable as a study model for depression in general.

17 [198]. Bellmunt, J., C. Szczylik, et al. (2008). "Temsirolimus safety profile and management of toxic effects in patients with advanced renal cell carcinoma and poor prognostic features." Ann Oncol **19**(8): 1387-1392.

BACKGROUND: Temsirolimus, a novel inhibitor of mammalian target of rapamycin, has demonstrated prolonged overall survival and progression-free survival compared with interferon alfa (IFN) in patients with advanced renal cell carcinoma (RCC) and poor prognostic features. Adverse events (AEs) of any causality were previously reported, but AEs that were deemed temsirolimus related are of particular relevance for poor-risk patients and for defining mammalian target of rapamycin inhibitor-specific side-effects. PATIENTS AND METHODS: Patients with advanced RCC, no prior systemic therapy, and three or more of six poor-risk factors were randomly assigned to one of three groups: (i) IFN s.c. up to 18 MU thrice weekly, (ii) temsirolimus i.v. 25 mg weekly, or (iii) temsirolimus i.v. 15 mg weekly plus interferon s.c. 6 MU thrice weekly. RESULTS: Among 208 patients, the most common temsirolimus-related grades 3-4 AEs were anemia (13%), hyperglycemia (9%), and asthenia (8%). Grades 3-4 hypercholesterolemia (1%), hypertriglyceridemia (3%), and hypophosphatemia (4%) were also seen. Although pneumonitis occurred infrequently, vigilance for its development is needed. Guidelines for management of toxic effects are presented on the basis of available clinical experience. CONCLUSIONS: Temsirolimus-related grades 3-4 AEs were primarily metabolic in nature and easily controlled medically. In general, these did not negatively impact patient quality of life.

18 [237]. Bex, A., J. Kerst, et al. (2005). "Extended continuous oral temozolomide in patients with progressive metastatic renal cell carcinoma not responding to interferon alpha 2b." J Chemother **17**(6): 674-678.

The aim of the study was to evaluate the toxicity and efficacy of oral extended continuous temozolomide in patients with progressive metastatic renal cell carcinoma (RCC) not responding to immunotherapy after removal of the primary tumor. Patients with progressive metastatic RCC received protracted temozolomide 100 mg/m2 orally on days 1-21 every 28 days. Response was assessed after 2 cycles to be followed by another 2 cycles in the absence of progression. After 4 cycles only patients with further remission and acceptable toxicity were to continue. No objective responses were observed in 12 patients and the trial was stopped prematurely in stage 1. Six patients remained stable during 4 cycles of temozolomide (4 months), only one of these remained stable for another 2 months after having stopped treatment. Five patients progressed after the initial 2 cycles and one after the first cycle. Overall survival was 15.5 months (range 1-36 months). Repeated cycles of 3 weeks oral temozolomide 100 mg/m2 followed by one week rest proved tolerable though this regimen may only have limited activity against metastatic RCC.

19 [239]. Bex, A., M. Kerst, et al. (2006). "Interferon alpha 2b as medical selection for nephrectomy in patients with synchronous metastatic renal cell carcinoma: a consecutive study." Eur Urol **49**(1): 76-81.

OBJECTIVE: Up to 25% of the patients with synchronous metastatic renal cell carcinoma (mRCC) treated with nephrectomy and interferon alpha-2b (IFN-alpha) will progress rapidly at metastatic sites and undergo needless surgery for an asymptomatic primary. We reversed the timing of surgery and immunotherapy and evaluated the role of initial IFN-alpha as selection for nephrectomy. PATIENTS AND METHODS: Sixteen patients with mRCC and the primary in-situ received initial IFN-alpha for 8 weeks (2 weeks 5x3x10(6)IU/wk; 2 weeks 5x6x10(6)IU/wk; 2 weeks 5x9x10(6)IU/wk and 2 weeks 3x9x10(6)IU/wk). Patients with either partial remission (PR) or stable disease (SD) underwent nephrectomy followed by IFN-alpha maintenance at 3x9x10(6)IU/wk. Patients were evaluated with regard to age, sex, metastatic sites, morbidity, response, nephrectomy rate, time to progression and survival. RESULTS: Thirteen patients received 2 months of preoperative IFN-alpha; 3 stopped during the 2 months period due to progressive disease (PD). Eight patients developed either a PR (n=3) or SD (n=5) at metastatic sites and underwent nephrectomy. Survival at 1 year is 50% (4/8 patients). Median progression-free survival was 6 months (3-17 months). Two of the 3 patients with PR developed a CR after 2 months maintenance following surgery. Eight patients with PD did not undergo surgery and had a median survival of 4 months (range 1-8 months). CONCLUSIONS: Absence of progression at metastatic sites following IFN-alpha with the primary tumor in place may be used as selection for nephrectomy in patients with an intermediate prognosis. Currently, a randomized study is underway to assess the role of initial versus delayed nephrectomy in combination with IFN-alpha with regard to morbidity and survival.

20 [249]. Bex, A., H. Mallo, et al. (2005). "A phase-II study of pegylated interferon alfa-2b for patients with metastatic renal cell carcinoma and removal of the primary tumor." Cancer Immunol Immunother **54**(7): 713-719.

Twenty-two patients with metastatic renal cell carcinoma and removal of the primary tumor were treated with subcutaneous pegylated interferon alfa-2b (PEG-Intron) to evaluate toxicity and efficacy. Start dose was 3.0 microg/kg/week, escalated to 6.0 microg/kg/week. After 2 months, therapy was extended in case of response or stable disease (SD) until progressive disease (PD) or relapse for a maximum of 2 years. National Cancer Institute common toxicity criteria (NCI-CTC) were monitored every 2-4 weeks. After 2 months, nine patients did not continue (8 PD, 1 SD with grade 4 CTC) and 13 extended treatment [three partial response (PR), 10 SD], of these, 11 progressed. One patient with PR developed a durable complete response later. Overall response rate was 13.6% (3/22). Median overall survival is 13 months (range 3-35 months). Dosage was escalated to 6 microg/kg/week in three patients. NCI-CTC grade 2 and 3 required dose attenuation in 12 patients during escalation, and reduction in 10 during the trial. Three patients discontinued because of grade 4 CTC (two fatigue, one hyperglycemia). Fatigue was the major dose-limiting toxicity. These results suggest an efficacy and toxicity of PEG-Intron comparable to standard interferon alfa-2b in patients with mRCC and removal of the primary tumor.

21 [126]. Blagoev, K. B., J. Wilkerson, et al. (2013). "Sunitinib does not accelerate tumor growth in patients with metastatic renal cell carcinoma." Cell Rep **3**(2): 277-281.

Preclinical studies have suggested that sunitinib accelerates metastases in animals, ascribing this to inhibition of the vascular endothelial growth factor receptor or the tumor's adaptation. To address whether sunitinib accelerates tumors in humans, we analyzed data from the pivotal randomized phase III trial comparing sunitinib and interferon alfa in patients with metastatic renal cell carcinoma. The evidence clearly shows that sunitinib was not harmful, did not accelerate tumor growth, and did not shorten survival. Specifically, neither longer sunitinib treatment nor a greater effect of sunitinib on tumors reduced survival. Sunitinib did reduce the tumor's growth rate while administered, thereby improving survival, without appearing to alter tumor biology after discontinuation. Concerns arising from animal models do not apply to patients receiving sunitinib and likely will not apply to similar agents.

22 [216]. Boorjian, S. A., M. I. Milowsky, et al. (2007). "Phase 1/2 clinical trial of interferon alpha2b and weekly liposome-encapsulated all-trans retinoic acid in patients with advanced renal cell carcinoma." J Immunother **30**(6): 655-662.

To evaluate the feasibility, efficacy, and biologic effects of weekly liposome-encapsulated all-trans retinoic acid (ATRA-IV) plus interferon alpha2b (IFN) in patients with advanced renal cell carcinoma (RCC). Twenty-six patients with metastatic RCC were treated on a phase 1/2 trial with weekly ATRA-IV and IFN SQ daily 5 d/wk. Twelve patients received ATRA-IV at three dose levels (60, 75, and 90 mg/m2) according to phase 1 methodology, and 14 additional patients received 90 mg/m2. Response was assessed according to an intention-to-treat analysis. Serum retinoic acid (RA) concentrations were assayed and peripheral blood mononuclear cell mRNA expression of RA and IFN-inducible genes (RARalpha, RARbeta2, IRF1, CRABP2, and TRAIL) were examined. No dose limiting toxicities occurred at 60 mg/m2; grade 3 leukopenia affected 1/6 patients at 75 mg/m2, whereas 3 patients received 90 mg/m2 without a dose limiting toxicities. Fourteen additional patients received 90 mg/m2 ATRA-IV without grade 3/4 toxicity. Five of 26 (19%) patients achieved a major response, with a median duration of 14 months (range 9 to 23); 9 additional patients (41%) demonstrated stable disease or minor response lasting > or =4 months. No significant differences in serum (RA) after ATRA infusion were detected between weeks 1 and 8 of treatment. Peripheral blood mononuclear cell mRNA expression did not correlate with clinical response. The addition of weekly ATRA-IV to IFN therapy is feasible and well tolerated, resulting in sustainable increased serum (RA). This regimen demonstrates antitumor activity in metastatic RCC, and suggests ATRA-IV augments IFN therapy.

23 [155]. Bracarda, S., J. Bellmunt, et al. (2011). "Overall survival in patients with metastatic renal cell carcinoma initially treated with bevacizumab plus interferon-alpha2a and subsequent therapy with tyrosine kinase inhibitors: a retrospective analysis of the phase III AVOREN trial." BJU Int **107**(2): 214-219.

OBJECTIVE: * To retrospectively evaluate the effect of subsequent tyrosine kinase inhibitors (TKIs) after first-line bevacizumab + interferon-alpha2a (IFN) or IFN + placebo in the phase III AVOREN (Avastin and Roferon in Renal Cell Carcinoma) trial. PATIENTS AND METHODS: * A total of 649 patients with untreated metastatic renal cell carcinoma (mRCC) were randomized to receive IFN (9 MIU three times a week for up to 1 year) in combination with bevacizumab (10 mg/kg every 2 weeks) or placebo until disease progression. * The protocol allowed the use of any post-protocol anti-cancer therapy for patients with progressive disease or those in whom the trial therapy was discontinued. Data regarding the timing and type of subsequent therapy were recorded and overall survival (OS) analysed. RESULTS: * Patients were randomized to bevacizumab + IFN (n= 327) or IFN + placebo (n= 322); 180 (55%) patients in the bevacizumab + IFN, and 202 (63%) in the IFN + placebo arm, received post-protocol anti-cancer therapy. * TKIs were the most common post-protocol therapy, received by 113 (35%) and 120 (37%) patients in the bevacizumab + IFN and IFN + placebo arms, respectively. * The median OS in patients who received any subsequent TKI was 38.6 months in the bevacizumab + IFN arm and 33.6 months in IFN + placebo arm [hazard ratio (HR), 0.80; 95% confidence interval (CI), 0.56-1.13; P= 0.203]. In an additional retrospective analysis that censored patients who received subsequent TKIs, median OS was 25.0 and 20.7 months, respectively, in the bevacizumab + IFN and IFN + placebo arms (HR, 0.84; 95% CI, 0.67-1.05; P= 0.123). CONCLUSIONS: * These retrospective exploratory data of sequential bevacizumab + IFN followed by TKIs in patients able to receive multiple lines of therapy suggest that sequential therapy could be a promising approach to improve patient outcomes in mRCC.

24 [130]. Bracarda, S., C. Porta, et al. (2013). "Could interferon still play a role in metastatic renal cell carcinoma? A randomized study of two schedules of sorafenib plus interferon-alpha 2a (RAPSODY)." Eur Urol **63**(2): 254-261.

BACKGROUND: Sorafenib has proven efficacy in metastatic renal cell carcinoma (mRCC). Interferon (IFN) has antiangiogenic activity that is thought to be both dose- and administration-schedule dependent. OBJECTIVE: To compare two different schedules of IFN combined with sorafenib. DESIGN, SETTING, AND PARTICIPANTS: Single-stage, prospective, noncomparative, randomized, open-label, multicenter, phase 2 study on previously untreated patients with mRCC and Eastern Cooperative Oncology Group performance status 0-2. INTERVENTION: Sorafenib 400mg twice daily plus subcutaneous IFN, 9 million units (MU) three times a week (Arm A) or 3 MU five times a week (Arm B). OUTCOME MEASUREMENTS AND STATISTICAL ANALYSIS: Primary end points were progression-free survival (PFS) for each arm and safety. Data were evaluated according to an intent-to-treat analysis. RESULTS AND LIMITATIONS: A total of 101 patients were evaluated. Median PFS was 7.9 mo in Arm A and 8.6 mo in Arm B (p=0.049) and the median duration of response was 8.5 and 19.2 mo, respectively (p=0.0013). Nine partial responses were observed in Arm A, and three complete and 14 partial responses were observed in Arm B (17.6% vs 34.0%; p=0.058); 24 and 21 patients (47% and 42%), respectively, achieved stable disease. The most common grade 3-4 toxicities were fatigue plus asthenia (28% vs 16%; p=0.32) and hand-foot skin reactions (20% vs 18%). CONCLUSIONS: Sorafenib plus frequent low-dose IFN showed good efficacy and tolerability. Further investigations should be warranted to identify a possible positioning of this intriguing regimen (6% complete response rate) in the treatment scenario of mRCC.

25 [246]. Brinkmann, O. A., F. Bruns, et al. (2005). "Treatment of bone metastases and local recurrence from renal cell carcinoma with immunochemotherapy and radiation." World J Urol **23**(3): 185-190.

Immunotherapies using interferons and/or interleukins are currently the treatment of choice for metastatic renal cell carcinoma (RCC). Bone metastases and non-resectable local recurrence are negative predictors for successful immunotherapy and signs of poor prognosis. The present study was designed to evaluate the effectiveness of combined immunochemotherapy (ICT) and radiation therapy (RT) for bone metastases or local recurrence from RCC in a prospective fashion. From September 1997 to September 1999, 20 patients with progressive RCC were treated with a combination of RT and ICT [s.c. interleukin-2a (IL-2), s.c. interferon alpha (IFN-alpha) and i.v. 5-fluorouracil]. RT started in week 2 of ICT. The radiation field was limited to the symptomatic bone metastases (15 patients) or the local recurrence (five patients). The total dosages of the RT ranged between 45 and 50 Gy, administered in fractions of from 1.8 to 2 Gy daily. In case of objective response or stable disease, the patients received up to two further ICT courses. All patients had good pain relief. Three out of 20 achieved complete remission, three had a partial remission, nine were stable and five patients had progressive disease under the combined treatment. Median survival was 21 months, mean survival 24 months (range: 5-59 months). The side effects of the combined treatment are in the same range as with ICT alone (World Health Organisation grade 2 and 3). Of 20 patients, 19 had their pain medication reduced after treatment. The combination of ICT and RT is feasible. There is remarkable pain relief. Our data suggest that the combination of immunochemotherapy and radiation therapy may induce a synergistic antitumor effect for the treatment of bone metastases or local recurrence from RCC compared to data from the literature for ICT or RT alone.

26 [253]. Bromwich, E., D. C. McMillan, et al. (2004). "The systemic inflammatory response, performance status and survival in patients undergoing alpha-interferon treatment for advanced renal cancer." Br J Cancer **91**(7): 1236-1238.

The prognostic value of C-reactive protein, compared with ECOG performance status (ECOG-ps), in patients receiving alpha-interferon treatment for advanced renal cancer was assessed in 58 patients. In all, 55 patients died on follow-up. On multivariate analysis with ECOG-ps and C-reactive protein entered as covariates, only C-reactive protein was a significant independent predictor of survival (HR 2.03, 95% CI 1.09-3.80, P=0.026).

27 [264]. Capuron, L., A. Ravaud, et al. (2004). "Baseline mood and psychosocial characteristics of patients developing depressive symptoms during interleukin-2 and/or interferon-alpha cancer therapy." Brain Behav Immun **18**(3): 205-213.

It has been suggested that patients with subclinical mood symptoms prior to initiating cytokine treatment (as revealed by elevated baseline scores on depression rating scales) are more likely to become clinically depressed during the course of cytokine therapy. The present study was designed to identify which specific preexisting symptoms predict development of depressive symptomatology during treatment with the cytokines, interleukin-2 (IL-2) and/or interferon-alpha (IFN-alpha), in patients with cancer. Thirty-two patients with renal cell carcinoma or malignant melanoma eligible to receive treatment with IL-2 and/or IFN-alpha were enrolled in the study. At baseline and after one month of cytokine therapy (endpoint), depressive symptoms were assessed using the clinician-administered Montgomery-Asberg depression rating scale (MADRS). Illness-related coping strategies, social support, somatic complaints, quality of sleep and demographic factors were also assessed as relevant baseline predictive factors. MADRS scores significantly increased during cytokine therapy. Patients with moderate to marked depressive symptomatology at study endpoint exhibited higher baseline scores in dimensions of the MADRS scale assessing emotional (especially reported sadness), cognitive (especially pessimistic thoughts) and neurovegetative (sleep disturbances) symptoms compared to patients who remained free of depressive symptoms during cytokine therapy. Interestingly, only emotional symptoms and sleep disturbance at baseline, along with low social support, predicted severity of depressive symptoms at the end of the first month of therapy. By documenting specific behavioral vulnerability factors for cytokine-induced depressive symptoms, these findings may help identify patients at risk for mood disturbances during cytokine treatment and help target specific patient populations and specific symptoms for preventative strategies.

28 [178]. Castellano, D., X. G. del Muro, et al. (2009). "Patient-reported outcomes in a phase III, randomized study of sunitinib versus interferon-{alpha} as first-line systemic therapy for patients with metastatic renal cell carcinoma in a European population." Ann Oncol **20**(11): 1803-1812.

BACKGROUND: The purpose of this study is to evaluate the impact on the health-related quality of life (HRQoL) of sunitinib versus interferon-alpha (IFN-alpha) treatment in patients with metastatic renal cell carcinoma (mRCC). PATIENTS AND METHODS: In all, 304 mRCC patients (European cohort) were randomized 1 : 1 to receive sunitinib (50 mg/day for 4 weeks, followed by 2 weeks off) or IFN-alpha (9 million units s.c. injection three times/week). The following questionnaires were completed (days 1 and 28 per cycle): Functional Assessment of Cancer Therapy-General (FACT-G), the FACT-Kidney Symptom Index and the EuroQol Group's EQ-5D self-report questionnaire (EQ-5D). Results correspond to an ongoing trial with progression-free survival time as primary end point, and patients were still being followed up. Data were analyzed using repeated measures mixed effects models (MEMs) that allow the inclusion of initial differences and uncompleted repeated measures, with the assumption of data missing at random. Six-cycle results were included. RESULTS: Results consistently showed that patients in sunitinib group experienced statistically significantly milder kidney-related symptoms, better cancer-specific HRQoL and general health status (in social utility scores) during the study period as measured by these patient-reported outcome end points. No statistical differences between groups were found on the FACT-G physical well-being subscale or the EQ-5D VAS values. CONCLUSIONS: Results from MEM showed the sunitinib's benefit on HRQoL compared with IFN-alpha.

29 [191]. Cella, D., J. Z. Li, et al. (2008). "Quality of life in patients with metastatic renal cell carcinoma treated with sunitinib or interferon alfa: results from a phase III randomized trial." J Clin Oncol **26**(22): 3763-3769.

PURPOSE: In an international, randomized phase III trial, sunitinib demonstrated statistically significant efficacy over interferon alfa (IFN-alpha) as first-line therapy in patients with metastatic renal cell carcinoma (mRCC) (progression-free survival time, 11 v 5 months, respectively; P < .001; objective response rate, 31% v 6%, respectively; P < .001). We report health-related quality-of-life (QOL) results from this trial. PATIENTS AND METHODS: Seven hundred fifty mRCC patients were randomly assigned to sunitinib (6-week cycles: 50 mg orally once daily for 4 weeks, followed by 2 weeks off) or IFN-alpha (9 million units subcutaneous injections, three times weekly). QOL measures included the Functional Assessment of Cancer Therapy-General (FACT-G), the FACT-Kidney Symptom Index-15 item (FKSI-15), and the EuroQoL-5D's utility score (EQ-5D Index) and its visual analog scale (EQ-VAS). The primary QOL end point was the FKSI Disease-Related Symptoms (FKSI-DRS) subscale. Higher scores indicated better outcomes (better QOL or fewer symptoms). Data were analyzed for the intent-to-treat population using mixed-effects models, supplemented with pattern-mixture models. RESULTS: Patients receiving sunitinib reported higher FKSI-15 and FKSI-DRS scores at each cycle than those receiving IFN-alpha, with a significant difference in the overall least squares means (3.27 and 1.98, respectively; P < .0001). Similarly, differences in least squares means for FACT-G (and all subscales), EQ-5D Index, and EQ-VAS were all significantly favorable for sunitinib (P < .01). Per pre-established thresholds, between-treatment differences in the mean scores were clinically meaningful after cycle 4 for FKSI-DRS and at all assessments for FKSI-15, FACT-G, and the FACT-G functional well-being subscale. CONCLUSION: Sunitinib provides superior QOL compared with IFN-alpha in mRCC patients.

30 [166]. Cella, D., M. D. Michaelson, et al. (2010). "Health-related quality of life in patients with metastatic renal cell carcinoma treated with sunitinib vs interferon-alpha in a phase III trial: final results and geographical analysis." Br J Cancer **102**(4): 658-664.

BACKGROUND: In a randomised phase III trial, sunitinib significantly improved efficacy over interferon-alpha (IFN-alpha) as first-line therapy for metastatic renal cell carcinoma (mRCC). We report the final health-related quality of life (HRQoL) results. METHODS: Patients (n=750) received oral sunitinib 50 mg per day in 6-week cycles (4 weeks on, 2 weeks off treatment) or subcutaneous IFN-alpha 9 million units three times weekly. Health-related quality of life was assessed with nine end points: the Functional Assessment of Cancer Therapy-General and its four subscales, FACT-Kidney Symptom Index (FKSI-15) and its Disease-Related Symptoms subscale (FKSI-DRS), and EQ-5D questionnaire's EQ-5D Index and visual analogue scale. Data were analysed using mixed-effects model (MM), supplemented with pattern-mixture models (PMM), for the total sample and the US and European Union (EU) subgroups. RESULTS: Patients receiving sunitinib reported better scores in the primary end point, FKSI-DRS, across all patient populations (P<0.05), and in nine, five, and six end points in the total sample, in the US and EU groups respectively (P<0.05). There were no significant differences between the US and EU groups for all end points with the exception of the FKSI item 'I am bothered by side effects of treatment' (P=0.02). In general, MM and PMM results were similar. CONCLUSION: Patients treated with sunitinib in this study had improved HRQoL, compared with patients treated with IFN-alpha. Treatment differences within the US cohort did not differ from those within the EU cohort.

31 [206]. Clark, J. I., J. Mehrabi, et al. (2007). "Phase I/II trial of outpatient PEG-interferon with interleukin-2 in advanced renal cell carcinoma: a cytokine working group study." J Immunother **30**(8): 839-846.

A phase I/II trial was undertaken to determine the maximum tolerated dose of polyethylene glycol interferon-alpha-2b (PEG-IFN) with interleukin-2 (IL-2), and to evaluate the efficacy and toxicity in patients with metastatic renal cell carcinoma. Patients initially received subcutaneous PEG-IFN, 3.0 mcg/kg/wk, combined with IL-2, but owing to unexpected toxicity a revised phase I schedule ensued. Patients received 1.0, 1.5, 2.0, or 3.0 mcg/kg/wk of PEG-IFN on days 1, 8, 15, and 22; subcutaneous IL-2 was given at a dose of 5 x 10 IU/m2 every 8 hours x 3 on day 1, followed daily at 5 x 10 IU/m2 days 2, 3, 4, and 5 of week 1, then 5 times per week for 3 weeks, followed by 2 weeks off. The maximum tolerated dose of PEG-IFN was 2.0 mcg/kg/wk. Fifty-four patients were enrolled. Frequent grade III/IV cardiac and neurologic toxicities led to an expanded phase I trial. Eleven serious events in 33 patients in the phase II portion led to early termination. No patient died from treatment. The overall response rate in 53 evaluable patients was 30.2% (95% confidence interval 20.5-39.9), with 2 complete responses and 14 partial responses and at least 1 response at each dose level. The median duration of response was 11 months (range, 2 to 65+ mo); median survival was 20 months (range, 2 to 71+ mo); median time to progression was 4 months. Despite clinical efficacy, the study was closed prematurely owing to excess toxicity. Although all serious adverse events resolved, this degree of toxicity is unacceptable for an outpatient treatment regimen.

32 [257]. Clark, P. E., M. C. Hall, et al. (2004). "Phase II trial of combination interferon-alpha and thalidomide as first-line therapy in metastatic renal cell carcinoma." Urology **63**(6): 1061-1065.

OBJECTIVES: To present the results of a Phase II trial of thalidomide and interferon-alpha in renal cell carcinoma. METHODS: Patients with metastatic clear cell renal cell carcinoma and no prior systemic therapy were accrued. Interferon-alpha was administered at 5 million units subcutaneously three times per week. Thalidomide was started at 100 mg/day for 2 weeks and then escalated 200 mg every 2 weeks to 1000 mg or until grade 3-4 toxicity developed. Patients were assessed radiographically at baseline and after 12 weeks. Steady-state thalidomide plasma concentrations were determined. RESULTS: Thirty patients were enrolled. The median age was 62 years. Seventeen patients (57%) had undergone nephrectomy before therapy. One patient died during therapy. Of the 30 patients, 29 had at least grade 2 toxicity and 17 patients had at least grade 3. At 12 weeks, no patient had a complete response, 2 had a partial response (6.7%), 8 had stable disease (26.7%), and 11 (including 1 patient with an initial partial response) had disease progression (36.7%). Nine patients were removed from the study before 12 weeks. The median follow-up was 49.6 weeks (range 2.4 to 123.7). The median time of participation in the study was 11.1 weeks (range 1.4 to 63.9). At last follow-up, 2 patients were receiving the study therapy, 1 with stable disease at 64 weeks and 1 with a partial response at 53 weeks. The median survival was 68 weeks. A linear relationship was found between the thalidomide plasma concentration and dose. No relationship was apparent between the concentration and either treatment-related toxicity or response. CONCLUSIONS: Interferon-alpha and thalidomide as front-line therapy for metastatic renal cell carcinoma showed limited activity. The objective response rate was 7%. One third of patients experienced toxicity that required discontinuation of thalidomide. Randomized controlled studies are needed to determine any objective benefit of this regimen over either drug alone.

33 [240]. Dannull, J., Z. Su, et al. (2005). "Enhancement of vaccine-mediated antitumor immunity in cancer patients after depletion of regulatory T cells." J Clin Invest **115**(12): 3623-3633.

In this study, we investigated whether elimination of CD4+/CD25+ Tregs using the recombinant IL-2 diphtheria toxin conjugate DAB(389)IL-2 (also known as denileukin diftitox and ONTAK) is capable of enhancing the immunostimulatory efficacy of tumor RNA-transfected DC vaccines. We show that DAB(389)IL-2 is capable of selectively eliminating CD25-expressing Tregs from the PBMCs of cancer patients without inducing toxicity on other cellular subsets with intermediate or low expression of CD25. DAB(389)IL-2-mediated Treg depletion resulted in enhanced stimulation of proliferative and cytotoxic T cell responses in vitro but only when DAB(389)IL-2 was omitted during T cell priming. DAB(389)IL-2 significantly reduced the number of Tregs present in the peripheral blood of metastatic renal cell carcinoma (RCC) patients and abrogated Treg-mediated immunosuppressive activity in vivo. Moreover, DAB(389)IL-2-mediated elimination of Tregs followed by vaccination with RNA-transfected DCs significantly improved the stimulation of tumor-specific T cell responses in RCC patients when compared with vaccination alone. Our findings may have implications in the design of immune-based strategies that may incorporate the Treg depletion strategy to achieve potent antitumor immunity with therapeutic impact.

34 [234]. Dillman, R. O., M. C. Wiemann, et al. (2006). "Phase II trial of subcutaneous interferon followed by intravenous hybrid bolus/continuous infusion interleukin-2 in the treatment of renal cell carcinoma: final results of Cancer Biotherapy Research Group 95-09." Cancer Biother Radiopharm **21**(2): 130-137.

OBJECTIVE: We conducted a phase II trial in metastatic renal cell cancer of outpatient subcutaneous (s.c.) interferon-alpha2b (IFN), followed by an inpatient hybrid schedule of bolus and continuous interleukin-2 (IL- 2). METHODS: Treatment consisted of monthly IFN 10 MU/m(2) s.c. for 4 consecutive days, followed by 36 MIU/m(2) bolus IL-2, then 72-hour continuous intravenous (i.v.) infusion of 18 MIU/m(2) IL-2 per day. Between May 1997 and June 2000, 25 men and 11 women enrolled, with a median age of 57 years (range, 42-77), including 9 patients over 65. Prior treatment included nephrectomy (31), radiation (8), biotherapy (7), and chemotherapy (4). Sites of disease included 26 lung, 13 lymph node, 9 bone, 8 liver, 4 kidney, and 4 adrenal locations. Patients received an average of 3.1 treatment cycles (range, 1-6). RESULTS: There was 1 complete and 3 partial responses, for a response rate of 11% (3% to 27%; 95% confidence interval [CI]); 40% had stable disease. Median failure-free survival was 2.5 months; median overall survival was 15.0 months. The 1-, 2-, and 5-year survival rates were 53%, 30%, and 12%, respectively. Only 8 patients required a reduction in IL-2 dose. The most frequent grade 3 or 4 toxicities were 11% fatigue, 9% renal insufficiency, and 7% hypotension. CONCLUSIONS: Response and survival rates were similar to those seen in other multicenter trials using inpatient high-dose IL-2.

35 [269]. Donskov, F., N. Marcussen, et al. (2004). "In vivo assessment of the antiproliferative properties of interferon-alpha during immunotherapy: Ki-67 (MIB-1) in patients with metastatic renal cell carcinoma." Br J Cancer **90**(3): 626-631.

The aim of the present study was to investigate the in vivo antiproliferative effect of interferon alpha (IFN-alpha) in patients with metastatic renal cell carcinoma (mRCC). Core needle biopsies of metastatic and/or the primary kidney cancer were obtained before interleukin-2 (IL-2)- and IFN-alpha-based immunotherapy in 34 patients and repeated after 5 weeks in 25 patients. Tumour proliferation was assessed by use of the anti-Ki-67 antibody MIB-1 and evaluated in multiple, random systematic sampled fields of vision. Ki-67 labelling index (LI) at baseline was median 13.6% (range 1.2-85.0) and median 10.6% (range 1.3-48.6%) at week 5 with a median overall decline of 15.2% (range -95 to +258%) from baseline to week 5. There was no difference between responding and nonresponding patients. Ki-67 LI at week 5 was significantly correlated to survival. Thus, median survival of patients with Ki-67 LI <or=10.6% at week 5 was 25.1 months compared to 11.5 months for patients with Ki-67 LI >10.6% (P=0.016). Baseline or change in Ki-67 LI did not correlate to survival. These data suggest that IFN-alpha in vivo has only modest effect on tumour proliferation in patients with mRCC. Tumour Ki-67 (MIB-1) reactivity after 1 month of immunotherapy appears to be a significant predictor of patient survival.

36 [184]. Dutcher, J. P., P. de Souza, et al. (2009). "Effect of temsirolimus versus interferon-alpha on outcome of patients with advanced renal cell carcinoma of different tumor histologies." Med Oncol **26**(2): 202-209.

Purpose Exploratory subgroup analyses from the phase 3 global advanced renal cell carcinoma (ARCC) trial were conducted to assess the influence of tumor histology on outcome of patients treated with temsirolimus (Torisel) or interferon-alpha (IFN). Patients and methods Patients with ARCC including clear cell and other types such as papillary and chromophobe histologies received either IFN (3 million units [MU] subcutaneously three times weekly, escalating to 18 MU) or temsirolimus (25 mg intravenously weekly). Results Approximately 80% of patients had clear cell and 20% of patients had other histologies, the majority of which were papillary. Patients with clear cell and other RCC histologies, treated with temsirolimus, demonstrated comparable median overall and progression-free survival. In contrast, patients with other RCC histologies, treated with IFN, demonstrated shorter median overall and progression-free survival than patients with clear cell RCC. Hazard ratios for death for treatment with temsirolimus versus IFN were less than 1 for patients regardless of tumor histology. For patients treated with temsirolimus, 59% with clear cell and 68% with other RCC histologies experienced tumor reductions. For patients treated with IFN, 35% with clear cell and 14% with other RCC histologies had tumor reductions. However, temsirolimus did not appear to improve the objective response rate compared to IFN. Temsirolimus resulted in a superior clinical benefit rate compared with IFN, regardless of tumor histology. Conclusion Temsirolimus appears to be efficacious in patients with clear cell and non-clear cell histologies and can, therefore, be used for the treatment of all types of RCC.

37 [162]. Escudier, B., J. Bellmunt, et al. (2010). "Phase III trial of bevacizumab plus interferon alfa-2a in patients with metastatic renal cell carcinoma (AVOREN): final analysis of overall survival." J Clin Oncol **28**(13): 2144-2150.

PURPOSE: A phase III trial of bevacizumab combined with interferon alfa-2a (IFN) showed significant improvements in progression-free survival (PFS) in metastatic renal cell carcinoma (mRCC). Here, we report overall survival (OS) data. PATIENTS AND METHODS: Six hundred forty-nine patients with previously untreated mRCC were randomly assigned to receive bevacizumab (10 mg/kg every 2 weeks) plus IFN (9 MIU subcutaneously three times a week; n = 327) or IFN plus placebo (n = 322) in a multicenter, randomized, double-blind, phase III trial. The primary end point was OS. Final analysis of the secondary end point (PFS) was reported earlier. RESULTS: Median OS was 23.3 months with bevacizumab plus IFN and 21.3 months with IFN plus placebo (unstratified hazard ratio [HR] = 0.91; 95% CI, 0.76 to 1.10; P = .3360; stratified HR = 0.86; 95% CI, 0.72 to 1.04; P = .1291). Patients (> 55%) in both arms received at least one postprotocol antineoplastic therapy, possibly confounding the OS analysis. Patients receiving postprotocol therapy including a tyrosine kinase inhibitor had longer median OS (bevacizumab plus IFN arm: 38.6 months; IFN plus placebo arm: 33.6 months; HR = 0.80; 95% CI, 0.56 to 1.13). Tolerability was similar to that reported previously. CONCLUSION: Bevacizumab plus IFN is active as first-line treatment in patients with mRCC. Most patients with mRCC receive multiple lines of therapy, so considering the overall sequence of therapy when selecting first-line therapy may optimize patient benefit.

38 [221]. Escudier, B., N. Lassau, et al. (2007). "Phase I trial of sorafenib in combination with IFN alpha-2a in patients with unresectable and/or metastatic renal cell carcinoma or malignant melanoma." Clin Cancer Res **13**(6): 1801-1809.

PURPOSE: To determine the safety, maximum tolerated dose, pharmacokinetics, and efficacy, and to evaluate biomarkers, of the multikinase inhibitor sorafenib plus IFN alpha-2a in advanced renal cell carcinoma (RCC) or melanoma. EXPERIMENTAL DESIGN: Patients received 28-day cycles of continuous, oral sorafenib twice daily and s.c. IFN thrice weekly: sorafenib 200 mg twice daily plus IFN 6 million IU (MIU) thrice weekly (cohort 1); and sorafenib 400 mg twice daily plus IFN 6 MIU thrice weekly (cohort 2); or plus IFN 9 MIU thrice weekly (cohort 3). Tumor response was assessed by Response Evaluation Criteria in Solid Tumors and dynamic contrast-enhanced ultrasonography. RESULTS: Thirteen patients received at least one dose of sorafenib plus IFN (12 RCC; one melanoma). The maximum tolerated dose was not reached [only one dose-limiting toxicity (grade 3 asthenia)]. Most frequently reported drug-related adverse events were grade 2 or less in severity, including fatigue, diarrhea, nausea, alopecia, and hand-foot skin reaction. One (7.7%) RCC patient achieved partial response and eight (61.5%) had stable disease (including the melanoma patient). Good responders assessed by dynamic contrast-enhanced ultrasonography had increased progression-free survival and overall survival, relative to poor responders. IFN had no effect on the pharmacokinetics of sorafenib. There were no significant changes in absolute values of lymphocytes, levels of proangiogenic cytokines, or inhibition of phosphorylated extracellular signal-regulated kinase in T cells or natural killer cells, with combination therapy. CONCLUSIONS: This sorafenib combination was well tolerated, with preliminary antitumor activity in advanced RCC and melanoma patients. There were no drug-drug interactions and the recommended dose for future studies is sorafenib 400 mg twice daily plus IFN 9 MIU.

39 [204]. Escudier, B., A. Pluzanska, et al. (2007). "Bevacizumab plus interferon alfa-2a for treatment of metastatic renal cell carcinoma: a randomised, double-blind phase III trial." Lancet **370**(9605): 2103-2111.

BACKGROUND: Vascular endothelial growth factor (VEGF) inhibition is a valid therapeutic approach in renal cell carcinoma. Therefore, an investigation of the combination treatment of the humanised anti-VEGF monoclonal antibody bevacizumab with interferon alfa was warranted. METHODS: In a multicentre, randomised, double-blind, phase III trial, 649 patients with previously untreated metastatic renal cell carcinoma were randomised to receive interferon alfa-2a (9 MIU subcutaneously three times weekly) and bevacizumab (10 mg/kg every 2 weeks; n=327) or placebo and interferon alfa-2a (n=322). The primary endpoint was overall survival. Secondary endpoints included progression-free survival and safety. An interim analysis of overall survival was prespecified after 250 deaths. On the basis of new second-line therapies that became available while the trial was in progress, which could have confounded analyses of overall survival data, we agreed with regulatory agencies that the pre-planned final analysis of progression-free survival would be acceptable for regulatory submission. The protocol was amended to allow the study to be unblinded at this point. The final analysis of progression-free survival is reported here. Efficacy analyses were done by intention to treat. This trial is registered with centerwatch.com, number BO17705E. FINDINGS: 325 patients in the bevacizumab plus interferon alfa group and 316 in the placebo plus interferon alfa group received at least one dose of study treatment. At the time of unblinding, 230 progression events had occurred in the bevacizumab plus interferon alfa group and 275 in the control group; there were 114 deaths in the bevacizumab plus interferon alfa group and 137 in the control group. Median duration of progression-free survival was significantly longer in the bevacizumab plus interferon alfa group than it was in the control group (10.2 months vs 5.4 months; HR 0.63, 95% CI 0.52-0.75; p=0.0001). Increases in progression-free survival were seen with bevacizumab plus interferon alfa irrespective of risk group or whether reduced-dose interferon alfa was received. Deaths due to adverse events were reported in eight (2%) patients who received one or more doses of bevacizumab and seven (2%) of those who did not receive the drug. Only three deaths in the bevacizumab arm were considered by investigators to be possibly related to bevacizumab. The most commonly reported grade 3 or worse adverse events were fatigue (40 [12%] patients in the bevacizumab group vs 25 [8%] in the control group) and asthenia (34 [10%] vs 20 [7%]). INTERPRETATION: The combination of bevacizumab with interferon alfa as first-line treatment in patients with metastatic renal cell carcinoma results in a significant improvement in progression-free survival, compared with interferon alfa alone.

40 [187]. Escudier, B., C. Szczylik, et al. (2009). "Randomized phase II trial of first-line treatment with sorafenib versus interferon Alfa-2a in patients with metastatic renal cell carcinoma." J Clin Oncol **27**(8): 1280-1289.

PURPOSE: An open-label, phase II study to evaluate progression-free survival (PFS), overall best response, adverse events (AEs), and patient-reported outcomes with sorafenib versus interferon alfa-2a (IFN-alpha-2a) in patients with untreated, advanced renal cancer. PATIENTS AND METHODS: A total of 189 patients were randomly assigned to oral sorafenib 400 mg twice daily or to subcutaneous IFN-alpha-2a 9 million U three times weekly (period 1). Sorafenib patients who progressed were dose-escalated to 600 mg twice daily; IFN-alpha-2a patients who progressed were switched to sorafenib 400 mg twice daily (period 2). RESULTS: In period 1 PFS was similar for sorafenib-treated (n = 97; 5.7 months) and IFN-alpha-2a-treated patients (n = 92; 5.6 months); more sorafenib-treated patients had tumor shrinkage (68.2% v 39.0%). Common drug-related AEs (Grades > or = 3) for sorafenib were hand-foot skin reaction (11.3%), diarrhea (6.2%), and rash/desquamation (6.2%); for IFN-alpha-2a, these were fatigue (10.0%), nausea (3.3%), flu-like syndrome (2.2%), and anorexia (2.2%). Sorafenib-treated patients reported fewer symptoms, better quality of life (QOL), and greater treatment satisfaction. In period 2, 41.9% of patients who received sorafenib 600 mg twice daily (n = 43) experienced tumor reduction (median PFS, 3.6 months). After the switch to sorafenib 400 mg twice daily, tumors were reduced in 76.2% of 50 patients (median PFS, 5.3 months). AEs were mostly grade 1 to 2; no increase in AEs of grades > or = 3 occurred after sorafenib dose escalation. CONCLUSION: In this study, sorafenib resulted in similar PFS as IFN-alpha-2a in patients with untreated RCC. However, sorafenib-treated patients experienced greater rates of tumor size reduction, better QOL, and improved tolerability. Both dose escalation of sorafenib after progression and a switch to sorafenib after progression on IFN-alpha-2a resulted in clinical benefit.

41 [129]. Eto, M., T. Kamba, et al. (2013). "STAT3 polymorphism can predict the response to interferon-alpha therapy in patients with metastatic renal cell carcinoma." Eur Urol **63**(4): 745-752.

BACKGROUND: In our 2007 retrospective study, we reported that single nucleotide polymorphisms (SNPs) in the signal transducer and activator of transcription 3 (acute-phase response factor) (STAT3) gene were significantly associated with better response to interferon (IFN)-alpha in patients with metastatic renal cell carcinoma (mRCC). OBJECTIVE: To prospectively confirm those results, the Japan Immunotherapy SNPs-Study Group for Kidney Cancer conducted this trial. DESIGN, SETTING, AND PARTICIPANTS: In this multicenter, prospective study, 203 eligible patients were enrolled. We evaluated the correlation between the antitumor effects of IFN-alpha and 11 SNPs (STAT3-2, STAT3-0, SOCS3-1, IL4R-34, PTGS1-3, PTGS1-4, PTGS1-5, PTGS2-12, IRF2-67, ICSBP-38, and TAP2-5) in eight genes in 180 patients who received IFN-alpha for >12 wk. INTERVENTIONS: Patients were treated with three doses per week of IFN-alpha 5 million IU. OUTCOME MEASUREMENTS AND STATISTICAL ANALYSIS: We analyzed the association of response to IFN-alpha and overall survival (OS) with genetic polymorphisms using a chi-square test and a logistic regression model. RESULTS AND LIMITATIONS: The response rate of IFN-alpha was 13.8% (28 of 203 patients; 9 complete responses [CRs], 19 partial responses [PRs]). The CR rate of 4.4% was higher than we expected. Response to IFN-alpha was not associated with any of the 11 SNPs examined. However, when we assessed patients with CR, PR, and stable disease >24 wk as a group representing those with clinical response, a significant association was observed between STAT3-2 (rs1905341) and the clinical response of IFN-alpha (p=0.039). Namely, C/C genotype of STAT3-2 was significantly associated with the clinical response of IFN-alpha and OS. These results were generated in Japanese patients and should be studied in other ethnic groups. CONCLUSIONS: This is the first prospective study demonstrating that a STAT3 polymorphism can be a predictive marker for treatment with IFN-alpha for patients with mRCC.

42 [194]. Feldman, D. R., G. V. Kondagunta, et al. (2008). "Phase II trial of pegylated interferon-alpha 2b in patients with advanced renal cell carcinoma." Clin Genitourin Cancer **6**(1): 25-30.

BACKGROUND: Pegylated interferon (IFN) has a longer serum half-life compared with standard IFN, and this allows for weekly dosing. In this study, the efficacy and toxicity of pegylated IFN was assessed in patients with metastatic renal cell carcinoma (mRCC). PATIENTS AND METHODS: Thirty-two patients with previously untreated mRCC were treated with pegylated IFN-alpha 2b in a prospective, single-arm phase II trial. Pegylated IFN was given by subcutaneous administration on a weekly schedule at a dose of 4.5 microg/kg. RESULTS: Of the 32 assessable patients, 29 (91%) had a nephrectomy previously, and none had been treated previously with systemic therapy. Forty-one percent had good-risk, 53% had intermediate-risk, and 6% had poor-risk features per Memorial Sloan-Kettering Cancer Center risk criteria. The best response was a complete response (CR) in 1 patient (3%). Nine patients (28%) had a partial response. Fifteen patients (47%) had stable disease. The median progression-free survival (PFS) was 5 months (95% CI, 3-7 months), and median overall survival was 31 months (95% CI, 18 months to not reached). Five patients had a prolonged PFS of > or = 17 months, 1 of whom achieved a CR. There were no grade 4 toxicities; primary grade 3 toxicities were hematologic (11 of 32 patients; 34%) and fatigue (4 of 32 patients; 13%). CONCLUSION: Pegylated IFN administered weekly has antitumor activity in patients with mRCC with predominantly good- and intermediate-risk features. This study suggests comparable efficacy and safety compared with standard IFN-alpha.

43 [179]. Figlin, R. A., P. de Souza, et al. (2009). "Analysis of PTEN and HIF-1alpha and correlation with efficacy in patients with advanced renal cell carcinoma treated with temsirolimus versus interferon-alpha." Cancer **115**(16): 3651-3660.

BACKGROUND: Exploratory subgroup analyses from the phase 3 global advanced renal cell carcinoma (ARCC) trial were conducted to determine if baseline levels of the tumor molecular markers PTEN and HIF1 alpha correlated with efficacy in patients treated with temsirolimus (Torisel) versus interferon-alpha (IFN). METHODS: Patients in the IFN group received 3 million U (MU) subcutaneously 3x weekly, escalating to 18 MU. Patients in the temsirolimus group received 25 mg intravenously weekly. PTEN and HIF1 alpha baseline levels were measured in archived tumor specimens by immunohistochemistry. RESULTS: There was no correlation between baseline PTEN and HIF1 alpha levels and treatment effect with respect to overall survival (OS), progression-free survival, or objective response rate (ORR) in patients with advanced renal cell carcinoma with poor-risk prognostic factors. CONCLUSIONS: The baseline status of the molecular markers PTEN and HIF1 alpha did not correlate with efficacy in renal cell carcinoma patients treated with temsirolimus versus IFN. Patients demonstrated OS and progression-free survival benefit when treated with temsirolimus regardless of PTEN and HIF1 alpha status. Thus, baseline PTEN and HIF-1 levels may not predict response to temsirolimus. Alternatively, the lack of correlation may be due to the variability in tumor specimens that occurred because of the global nature of the clinical trial. Other markers in the phosphoinositide 3-kinase (PI3K)/Akt pathway may be of utility as predictors of response to temsirolimus in patients with advanced renal cell carcinoma.

44 [203]. Fishman, M., T. B. Hunter, et al. (2008). "Phase II trial of B7-1 (CD-86) transduced, cultured autologous tumor cell vaccine plus subcutaneous interleukin-2 for treatment of stage IV renal cell carcinoma." J Immunother **31**(1): 72-80.

We report a single center phase II trial of sequential vaccination followed with vaccine plus interleukin-2 (IL-2). Vaccination consisted of autologous cells cultured from primary tumor or resected metastasis, transduced to express B7.1 surface molecule and then irradiated. The vaccine would hypothetically costimulate tumor-reactive T cells before IL-2 exposure. Treatment plan was 3 subcutaneous vaccine injections at 4-week intervals and subcutaneous IL-2 treatment for 6 weeks starting at week 7. Sixty-six patients enrolled, of whom 39 received at least 1 vaccine; most observed toxicity was attributable to IL-2 not vaccine; best responses were 3% pathologic complete response, 5% partial response, 64% stable disease, and 28% disease progression. Median survival was 21.8 months (95% confidence interval 17.8 to 29.6). Significant postvaccination increases in IFN-gamma responses to autologous tumor were observed in 2/26 cases. Eighty-one percent of posttreatment subdermal delayed-type hypersensitivity tests (using nontransduced, irradiated autologous tumor cells) had biopsies demonstrating injection site lymphocytic infiltration. Post hoc comparison of the median survival of subjects whose biopsies had lymphocytic infiltration appears longer than in the 19% noninfiltrated (28.4 vs. 17.8 mo, P=0.045, two-sided log-rank test). The single arm design precludes conclusive comparison of objective response rates (not different here) or median survival (longer here) versus those of historical series using similar IL-2 schedules alone. Better outcomes could be logically associated to vaccine response (detectable lymphocytic infiltrates) or to random events that a single arm study design cannot address. This vaccine approach may merit further clinical development.

45 [268]. Flanigan, R. C., G. Mickisch, et al. (2004). "Cytoreductive nephrectomy in patients with metastatic renal cancer: a combined analysis." J Urol **171**(3): 1071-1076.

PURPOSE: Metastatic renal cancer is associated with a poor prognosis. Recent advances in immunotherapy for this problem have rekindled interest in cytoreductive nephrectomy. We report a combined analysis of 2 prospective randomized trials that used an identical study protocol. MATERIALS AND METHODS: A total of 331 patients were randomized to 2 identical protocols comparing cytoreductive nephrectomy plus interferon alpha-2b vs interferon alpha-2b alone in patients with metastatic renal cancer, in whom the primary tumor was present and believed to be resectable. The primary end point for each trial was overall survival with a secondary end point of the response rate. Patients were stratified at pre-randomization by performance status (0 or 1), site of metastases (lung only vs other) and disease measurability. All results were analyzed by intent to treat criteria. Assuming a median survival of 1 year for interferon only, the Southwest Oncology Group trial was designed to detect a 50% improvement in median survival duration and a 15% improvement in response rate with a power of 0.85. The European Organization for the Research and Treatment of Cancer accrued an additional 80 patients in that study. RESULTS: The combined analysis of these 2 trials yielded a median survival of 13.6 months for nephrectomy plus interferon vs 7.8 months for interferon alone. This difference represents a 31% decrease in the risk of death (p = 0.002). There was no evidence of a difference in the size of the treatment effect according to pre-randomization stratification factors. CONCLUSIONS: Cytoreductive nephrectomy appears to improve significantly overall survival in patients with metastatic renal cancer treated with interferon immunotherapy independent of patient performance status, the site of metastases and the presence of measurable disease. Although it is highly statistically significant, the overall survival advantage is only 5.8 months for the entire group. These data emphasize the need to determine if this survival advantage can be further improved using more aggressive immunotherapy or other novel agents in the setting of cytoreductive nephrectomy.

46 [254]. Fossa, S. D., G. H. Mickisch, et al. (2004). "Interferon-alpha-2a with or without 13-cis retinoic acid in patients with progressive, measurable metastatic renal cell carcinoma." Cancer **101**(3): 533-540.

BACKGROUND: In patients with metastatic renal cell carcinoma (MRCC), interferon-alpha (IFN) monotherapy leads to response rates of 5-15%, dependent on the selection of patients. In 1995, preclinical and clinical data indicated an improvement of these results if IFN was combined with 13-cis retinoic acid (CRA). METHODS: In a randomized Phase II study, patients with measurable MRCC received either subcutaneous IFN (9 MU daily; Arm A) or the same daily subcutaneous dose of IFN plus oral CRA (1 mg/kg; Arm B). A central expert panel reviewed the X-ray documentation of objective responses. RESULTS: In the 50 eligible patients from Arm A, the objective, expert-reviewed response rate was 6% (95% confidence interval [95% CI], 1.3-16.6%; 2 complete responses [CRs] and 1 partial response [PR]). A 19% response rate (95% CI, 9.4-32.0%) was stated for 53 eligible patients from Arm B (2 CRs and 8 PRs). Only one of the four CRs claimed by the clinical investigator was confirmed by the central review committee. Conversely, the expert committee deemed that 3 of 12 investigator-stated PRs were CRs. Constitutional toxicity (flu-like symptoms) and/or side effects from skin, mucosa, or eyes led to discontinuation of treatment in 22% of nonprogressing patients, more often in Arm B than in Arm A. CONCLUSIONS: The results from this randomized Phase II study support expansion of the trial into a Phase III study to evaluate the effect of IFN-CRA combination therapy on the survival of patients who undergo nephrectomy prior to IFN-based immunotherapy. The considerable interobserver variability of response evaluation (individual investigator vs. expert panel) indicates the necessity of a central review of claimed responses in future Phase II studies involving patients with MRCC.

47 [133]. Fujioka, T., W. Obara, et al. (2012). "Evidence-based clinical practice guideline for renal cell carcinoma: the Japanese Urological Association 2011 update." Int J Urol **19**(6): 496-503.

Remarkable advances have been made in medical practice in relation to renal cell carcinoma in recent years, and a large amount of new evidence has been accumulated. In keeping with the plan at the time the first version of the "Evidence-Based Clinical Practice Guideline for Renal Cell Carcinoma" compiled by the Japanese Urological Association was published in 2007, the Japanese Urological Association has just published a revised 2011 version. The main revisions regard the selection of treatment methods according to prognostic factors, reconsideration of treatment methods for small-diameter renal cell carcinoma and selection criteria for medical treatment of advanced renal cell carcinoma, including selection of neoadjuvant treatment with molecular targeted medicines. This Guideline presents clinical practice methods that are thought to be the most standard methods in Japan at the present time. In this English translation of a shortened version of the original Guideline, we cited particularly important clinical questions and references.

48 [153]. Garcia, J. A., T. Mekhail, et al. (2012). "Phase I/II trial of subcutaneous interleukin-2, granulocyte-macrophage colony-stimulating factor and interferon-alpha in patients with metastatic renal cell carcinoma." BJU Int **109**(1): 63-69.

OBJECTIVE: To determine, in a phase I/II trial, the maximum tolerated dose (MTD), clinical activity and safety of concurrent subcutaneous (s.c.) interleukin-2 (IL-2), interferon-alpha2b (IFN-alpha) and granulocyte-macrophage colony-stimulating factor (GM-CSF). PATIENTS AND METHODS: Patients with metastatic renal cell carcinoma (RCC) received on a 3+3 trial design escalating doses of s.c. GM-CSF, IL-2 and IFN-alpha. Dose-limiting toxicities (DLTs) during the first 6-week cycle were used to determine the MTD. A phase II trial was then initiated to determine clinical activity. RESULTS: A total of sixty patients were enrolled in the study (phase I = 31; phase II = 29). Two DLTs were observed (G3 nausea/vomiting and fatigue) and the MTD was determined to be GM-CSF 5.0 microg/kg/day, IL-2 9.0 mIU/m(2)/day and IFN-alpha 5.0 mU/m(2)/day. Patients received a median (range) of four (one to 11) cycles of therapy. G3 adverse events were reported in 10 of 31 (32%) patients. The overall response rate was 20% (one complete response and 11 partial responses), including patients who were rendered free of disease with surgery. The median progression-free survival and overall survival were 6.0 and 23.4 months, respectively. CONCLUSIONS: Immunotherapy with concurrent s.c. GM-CSF, IL-2 and IFN-alpha is generally well tolerated. The overall response rate observed with this combination continues to show the efficacy of immunotherapy in a selected group of metastatic RCC patients.

49 [212]. George, S., T. E. Hutson, et al. (2008). "Phase I trial of PEG-interferon and recombinant IL-2 in patients with metastatic renal cell carcinoma." Cancer Chemother Pharmacol **62**(2): 347-354.

PURPOSE: Pegylated interferon alpha-2b (PEG-Intron) is a conjugate of polyethylene glycol (PEG) and interferon alpha-2b, has a prolonged half-life, and an increased area under the curve (AUC) for interferon alpha-2b. The combination of PEG-Intron with recombinant interleukin-2 (rIL-2) was investigated in a phase 1 trial. To determine the maximal tolerable dose (MTD) and preliminary efficacy of concurrent subcutaneous (SC) administration of PEG-Intron and rIL-2 in patients with metastatic renal cell carcinoma (RCC). METHODS: Cohorts of 3-6 patients received escalating doses of PEG-Intron (I-1.5, II- 1.5, III-3.0, IV-3.0, V-4.5 microg/kg SC) given weekly in combination with rIL-2 administered three times weekly (TIW) for 6 weeks. rIL-2 dose levels were escalated in weeks 1 and 4 (I-10.0, II-15.0, III-15.0, IV-20.0, V-20.0 MIU/m(2) SC), and 5.0 MIU/m(2) SC TIW was administered during weeks 2, 3, 5 and 6. RESULTS: Thirty-four patients (24 men; 10 women) were accrued at dose levels I (n = 4), II (n = 4), III (n = 6), IV (n = 14), and V (n = 6) between October 2000 and October 2002. All but one patient had prior nephrectomy (n = 33) and all but one patient (97%) had received no prior systemic therapy. Patients received a median of four cycles of treatment (range 1-9). Dose limiting toxicity occurred at dose level V and included grade 4 neutropenia and hypoxemia. A partial response was found in 5 pts (15%). Median progression-free and overall survival were 9.0 (95% C.I. 5.6-13.1 months) and 31.9 months (95% C.I. 17.2-61.9 months), respectively. CONCLUSION: The combination of PEG-Interferon and SC rIL-2 can be administered with acceptable toxicity.

50 [128]. Gigante, M., G. Li, et al. (2012). "Prognostic value of serum CA9 in patients with metastatic clear cell renal cell carcinoma under targeted therapy." Anticancer Res **32**(12): 5447-5451.

AIM: Carbonic anhydrase 9 (CA9) has been found to be one of most powerful biomarkers for clear-cell renal cell carcinoma (RCC). The serum CA9 is detectable. The aim of this study was to evaluate the potential prognostic role of serum CA9 in patients with metastatic clear-cell RCC patients under targeted therapy. PATIENTS AND METHODS: Serum samples came from the randomized phase 2 TORAVA trial. All patients received a targeted therapy (arm A designed as experimental group: temsirolimus and bevacizumab combination; arm B: sunitinib; arm C: interferon-alfa and bevacizumab). Seventy cases of metastatic clear-cell RCC were analyzed. There were 49 males and 21 females. The age ranged from 33.5 to 79.1 years with a median of 61.2 years. Serum samples were collected before treatment. Serum CA9 was quantified by enzyme-linked immunosorbent assay (ELISA). The correlation of the serum CA9 levels with the clinical parameters, treatment response and overall survival was analyzed. Overall survival estimates were calculated using the Kaplan-Meier method and compared by the log-rank test. RESULTS: Serum concentrations of CA9 ranged between 0 and 897.3 pg/ml, with an average of 94.4+/-176.6 pg/ml. There was no association between serum CA9 and clinical parameters such as Eastern Cooperative Oncology Group (ECOG) Performance Status (p=0.367) or Motzer classification (p=0.431). The serum CA9 levels were lower in the response group (64.7+/-104.7 pg/ml) than the no-response group (108.2+/-203.8 pg/ml), but the difference was not statisticlly significant (p=0.366). For the patient group overall, the Kaplan-Meier survival curve showed that high serum CA9 levels were significantly associated with shorter overall survival (hazard ratio=2.65, 95% confidence interval=1.19-5.92, log-rank test p=0.0136). For the major group of patients treated with temsirolimus and bevacizumab, the Kaplan-Meier survival curve showed that high serum CA9 levels were significantly associated with shorter overall survival (p=0.0006). CONCLUSION: Serum CA9 levels may be of clinical interest to predict the outcome for patients under targeted therapy for metastatic clear-cell RCC. CA9 may be used to select patients with metastatic clear cell RCC for clinical trials.

51 [218]. Gollob, J. A., W. K. Rathmell, et al. (2007). "Phase II trial of sorafenib plus interferon alfa-2b as first- or second-line therapy in patients with metastatic renal cell cancer." J Clin Oncol **25**(22): 3288-3295.

PURPOSE: We undertook this study to determine the activity and tolerability of sorafenib administered with interferon alfa-2b (IFN-alpha-2b) as first- or second-line therapy in metastatic renal cell cancer (RCC). PATIENTS AND METHODS: Between November 2004 and October 2006, 40 patients at two sites were enrolled onto a phase II trial of sorafenib plus IFN-alpha-2b. Treatment consisted of 8-week cycles of sorafenib 400 mg orally bid plus IFN-alpha-2b 10 million U subcutaneously three times a week followed by a 2-week break. Patients were eligible to receive additional cycles of therapy until disease progression. Dose reduction of both drugs by 50% was permitted once for toxicity. RESULTS: The response rate was 33% (95% CI, 19% to 49%; 13 of 40 patients), including 28% partial responses (n = 11) and 5% complete responses (n = 2). Responses were seen in treatment-naive and interleukin-2 (IL-2) -treated patients within the first two cycles. The median duration of response was 12 months. With a median follow-up time of 14 months, median progression-free survival time was 10 months (95% CI, 8 to 18 months), and median overall survival time has not yet been reached. Fatigue, anorexia, anemia, diarrhea, hypophosphatemia, rash, nausea, and weight loss were the most common toxicities. Grade 3 toxicities were uncommon but included hypophosphatemia, neutropenia, rash, fatigue, and anemia. Dose reductions were required in 65% of patients. CONCLUSION: The combination of sorafenib and IFN-alpha-2b has substantial activity in treatment-naive and IL-2-treated patients with RCC. The toxicity exceeded that of either drug alone, but dose reductions and breaks between cycles allowed for chronic therapy. A larger, randomized trial would determine whether there is any advantage to this regimen compared with sorafenib alone.

52 [137]. Groenewegen, G., M. Walraven, et al. (2012). "Targeting the endothelin axis with atrasentan, in combination with IFN-alpha, in metastatic renal cell carcinoma." Br J Cancer **106**(2): 284-289.

BACKGROUND: The endothelin system is involved in tumour growth. Atrasentan, a selective endothelin-A-receptor antagonist, blocks endothelin signalling. This phase I trial studied combining treatment of interferon-alpha (IFN-alpha) with atrasentan in renal cell carcinoma (RCC). PATIENTS AND METHODS: This study evaluated the safety and tolerance of IFN-alpha (9MU subcutaneously (s.c.) three times a week) in combination with atrasentan (2.5, 5 and 10 mg orally once daily) in untreated metastatic RCC. Cohort 10 mg was extended to obtain insights in efficacy and pharmacodynamics. RESULTS: Observed toxicities mainly consisted of known IFN-like toxicities (anorexia, chills, fever, fatigue and nausea), and of nasal congestion (associated to atrasentan). None of these toxicities were considered dose limiting. Cohort 10 mg was extended up to 32 patients; in a subset of patients treated according to the protocol (n=27), median overall survival (OS) was 17.3 months. One patient (3.1%) showed a partial response lasting 14.3 months. In an exploratory analysis, we observed that in the subset of patients with declining vascular endothelial growth factor (VEGF) levels (in combination with rising Endothelin-1 levels), median OS was 22.2 months compared with 2.2 months in patients with increasing VEGF levels. CONCLUSION: Combination treatment of IFN-alpha 9MU-alpha s.c. three times a week and atrasentan 10 mg once daily is tolerated. Clinical activity, especially OS, and biomarkers in our view warrant further studies targeting the endothelin axis.

53 [140]. Grunwald, V., C. Seidel, et al. (2011). "Treatment of everolimus-resistant metastatic renal cell carcinoma with VEGF-targeted therapies." Br J Cancer **105**(11): 1635-1639.

BACKGROUND: Treatment of everolimus-resistant disease remains largely undefined in metastatic renal cell carcinoma (mRCC). We report on 40 patients (pts) who receive systemic treatment after failure of everolimus. PATIENTS AND METHODS: Forty pts received sunitinib (n=19), sorafenib (n=8), dovitinib (n=10) or bevacizumab/interferon (n=3) after failure of everolimus. Median progression-free survival (PFS), overall survival (OS) and best tumour response (according to Response Evaluation Criteria In Solid Tumors) were analysed retrospectively. Kaplan-Meier, log-rank test and Cox regression analyses were used to estimate or predict OS and PFS. RESULTS: Treatment of everolimus-resistant disease was associated with a PFS of 5.5 months. (range 0.4-22.3) and an objective partial remission (PR) in 4 pts (10%) and stable disease (SD) in 22 pts (55%). In univariate analyses, first-line treatment with sorafenib was the only variable to correlate with a prolonged PFS of treatment in everolimus-resistant disease (P=0.036). However, its significance as a predictive marker for subsequent therapy could not be verified in multivariate analyses. CONCLUSIONS: Vascular endothelial growth factor targeted therapy shows promising activity in everolimus-resistant metastatic renal cancer and warrants further studies.

54 [117]. Halabi, S., B. Rini, et al. (2014). "Progression-free survival as a surrogate endpoint of overall survival in patients with metastatic renal cell carcinoma." Cancer **120**(1): 52-60.

BACKGROUND: The current study was conducted to investigate the dependence between progression-free survival (PFS) and overall survival (OS) in patients with metastatic renal cell carcinoma (mRCC) and to explore whether PFS can be used as an intermediate endpoint of OS in this patient population. METHODS: A total of 1381 patients from 2 prospective phase 3 trials (Cancer and Leukemia Group B [CALGB] 90206 and AVOREN) of interferon-alpha with or without bevacizumab were analyzed. Both trials recruited previously untreated patients with clear cell mRCC with an Eastern Cooperative Oncology Group performance status of 0 to 2; adequate bone marrow, hepatic, cardiac, and renal function; and controlled blood pressure. The CALGB study served as the training data set, and the AVOREN study served as the testing data set. The dependence between PFS and OS was investigated using the Kendall tau for bivariate time-to-event endpoints. RESULTS: In the training data set, the median OS times among patients who experienced progressive disease at 3 months or 6 months were 6 months and 8 months, respectively, compared with 25 months and 30 months, respectively, (P < .001) in patients who did not develop disease progress. The adjusted hazard ratios (HR) were 2.6 (P < .0001) and 2.8 (P < .0001), respectively, for patients who did and did not progress at 3 months or 6 months. The dependence between PFS and OS was 0.53. These associations were confirmed in the testing data set. CONCLUSIONS: In patients with mRCC who were treated with interferon-alpha with or without bevacizumab, the PFS at 3 months and 6 months was found to be predictive of OS. A high dependence between PFS and OS was observed, suggesting that PFS may be used as a surrogate endpoint for OS. Although this is a novel observation for RCC, these findings require validation in patients with mRCC who are treated with other targeted agents.

55 [119]. Harmon, C. S., S. E. DePrimo, et al. (2014). "Circulating proteins as potential biomarkers of sunitinib and interferon-alpha efficacy in treatment-naive patients with metastatic renal cell carcinoma." Cancer Chemother Pharmacol **73**(1): 151-161.

PURPOSE: We investigated potential biomarkers of efficacy in a phase III trial of sunitinib versus interferon-alpha (IFN-alpha), first-line in metastatic renal cell carcinoma (mRCC), by analyzing plasma levels of vascular endothelial growth factor (VEGF)-A, VEGF-C, soluble VEGF receptor-3 (sVEGFR-3) and interleukin (IL)-8. METHODS: Seven hundred and fifty mRCC patients were randomized to oral sunitinib 50 mg/day in repeated cycles of a 4-week on/2-week off schedule or IFN-alpha 9 million units subcutaneously thrice weekly. Plasma samples collected from a subset of 63 patients on days 1 and 28 of cycles 1-4 and at end of treatment were analyzed by ELISA. RESULTS: Baseline characteristics of biomarker-evaluated patients in sunitinib (N = 33) and IFN-alpha (N = 30) arms were comparable to their respective intent-to-treat populations. By univariate Cox regression analysis, low baseline soluble protein levels were associated with lower risk of progression/death (all P < 0.05): in both treatment arms, baseline VEGF-A and IL-8 were associated with overall survival (OS) and baseline VEGF-C with progression-free survival (PFS); in the sunitinib arm, baseline VEGF-A was associated with PFS and baseline sVEGFR-3 with PFS and OS; in the IFN-alpha arm, baseline IL-8 was associated with PFS. In multivariate analysis, baseline sVEGFR-3 and IL-8 remained independent predictors of OS in the sunitinib arm, while no independent predictors of outcome remained in the IFN-alpha arm. Pharmacodynamic changes were not associated with PFS or OS for any plasma protein investigated. CONCLUSIONS: Our findings suggest that, in mRCC, baseline VEGF-A and IL-8 may have prognostic value, while baseline sVEGFR-3 may predict sunitinib efficacy.

56 [151]. Harrop, R., W. H. Shingler, et al. (2011). "MVA-5T4-induced immune responses are an early marker of efficacy in renal cancer patients." Cancer Immunol Immunother **60**(6): 829-837.

Few immunotherapy compounds have demonstrated a direct link between the predicted mode of action of the product and benefit to the patient. Since cancer vaccines are thought to have a delayed therapeutic effect, identification of the active moiety may enable the development of an early marker of efficacy. Patients with renal cancer and requiring first-line treatment for metastatic disease were randomized 1:1 to receive MVA-5T4 (TroVax((R))) or placebo alongside Sunitinib, IL-2 or IFN-alpha in a multicentre phase III trial. Antibody responses were quantified following the 3rd and 4th vaccinations. A surrogate for 5T4 antibody response (the immune response surrogate; IRS) was constructed and then used in a survival analysis to evaluate treatment benefit. Seven hundred and thirty-three patients were randomized, and immune responses were assessed in 590 patients. A high 5T4 antibody response was associated with longer survival within the MVA-5T4-treated group. The IRS was constructed as a linear combination of pre-treatment 5T4 antibody levels, hemoglobin and hematocrit and was shown to be a significant predictor of treatment benefit in the phase III study. Importantly, the IRS was also associated with antibody response and survival in an independent dataset comprising renal, colorectal and prostate cancer patients treated with MVA-5T4 in phase I-II studies. The derivation of the IRS formed part of an exploratory, retrospective analysis; however, if confirmed in future studies, the results have important implications for the development and use of the MVA-5T4 vaccine and potentially for other similar vaccines.

57 [132]. Harrop, R., P. Treasure, et al. (2012). "Analysis of pre-treatment markers predictive of treatment benefit for the therapeutic cancer vaccine MVA-5T4 (TroVax)." Cancer Immunol Immunother **61**(12): 2283-2294.

Cancer vaccines such as MVA-5T4 (TroVax((R))) must induce an efficacious immune response to deliver therapeutic benefit. The identification of biomarkers that impact on the clinical and/or immunological efficacy of cancer vaccines is required in order to select patients who are most likely to benefit from this treatment modality. Here, we sought to identify a predictor of treatment benefit for renal cancer patients treated with MVA-5T4. Statistical modeling was undertaken using data from a phase III trial in which patients requiring first-line treatment for metastatic renal cell carcinoma were randomized 1:1 to receive MVA-5T4 or placebo alongside sunitinib, IL-2 or IFN-alpha. Numerous pre-treatment factors associated with inflammatory anemia (e.g., CRP, hemoglobin, hematocrit, IL-6, ferritin, platelets) demonstrated a significant relationship with tumor burden and patient survival. From these prognostic factors, the pre-treatment mean corpuscular hemoglobin concentration (MCHC) was found to be the best predictor of treatment benefit (P < 0.01) for MVA-5T4 treated patients and also correlated positively with tumor shrinkage (P < 0.001). Furthermore, MCHC levels showed a significant positive association with 5T4 antibody response (P = 0.01). The latter result was confirmed using an independent data set comprising phase II trials of MVA-5T4 in patients with colorectal, renal and prostate cancers. Retrospective analyses demonstrated that RCC patients who had very large tumor burdens and low MCHC levels received little or no benefit from treatment with MVA-5T4; however, patients with smaller tumor burdens and normal MCHC levels received substantial benefit from treatment with MVA-5T4.

58 [182]. Hawkins, R. E., C. Macdermott, et al. (2009). "Vaccination of patients with metastatic renal cancer with modified vaccinia Ankara encoding the tumor antigen 5T4 (TroVax) given alongside interferon-alpha." J Immunother **32**(4): 424-429.

Approximately 90% of renal cell tumors overexpress the tumor antigen 5T4. The attenuated strain of vaccinia virus, modified vaccinia Ankara, has been engineered to express 5T4 (TroVax). We conducted an open-label phase 1/2 trial in which TroVax was administered alongside interferon-alpha (IFNalpha) to 11 patients with metastatic renal cell carcinoma. Antigen-specific cellular and humoral responses were monitored throughout the study, and clinical responses were assessed by measuring the changes in tumor burden by computed tomography scan (Response Evaluation Criteria In Solid Tumors). The primary objective was to assess the safety, immunogenicity, and efficacy of TroVax when given alongside IFNalpha. Treatment with TroVax plus IFNalpha was well tolerated with no serious adverse events attributed to TroVax. All 11 patients mounted 5T4-specific antibody responses and 5 (45%) mounted cellular responses. No objective tumor responses were seen, but the overall median time to progression (TTP) of 9 months (range: 2.1 to 26+ mo) was longer than expected for IFNalpha alone. For the 10 clear cell patients the TTP ranged from 3.9 to 26+ months, with a median TTP of 10.4 months. The high frequency of 5T4-specific immune responses and prolonged median TTP for clear cell patients compared with that expected for IFNalpha alone is encouraging and warrants further investigation.

59 [139]. Hinotsu, S., K. Kawai, et al. (2013). "Randomized controlled study of natural interferon alpha as adjuvant treatment for stage II or III renal cell carcinoma." Int J Clin Oncol **18**(1): 68-74.

BACKGROUND: The prophylactic effect of postoperative interferon on recurrence and distant metastasis in stage II or III renal cell carcinoma is unclear. In most studies, interferon has been administered for 6 months or less. Therefore, we performed a clinical study of the efficacy of 1-year postoperative administration of natural interferon alpha, which is generally used in Japan. METHODS: The subjects were patients diagnosed with stage II or III renal cell carcinoma who underwent radical nephrectomy. The subjects were randomly allocated to receive an intramuscular injection of natural interferon alpha (3 million to 6 million units) 3 times a week for 1 year or to receive follow-up observation until recurrence or metastasis occurred. Chest and abdominal CT were performed once yearly for all patients. The primary endpoint was progression-free survival. RESULTS: From September 2001 to August 2006, a total of 107 patients were registered, but 7 subsequently withdrew from the study. Therefore, 100 patients were included in the analysis. The primary endpoint of progression-free survival did not differ significantly between the groups that received natural interferon alpha or follow-up observation (p = 0.456, log-rank test). However, peak hazards of progression in the interferon group were delayed for about 6-10 months compared with the observation group. CONCLUSION: Progression-free survival showed no improvement after administration of natural interferon alpha to patients with stage II or III renal cell carcinoma for 1 year after radical nephrectomy. The peak hazards of progression might be delayed by about 6 months by interferon administration.

60 [170]. Hoyle, M., C. Green, et al. (2010). "Cost-effectiveness of temsirolimus for first line treatment of advanced renal cell carcinoma." Value Health **13**(1): 61-68.

OBJECTIVES: To estimate the cost-effectiveness of temsirolimus compared to interferon-alpha for first line treatment of patients with advanced, poor prognosis renal cell carcinoma, from the perspective of the UK National Health Service. METHODS: A decision-analytic model was developed to estimate the cost-effectiveness of temsirolimus. The clinical effectiveness of temsirolimus compared with interferon-alpha and the utility values (using EQ-5D tariffs) were taken from a recent phase III randomized clinical trial. Cost data were obtained from published literature and based on current UK practice. The effect of parameter uncertainty on cost-effectiveness was explored through extensive one-way and probabilistic sensitivity analyses. RESULTS: Compared to interferon-alpha, temsirolimus treatment resulted in an incremental cost per QALY gained of pound94,632; based on an estimated mean gain of 0.24 quality-adjusted life years (QALYs) per patient, at a mean additional cost of pound22,331 (inflated to 2007/8). The cost per QALY for patient subgroups ranged from pound74,369 to pound154,752. The probability that temsirolimus is cost-effective compared to interferon-alpha at a willingness to pay threshold of pound30,000 per QALY for all patient groups is expected to be close to zero. The cost per QALY was sensitive to the clinical effectiveness parameters, health state utilities, drug costs and the cost of administration of temsirolimus. CONCLUSIONS: Temsirolimus has been shown to be clinically effective compared to interferon-alpha offering additional health benefits, however, with a cost per QALY in excess of pound90,000, it may not be regarded as a cost-effective use of resources in some health care settings.

61 [120]. Huang, T., X. S. Song, et al. (2013). "[Efficacies of sorafenib plus interferon in advanced renal cell carcinoma: a report of 57 cases]." Zhonghua Yi Xue Za Zhi **93**(24): 1903-1905.

OBJECTIVE: To explore the efficacies and adverse events of sorafenib in the treatment of advanced metastatic renal cell carcinoma. METHODS: A total of 57 patients with advanced kidney cancer were recruited from our hospital from April 2007 to October 2011. They were divided into sorafenib group (A, n = 24) and sorafenib + IFN group (B, n = 33). The primary endpoints included objective response rate and progression-free survival (PFS). And the secondary endpoints were overall survival (OS) and incidence of adverse events. RESULTS: The mean medication time of group A was 15 (7-56) months. The outcomes were partial response (PR, n = 1), stable disease (SD, n = 8), progressive disease (PD, n = 1) and death (n = 14). The rates of objective response and disease control were 4.2% (1/24) and 37.5% (9/24) respectively. For group B, the mean medication time was 15 (4-30) months. The outcomes were PR (n = 2), and include 2 patients of PR, 21 examples of SD, 1 patient of PD and death. The rates of objective response and disease control were 6.1% (2/33) and 69.7% (23/33) respectively. Two groups had no significant difference in incidence or severity of adverse events (both P > 0.05). CONCLUSIONS: As a safe and effective agent for advanced kidney cancer, sorafenib is well-tolerated in patients. The combined use of interferon may improve the therapeutic efficacies without an occurrence of adverse events.

62 [219]. Hudes, G., M. Carducci, et al. (2007). "Temsirolimus, interferon alfa, or both for advanced renal-cell carcinoma." N Engl J Med **356**(22): 2271-2281.

BACKGROUND: Interferon alfa is widely used for metastatic renal-cell carcinoma but has limited efficacy and tolerability. Temsirolimus, a specific inhibitor of the mammalian target of rapamycin kinase, may benefit patients with this disease. METHODS: In this multicenter, phase 3 trial, we randomly assigned 626 patients with previously untreated, poor-prognosis metastatic renal-cell carcinoma to receive 25 mg of intravenous temsirolimus weekly, 3 million U of interferon alfa (with an increase to 18 million U) subcutaneously three times weekly, or combination therapy with 15 mg of temsirolimus weekly plus 6 million U of interferon alfa three times weekly. The primary end point was overall survival in comparisons of the temsirolimus group and the combination-therapy group with the interferon group. RESULTS: Patients who received temsirolimus alone had longer overall survival (hazard ratio for death, 0.73; 95% confidence interval [CI], 0.58 to 0.92; P=0.008) and progression-free survival (P<0.001) than did patients who received interferon alone. Overall survival in the combination-therapy group did not differ significantly from that in the interferon group (hazard ratio, 0.96; 95% CI, 0.76 to 1.20; P=0.70). Median overall survival times in the interferon group, the temsirolimus group, and the combination-therapy group were 7.3, 10.9, and 8.4 months, respectively. Rash, peripheral edema, hyperglycemia, and hyperlipidemia were more common in the temsirolimus group, whereas asthenia was more common in the interferon group. There were fewer patients with serious adverse events in the temsirolimus group than in the interferon group (P=0.02). CONCLUSIONS: As compared with interferon alfa, temsirolimus improved overall survival among patients with metastatic renal-cell carcinoma and a poor prognosis. The addition of temsirolimus to interferon did not improve survival. (ClinicalTrials.gov number, NCT00065468 [ClinicalTrials.gov].).

63 [241]. Huie, M., K. Oettel, et al. (2006). "Phase II study of interferon-alpha and doxycycline for advanced renal cell carcinoma." Invest New Drugs **24**(3): 255-260.

OBJECTIVE: To assess the efficacy and toxicity of the combination of interferon-alpha and doxycycline in patients with metastatic renal cell carcinoma and to assess the effect of this treatment on serum vascular endothelial growth factor (VEGF) levels. PATIENTS AND METHODS: Seventeen patients with Eastern Cooperative Oncology Group (ECOG) performance status of 0 or 1 and life expectancy greater than 4 months with radiologically evident advanced renal cell carcinoma were enrolled. Eight patients had prior nephrectomy and 10 patients were treated within 4 months of their diagnosis. Treatment consisted of interferon-alpha up to 9 million units subcutaneously three times per week and doxycycline 300 mg orally twice per day for weeks one and three of each four-week cycle. Toxicity was evaluated on a biweekly basis and response on a bimonthly basis. VEGF plasma levels were assessed monthly as a measure of potential antiangiogenic effect. RESULTS: No objective responses were seen. The mean duration of study was 2.6 cycles (range: 0.8-6.0 cycles). Three patients (17%) tolerated therapy and displayed stable disease for greater than four months. Five patients withdrew from study before the first response evaluation. Ten patients experienced grade 2 gastrointestinal toxicity requiring dose reduction of doxycycline. Eight patients experienced grade 2 fatigue requiring dose reduction of interferon. VEGF plasma levels were initially suppressed in patients who demonstrated progressive disease but not in patients with stable disease. CONCLUSION: This regimen of doxycycline and interferon-alpha was not efficacious as treatment for renal cell carcinoma. Plasma VEGF levels were significantly decreased during the first two cycles of treatment, but this does not correlate with clinical outcome.

64 [123]. Iacovelli, R., D. Alesini, et al. (2014). "Targeted therapies and complete responses in first line treatment of metastatic renal cell carcinoma. A meta-analysis of published trials." Cancer Treat Rev **40**(2): 271-275.

Antiangiogenic agents (AAs) have reported grater efficacy compared to interferon. Despite these advances, radiological complete response to therapy is rare. We meta-analyzed the incidence of complete response in patients treated with AAs and in controls in main randomized clinical trials for first-line therapy in metastatic renal cell carcinoma. PubMed was reviewed for phase II-III randomized clinical trials with AAs vs. non-AAs in patients with good or intermediate prognosis. We calculated the relative risk of events in patients assigned to AAs compared to control. Five RCTs were found; four were phase III and one was phase II. A total of 2747 patients was valuable for final analysis and randomized to receive AAs or control. Patients in the control-group had interferon (85%) or placebo (15%); patients in the AAs-group received bevacizumab (48%), sunitinib (26%), pazopanib (20%) or sorafenib (6%). The incidence of complete response in patients treated with AAs was 2.0% (95% CI, 1.2-2.8) compared to 1.4% (95% CI, 0.7-2.1) in the control arm. Comparing the different type of AAs, the incidence of complete response was 2.5% (95% CI, 1.2-3.8) in the bevacizumab group and 1.6% (95% CI, 0.1-2.5) in the TKIs group. The relative risk to have a complete response was 1.52 (95% CI, 0.85-2.73; p=0.16) in patients treated with AAs compared to controls; this was found higher in patients treated with TKIs compared to bevacizumab. The complete response is a rare event in metastatic kidney tumor, even if AAs reported greater efficacy in terms of progression-free survival and of overall response rate, they did not increase the curative rate of metastatic disease. Probably, some biologic factors other than angiogenesis may influence the complete response in this disease.

65 [208]. Irani, J. (2007). "[Sunitinib versus interferon-alpha in metastatic renal-cell carcinoma]." Prog Urol **17**(5): 996.

66 [168]. Jonasch, E., P. Corn, et al. (2010). "Upfront, randomized, phase 2 trial of sorafenib versus sorafenib and low-dose interferon alfa in patients with advanced renal cell carcinoma: clinical and biomarker analysis." Cancer **116**(1): 57-65.

BACKGROUND: The objective of this study was to independently evaluate the objective response rate of sorafenib and sorafenib plus low-dose interferon-alfa 2b (IFN) as frontline therapy in patients with metastatic renal cell carcinoma (mRCC). METHODS: Untreated patients with clear cell mRCC were randomized to receive sorafenib 400 mg orally twice daily or sorafenib 400 mg orally twice daily plus subcutaneous IFN 0.5 million U (MU) twice daily. Primary endpoints included the objective response rate (ORR) and safety. Secondary endpoints included progression-free survival (PFS) and overall survival (OS). Exploratory endpoints included the predictive value of tumor tissue biomarkers. RESULTS: Eighty patients were enrolled. The median follow-up was 19.7 months (range, 0-34.2 months). The ORR was 30% (95% confidence interval [CI], 16.6%-46.5%) in the sorafenib arm and 25% (95% CI, 12.7%-41.2%) in the combination arm. The median PFS was 7.39 months in the sorafenib-alone arm (95% CI, 5.52-9.20 months) and 7.56 months in the sorafenib plus IFN arm (95% CI, 5.19-11.07 months). The median OS was 27.04 months in the combination arm (95% CI, from 22.31 to not attained) and was not reached in the sorafenib arm. Toxicities were comparable in both arms. In a multivariate model, increased phosphorylated protein kinase B (pAKT) levels were associated with poorer PFS (hazard ratio, 1.04; 95% CI, 1.00-1.08; P = .0411) and OS (hazard ratio, 1.15; 95% CI, 1.02-1.29; P = .0173). CONCLUSIONS: The addition of low-dose IFN to sorafenib resulted in efficacy outcomes that were comparable to those achieved with sorafenib monotherapy. The current results indicated that pAKT levels may predict for clinical outcome, but further mechanistic study is required.

67 [236]. Kinouchi, T., J. Sakamoto, et al. (2006). "Prospective randomized trial of natural interferon-alpha versus natural interferon-alpha plus cimetidine in advanced renal cell carcinoma with pulmonary metastasis." J Cancer Res Clin Oncol **132**(8): 499-504.

PURPOSE: In a preliminary non-randomized study, combination therapy with natural (i.e. non-recombinant) interferon-alpha plus cimetidine obtained a high response rate in patients with advanced renal cell carcinoma. We conducted a prospective randomized phase III trial to determine whether combination therapy with natural interferon-alpha plus cimetidine is superior to natural interferon-alpha alone in patients with advanced renal cell carcinoma with pulmonary metastasis. METHODS: Patients received 5 million units (MU) natural interferon-alpha per day, five times a week, or the 5 MU natural interferon-alpha regimen plus a daily oral cimetidine. The primary and secondary end points were the response rate, and the time to progression (TTP), respectively. RESULTS: Between April 1998 and March 2002, 71 patients from 32 institutions were randomly assigned to the 2 treatment groups. One patient in each group did not receive any natural interferon-alpha whatsoever. Two patients in the natural interferon-alpha alone group stopped treatment: on day 9 and on day 10, respectively. In the intent-to-treat analysis, 1 complete response (CR), 4 partial responses (PRs), 16 no changes (NCs), and 12 progressive diseases (PDs) were observed among the 36 patients in the interferon-alpha alone group with a response rate of 13.9%. Of the 35 patients in the natural interferon-alpha plus cimetidine group, there were two CRs, 8 PRs, 13 NCs, and 11 PDs, yielding a response rate of 28.6% (P=0.13). TTP ranged from 9 to 845 days (median 112 days) in the natural interferon-alpha-alone group, and from 31 to 1,568 days (median 125 days) in the natural interferon-alpha plus cimetidine group (P=0.87). CONCLUSIONS: Combined treatment with natural interferon-alpha plus cimetidine for advanced renal cell carcinoma did not result in a significant improvement in response rates or TTP compared to natural interferon-alpha therapy alone.

68 [200]. Klatte, T., A. Ittenson, et al. (2008). "Pretreatment with interferon-alpha2a modulates perioperative immunodysfunction in patients with renal cell carcinoma." Onkologie **31**(1-2): 28-34.

INTRODUCTION: Complex perioperative immunodysfunction occurs in patients with renal cell carcinoma undergoing surgery. Here, we report on the effect of preoperative treatment with interferon-alpha2a (IFN-alpha2a). MATERIALS AND METHODS: 30 patients with a renal tumour received preoperative IFN-alpha2a for 6 days beginning 1 week before nephrectomy, 30 did not. Parameters of cellular and humoral immunity were measured in venous blood at various intervals using flow cytometry and ELISA. Endpoints included effects on immune parameters, toxicity, and survival. RESULTS: Toxicity was grade 1 in 52%, 2 in 30%, and 3 in 4%. During IFN-alpha2a administration, leukocytes, monocytes, granulocytes, B-cell marker CD19, activation markers, CD4+CD25+ regulatory T-cells, and vascular endothelial growth factor (VEGF) dropped significantly, but no difference was observed in T-cell and natural killer (NK)-cell markers, and IL-10. Postoperatively, T-cell and activation markers decreased in both groups, but CD4, CD28, IL-6, IL-10, and HLA-DR alterations were significantly less accentuated in patients who had been treated with IFN-alpha2a. After a median follow-up of 23 months, survival did not differ between the groups (p = 0.54). CONCLUSIONS: Perioperative immunodysfunction can be modulated by preoperative administration of IFN- alpha2a. IFN-alpha2a decreased the level of VEGF and CD4+CD25+ regulatory T-cells implicating a potential combination with tyrosine kinase inhibitors and vaccines.

69 [163]. Kwitkowski, V. E., T. M. Prowell, et al. (2010). "FDA approval summary: temsirolimus as treatment for advanced renal cell carcinoma." Oncologist **15**(4): 428-435.

This report summarizes the U.S. Food and Drug Administration (FDA)'s approval of temsirolimus (Torisel), on May 30, 2007, for the treatment of advanced renal cell carcinoma (RCC). Information provided includes regulatory history, study design, study results, and literature review. A multicenter, three-arm, randomized, open-label study was conducted in previously untreated patients with poor-prognosis, advanced RCC. The study objectives were to compare overall survival (OS), progression-free survival (PFS), objective response rate, and safety in patients receiving interferon (IFN)-alpha versus those receiving temsirolimus alone or in combination with IFN-alpha. In the second planned interim analysis of the intent-to-treat population (n = 626), there was a statistically significant longer OS time in the temsirolimus (25 mg) arm than in the IFN-alpha arm (median, 10.9 months versus 7.3 months; hazard ratio [HR], 0.73; p = .0078). The combination of temsirolimus (15 mg) and IFN-alpha did not lead to a significant difference in OS compared with IFN-alpha alone. There was also a statistically significant longer PFS time for the temsirolimus (25 mg) arm than for the IFN-alpha arm (median, 5.5 months versus 3.1 months; HR, 0.66, p = .0001). Common adverse reactions reported in patients receiving temsirolimus were rash, asthenia, and mucositis. Common laboratory abnormalities were anemia, hyperglycemia, hyperlipidemia, and hypertriglyceridemia. Serious but rare cases of interstitial lung disease, bowel perforation, and acute renal failure were observed. Temsirolimus has demonstrated superiority in terms of OS and PFS over IFN-alpha and provides an additional treatment option for patients with advanced RCC.

70 [188]. Lara, P. N., Jr., C. M. Tangen, et al. (2009). "Predictors of survival of advanced renal cell carcinoma: long-term results from Southwest Oncology Group Trial S8949." J Urol **181**(2): 512-516; discussion 516-517.

PURPOSE: S8949 demonstrated improved overall survival for debulking nephrectomy in interferon treated patients with advanced renal cell carcinoma. We present an updated analysis of S8949, now with a median followup of 9 years. We explored clinical predictors of overall survival. MATERIALS AND METHODS: Univariate and multivariate Cox regression analysis was performed to evaluate the impact of clinical variables potentially influencing survival. RESULTS: Of 246 patients 241 were eligible and randomized to interferon with or without nephrectomy. Patients randomized to nephrectomy continued to have improved overall survival (HR 0.74, 95% CI 0.57-0.96, p = 0.022). Multivariate analysis showed that performance status 1 vs 0 (HR 1.95, p <0.0001), high alkaline phosphatase (HR 1.5, p = 0.002) and lung metastasis only (HR 0.73, p = 0.028) were overall survival predictors. There was no evidence of an interaction of performance status, measurable disease or lung metastases with nephrectomy (each p >0.30). In a patient subset that survived at least 90 days after randomization early progressive disease within 90 days was prognostic of overall survival in a multivariate model (HR 2.1, p <0.0001), as was performance status (HR 1.7, p = 0.0006). CONCLUSIONS: Nephrectomy prolonged long-term overall survival in this updated analysis, supporting its role as standard therapy in patients with advanced renal cell carcinoma. A nephrectomy benefit was seen across all prespecified patient subsets. Early progressive disease and performance status were strong predictors of overall survival. These results support efforts to identify biomarkers of renal cell carcinoma resistance to treatment and early progressive disease to facilitate rational patient selection for systemic therapy.

71 [138]. Liu, F., X. Chen, et al. (2011). "VEGF pathway-targeted therapy for advanced renal cell carcinoma: a meta-analysis of randomized controlled trials." J Huazhong Univ Sci Technolog Med Sci **31**(6): 799-806.

Immunotherapy which has been in practice for more than 20 years proves effective for the treatment of metastatic renal cell carcinoma (mRCC). Anti-angiogenesis-targeted therapy has recently been identified as a promising therapeutic strategy for mRCC. This study was aimed to evaluate the effectiveness of vascular endothelial growth factor (VEGF) pathway-targeted therapy for mRCC by comparing its effectiveness with that of immunotherapy. The electronic databases were searched. Randomized controlled trials (RCTs) on comparison of VEGF inhibiting drugs (sorafenib, sunitinib and bevacizumab) with interferon (IFN) or placebo for mRCC treatment were included. Data were pooled to meta-analyze. A total of 7 RCTs with 3451 patients were involved. The results showed that anti-VEGF agents improved progression-free survival (PFS) and offered substantial clinical benefits to patients with mRCC. Among them, sunitinib had a higher overall response rate (ORR) than IFN (47% versus 12%, P<0.000001). Bevacizumab plus IFN produced a superior PFS [risk ratio (RR): 0.86, 95% confidence interval (CI): 0.76-0.97; P=0.01] and ORR (RR: 2.19; 95% CI: 1.72-2.78; P<0.00001) in patients with mRCC over IFN, but it yielded an increase by 31% in the risk of serious toxic effects (RR: 1.31; 95% CI: 1.20-1.43; P<0.00001) as compared with IFN. The overall survival (OS) was extended by sorafenib (17.8 months) and sunitinib (26.4 months) as compared with IFN (13 months). It was concluded that compared with IFN therapy, VEGF pathway-targeted therapies improved PFS and achieved significant therapeutic benefits in mRCC. However, the risk to benefit ratio of these agents needs to be further evaluated.

72 [169]. Liu, J. Z., S. G. Chen, et al. (2009). "Effect of haishengsu as an adjunct therapy for patients with advanced renal cell cancer: a randomized and placebo-controlled clinical trial." J Altern Complement Med **15**(10): 1127-1130.

OBJECTIVE: The purpose of this study was to investigate the effect of Haishengsu, an extract from Tegillarca L. granosa, on the effects and side-effects of immunotherapy in patients with advanced renal cell cancer. METHODS: Fifty-five (55) patients with renal cell cancer were randomly divided into a Haishengsu group (n = 27, 2.4 mg, intravenously for 15 days) and a control group (n = 28). All patients were also treated with interleukin-2, interferon-alpha, and fluorouracil. RESULTS: In the Haishengsu group, the prevalence of gastrointestinal reactions to the immunotherapy was lower than in the control group (18.5% versus 64.3%, p < 0.01). In comparison with the control group, more patients from the Haishengsu group had increased food intake (74.1% versus 14.3%, p < 0.01), weight gain (77.8% versus 10.7%, p < 0.01) or an increase in Karnofsky Performance Status score (55.6% versus 17.9%, p < 0.01). The remission rate of cancer in the Haishengsu group was higher than in the control group (51.9% and 21.4%, p < 0.01). CONCLUSIONS: Addition of Haishengsu to the conventional immunotherapy is associated with an increased remission rate in patients with advanced renal cell cancer. Haishengsu was also associated with a reduced rate of gastrointestinal side-effects from the immunotherapeutic agents, and an improvement in the physical functionality of the patients.

73 [207]. Loppow, D., E. Huland, et al. (2007). "Interleukin-2 inhalation therapy temporarily induces asthma-like airway inflammation." Eur J Med Res **12**(11): 556-562.

BACKGROUND: Inhaled interleukin-2 (IL-2) is an effective and safe treatment in metastasing renal cell carcinoma (mRCC) but known to potentially elicit respiratory symptoms. OBJECTIVES: The present study analyses the effects of IL-2 using a panel of measures including markers of airway inflammation. METHODS: Ten patients with mRCC (7m/3f; mean age, 63 yrs) were measured at baseline, 6-10 days after start of therapy (n = 5, inhaled IL-2 only; n = 5, inhaled IL-2 plus 1/11th of daily dose subcutaneously), and 16-29 days later under continuous combined (inhaled plus subcutaneous) therapy, including additional subcutaneous IFN-alpha in 8 patients. RESULTS: After start of therapy median FEV1 declined from 108 to 85 to 90 % predicted and the provocative concentration of methacholine eliciting a 20 % fall in FEV1 (PC20 FEV1) from 16 to 8 to 3 mg/mL, while the level of exhaled nitric oxide (FENO) rose from 27 to 79 to 60 ppb and the percentage of sputum eosinophils from 2 to 18 to 37 % (p<0.01, each), accompanied by cough and dyspnoea (p<0.05). One patient who stopped therapy, was back to baseline values when measured 2 months later. Cytokine production by blood or sputum T lymphocytes was not markedly altered by IL-2 inhalation. CONCLUSIONS: IL-2 inhalation therapy in patients with metastasing renal cell carcinoma is capable of temporarily inducing symptomatic, functional and inflammatory alterations similar to those of bronchial asthma.

74 [183]. Lyrdal, D., U. Stierner, et al. (2009). "Metastatic renal cell carcinoma treated with Peg-interferon alfa-2b." Acta Oncol **48**(6): 901-908.

INTRODUCTION: Peginterferon has an increased plasma half-life and enables a constant exposure to interferon. This modification might increase the antiangiogenic effect of the treatment and influence the efficacy. We report the results of a phase II open-label study with Peginterferon alfa-2b (Pegintron Schering-Plough) on efficacy and tolerability in patients with advanced renal cell carcinoma (MRCC). MATERIALS AND METHODS: Twenty eight patients with MRCC were treated with Peginterferon in escalating doses of 0.5 microg/kg once weekly until 2 microg/kg was reached or prohibited toxicity occurred. Lesions were evaluated according to Response Evaluation Criteria in Solid Tumors (RECIST). RESULTS: Thirteen patients tolerated a dose of 2 microg/kg/week. At 6 months 16 patients (57%) had disease control of which four had partial response (PR) and 12 stable disease whereas 12 (43%) had progressed. PR was only seen in the lung parenchyma or mediastinum. Median time to progression (TTP) was 8 months in all patients and 13 months for PR and SD patients. Correspondingly, median survival was 19.5 months and 28 months, respectively (seven patients received second-line treatment with tyrosine kinase inhibitor). The mean dose during long-term treatment was 1.5 and at the end of treatment 1.2 microg/kg/week. Most side effects were grade 1-2 and only two patients stopped treatment for that reason. VEGF levels in serum before and during treatment did not correlate to the therapeutic response. DISCUSSION: Peginterferon was well tolerated in MRCC albeit with dose modification during long-term treatment. Response pattern seems to be the same as with nonpegylated interferon. Peginterferon may be used as monotherapy in selected patients and in trials of combinations with targeted drugs.

75 [220]. Margolin, K., T. W. Synold, et al. (2007). "Oblimersen and alpha-interferon in metastatic renal cancer: a phase II study of the California Cancer Consortium." J Cancer Res Clin Oncol **133**(10): 705-711.

PURPOSE: Oblimersen is an 18-base oligodeoxynucleotide encoding antisense to the gene for bcl-2, an anti-apoptotic protein that is upregulated in renal and other cancers. This study was designed to evaluate the combination of oblimersen with alpha-Interferon in advanced renal cancer. Trial endpoints were antitumor efficacy and toxicity, pharmacokinetics, and evidence of apoptosis in peripheral blood mononuclear cells. METHODS: Patients with measurable advanced renal cancer and 0-1 prior therapy were eligible. Treatment consisted of oblimersen, 7 mg/kg/day, as a continuous intravenous infusion 7 days of every 14 day cycle, plus alpha-IFN, 5 million units/m(2) subcutaneously, days 4 and 6 of the first oblimersen infusion, then thrice weekly. Blood for laboratory correlates was collected before treatment, during oblimersen, and during therapy with both agents. RESULTS: Twenty-three patients were enrolled, five of whom had prior systemic therapy (three with prior high-dose interleukin-2). The median number of treatment cycles was 4 (range 1-12). One patient had a partial response lasting 2.5 months. The Grade 3-4 toxicities were fatigue, fever, myelosuppression, hepatic enzyme and metabolic abnormalities. Laboratory analyses of CD3+ lymphocyte apoptotic markers demonstrated no change between pre-treatment and on-treatment levels of bcl-2 or Annexin/PI positivity by flow cytometry. Mean oblimersen steady-state plasma concentration and clearance was 2.3 +/- 0.9 microg/ml and 0.15 +/- 0.07 l/h/kg, respectively. CONCLUSIONS: Oblimersen given in this dose and schedule with alpha-IFN does not appear sufficiently active to warrant further study in advanced renal cancer. Combinations with newer targeted agents may show greater promise.

76 [250]. McDermott, D. F., M. M. Regan, et al. (2005). "Randomized phase III trial of high-dose interleukin-2 versus subcutaneous interleukin-2 and interferon in patients with metastatic renal cell carcinoma." J Clin Oncol **23**(1): 133-141.

PURPOSE: The Cytokine Working Group conducted a randomized phase III trial to determine the value of outpatient interleukin-2 (IL-2) and interferon alfa-2b (IFN) relative to high-dose (HD) IL-2 in patients with metastatic renal cell carcinoma. PATIENTS AND METHODS: Patients were stratified for bone and liver metastases, primary tumor in place, and Eastern Cooperative Oncology Group performance status 0 or 1 and then randomly assigned to receive either IL-2 (5 MIU/m(2) subcutaneously every 8 hours for three doses on day 1, then daily 5 days/wk for 4 weeks) and IFN (5 MIU/m(2) subcutaneously three times per week for 4 weeks) every 6 weeks or HD IL-2 (600,000 U/kg/dose intravenously every 8 hours on days 1 through 5 and 15 to 19 [maximum 28 doses]) every 12 weeks. RESULTS: One hundred ninety-two patients were enrolled between April 1997 and July 2000. Toxicities were as anticipated for these regimens. The response rate was 23.2% (22 of 95 patients) for HD IL-2 versus 9.9% (nine of 91 patients) for IL-2/IFN (P = .018). Ten patients receiving HD IL-2 were progression-free at 3 years versus three patients receiving IL-2 and IFN (P = .082). The median response durations were 24 and 15 [corrected] months (P = .18) [corrected] and median survivals were 17.5 and 13 months (P = .24). For patients with bone or liver metastases (P = .001) or a primary tumor in place (P = .040), survival was superior with HD IL-2. CONCLUSION: This randomized phase III trial provides additional evidence that HD IL-2 should remain the preferred therapy for selected patients with metastatic renal cell carcinoma.

77 [197]. Melichar, B., P. Koralewski, et al. (2008). "First-line bevacizumab combined with reduced dose interferon-alpha2a is active in patients with metastatic renal cell carcinoma." Ann Oncol **19**(8): 1470-1476.

BACKGROUND: In patients with untreated metastatic renal cell carcinoma (mRCC), progression-free survival (PFS) was longer with bevacizumab + interferon (IFN)-alpha than IFN + placebo (AVOREN trial). In this hypothesis-generating study, subgroup analysis was carried out to determine the effect of IFN dose reduction. PATIENTS AND METHODS: A total of 649 patients received IFN 9 MIU s.c. three times weekly plus bevacizumab 10 mg/kg or placebo every 2 weeks until disease progression. The IFN dose was reduced to 6 or 3 MIU with the development of IFN-attributed toxicity. Differences between treatment arms in PFS, response rate and tolerability were analysed in the reduced-dose group. RESULTS: IFN dose was reduced in 131 patients in the bevacizumab + IFN arm and 97 patients in the IFN + placebo arm during the trial. PFS rates in the bevacizumab + reduced-dose IFN group were comparable with the total population (Kaplan-Meier estimates of event-free rate at 1 year: 0.524 versus 0.427). Bevacizumab + reduced-dose IFN was well tolerated, with substantial decreases in the rate of adverse events following dose reduction. CONCLUSION: This retrospective subgroup analysis suggests that the dose of IFN can be reduced to manage side-effects while maintaining efficacy in patients with mRCC receiving bevacizumab + IFN.

78 [186]. Mills, E. J., B. Rachlis, et al. (2009). "Metastatic renal cell cancer treatments: an indirect comparison meta-analysis." BMC Cancer **9**: 34.

BACKGROUND: Treatment for metastatic renal cell cancer (mRCC) has advanced dramatically with understanding of the pathogenesis of the disease. New treatment options may provide improved progression-free survival (PFS). We aimed to determine the relative effectiveness of new therapies in this field. METHODS: We conducted comprehensive searches of 11 electronic databases from inception to April 2008. We included randomized trials (RCTs) that evaluated bevacizumab, sorafenib, and sunitinib. Two reviewers independently extracted data, in duplicate. Our primary outcome was investigator-assessed PFS. We performed random-effects meta-analysis with a mixed treatment comparison analysis. RESULTS: We included 3 bevacizumab (2 of bevacizumab plus interferon-a [IFN-a]), 2 sorafenib, 1 sunitinib, and 1 temsirolimus trials (total n = 3,957). All interventions offer advantages for PFS. Using indirect comparisons with interferon-alpha as the common comparator, we found that sunitinib was superior to both sorafenib (HR 0.58, 95% CI, 0.38-0.86, P = < 0.001) and bevacizumab + IFN-a (HR 0.75, 95% CI, 0.60-0.93, P = 0.001). Sorafenib was not statistically different from bevacizumab +IFN-a in this same indirect comparison analysis (HR 0.77, 95% CI, 0.52-1.13, P = 0.23). Using placebo as the similar comparator, we were unable to display a significant difference between sorafenib and bevacizumab alone (HR 0.81, 95% CI, 0.58-1.12, P = 0.23). Temsirolimus provided significant PFS in patients with poor prognosis (HR 0.69, 95% CI, 0.57-0.85). CONCLUSION: New interventions for mRCC offer a favourable PFS for mRCC compared to interferon-alpha and placebo.

79 [205]. Motzer, R. J. and E. Basch (2007). "Targeted drugs for metastatic renal cell carcinoma." Lancet **370**(9605): 2071-2073.

80 [185]. Motzer, R. J., G. Hudes, et al. (2009). "Phase I trial of sunitinib malate plus interferon-alpha for patients with metastatic renal cell carcinoma." Clin Genitourin Cancer **7**(1): 28-33.

BACKGROUND: Sunitinib malate is an oral, multitargeted tyrosine kinase inhibitor that has demonstrated superior efficacy over interferon (IFN)-alpha in a phase III trial in first-line, metastatic renal cell carcinoma (RCC). Herein, we report the results of a phase I dose-finding study of sunitinib in combination with IFN-alpha as first-line treatment in patients with metastatic RCC. PATIENTS AND METHODS: Treatment-naive patients with clear-cell metastatic RCC received sunitinib at a starting dose of 50 mg or 37.5 mg orally once daily in 6-week cycles (schedule 4/2) plus IFN-alpha at a starting dose of 3 MU subcutaneously 3 times a week, with weekly intrapatient dose escalation to a maximum of 9 MU as tolerated. Patients who did not tolerate either drug received lower doses of either or had dose interruptions. RESULTS: Twenty-five patients were enrolled; their median age was 64 years (range, 45-77 years). All patients experienced grade 3/4 treatment-emergent adverse events; the most common were neutropenia, fatigue, and thrombocytopenia. After a median of 4 cycles (range, 1-9 cycles), 3 patients (12%) had a partial response, and 20 (80%) had stable disease. CONCLUSION: Although reduced starting doses were tolerated (37.5 mg for sunitinib and 3 MU for IFN-alpha), even these lower doses might not be well tolerated for long-term treatment of patients with metastatic RCC. Based on historical data, sunitinib on schedule 4/2 appears to be more effective as single-agent therapy. Further study of sunitinib plus IFN-alpha on this schedule is not being pursued in RCC.

81 [214]. Motzer, R. J., G. R. Hudes, et al. (2007). "Phase I/II trial of temsirolimus combined with interferon alfa for advanced renal cell carcinoma." J Clin Oncol **25**(25): 3958-3964.

PURPOSE: Temsirolimus, an inhibitor of the mammalian target of rapamycin, has single-agent activity against advanced renal cell carcinoma (RCC). A recommended dose and safety profile for the combination of temsirolimus and interferon alfa (IFN) were determined in patients with advanced RCC. PATIENTS AND METHODS: Patients were enrolled onto a multicenter, ascending-dose study of temsirolimus (5, 10, 15, 20, or 25 mg) administered intravenously once a week combined with IFN (6 or 9 million units [MU]) administered subcutaneously three times per week. An expanded cohort was treated at the recommended dose to obtain additional safety and efficacy information. RESULTS: Seventy-one patients were entered to receive one of six dose levels. The recommended dose was temsirolimus 15 mg/IFN 6 MU based on dose-limiting toxicities of stomatitis, fatigue, and nausea/vomiting, which were observed at higher doses of temsirolimus and IFN. The most frequent grade 3 or 4 toxicities occurring in any cycle included leukopenia, hypophosphatemia, asthenia, anemia, and hypertriglyceridemia for all patients and those who received the recommended dose. Among patients who received the recommended dose (n = 39), 8% achieved partial response and 36% had stable disease for at least 24 weeks. Median progression-free survival for all patients in the study was 9.1 months. CONCLUSION: The combination of temsirolimus and IFN has an acceptable safety profile and displays antitumor activity in patients with advanced RCC. Temsirolimus 15 mg plus IFN 6 MU is the recommended dose for evaluation in a randomized phase III study.

82 [180]. Motzer, R. J., T. E. Hutson, et al. (2009). "Overall survival and updated results for sunitinib compared with interferon alfa in patients with metastatic renal cell carcinoma." J Clin Oncol **27**(22): 3584-3590.

PURPOSE: A randomized, phase III trial demonstrated superiority of sunitinib over interferon alfa (IFN-alpha) in progression-free survival (primary end point) as first-line treatment for metastatic renal cell carcinoma (RCC). Final survival analyses and updated results are reported. PATIENTS AND METHODS: Seven hundred fifty treatment-naive patients with metastatic clear cell RCC were randomly assigned to sunitinib 50 mg orally once daily on a 4 weeks on, 2 weeks off dosing schedule or to IFN-alpha 9 MU subcutaneously thrice weekly. Overall survival was compared by two-sided log-rank and Wilcoxon tests. Progression-free survival, response, and safety end points were assessed with updated follow-up. RESULTS: Median overall survival was greater in the sunitinib group than in the IFN-alpha group (26.4 v 21.8 months, respectively; hazard ratio [HR] = 0.821; 95% CI, 0.673 to 1.001; P = .051) per the primary analysis of unstratified log-rank test (P = .013 per unstratified Wilcoxon test). By stratified log-rank test, the HR was 0.818 (95% CI, 0.669 to 0.999; P = .049). Within the IFN-alpha group, 33% of patients received sunitinib, and 32% received other vascular endothelial growth factor-signaling inhibitors after discontinuation from the trial. Median progression-free survival was 11 months for sunitinib compared with 5 months for IFN-alpha (P < .001). Objective response rate was 47% for sunitinib compared with 12% for IFN-alpha (P < .001). The most commonly reported sunitinib-related grade 3 adverse events included hypertension (12%), fatigue (11%), diarrhea (9%), and hand-foot syndrome (9%). CONCLUSION: Sunitinib demonstrates longer overall survival compared with IFN-alpha plus improvement in response and progression-free survival in the first-line treatment of patients with metastatic RCC. The overall survival highlights an improved prognosis in patients with RCC in the era of targeted therapy.

83 [224]. Motzer, R. J., T. E. Hutson, et al. (2007). "Sunitinib versus interferon alfa in metastatic renal-cell carcinoma." N Engl J Med **356**(2): 115-124.

BACKGROUND: Since sunitinib malate has shown activity in two uncontrolled studies in patients with metastatic renal-cell carcinoma, a comparison of the drug with interferon alfa in a phase 3 trial is warranted. METHODS: We enrolled 750 patients with previously untreated, metastatic renal-cell carcinoma in a multicenter, randomized, phase 3 trial to receive either repeated 6-week cycles of sunitinib (at a dose of 50 mg given orally once daily for 4 weeks, followed by 2 weeks without treatment) or interferon alfa (at a dose of 9 MU given subcutaneously three times weekly). The primary end point was progression-free survival. Secondary end points included the objective response rate, overall survival, patient-reported outcomes, and safety. RESULTS: The median progression-free survival was significantly longer in the sunitinib group (11 months) than in the interferon alfa group (5 months), corresponding to a hazard ratio of 0.42 (95% confidence interval, 0.32 to 0.54; P<0.001). Sunitinib was also associated with a higher objective response rate than was interferon alfa (31% vs. 6%, P<0.001). The proportion of patients with grade 3 or 4 treatment-related fatigue was significantly higher in the group treated with interferon alfa, whereas diarrhea was more frequent in the sunitinib group (P<0.05). Patients in the sunitinib group reported a significantly better quality of life than did patients in the interferon alfa group (P<0.001). CONCLUSIONS: Progression-free survival was longer and response rates were higher in patients with metastatic renal-cell cancer who received sunitinib than in those receiving interferon alfa (ClinicalTrials.gov numbers, NCT00098657 and NCT00083889 [ClinicalTrials.gov]).

84 [164]. Naito, S., T. Tsukamoto, et al. (2010). "An early phase II trial of S-1 in Japanese patients with cytokine-refractory metastatic renal cell carcinoma." Cancer Chemother Pharmacol **66**(6): 1065-1070.

PURPOSE: S-1, an oral anticancer agent, contains tegafur (FT), 5-chloro-2,4-dihydroxypyridine (CDHP), and potassium oxonate (Oxo) at a molar ratio of FT:CDHP:Oxo = 1:0.4:1. The aim of this trial was to investigate the efficacy and safety of S-1 in Japanese patients with cytokine-refractory metastatic renal cell carcinoma (RCC). METHODS: We conducted a non-randomized, open-label trial in Japanese patients with metastatic RCC who had received nephrectomy and had failed cytokine-based immunotherapy. The primary endpoint was response rate. S-1 40-60 mg based on the body surface area was administered twice daily (80-120 mg/day) for 4 consecutive weeks, followed by a 2-week rest period; cycles were repeated every 6 weeks. Patients continued treatment until disease progression, unacceptable toxicity, or withdrawal of consent. RESULTS: A total of 20 eligible patients were enrolled. Among these, 3 patients had partial response, yielding objective response rate of 15%; 13 patients had no change; 4 patients had progressive disease. The median time-to-progression and median overall survival were 12.0 and 25.7 months, respectively. The initial adverse event was generally mild to moderate in severity. The most common grade 3/4 drug-related hematological and non-hematological adverse events were neutropenia (20.0%) and anorexia (20.0%), respectively. CONCLUSIONS: S-1 is active and well tolerated for the treatment of cytokine-refractory metastatic RCC.

85 [143]. Negrier, S., G. Gravis, et al. (2011). "Temsirolimus and bevacizumab, or sunitinib, or interferon alfa and bevacizumab for patients with advanced renal cell carcinoma (TORAVA): a randomised phase 2 trial." Lancet Oncol **12**(7): 673-680.

BACKGROUND: Combining targeted treatments for renal cell carcinoma has been suggested as a possible method to improve treatment efficacy. We aimed to assess the potential synergistic or additive effect of the combination of bevacizumab, directed against the VEGF receptor, and temsirolimus, an mTOR inhibitor, in metastatic renal cell carcinoma. METHODS: TORAVA was an open-label, multicentre randomised phase 2 study undertaken in 24 centres in France. Patients aged 18 years or older who had untreated metastatic renal cell carcinoma were randomly assigned (2:1:1) to receive the combination of bevacizumab (10 mg/kg every 2 weeks) and temsirolimus (25 mg weekly; group A), or one of the standard treatments: sunitinib (50 mg/day for 4 weeks followed by 2 weeks off; group B), or the combination of interferon alfa (9 mIU three times per week) and bevacizumab (10 mg/kg every 2 weeks; group C). Randomisation was done centrally and independently from other study procedures with computer-generated permuted blocks of four and eight patients stratified by participating centre and Eastern Cooperative Oncology Group performance status. The primary endpoint was progression-free survival (PFS) at 48 weeks (four follow-up CT scans), which was expected to be above 50% in group A. Analysis was by intention to treat. The study is ongoing for long-term overall survival. This study is registered with ClinicalTrials.gov, number NCT00619268. FINDINGS: Between March 3, 2008 and May 6, 2009, 171 patients were randomly assigned: 88 to the experimental group (group A), 42 to group B, and 41 to group C. PFS at 48 weeks was 29.5% (26 of 88 patients, 95% CI 20.0-39.1) in group A, 35.7% (15 of 42, 21.2-50.2) in group B, and 61.0% (25 of 41, 46.0-75.9) in group C. Median PFS was 8.2 months (95% CI 7.0-9.6) in group A, 8.2 months (5.5-11.7) in group B, and 16.8 months (6.0-26.0) in group C. 45 (51%) of 88 patients in group A stopped treatment for reasons other than progression compared with five (12%) of 42 in group B and 15 (38%) of 40 in group C. Grade 3 or worse adverse events were reported in 68 (77%) of 88 patients in group A versus 25 (60%) of 42 in group B and 28 (70%) of 40 in group C. Serious adverse events were reported in 39 (44%) of 88, 13 (31%) of 42, and 18 (45%) of 40 patients in groups A, B, and C, respectively. INTERPRETATION: The toxicity of the temsirolimus and bevacizumab combination was much higher than anticipated and limited treatment continuation over time. Clinical activity was low compared with the benefit expected from sequential use of each targeted therapy. This combination cannot be recommended for first-line treatment in patients with metastatic renal cell carcinoma. FUNDING: French Ministry of Health and Wyeth Pharmaceuticals.

86 [174]. Negrier, S., E. Jager, et al. (2010). "Efficacy and safety of sorafenib in patients with advanced renal cell carcinoma with and without prior cytokine therapy, a subanalysis of TARGET." Med Oncol **27**(3): 899-906.

Before the development of targeted therapies, administration of cytokines (e.g., interleukin-2, interferon-alpha) was the primary systemic treatment option for advanced renal cell carcinoma. Sorafenib, an oral targeted, multikinase inhibitor, significantly prolonged progression-free survival and overall survival in the Treatment Approaches in Renal Cancer Global Evaluation Trial (TARGET), a large (N = 903) phase III, double-blind, randomised, placebo-controlled study of patients with advanced renal cell carcinoma resistant to standard therapy. This analysis of a patient subgroup from TARGET evaluated the safety and efficacy of sorafenib in patients who had received prior cytokine therapy (sorafenib: n = 374; placebo: n = 368) and in patients who were cytokine-naive (sorafenib: n = 77; placebo: n = 84). Progression-free survival was significantly prolonged with sorafenib therapy compared with placebo among patients with and without prior cytokine therapy (respectively 5.5 vs. 2.7 months; hazard ratio, 0.54; 95% confidence interval, 0.45-0.64 and 5.8 vs. 2.8 months; hazard ratio, 0.48; 95% confidence interval, 0.32-0.73). Clinical benefit rates for sorafenib-treated patients compared with placebo patients were also higher (cytokine-treated: 83 vs. 54.3%; cytokine-naive: 85.7 vs. 56.0%). Sorafenib was well tolerated in both subgroups (grade 3/4: 20 and 22%, respectively). Sorafenib demonstrated a consistent, significant clinical benefit against advanced renal cell carcinoma, with a twofold improvement in progression-free survival and disease control rate, with similar toxicities in patients with or without prior cytokine treatment.

87 [190]. Negrier, S., D. Perol, et al. (2008). "Randomized study of intravenous versus subcutaneous interleukin-2, and IFNalpha in patients with good prognosis metastatic renal cancer." Clin Cancer Res **14**(18): 5907-5912.

PURPOSE: Metastatic renal cancer patients with a single metastatic site are potentially amenable to interleukin 2 (IL-2) + IFN-alpha. A French immunotherapy intergroup multicenter trial assessed the potential benefit of i.v. over s.c. administration of IL-2 in this combination. EXPERIMENTAL DESIGN: Untreated patients with one metastatic site were randomized to continuous i.v. infusion (18 x 10(6) IU/m(2)/d; arm A) or twice daily s.c. injections (9 x 10(6) or 18 x 10(6) IU; arm B) of IL-2, associated with s.c. IFN-alpha (6 x 10(6) IU) 3 days per week in both arms. Tumor response was assessed (WHO criteria) at weeks 12 and 24 to 26. The primary end point was overall survival, with an expected 15% improvement at 4 years with i.v. IL-2. The planned sample size was 220 (80% power, 5% significance, one-sided test). Intent-to-treat analysis was done and survivals were compared using log-rank tests. RESULTS: From January 2000 to January 2005, 80 and 75 patients were randomized to arms A and B, respectively. Enrollment was stopped early because of low accrual; analysis was done at 42.5 months median follow-up. Patient characteristics were well balanced between groups. Response rates were 17.9% versus 21.3% in arms A and B. Progression-free survival rates were not significantly different. Overall survival difference was not significant: median 33 months (95% confidence interval, 27.0-40.2; P = 0.202). CONCLUSIONS: In combination with IFN-alpha in selected, good prognosis metastatic renal cell carcinoma patients, i.v. IL-2 offers no significant advantage over s.c. IL-2 and induces higher toxicity. Although i.v. IL-2 induced longer responses, it seems unreasonable to continue recommending this regimen after the recent introduction of more effective therapies.

88 [210]. Negrier, S., D. Perol, et al. (2007). "Medroxyprogesterone, interferon alfa-2a, interleukin 2, or combination of both cytokines in patients with metastatic renal carcinoma of intermediate prognosis: results of a randomized controlled trial." Cancer **110**(11): 2468-2477.

BACKGROUND: Few randomized trials have compared the survival benefit of interferon-alfa over controls in metastatic renal cell carcinoma, and none has been performed using interleukin-2. The Programme Etude Rein Cytokines (PERCY) Quattro trial was designed to evaluate both cytokines for their survival benefit to intermediate prognosis patients, who represent the majority of candidates for these treatments. METHODS: Patients were randomized in a 2-by-2 factorial design to medroxyprogesterone acetate 200 mg daily, interferon-alfa 9 million IU 3 times a week, subcutaneous interleukin-2 9 million IU daily, or a combination of both cytokines. Tumor response was evaluated at Week 12 and Month 6; progression-free patients received further identical treatment for a maximum of 3 additional months. Primary endpoint was overall survival; secondary endpoints were disease-free survival, response rate, toxicity, and quality of life. Survival was analyzed on an intent-to-treat basis. RESULTS: From January 2000 to July 2004, 492 patients were enrolled. Analysis was performed after a 29.2-month median follow-up (range, 0 months to 54.6 months). There were no significant survival differences between the 244 interferon-alfa-treated patients and 248 noninterferon-alfa patients (hazard ratio, 1.00; 95% CI, 0.81-1.24) or between the 247 interleukin-2 and 245 noninterleukin-2-treated patients (hazard ratio, 1.07; 95% CI, 0.87-1.33; log rank, 0.99 and 0.52, respectively). Grade 3-4 toxicities were significantly more frequent in cytokine-treated patients than in medroxyprogesterone-treated patients. CONCLUSIONS: Subcutaneous interleukin-2 and/or interferon-alfa provide no survival benefit in metastatic renal cancers of intermediate prognosis, and they induce a significant risk of toxicity. Newly available angiogenesis inhibitors should be preferred for these patients.

89 [167]. Ng, C. S., X. Wang, et al. (2010). "Perfusion CT in patients with metastatic renal cell carcinoma treated with interferon." AJR Am J Roentgenol **194**(1): 166-171.

OBJECTIVE: The objective of our study was to assess the potential value of tumor perfusion parameters measured by perfusion CT as possible biomarkers of prognosis and early indicator of treatment efficacy in patients with metastatic conventional renal cell carcinoma (RCC) treated with interferon. MATERIALS AND METHODS: This study comprised 37 patients with metastatic RCC who were enrolled in a larger (n=118) randomized clinical trial of intermediate- versus low-dose interferon. Tumor perfusion parameters-that is, tumor blood flow, blood volume, mean transit time (MTT), and permeability-surface area product-of index metastatic lesions were obtained at baseline and at 8-week follow-up. Baseline perfusion parameters and changes at follow-up were compared, and their associations with patient progression-free survival were estimated. Univariate and multivariate analyses were performed. RESULTS: Twenty-eight patients were assessable. Median progression-free survival was 5.3 months (95% CI, 2.4-7.4 months), with one partial response. Tumor blood flow at baseline was inversely associated with patient progression-free survival in both univariate (hazard ratio [HR]=1.006, p=0.025) and multivariate (HR=1.007, p=0.012) analyses. There were significant increases in tumor blood flow and reductions in MTT on follow-up scans compared with baseline scans (both, p=0.04), but no association between changes in perfusion parameters and progression-free survival was detected. CONCLUSION: Patients with highly vascularized metastatic RCC as shown by high baseline tumor blood flow appear to have a worse prognosis than those who do not. Tumor perfusion may be a useful biomarker of prognosis and additionally, in the future, may assist in treatment stratification. The potential utility of perfusion CT as an early response indicator was probably inadequately assessed in this study because of the limited antiangiogenic activity of interferon in metastatic RCC.

90 [152]. Niwakawa, M., K. Hashine, et al. (2012). "Phase I trial of sorafenib in combination with interferon-alpha in Japanese patients with unresectable or metastatic renal cell carcinoma." Invest New Drugs **30**(3): 1046-1054.

BACKGROUND: We investigated the safety, pharmacokinetics, tumor response, and immunological parameters of sorafenib plus interferon alpha-2b [corrected] (IFN) in Japanese patients with advanced RCC. PATIENTS AND METHODS: After 2 weeks of IFN-alone treatment, eligible patients received 28-day cycles of continuous sorafenib 200 mg (Cohort 1) or 400 mg (Cohorts 2 and 3) twice daily combined with intramuscular IFN 6 (Cohorts 1 and 2) or 9 (Cohort 3) million international units (MIU) three times a week. RESULTS: A total of 18 patients received at least one dose of sorafenib plus IFN. Five patients had dose-limiting toxicities (DLTs). The most common DLT was fatigue, experienced in four DLT patients. All 18 patients experienced at least one treatment-emergent adverse event (AE). The most common treatment-emergent AEs included fatigue, fever, platelets, leukocytes, hemoglobin, weight loss and anorexia. Five patients had confirmed partial response and 11 had stable disease, a response rate of 27.8%. IFN had no relevant impact on the pharmacokinetics of sorafenib. CONCLUSIONS: Sorafenib administered in combination with IFN was well tolerated, with promising results in efficacy. Continuous sorafenib 400 mg twice daily in combination with IFN 6 MIU three times a week is recommended in Japanese patients with advanced RCC.

91 [192]. Obara, W., Y. Mizutani, et al. (2008). "Prospective study of combined treatment with interferon-alpha and active vitamin D3 for Japanese patients with metastatic renal cell carcinoma." Int J Urol **15**(9): 794-799.

OBJECTIVES: To assess the safety and efficacy of combined therapy with interferon-alpha (INF-alpha) and active vitamin D(3) for metastatic renal cell carcinoma (RCC). METHODS: Sixteen patients with metastatic RCC were enrolled in this prospective study. All received oral alfacalcidol (1 microg once daily) and INF-alpha (Sumiferon; 3 million units, three times a week). The primary endpoint was the response rate (defined as complete + partial remission). Secondary endpoints were cancer-specific survival and toxicity. The median follow-up period was 17 months (range: 5-49 months). RESULTS: The median age of the patients was 68 years (range: 41-73 years). The sites of metastases were: lung in 13 patients, bone in one, lung and bone in one, and lung, bone, and lymph nodes in one. Four patients (25%) had a partial response (PR), 10 patients (62.5%) showed no change (NC), and two patients (12.5%) had progressive disease (PD). The median cancer-specific survival time was 45 months. One patient had to discontinue vitamin D(3) because of hypercalcemia. Kaplan-Meier survival analysis revealed that metastasis at the time of initial diagnosis and older than average age were significant predictors of poor survival (P < 0.05). CONCLUSIONS: Combined treatment with INF-alpha and active vitamin D(3) has shown to be safe and effective for metastatic RCC patients.

92 [260]. O'Brien, M. F., D. Rea, et al. (2004). "Interleukin-2, interferon-alpha and 5-fluorouracil immunotherapy for metastatic renal cell carcinoma: the all Ireland experience." Eur Urol **45**(5): 613-618; discussion 619.

OBJECTIVE: To analyse the long-term efficacy of combined interferon-alpha (IFN-alpha) and interleukin-2 (IL-2) subcutaneously, with 5-fluorouracil (5-FU) intravenously in a general multicentre setting, as treatment for metastatic renal cell carcinoma (RCC). METHODS: Fifty-nine patients with metastatic RCC were scheduled to receive an 8-week cycle of immunotherapy. Karnofsky score ranged from 70 to 100 (median 90). Thirty-one patients at presentation had metastases of which 14 underwent nephrectomy. Metastases occurred in multiple organs (lung 74%, mediastinal lymphadenopathy 22%, bone 21%). Therapeutic response and survival were analysed. RESULTS: Nine patients died from disease progression prior to completion of one full cycle. Six cases (10%) have stable disease at a follow-up of 51 months (range 20-88 months). Currently 11 patients (19%) are alive at a mean follow-up of 45 months (range 18-88 months). Forty-eight patients (81%) died of their disease at a mean follow-up of 10 months (range 0.5-46 months). Survival rate at 1 year was 53%, at 2 years 21%, at 3 years 16% and at 5 years 5%. Overall median survival is 10 months. CONCLUSION: IL-2 and IFN-alpha with 5-FU based immunotherapy achieve durable survival rates at 3 years in a minority of patients. Addition of 5-FU does not increase survival in our group. This study population is very different to other reported series. However it reflects better the entire population with metastatic RCC though results are subsequently poorer. Identifying patients that will respond is paramount.

93 [199]. Pacheco, A. V., K. Rasila, et al. (2008). "Phase II studies of antiangiogenic four drug regimens for the treatment of advanced renal cell carcinoma: FUNIL-retinoid and the FUNIL-thalidomide protocols." Urol Oncol **26**(6): 610-615.

BACKGROUND AND PURPOSE: The objective of these studies was to determine the activity of two alternative 4- drug combinations using cis-retinoic acid or thalidomide administered with a previously developed combination of 5 fluorouracil, interferon-alpha, and interleukin 2 (FUNIL), for patients with metastatic renal cell carcinoma (RRC). METHODS: Patients enrolled in these studies had progressive measurable metastatic renal cell cancer and signed an informed consent. Treatments included continuous infusions of 5-fluorouracil, interferon-alpha, 6 MIU/m2 given subcutaneous on days 1, 3, and 5 every week, interleukin-2 6 MIU/m2/day given by continuous infusion days 2 to 5 every week, and either cis-retinoic acid at a dose of 1 mg/kg/day orally in two divided doses or thalidomide given at an initial dose of 200 mg per day. Each cycle consisted of 6 or 4 weeks of the combinations, respectively, followed by a 2-week rest. Patients were evaluated for response prior to each successive cycle. A 2-step mini-max statistical design was used. RESULTS: In the cis-retinoid study, 20 patients were enrolled. One patient was ineligible. There were 1 complete and 2 partial responses (one confirmed and one unconfirmed) (15.8%), 1 stable disease, and 15 disease progression. In the thalidomide combination study, 20 patients were enrolled, but only 19 are assessable. One patient progressed early and was never treated. There were 2 partial responses (10.5%), 4 stable disease, and 13 progressive disease. CONCLUSION: Neither the FUNIL-cis-retinoid nor the FUNIL-thalidomide regimens met their primary objective first step endpoint of 3 confirmed responses. Both regimens had significant adverse effects and neither is considered promising for further study.

94 [252]. Padrik, P., K. Leppik, et al. (2004). "Combination therapy with capecitabine and interferon alfa-2A in patients with advanced renal cell carcinoma: a phase II study." Urol Oncol **22**(5): 387-392.

Capecitabine is a fluoropyrimidine carbamate capable of exploiting the high concentrations of thymidine phosphorylase in tumor tissue to achieve activation preferentially at the tumor site. Thymidine phosphorylase activity is high in renal cell carcinoma tissue. Interferon alfa has been proved to be the agent for standard therapy in metastatic renal cell carcinoma. The purpose of the study was to assess the efficacy and toxicity of combining capecitabine and interferon alfa-2A in patients with advanced renal cell carcinoma. Twenty-five patients with advanced renal cell carcinoma and no prior systemic therapy were treated with the combination of capecitabine at a dose of 1,250 mg/m2 twice daily for 2 weeks after every 21 days and interferon alfa-2A 6 million U three times a week. The overall response rate was 24.0% (95% CI, 9.4-45.1%), from 6 responded patients 5 had partial responses and 1 complete response. Stable disease status was achieved in 9 patients (36.0% with 95% CI 18.0-57.5%). The median survival time was 248 days (95% CI, 173-265 days). The median time to progression was 126 days (95% CI, 49-165 days). Grade 3-4 toxicities occurred in 12 patients and included fatigue (33.3%), nausea, hand-foot syndrome (both 12.5%), anorexia (8.3%), vomiting, anemia and neutropenia (all 4.2%). The capecitabine and interferon alfa-2A combination has clinical activity and an acceptable toxicity profile in patients with metastatic renal cell carcinoma. The importance of adding capecitabine to interferon alfa needs to be confirmed in a randomized trial.

95 [134]. Patil, S., R. A. Figlin, et al. (2012). "Q-TWiST analysis to estimate overall benefit for patients with metastatic renal cell carcinoma treated in a phase III trial of sunitinib vs interferon-alpha." Br J Cancer **106**(10): 1587-1590.

BACKGROUND: In a randomised phase III trial of treatment-naive patients with metastatic renal cell carcinoma, sunitinib showed significant improvement in progression-free survival (PFS) compared with interferon (IFN)-alpha. We assessed between-treatment differences in overall benefit using a quality-adjusted Time Without Symptoms of disease progression or Toxicity of treatment (TWiST; Gelber and Goldhirsch) analysis. METHODS: In this analysis, in which only grade 3/4 treatment-related toxicities were included, overall survival was partitioned into three health states: toxicity (time with toxicity after randomisation and before progression), time without symptoms of disease progression or toxicity, and time from progression until death. Between-treatment differences in the mean duration of each state were calculated. A threshold utility analysis was used to assess quality-adjusted TWiST (Q-TWiST) outcomes. RESULTS: Q-TWiST scores showed that quality-adjusted survival time was greater with sunitinib than with IFN-alpha, even though certain grade 3/4 toxicities occurred more frequently with sunitinib. For both treatments, the mean number of days with toxicity was small compared with PFS. This effect was more pronounced with sunitinib in which time spent without progression or toxicity was 151 days greater than with IFN-alpha. CONCLUSION: Patients randomised to sunitinib had longer clinical benefit, defined as Q-TWiST scores, than patients randomised to IFN-alpha.

96 [121]. Peri, S., K. Devarajan, et al. (2013). "Meta-analysis identifies NF-kappaB as a therapeutic target in renal cancer." PLoS One **8**(10): e76746.

OBJECTIVE: To determine the expression patterns of NF-kappaB regulators and target genes in clear cell renal cell carcinoma (ccRCC), their correlation with von Hippel Lindau (VHL) mutational status, and their association with survival outcomes. METHODS: Meta-analyses were carried out on published ccRCC gene expression datasets by RankProd, a non-parametric statistical method. DEGs with a False Discovery Rate of < 0.05 by this method were considered significant, and intersected with a curated list of NF-kappaB regulators and targets to determine the nature and extent of NF-kappaB deregulation in ccRCC. RESULTS: A highly-disproportionate fraction (~40%; p < 0.001) of NF-kappaB regulators and target genes were found to be up-regulated in ccRCC, indicative of elevated NF-kappaB activity in this cancer. A subset of these genes, comprising a key NF-kappaB regulator (IKBKB) and established mediators of the NF-kappaB cell-survival and pro-inflammatory responses (MMP9, PSMB9, and SOD2), correlated with higher relative risk, poorer prognosis, and reduced overall patient survival. Surprisingly, levels of several interferon regulatory factors (IRFs) and interferon target genes were also elevated in ccRCC, indicating that an 'interferon signature' may represent a novel feature of this disease. Loss of VHL gene expression correlated strongly with the appearance of NF-kappaB- and interferon gene signatures in both familial and sporadic cases of ccRCC. As NF-kappaB controls expression of key interferon signaling nodes, our results suggest a causal link between VHL loss, elevated NF-kappaB activity, and the appearance of an interferon signature during ccRCC tumorigenesis. CONCLUSIONS: These findings identify NF-kappaB and interferon signatures as clinical features of ccRCC, provide strong rationale for the incorporation of NF-kappaB inhibitors and/or and the exploitation of interferon signaling in the treatment of ccRCC, and supply new NF-kappaB targets for potential therapeutic intervention in this currently-incurable malignancy.

97 [233]. Polite, B. N., A. A. Desai, et al. (2006). "Combination therapy of imatinib mesylate and interferon-alpha demonstrates minimal activity and significant toxicity in metastatic renal cell carcinoma: results of a single- institution phase II trial." Clin Genitourin Cancer **4**(4): 275-280.

BACKGROUND: Renal cell carcinoma (RCC) is characterized by increased expression of vascular endothelial growth factor and platelet-derived growth factor (PDGF)-beta, both of which contribute to its angiogenic phenotype. Interferon-alpha (IFN-alpha) improves survival in patients with metastatic RCC, perhaps partly because of its antiangiogenic properties. Imatinib mesylate inhibits PDGF-mediated signal transduction and might thus have antiangiogenic activity as well. PATIENTS AND METHODS: Patients with metastatic RCC were treated with IFN-alpha (9 million IU subcutaneously 3 times weekly) and oral imatinib mesylate (600 mg daily starting on day 8). Therapy was continuous, and response was evaluated at 8-week intervals using the Response Evaluation Criteria in Solid Tumors. Baseline plasma PDGF-AA, PDGF-AB, and PDGF-BB levels were obtained. RESULTS: Between January 2003 and January 2005, 17 patients were treated. One patient (6%) had a partial response, 4 (24%) had stable disease, 7 (41%) had progressive disease, and 5 (29%) were unevaluable because of early withdrawal secondary to toxicity. Median time to progression (TTP) using the Kaplan-Meier method was 8 weeks, and median overall survival was 17.8 months. Six patients (35%) withdrew from therapy because of toxicity, and 9 patients (53%) experienced > or = 1 grade 3/4 toxicity. Platelet-derived growth factor AA, AB, and BB plasma levels did not correlate with TTP or overall survival. CONCLUSION: Based on a response rate of only 6%, a median TTP of 2 months, and significant toxicities, further study of IFN-alpha in combination with imatinib mesylate is not recommended in patients with metastatic RCC.

98 [263]. Rathmell, W. K., S. B. Malkowicz, et al. (2004). "Phase II trial of 5-fluorouracil and leucovorin in combination with interferon-alpha and interleukin-2 for advanced renal cell cancer." Am J Clin Oncol **27**(2): 109-112.

Recent clinical trials have demonstrated activity of chemoimmunotherapy with interleukin-2 (IL-2), interferon-[alpha], and 5-fluorouracil (5-FU) in advanced renal cell cancer. A phase II study was performed to evaluate the affect of adding the potentiating agent leucovorin to this combination regimen. Treatment courses consisted of IL-2 5 MIU/m2 subcutaneously days 1, 3, and 5 of weeks 1 to 4, interferon-[alpha] 3 MIU/m2 subcutaneously on days 1, 3, and 5 of weeks 1 to 4, and leucovorin 50 mg/m2 IV followed by 5-FU 450 mg/m2 IV infusion weekly weeks 1 to 4. Patients were given no treatment on weeks 5 and 6 of the 6-week treatment cycle. Of the 20 patients enrolled in the study, 16 were evaluable for toxicity and 15 were evaluable for tumor response. The most severe toxicities included three reports of grade IV diarrhea; overall, nine incidents of grade III or IV toxicity were reported. No objective antitumor responses were observed, and the median time to progression was 2.8 months. We conclude that this combination chemoimmunotherapy regimen has substantial toxicity but no significant antitumor activity in patients with advanced stage renal cell carcinoma.

99 [118]. Rini, B. I., J. Bellmunt, et al. (2014). "Randomized phase III trial of temsirolimus and bevacizumab versus interferon alfa and bevacizumab in metastatic renal cell carcinoma: INTORACT trial." J Clin Oncol **32**(8): 752-759.

PURPOSE: To prospectively determine the efficacy of combination therapy with temsirolimus plus bevacizumab versus interferon alfa (IFN) plus bevacizumab in metastatic renal cell carcinoma (mRCC). PATIENTS AND METHODS: In a randomized, open-label, multicenter, phase III study, patients with previously untreated predominantly clear-cell mRCC were randomly assigned, stratified by prior nephrectomy and Memorial Sloan-Kettering Cancer Center prognostic group, to receive the combination of either temsirolimus (25 mg intravenously, weekly) or IFN (9 MIU subcutaneously thrice weekly) with bevacizumab (10 mg/kg intravenously, every 2 weeks). The primary end point was independently assessed progression-free survival (PFS). RESULTS: Median PFS in patients treated with temsirolimus/bevacizumab (n = 400) versus IFN/bevacizumab (n = 391) was 9.1 and 9.3 months, respectively (hazard ratio [HR], 1.1; 95% CI, 0.9 to 1.3; P = .8). There were no significant differences in overall survival (25.8 nu 25.5 months; HR, 1.0; P = .6) or objective response rate (27.0% nu 27.4%) with temsirolimus/bevacizumab versus IFN/bevacizumab, respectively. Patients receiving temsirolimus/bevacizumab reported significantly higher overall mean scores in the Functional Assessment of Cancer Therapy-Kidney Symptom Index (FKSI) -15 and FKSI-Disease Related Symptoms subscale compared with IFN/bevacizumab (indicating improvement); however, no differences in global health outcome measures were observed. Treatment-emergent all-causality grade >/= 3 adverse events more common (P < .001) with temsirolimus/bevacizumab were mucosal inflammation, stomatitis, hypophosphatemia, hyperglycemia, and hypercholesterolemia, whereas neutropenia was more common with IFN/bevacizumab. Incidence of pneumonitis with temsirolimus/bevacizumab was 4.8%, mostly grade 1 or 2. CONCLUSION: Temsirolimus/bevacizumab combination therapy was not superior to IFN/bevacizumab for first-line treatment in clear-cell mRCC.

100 [161]. Rini, B. I., S. Halabi, et al. (2010). "Phase III trial of bevacizumab plus interferon alfa versus interferon alfa monotherapy in patients with metastatic renal cell carcinoma: final results of CALGB 90206." J Clin Oncol **28**(13): 2137-2143.

PURPOSE: Bevacizumab is an antibody that binds vascular endothelial growth factor and has activity in metastatic renal cell carcinoma (RCC). Interferon alfa (IFN-alpha) is the historic standard initial treatment for RCC. A prospective, randomized, phase III trial of bevacizumab plus IFN-alpha versus IFN-alpha monotherapy was conducted. PATIENTS AND METHODS: Patients with previously untreated, metastatic clear cell RCC were randomly assigned to receive either bevacizumab (10 mg/kg intravenously every 2 weeks) plus IFN-alpha (9 million units subcutaneously three times weekly) or the same dose and schedule of IFN-alpha monotherapy in a multicenter phase III trial. The primary end point was overall survival (OS). Secondary end points were progression-free survival (PFS), objective response rate, and safety. RESULTS: Seven hundred thirty-two patients were enrolled. The median OS time was 18.3 months (95% CI, 16.5 to 22.5 months) for bevacizumab plus IFN-alpha and 17.4 months (95% CI, 14.4 to 20.0 months) for IFN-alpha monotherapy (unstratified log-rank P = .097). Adjusting on stratification factors, the hazard ratio was 0.86 (95% CI, 0.73 to 1.01; stratified log-rank P = .069) favoring bevacizumab plus IFN-alpha. There was significantly more grade 3 to 4 hypertension (HTN), anorexia, fatigue, and proteinuria for bevacizumab plus IFN-alpha. Patients who developed HTN on bevacizumab plus IFN-alpha had a significantly improved PFS and OS versus patients without HTN. CONCLUSION: OS favored the bevacizumab plus IFN-alpha arm but did not meet the predefined criteria for significance. HTN may be a biomarker of outcome with bevacizumab plus IFN-alpha.

101 [189]. Rini, B. I., S. Halabi, et al. (2008). "Bevacizumab plus interferon alfa compared with interferon alfa monotherapy in patients with metastatic renal cell carcinoma: CALGB 90206." J Clin Oncol **26**(33): 5422-5428.

PURPOSE: Bevacizumab is an antibody that binds to vascular endothelial growth factor (VEGF) and has activity in metastatic renal cell carcinoma (RCC). Interferon alfa (IFN) is a historic standard first-line treatment for RCC. A prospective, randomized phase III trial of bevacizumab plus IFN versus IFN monotherapy was conducted. PATIENTS AND METHODS: Patients with previously untreated, metastatic clear-cell RCC were randomly assigned to receive either bevacizumab (10 mg/kg intravenously every 2 weeks) plus IFN (9 million U subcutaneously three times weekly) or the same dose and schedule of IFN monotherapy in a multicenter phase III trial. The primary end point was overall survival (OS). Secondary end points were progression-free survival (PFS), objective response rate (ORR), and safety. RESULTS: Between October 2003 and July 2005, 732 patients were enrolled. The prespecified stopping rule for OS has not yet been reached. The median PFS was 8.5 months in patients receiving bevacizumab plus IFN (95% CI, 7.5 to 9.7 months) versus 5.2 months (95% CI, 3.1 to 5.6 months) in patients receiving IFN monotherapy (log-rank P < .0001). The adjusted hazard ratio was 0.71 (95% CI, 0.61 to 0.83; P < .0001). Bevacizumab plus IFN had a higher ORR as compared with IFN (25.5% [95% CI, 20.9% to 30.6%] v 13.1% [95% CI, 9.5% to 17.3%]; P < .0001). Overall toxicity was greater for bevacizumab plus IFN, including significantly more grade 3 hypertension (9% v 0%), anorexia (17% v 8%), fatigue (35% v 28%), and proteinuria (13% v 0%). CONCLUSION: Bevacizumab plus IFN produces a superior PFS and ORR in untreated patients with metastatic RCC as compared with IFN monotherapy. Toxicity is greater in the combination therapy arm.

102 [258]. Rini, B. I., S. Halabi, et al. (2004). "Cancer and Leukemia Group B 90206: A randomized phase III trial of interferon-alpha or interferon-alpha plus anti-vascular endothelial growth factor antibody (bevacizumab) in metastatic renal cell carcinoma." Clin Cancer Res **10**(8): 2584-2586.

The majority of sporadic clear cell renal cell carcinoma (RCC) is characterized by loss of heterozygosity of the von Hippel-Lindau (VHL) tumor suppressor gene and somatic inactivation of the remaining VHL allele. The resulting VHL gene silencing leads to induction of hypoxia-regulated genes including vascular endothelial growth factor (VEGF). Thus, therapeutic inhibition of VEGF holds promise for treatment of this historically refractory malignancy. An antibody to VEGF (bevacizumab, Avastin) has demonstrated a significant prolongation of time to disease progression compared with placebo in patients with metastatic RCC. Interferon-alpha (IFN-alpha) is a standard initial cytokine therapy in RCC with a modest response rate and a survival advantage demonstrated in randomized trials. We hypothesized that the addition of anti-VEGF therapy to IFN-alpha would prolong survival in untreated metastatic RCC patients. A Phase III trial is now being conducted randomizing untreated, metastatic clear cell RCC patients to IFN-alpha alone or IFN-alpha plus Avastin.

103 [238]. Rini, B. I., V. Weinberg, et al. (2006). "Maximal COX-2 immunostaining and clinical response to celecoxib and interferon alpha therapy in metastatic renal cell carcinoma." Cancer **106**(3): 566-575.

BACKGROUND: Cyclooxygenase-2 (COX-2) plays a major role in the development of cancer through numerous mechanisms. COX-2 is expressed in the majority of renal cell carcinoma (RCC) tumors and correlates with stage, grade, and microvessel density. Based on potential additive or synergistic antitumor effects, interferon-alpha (IFNalpha) and celecoxib, an oral COX-2 inhibitor, were given to metastatic RCC patients in a Phase II trial. METHODS: Patients with untreated, metastatic RCC received IFNalpha 3 million units (MU) daily and celecoxib 400 mg orally (p.o.) twice daily continuously until disease progression or unacceptable toxicity. Pretreatment, paraffin-embedded RCC tumor samples were immunohistochemically stained for COX-2 expression and plasma basic fibroblast growth factor (bFGF) and vascular endothelial growth factor (VEGF) levels were assayed to determine predictive or prognostic potential. RESULTS: There were three partial responses among 25 patients treated (objective response rate, 12%; 95% confidence interval [CI], 3-31%). The observed median time to disease progression (TTP) for the entire cohort was 3.3 months. A significant association between maximal COX-2 staining and clinical response was observed: all patients who experienced an objective response demonstrated 3+ COX-2 tumor immunostaining (trend test: P=0.03). Therapy was well tolerated without cardiac or other notable toxicity. CONCLUSIONS: The addition of celecoxib to IFNalpha did not increase the objective response rate or TTP of this unselected cohort. Maximal COX-2 tumor immunostaining may identify RCC patents more likely to achieve clinical benefit with COX-2 inhibition in combination with IFNalpha. Further investigation of this combination in 3+ COX-2-overexpressing RCC tumors is warranted.

104 [209].	Rixe, O., R. M. Bukowski, et al. (2007). "Axitinib treatment in patients with cytokine-refractory metastatic renal-cell cancer: a phase II study." Lancet Oncol **8**(11): 975-984.

BACKGROUND: Axitinib (AG-013736) is an oral, potent, and selective inhibitor of vascular endothelial growth factor receptors 1, 2, and 3. We aimed to assess the activity and safety of axitinib in patients with metastatic renal-cell cancer who had failed on previous cytokine-based treatment. METHODS: Between Oct 3, 2003, and April 7, 2004, 52 patients were enrolled. All patients who had at least one measurable target lesion received axitinib orally (starting dose 5 mg twice daily). The primary endpoint was objective response (ie, percentage of patients with confirmed complete response or partial response by use of Response Evaluation Criteria In Solid Tumors [RECIST] criteria. Secondary endpoints were duration of response, time to progression, overall survival, safety, pharmacokinetics, and patient-reported health-related quality of life. This trial is registered on the clinical trials site of the US National Cancer Institute website http://www.clinicaltrials.gov/ct/show/NCT00076011. FINDINGS: In an intention-to-treat analysis, two complete and 21 partial responses were noted, for an objective response rate of 44.2% (95% CI 30.5-58.7). Median response duration was 23.0 months (20.9-not estimable; range 4.2-29.8). However, 12 of 23 initial responders progressed with response duration ranging from 4.2 months to 26.5 months. Additionally, 22 patients showed stable disease for longer than 8 weeks, including 13 patients with stable disease for 24 weeks or longer. Four patients had early disease progression. Three patients had missing response data. Median time to progression was 15.7 months (8.4-23.4, range 0.03-31.5) and median overall survival was 29.9 months (20.3-not estimable; range 2.4-35.8). Treatment-related adverse events included diarrhoea, hypertension, fatigue, nausea, and hoarseness. Treatment-related hypertension occurred in 30 patients and resolved with antihypertensive treatment in all but eight patients, of whom seven patients had a history of hypertension at baseline. INTERPRETATION: Axitinib shows clinical activity in patients with cytokine-refractory metastatic renal-cell cancer. Although 28 patients had grade 3 or grade 4 treatment-related adverse events, these adverse events were generally manageable and controlled by dose modification or supportive care, or both. Further studies are needed to confirm these findings.

105 [202].	Royston, P., M. K. Parmar, et al. (2008). "Visualizing length of survival in time-to-event studies: a complement to Kaplan-Meier plots." J Natl Cancer Inst **100**(2): 92-97.

Because of censoring, standard methods of plotting individual survival times are invalid. Therefore, graphic display of time-to-event data usually takes the form of a Kaplan-Meier survival plot. Kaplan-Meier plots, however, make differences between groups seem larger than they really are. To overcome these limitations, we developed a technique for producing scatter plots with survival data and applied it to data from a randomized trial of patients with renal cancer. As of June 21, 2001, 25 of the 347 patients with kidney cancer in the Medical Research Council RE01 randomized treatment trial for whom data were available had been censored, and the remainder had died. Values of the censored survival times were imputed by assuming a log-normal distribution in survival times and by drawing a random sample given that that each patient with censored data survived at least to the point of censoring. The combined original and imputed data were then examined by use of dot plots and scatter plots. In the RE01 trial, median survival of patients treated with interferon was 3.0 months (95% confidence interval = 0.3 to 5.5 months) longer than that in patients treated with medroxyprogesterone acetate. The Kaplan-Meier analysis showed clear separation between treatment groups and between prognostic groups. In contrast, comparisons of individual observed and imputed survival times between groups of patients showed considerable overlap and gave a more realistic idea of the modest between-group differences than Kaplan-Meier comparisons. These graphs of the distribution of survival times for individuals in each study group, which are simple to produce, may usefully complement Kaplan-Meier plots.

106 [267].	Royston, P., W. Sauerbrei, et al. (2004). "Is treatment with interferon-alpha effective in all patients with metastatic renal carcinoma? A new approach to the investigation of interactions." Br J Cancer **90**(4): 794-799.

The first analysis of the MRC RE01 trial in metastatic renal carcinoma identified a 28% reduction in the hazard of death for patients treated with interferon-alpha compared with medroxyprogesterone acetate (MPA). No subgroup was identified in which treatment with interferon-alpha was more or less effective than MPA. We used a new approach based on fractional polynomials to investigate the updated data from this trial for the possible interaction of treatment with prognostic factors. In the spirit of hypothesis generation, we considered 10 possible prognostic variables, of which white cell count (WCC) was found to influence the effectiveness of interferon treatment. In patients treated with MPA, there was no prognostic effect of WCC, whereas, in patients treated with interferon, the risk of dying increased significantly with WCC level. We defined subgroups of patients based on WCC levels and estimated a hazard ratio of 0.53 in favour of interferon in patients with WCC <6.5 x 10(9), whereas for patients with WCC >10 x 10(9) the risk appears to be similar between the treatment groups, or even slightly raised in the interferon group. Since our results are derived from flexible statistical models, they may be interpreted as a new hypothesis and require validation in independent data.

107 [149].	Rudman, S. M., M. B. Jameson, et al. (2011). "A phase 1 study of AS1409, a novel antibody-cytokine fusion protein, in patients with malignant melanoma or renal cell carcinoma." Clin Cancer Res **17**(7): 1998-2005.

PURPOSE: AS1409 is a fusion protein comprising a humanized antibody BC1 linked to interleukin-12 (IL-12). It is designed to deliver IL-12 to tumor-associated vasculature using an antibody targeting the ED-B variant of fibronectin. EXPERIMENTAL DESIGN: We conducted a phase 1 trial of weekly infusional AS1409 in renal carcinoma and malignant melanoma patients. Safety, efficacy, markers of IL-12-mediated immune response, and pharmacokinetics were evaluated. RESULTS: A total of 11 melanoma and 2 renal cell carcinoma patients were treated. Doses of 15 and 25 mug/kg were studied. Most drug-related adverse events were grade 2 or less, and included pyrexia, fatigue, chills, headache, vomiting, and transient liver function abnormalities. Three dose limiting toxicities of grade 3 fatigue and transaminase elevation were seen at 25 mug/kg. IFN-gamma and interferon-inducible protein-10 (IP-10) were elevated in all patients, indicating activation of cell-mediated immune response; this was attenuated at subsequent cycles. Antidrug antibody responses were seen in all patients, although bioassays indicate these do not neutralize AS1409 activity. Plasma half-life was 22 hours and not dose-dependent. Five patients received 6 cycles or more and a best response of at least stable disease was seen in 6 (46%) patients. Partial response was seen in a melanoma patient, and disease shrinkage associated with metabolic response was maintained beyond 12 months in another melanoma patient despite previous rapid progression. CONCLUSIONS: The maximum tolerated dose was established at 15 mug/kg weekly. AS1409 is well tolerated at this dose. Evidence of efficacy assessed by RECIST, functional imaging, and biomarker response warrants the planned further investigation using this dose and schedule in malignant melanoma.

108 [217].	Ryan, C. W., B. H. Goldman, et al. (2007). "Sorafenib with interferon alfa-2b as first-line treatment of advanced renal carcinoma: a phase II study of the Southwest Oncology Group." J Clin Oncol **25**(22): 3296-3301.

PURPOSE: This phase II study evaluated the activity of combined treatment with interferon alfa-2b and sorafenib, a Raf and multiple receptor tyrosine kinase inhibitor, in patients with advanced renal carcinoma. PATIENTS AND METHODS: Eligible patients had metastatic or unresectable renal carcinoma with a clear-cell component, no prior systemic therapy, performance status 0 to 1, and measurable disease. Treatment consisted of interferon alfa-2b 10 x 10(6) U subcutaneously three times weekly and sorafenib 400 mg orally bid. The primary end point was confirmed Response Evaluation Criteria in Solid Tumors response rate. RESULTS: Twelve (19%) of 62 assessable patients achieved an objective confirmed response. An additional 31 (50%) had an unconfirmed partial response or stable disease as best response. The median progression-free survival was 7 months (95% CI, 4 to 11 months). The most common adverse events were fatigue, anorexia, anemia, diarrhea, nausea, rigors/chills, leukopenia, fever, and transaminase elevation. Von Hippel-Lindau gene mutations were detected in four (22%) of 18 archival tumor specimens. CONCLUSION: The confirmed response rate for the combination of sorafenib and interferon in advanced renal carcinoma is greater than expected with either interferon or sorafenib alone. The toxicity of this combination is dominated by adverse events common to interferon that limit further development of this regimen.

109 [222].	Schenck, M., C. Borgermann, et al. (2007). "[Immunotherapy of metastatic renal cell carcinoma with interleukin-2, interferon-alpha2a and erythropoietin-beta]." Urologe A **46**(5): 528-534.

BACKGROUND: The combination of interferon-alpha2a (IFN-alpha2a) and interleukin-2 (IL-2) induces objective responses in patients with metastatic renal cell carcinoma (MRCC). Anaemia is associated with poor cancer control and reduced quality of life. The aim of the study was to investigate response rate and quality of life in patients with MRCC receiving the combination of Erythropoetin, IFN-alpha2a and IL-2. MATERIAL AND METHOD: Patients with MRCC received epoetin beta (150 IU/kg and haemoglobin <130 g/l or 75 IU/kg and haemoglobin >or=130 g/l) three times weekly, from 14 days before and continuing throughout immunotherapy. In weeks 3-6 the patients received IFN-alpha2a 6 x 10(6) IU/m2 and IL-2 4.5 x 10(6) IU/m2 three times weekly on days 1, 3 and 5. The treatment was repeated two times and in the case of success a third cycle was added. The quality of life was assessed with the FACT questionnaire for fatigue, before, during and after therapy. RESULTS: A total of 21 patients were treated, 19 of whom could be evaluated concerning response, toxicity and quality of life. We observed 1 complete remission, 2 partial remissions, 5 cases of stable disease and 11 with progressive disease. The overall response rate was 16%. Toxicity was mild to moderate; there were no WHO grade III or IV toxicity. The quality of life increased in ten patients, nine of whom exhibited an increase in their haemoglobin during therapy. Five of the nine patients with decreased quality of life also experienced a decrease in their haemoglobin. The correlation of increased haemoglobin and quality of life was significant (p<0.05). CONCLUSION: The combination of IFN-alpha2a, IL-2 and epoetin beta resulted in objective remissions with mild to moderate toxicity. The quality of life correlates significantly with increasing haemoglobin.

110 [147].	Schwandt, A., J. A. Garcia, et al. (2011). "Clinical and immunomodulatory effects of celecoxib plus interferon-alpha in metastatic renal cell carcinoma patients with COX-2 tumor immunostaining." J Clin Immunol **31**(4): 690-698.

INTRODUCTION: Cycloxygenase-2 (COX-2) is an enzyme involved in prostaglandin E2 (PGE(2)) synthesis associated with higher renal cell carcinoma stage. COX-2 inhibition enhances interferon (IFN-alpha) anti-tumor immune effects in pre-clinical models. A phase II trial of celecoxib and IFN-alpha in a targeted population of metastatic renal cell carcinoma patients with maximal COX-2 expression was conducted. METHODS: Cytokine-naive metastatic renal cell carcinoma patients with tumors expressing >/=10% maximal COX-2 staining by immunohistochemistry received IFN-alpha 5 million units daily and celecoxib 400 mg orally twice daily in an open-label, single-arm phase II trial. RESULTS: There were 3 partial responses among 17 patients (objective response rate 18%; 95% confidence interval, 4-43%). Time to progression was 5.6 months. Increased tumor staining 3+ for COX-2 was associated with increased baseline peripheral blood PGE(2) levels, and these patients demonstrated less PGE(2) decrease with therapy. Patients with more 3+ COX-2 staining had significantly more CD3(+) (p = 0.004) and CD4(+) (p = 0.002) IFN-gamma T cells at baseline and a significantly greater decrease in these cells with therapy. DISCUSSION: Celecoxib plus IFN-alpha in renal cell carcinoma (RCC) patients with maximally staining COX-2 tumors does not significantly enhance overall response rates over IFN monotherapy. CONCLUSION: COX-2-expressing RCC demonstrates inherent immunosuppression. COX-2 inhibition with IFN results in minimal immunomodulation and no augmented clinical activity in RCC.

111 [225].	Segota, E., T. Mekhail, et al. (2007). "Phase II trial of capecitabine and rHu-interferon-alpha-2a in patients with metastatic renal cell carcinoma, limited efficacy, and moderate toxicity." Urol Oncol **25**(1): 46-52.

BACKGROUND: Capecitabine is an orally administered fluoropyrimidine that is converted to 5-fluorouracil by thymidine phosphorylase. In view of the recognized synergism of fluoropyrimidines with interferon-alpha (IFNalpha), a Phase II study to characterize the toxicity and efficacy of the combination of capecitabine and rHuIFNalpha-2a for the treatment of patients with renal cell carcinoma (RCC) was conducted. PATIENTS AND METHODS: Eligible patients had metastatic RCC, measurable disease, and no prior systemic therapy. A total of 32 patients were entered into the study. Histologic subtypes included clear cell (n = 28) and nonclear cell (n = 2). Histology was unknown for 2 patients. The first 14 patients were treated with capecitabine 1,000 mg/m(2) twice daily on days 1-14 and 22-36, combined with IFNalpha-2a 3.0 MU/m(2) subcutaneously 3 times weekly. Because of toxicity requiring dose reductions during the first cycle, the capecitabine dose was reduced to 825 mg/m(2) twice daily on days 1-14 and 22-36 in the subsequent 18 patients. RESULTS: Responses were seen in 4 of 32 patients (12%) (95% confidence interval 4% to 29%), with 1 complete response and 3 partial responses. There were 3 responses that occurred at the higher capecitabine starting dose level. Median response duration was 12 months (range 4.6-15.0). There were 12 patients (38%) who had stable disease for at least 2 cycles (duration 2.9 to 33.6+ months). One-year survival was 63%. Toxicity was moderate to severe and required dose reductions in 88% of patients. There were 23 patients who had grade > or =3 toxicity. CONCLUSION: The combination of capecitabine and IFNalpha-2a has limited activity in metastatic RCC and is associated with moderate-to-severe toxicity.

112 [150].	Shek, D., J. Longmate, et al. (2011). "A phase II trial of gefitinib and pegylated IFNalpha in previously treated renal cell carcinoma." Int J Clin Oncol **16**(5): 494-499.

BACKGROUND: This study was conducted to evaluate the efficacy of the epidermal growth factor receptor tyrosine kinase inhibitor gefitinib with pegylated-IFNalpha (PEG-IFNalpha) in patients with advanced renal cell carcinoma. METHODS: Progression-free survival (PFS) rate at 6 months >50% was considered promising for further evaluation. Patients with unresectable or metastatic disease, unlimited prior therapies, and adequate performance status and end-organ function were eligible. PEG-IFNalpha was dosed subcutaneously once weekly (initially 6 mug/kg/week, later reduced to 4 mug/kg/week) for 12 weeks. Gefitinib was given 250 mg orally once daily until progression or intolerance. RESULTS: Twenty-one patients were accrued. Fourteen patients had a prior nephrectomy, and twelve had prior systemic therapy. The 6-month PFS was 29% (95%CI 15-56%). Best responses by RECIST criteria: complete, partial (1, plus 3 unconfirmed) stable (Uhlman et al. Clin Cancer Res 1:913-920, 1995), and progression (Sirotnak et al. Clin Cancer Res 6:4885-4892, 2000). Response duration: complete response (35+ months) and partial response (2, 3, 3, 37 months). Median PFS and overall survival were 5.3 (95%CI 3-10.1) and 13.6 (95%CI 10.3-NA) months, respectively. Most common toxicities included myelosuppression, rash, and nausea. CONCLUSIONS: Although generally well tolerated, gefitinib plus PEG-IFNalpha did not meet the pre-specified 6-month PFS rate >50%. Further evaluation of similar regimens would require appropriate molecular selection of subjects most likely to benefit. Thus, preclinical studies to determine candidate predictive markers for this combination are warranted.

113 [175].	Shinohara, N., A. Kumagai, et al. (2009). "Multicenter phase II trial of combination therapy with meloxicam, a cox-2 inhibitor, and natural interferon-alpha for metastatic renal cell carcinoma." Jpn J Clin Oncol **39**(11): 720-726.

OBJECTIVE: We conducted a Phase II trial to investigate the efficacy of combined therapy with meloxicam, a cyclooxygenase-2 inhibitor and natural interferon (IFN)-alpha in renal cell carcinoma patients with distant metastasis. METHODS: The subjects of this study were patients with untreated renal cell carcinoma who were diagnosed from the results of imaging or pathological studies and who had measurable lesions according to the Response Evaluation Criteria in Solid Tumors (RECIST). Patients could be enrolled in the study irrespective of whether nephrectomy had been performed. Treatment involved the subcutaneous injection of natural IFN-alpha at 3 x 10(6) or 5 x 10(6) U three times weekly plus oral administration of meloxicam at 10 mg once daily. RESULTS: A total of 43 patients were enrolled in the present study, included 11 patients without nephrectomy, 23 patients with a high C-reactive protein (CRP) level and 23 patients with extrapulmonary metastasis. Four patients of complete response and 12 patients of partial response were confirmed, given an overall response rate of 37.2% (95% confidence interval, 23.0-53.3%). Stable disease for 6 months or longer was also obtained in 14 patients. The median time to progression was 14 months. Adverse events (AEs) observed were mainly flu-like symptoms due to cytokine. Although the Grade 3 or 4 AEs were fatigue, hepatic dysfunction, arthritis and gastric ulcer, all but one (gastric ulcer) were immediately improved by discontinuation of this combined therapy. CONCLUSIONS: The combination of meloxicam and natural IFN-alpha is considered to be an active regimen with tolerable toxicities as a first-line treatment of metastatic renal cell carcinoma.

114 [158].	Siebels, M., K. Rohrmann, et al. (2011). "A clinical phase I/II trial with the monoclonal antibody cG250 (RENCAREX(R)) and interferon-alpha-2a in metastatic renal cell carcinoma patients." World J Urol **29**(1): 121-126.

PURPOSE: To evaluate the efficacy and safety of WX-G250, a chimeric monoclonal antibody that binds to carboxy anhydrase IX, combined with low-dose interferon-alpha (LD-IFNalpha) in patients with progressive metastatic renal cell carcinoma (mRCC). PATIENTS AND METHODS: Thirty-one patients, nephrectomized for the primary tumor, clear cell progressive mRCC, were enrolled to receive weekly infusions of WX-G250 (20 mg i.v.; week 2-12) combined with LD-IFNalpha (3 MIU s.c. 3 times/week; week 1-12). At week 16, patients were evaluated for response and stratified into two groups: (a) responders into the extended treatment group for an additional 6 weeks of treatment or (b) the progressive group with no further study treatment. RESULTS: Of the 31 treated patients, 26 were evaluable for response to treatment. Two patients showed partial remission and 14 patients had stable disease as assessed in week 16. One patient experienced partial remission resulting in a complete remission lasting at least 17 months. Nine patients had durable stable disease of 24 weeks or longer. Clinical benefit was obtained in 42% (11/26) patients. The median overall survival achieved was 30 months and the 2-year survival was 57%. Patients receiving extended treatment showed a significantly longer 2-year survival rate than discontinued patients (79 vs. 30%; P=0.0083). In general, treatment was well tolerated with little toxicity. CONCLUSION: Treatment with the antibody WX-G250 in combination with LD-IFNalpha is safe, well tolerated, led to clinically meaningful disease stabilization and demonstrated clinical benefit in this progressive mRCC patient population.

115 [135].	Stein, W. D., J. Wilkerson, et al. (2012). "Analyzing the pivotal trial that compared sunitinib and IFN-alpha in renal cell carcinoma, using a method that assesses tumor regression and growth." Clin Cancer Res **18**(8): 2374-2381.

PURPOSE: We applied a method that analyzes tumor response, quantifying the rates of tumor growth (g) and regression (d), using tumor measurements obtained while patients receive therapy. We used data from the phase III trial comparing sunitinib and IFN-alpha in metastatic renal cell carcinoma (mRCC) patients. METHODS: The analysis used an equation that extracts d and g. RESULTS: For sunitinib, overall survival (OS) was strongly correlated with log g (Rsq = 0.44, P < 0.0001); much less with log d (Rsq = 0.04; P = 0.0002). The median g of tumors in these patients (0.00082 per days; log g = -3.09) was about half that (P < 0.001) of tumors in patients receiving IFN-alpha (0.0015 per day; log g = -2.81). With IFN-alpha, the OS/log g correlation (Rsq = 0.14) was weaker. Values of g from measurements obtained by study investigators or central review were highly correlated (Rsq = 0.80). No advantage resulted in including data from central review in regressions. Furthermore, g can be estimated accurately four months before treatment discontinuation. Extrapolating g in a model that incorporates survival generates the hypothesis that g increased after discontinuation of sunitinib but did not accelerate. CONCLUSIONS: In patients with mRCC, sunitinib reduced tumor growth rate, g, more than did IFN-alpha. Correlating g with OS confirms earlier analyses suggesting g may be an important clinical trial endpoint, to be explored prospectively and in individual patients.

116 [173].	Sunela, K. L., S. Koskinen, et al. (2010). "A phase-II study of combination of pegylated interferon alfa-2a and capecitabine in locally advanced or metastatic renal cell cancer." Cancer Chemother Pharmacol **66**(1): 59-67.

PURPOSE: Combination of capecitabine and interferon has shown activity in metastatic renal cell carcinoma. Pegylated interferons might have more clinical activity and fewer side effects. This study evaluated the efficacy, tolerability, and safety of the combination of capecitabine and pegylated interferon alfa-2a. METHODS: In this open label, single institution, non-randomized phase-II first-line study, 26 patients were included. Capecitabine was administered 2,000 mg/m(2) daily for 14 days followed by 1 week rest. Pegylated interferon alfa-2a was given once as weekly injections with a fixed dose of 180 microg. Overall survival, progression-free survival, and response rates were evaluated; safety and tolerability were monitored. RESULTS: Response rate was 27, with 4% complete responses. Stable disease was achieved in 42%. The treatment discontinued in 4 (15%) patients before first response evaluation because of toxicity. The median progression-free survival was 7.5 months; the median overall survival was 17 months. Grades 3-4 toxicity was seen in 46% of patients, but in 93% of cycles no serious toxicity was experienced. Dose reductions had to be done, but in 81% of cycles intensity of 70% or more was possible. Quality of life was better in cycle five than in the base line. CONCLUSIONS: The combination had moderate, but manageable toxicity. In the future studies, lower dose for capecitabine is recommended. The combination was active and the response rates seen here were in line with phase-II studies on former combinations of non-pegylated interferons. One complete remission was achieved.

117 [266].	Sunkara, U., J. R. Walczak, et al. (2004). "A phase II trial of temozolomide and IFN-alpha in patients with advanced renal cell carcinoma." J Interferon Cytokine Res **24**(1): 37-41.

The combination of temozolomide (TEM) and interferon-alpha (IFN-alpha) previously demonstrated a 30% response rate in metastatic melanoma. A single institution, phase II trial evaluating the efficacy of TEM/IFN in patients with advanced renal cell carcinoma (RCC) was conducted. Safety and tumor response were the main outcomes. Eligible patients received 200 mg/m(2)/day TEM orally on days 1-5 every 28 days, with IFN 2.5 million U/m(2)/day subcutaneously (s.c.) three alternate days/week for days 1-15 first cycle, then 5 million U/m(2)/day s.c. 3 alternate days/week throughout each 28-day cycle. Efficacy was evaluated every 8 weeks, and dose-limiting toxicities (DLTs) were treated with dose reductions of the culprit drug. Sixteen patients (ages 37-67) were initially enrolled. Of the 14 evaluable patients, there was one minor response. Best response was stable disease, with 7 patients remaining on study for > or =6 months. Five were alive for more than 2 years, and 2 remain alive at 45 and 50 months after enrollment. DLTs included TEM-induced myelosuppression and IFN-induced fever/chills. Other toxicities were mild to moderate (grades 1-3). The combination of TEM/IFN proved quite tolerable. This regimen appears inactive in terms of response in this population with poor prognosis, but the patients with stable disease > or =6 months remain of interest.

118 [232].	Tannir, N., E. Jonasch, et al. (2006). "Pilot trial of bone-targeted therapy with zoledronate, thalidomide, and interferon-gamma for metastatic renal cell carcinoma." Cancer **107**(3): 497-505.

BACKGROUND: The purpose of the study was to evaluate the efficacy and safety of a bone-targeted regimen consisting of zoledronate, thalidomide, and interferon-gamma in patients with renal cell carcinoma and bone metastases. METHODS: Eligible patients had radiographic evidence of bone metastasis. Impending pathologic fractures or spinal cord compressions must have been controlled by surgery or radiation therapy before enrollment. Zoledronate (4 mg) was given intravenously every 4 weeks, thalidomide (300 mg) was given orally once a day, and interferon-gamma (100 microg) was given subcutaneously once a week. Patients were evaluated for time to skeletal-related events, the appearance of calcification in osteolytic metastases, and levels of the bone formation/resorption markers. RESULTS.: Fifteen patients were treated between November 2002 and November 2003; 12 had previously undergone surgery, radiation, or embolization for their bone metastases; 11 had more than 3 sites of bone involvement; and 9 also had nonosseous metastases in the lung, liver, lymph node, pancreas, or adrenal gland. The median time to progression was 8.3 weeks (range, 2.1-48 weeks). The median time to a skeletal-related event was 12.0 weeks (range, 3.9-46.4 weeks). Two patients discontinued treatment because of adverse drug reactions (1 deep venous thrombosis and 1 myocardial infarction). Two patients experienced pain improvement and developed calcification in osseous metastases; these patients also showed favorable changes in bone marker levels. CONCLUSIONS: In this pilot study a bone-targeted regimen combining zoledronate, thalidomide, and interferon-gamma was well tolerated and might provide clinical benefit for a small subset of patients with renal cell carcinoma and bone metastases.

119 [227].	Tannir, N. M., L. Cohen, et al. (2006). "Improved tolerability and quality of life with maintained efficacy using twice-daily low-dose interferon-alpha-2b: results of a randomized phase II trial of low-dose versus intermediate-dose interferon-alpha-2b in patients with metastatic renal cell carcinoma." Cancer **107**(9): 2254-2261.

BACKGROUND: In vivo data have shown a more potent antiangiogenic effect and a higher antitumor activity of low-dose interferon (IFN) given twice daily. In a randomized Phase II trial, the authors tested the hypothesis that twice-daily low-dose IFN is more effective than daily intermediate-dose IFN in patients with metastatic renal cell cancer (MRCC). METHODS: A total of 118 patients (59 per arm) were randomly assigned to receive IFN at a dose of 0.5 million units (MU) given subcutaneously twice daily (IFN1) or IFN at a dose of 5 MU given subcutaneously daily (IFN5). The primary endpoint was progression-free survival (PFS). Secondary endpoints included response rate (RR), overall survival (OS), toxicity, and quality of life (QOL). RESULTS: There were no significant differences in either PFS or OS between IFN1 and IFN5 (median of 3.7 months and median of 3.4 months PFS, respectively; median of 25.5 months and median of 17.5 months OS, respectively). The RRs were identical in the 2 arms (6.7%; 95% confidence interval [95% CI], 1.8-16.5%). Two patients, 1 in each arm, remained in complete remission at the time of last follow-up, at 45+ and 38+ months from treatment. Thirty-two patients receiving IFN5 and 19 patients receiving IFN1 experienced Grade 3 or higher adverse events (graded using the National Cancer Institute Common Toxicity Criteria [version 2.0]) (P = .025). Eighteen patients receiving IFN5 and 4 patients receiving IFN1 had dose reductions (P = .002). There was a significant deterioration in QOL and an increase in depression associated with IFN5 but no change was noted with IFN1. CONCLUSIONS: Compared with IFN5, IFN1 is neither more nor less effective but is less toxic, with a better reported QOL. These results may have implications for the design of combination regimens incorporating IFN with targeted agents.

120 [154].	Tatokoro, M., Y. Fujii, et al. (2011). "Phase-II trial of combination treatment of interferon-alpha, cimetidine, cyclooxygenase-2 inhibitor and renin-angiotensin-system inhibitor (I-CCA therapy) for advanced renal cell carcinoma." Cancer Sci **102**(1): 137-143.

We have recently reported favorable responses to a combination treatment comprising cimetidine, a cyclooxygenase-2 inhibitor and a renin-angiotensin-system inhibitor in metastatic renal cell carcinoma (RCC). In view of the potential synergistic effects of these three agents and interferon-alpha (I-CCA therapy), we conducted a phase-II trial to examine the efficacy and toxicity of I-CCA as first-line treatment. Fifty-one patients with advanced RCC received natural interferon-alpha (3-6 million U thrice/week) and cimetidine (800 mg), cyclooxygenase-2 inhibitor meloxicam (10 mg), and renin-angiotensin-system inhibitor candesartan (4 mg) or perindopril (4 mg) orally daily. Memorial Sloan-Kettering Cancer Center prognostic categories were favorable, intermediate and poor in 10 (20%), 31 (61%) and 10 (20%) patients, respectively. The primary end-point was the objective response rate (ORR) and the secondary end-points included clinical benefit, progression-free survival (PFS), overall survival (OS) and safety. Median follow-up was 19 months. Complete response (CR) was observed in four patients (8%) and partial response in seven (14%), yielding an ORR of 22%. None of the four patients who achieved CR relapsed during the 16- to 81-month follow up. The ORR were 17% in the favorable- or intermediate-risk group and 40% in the poor-risk group. The other 24 patients (45%) had stable disease for at least 6 months, resulting in a clinical benefit rate of 67%. The median PFS and OS were 12 and 30 months, respectively. Grade 3/4 toxicities were never observed. The I-CCA therapy, providing favorable responses and low toxicity profiles, is worthy of further consideration as a first-line therapy for metastatic RCC.

121 [146].	Thakur, A., P. Littrup, et al. (2011). "Induction of specific cellular and humoral responses against renal cell carcinoma after combination therapy with cryoablation and granulocyte-macrophage colony stimulating factor: a pilot study." J Immunother **34**(5): 457-467.

Cryotherapy offers a minimally invasive treatment option for the management of both irresectable and localized prostate, liver, pulmonary, and renal tumors. The antineoplastic effects of cryotherapy are mediated by direct tumor lysis and by indirect effects, such as intracellular dehydration, pH changes, and microvascular damage resulting in ischemic necrosis. In this study, we investigated whether percutaneous cryoablation of lung metastasis from renal cell carcinoma (RCC) in combination with aerosolized granulocyte-macrophage colony stimulating factor can induce systemic cellular and humoral immune responses in 6 patients with RCC. Peripheral blood mononuclear cells (PBMCs) were sequentially studied up to 63 days post cryoimmunotherapy (CI). PBMC from pre and post CI were phenotyped for lymphocyte subsets and tested for cytotoxicity and interferon-gamma EliSpots directed at RCC cells. Humoral responses were measured by in vitro antibody synthesis assay directed at RCC cells. The immune monitoring data showed that CI induced tumor specific cytotoxic T lymphocyte, specific in vitro antitumor antibody responses, and enhanced Th1 cytokine production in 4 of 6 patients. More importantly, the magnitude of cellular and humoral antitumor response seems to be associated with clinical responses. These pilot data show that CI can induce robust and brisk cellular and humoral immune responses in patients with metastatic RCC, but requires further evaluation in optimized protocols.

122 [125].	Tsimafeyeu, I., J. S. Zart, et al. (2013). "Cytoreductive radiofrequency ablation in patients with metastatic renal cell carcinoma (RCC) with small primary tumours treated with sunitinib or interferon-alpha." BJU Int **112**(1): 32-38.

OBJECTIVES: To evaluate the role of cytoreductive radiofrequency ablation (cRFA) in patients with metastatic renal cell carcinoma (RCC) with small primary tumours treated with immuno- or targeted therapy. To assess the efficacy of sunitinib in patients with metastatic RCC with unresected small primary tumours. PATIENTS AND METHODS: Three parallel single-arm prospective studies were conducted. Eligibility criteria were nearly identical for all trials and included: histopathologically confirmed RCC; metastatic measurable disease; size of primary tumour <5 cm; good or intermediate prognosis according to the Memorial Sloan-Kettering Cancer Center model; and no previous therapy. Study 1: Patients were treated with percutaneous cRFA under computed tomography guidance followed by interferon (IFN)-alpha, 9 MIU, s.c., three times per week. Study 2: Patients received cRFA followed by sunitinib in repeated 6-week cycles of 50 mg/day orally for 4 weeks, then 2 weeks off treatment. Study 3: Patients with unresected primary RCC received sunitinib alone. The primary endpoint was progression-free survival (PFS). RESULTS: Baseline patient characteristics (age, gender, histology, Eastern Cooperative Oncology Group performance status, metastatic sites, primary tumour size) were similar in all three studies. Efficacy data for 114 evaluable patients showed an objective response rate of 8% (95% confidence interval [CI] 4.5, 10.5) for study 1, 28.9% (95% CI 15.2, 34) for study 2, and 31.6% (95% CI 20.3, 38.9) for study 3. The median (95% CI) PFS times were 9.1 (6.9, 10.2), 13.4 (9.8, 14.4) and 12.7 (11.3, 13.5) months for studies 1, 2 and 3, respectively. Objective response rate was significantly higher and PFS significantly longer in the sunitinib trials than in study 1 (P < 0.01 all differences); no differences were found between studies 2 and 3 (objective response rate, P = 0.1; PFS, P = 0.6). Study 1 met its primary endpoint, showing that PFS was significantly longer than the expected 5 months (P = 0.02). The median (95% CI) objective survival (OS) times were greater in study 2 (cRFA/sunitinib) and study 3 (sunitinib-alone) than in study 1 (IFN-alpha) at 27.2 (22.6, 31.8) and 22.5 (20.7, 24.3) vs 19.5 (16.3, 22.7) months, respectively. Differences were significant (study 1 vs 2, hazard ratio [HR] = 0.55; P = 0.003; study 1 vs study 3 HR = 0.6, P = 0.01). OS was significantly longer in the cRFA/sunitinib group compared with the sunitinib-alone group (HR = 0.71; P = 0.04). There were no unexpected toxicities of medical treatment or complications of cRFA. CONCLUSIONS: cRFA is a safe and effective approach for select patients with metastatic RCC treated with immunotherapy. The cRFA technique did not improve PFS in patients treated with sunitinib; cRFA probably has impact on OS in these patients. This needs to be tested in a larger trial. Sunitinib was effective in patients with metastatic RCC with unresected small primary tumours.

123 [228].	Vaishampayan, U. N., L. K. Heilbrun, et al. (2007). "Phase II trial of interferon and thalidomide in metastatic renal cell carcinoma." Invest New Drugs **25**(1): 69-75.

OBJECTIVES: To evaluate the toxicity and efficacy of interferon and thalidomide combination in a phase II clinical trial. PATIENTS AND METHODS: Eligibility included metastatic renal cancer with a maximum of two prior regimens, performance status of 0-2 and adequate renal, hepatic and bone marrow function. RESULTS: Twenty patients were enrolled on this phase II trial. Median age was 60.5 years (Range: 39-75 years). 17 patients had visceral metastases (lung/liver/both) and 3 patients had lymph node only metastases. A total of 26 cycles of 4 weeks each were administered; median of 1 cycle and range from 0-9 cycles. The therapy was poorly tolerated with grade 3 adverse events noted in 12 (60%) of the 20 patients. No objective responses were noted. Of the 14 response evaluable patients, one had an unconfirmed response (38% decrease in size) and one had prolonged disease stabilization for 10 months. The median time to progression was 1.0 month and median survival was 2.8 months. Pre and post therapy PET scans were performed nine weeks apart on one patient. The mean standardized uptake values (SUV) declined from 1.45 (SUV min-max 0.89-1.76) to 1.12 (SUV min-max 0.55-1.47), denoting anti vascular effect. The patient did not have an objective response but had a disease stabilization sustained for 10 months. CONCLUSION: The combination of interferon and thalidomide has minimal efficacy and considerable toxicity which makes this combination unworthy of future investigation in metastatic renal cancer.

124 [145].	Walter, B., I. Schrettenbrunner, et al. (2012). "Pioglitazone, etoricoxib, interferon-alpha, and metronomic capecitabine for metastatic renal cell carcinoma: final results of a prospective phase II trial." Med Oncol **29**(2): 799-805.

We enrolled 45 patients with metastatic renal cell carcinoma (RCC) at a progressive disease between March 2003 and April 2008 to assess the impact of an anti-inflammatory treatment regime in combination with metronomic low-dose chemotherapy. 42% of the patients had been systemically pre-treated. Therapy consisted of etoricoxib 60 mg daily plus pioglitazone 60 mg daily, day 1+, low-dose interferon-alpha 4.5 MU sc three times a week, week 1+ and low-dose capecitabine 1 g/m(2) twice daily orally for 14 days, every 3 weeks, day 1+, until disease progression. Objective response was observed in 35% of the patients (PR 27, CR 9%), which was paralleled by strong CRP decline for all patients with initially elevated CRP levels (n = 32). CRP values decreased from mean 42.3 mg/L (range 9.1-236), to 11.1 mg/L, (range 1.1-35.6), P = 0.006. Median overall survival and progression-free survival for the total cohort were 26.9 and 7.2 months for patients with elevated CRP 24.4 and 11.3 months (95% CI, 22.8-31.0/5.7-16.9) and 13.8-2.6 months (95% CI, 6.5-21.1/0.4-4.8) for the non-elevated CRP group, respectively (P = 0.082/0.017). Median observation time: 26.1 months; Overall survival at 5 years: 18%. Toxicity>WHO grade 3 was reported: Hand-foot syndrome in 16 patients (36%), diarrhea in 4, and pneumonia in 2 patients. Our data allow us to conclude that the control of tumor-associated inflammation is an important therapeutic principle in patients with metastatic RCC.

125 [181].	Westermann, J., A. C. Hecker, et al. (2009). "Granulocyte macrophage-colony stimulating factor plus interleukin-2 plus alpha-interferon plus 5-fluorouracil in the treatment of metastatic renal cell cancer: induction of CD80/86+ T cells indicates adverse outcome." J Immunother **32**(6): 667-675.

Even in the era of multitargeted therapies, cytokines remain at least one of different treatment options in renal cell cancer (RCC), particularly for patients belonging to the good prognostic risk category according to Memorial Sloan Kettering Cancer Center criteria. Granulocyte macrophage-colony stimulating factor plays a central role in the differentiation and activation of antigen presenting cells. This clinical phase 1/2 chemoimmunotherapy trial in metastatic RCC used sequential application of alpha-interferon /5-fluorouracil followed by granulocyte macrophage-colony stimulating factor/interleukin-2. The study was performed before multikinase inhibitors were available for routine use. Twenty patients with metastatic RCC were enrolled into this phase 1/2 protocol. Sequential chemoimmunotherapy was feasible and safe on an outpatient basis. The regimen had only modest antitumor activity with 4 mixed responses and 4 stable diseases being documented after 4 treatment cycles. Enhanced proliferative and stimulatory capacity of peripheral blood mononuclear cells was only observed in patients with mixed responses/stable diseases whereas patients with progressive disease did not show any change. Most interestingly, there was a significant increase of T cells expressing the costimulatory molecules CD80/86 in patients with progressive disease. This finding is reported here for the first time under chemoimmunotherapy of RCC. In conclusion, clinical response rates of this cytokine-based regimen do not justify further clinical evaluation. However, the study suggests that CD80/86+ T cells might have negative regulatory function under cytokine treatment and are possibly useful as a negative predictive marker for clinical response.

126 [159].	Yang, S., P. de Souza, et al. (2010). "Quality of life in patients with advanced renal cell carcinoma treated with temsirolimus or interferon-alpha." Br J Cancer **102**(10): 1456-1460.

BACKGROUND: Temsirolimus was approved in Europe as first-line treatment of poor-prognosis advanced renal cell carcinoma (advRCC) based on significant clinical benefits. METHODS: Patients with advRCC and multiple poor-prognostic factors were randomly assigned to receive 25 mg intravenous temsirolimus weekly, interferon-alpha (titrated to 18 mU) three times weekly, or 15 mg intravenous temsirolimus weekly plus 6 mU of interferon-alpha three times weekly. EuroQol-5D utility score (EQ-5D index) and the EQ-5D visual analogue scale (EQ-VAS) responses were recorded. For analysis, patients were required to have their EQ-5D data recorded at baseline, week 12, and last visit after week 12. The analysis was conducted using last-visit data and a repeated-measures mixed-effect (RMME) model to evaluate quality-of-life differences between temsirolimus and interferon-alpha, controlling for baseline covariates. RESULTS: Average EQ-5D score at the last measure was significantly higher in patients receiving temsirolimus compared with interferon-alpha: by 0.10 on EQ-5D index (P=0.0279) and by 6.61 on EQ-VAS (P=0.0095). In the RMME model, the least-square mean for on-treatment EQ-5D index score was 0.590 with temsirolimus and 0.492 with interferon-alpha (P=0.0022). CONCLUSION: Temsirolimus is associated with significantly higher EQ-5D scores compared with interferon-alpha in patients with previously untreated poor-prognosis advRCC.

127 [157].	Zbrozek, A. S., G. Hudes, et al. (2010). "Q-TWiST analysis of patients receiving temsirolimus or interferon alpha for treatment of advanced renal cell carcinoma." Pharmacoeconomics **28**(7): 577-584.

BACKGROUND AND OBJECTIVES: For patients with advanced cancers, it is important that treatment improves the quality as well as the quantity of survival. This quality-adjusted time without symptoms of progression or toxicity (Q-TWiST) analysis provides a combined measure of both the overall survival interval and the quality of survival for patients with advanced renal cell carcinoma (RCC) receiving temsirolimus, interferon (IFN)-alpha or the combination of these agents, using data from a phase III clinical trial. METHODS: Overall survival was partitioned into three distinct health states: time with serious toxicity (TOX), time after progression (REL) and time without symptoms of progression or toxicity (TWiST). Health states were quality weighted by patient-reported EQ-5D measures collected while receiving treatment. RESULTS: All 626 patients from the trial were included in computation of health-state durations. EQ-5D questionnaires were obtained from 260 patients upon progression and from 230 after a grade 3 or 4 adverse event, and from 278 patients in the TWiST state. Patients receiving temsirolimus had 38% longer TWiST than those receiving IFNalpha (6.5 vs 4.7 months, respectively; p = 0.0005). Patients receiving temsirolimus had 25% longer quality-adjusted survival in terms of Q-TWiST than those receiving IFNalpha (7.0 vs 5.6 months, respectively; p = 0.0015). Differences between the combination (temsirolimus + IFNalpha) and IFNalpha groups were not statistically significant. Threshold utility analysis indicated that temsirolimus was the preferred alternative for all possible utility weights for REL and TOX health states. CONCLUSION: Temsirolimus resulted in significantly longer Q-TWiST (quality-adjusted survival) in patients with advanced RCC than IFNalpha therapy.

128 [148].	Zurita, A. J., E. Jonasch, et al. (2012). "A cytokine and angiogenic factor (CAF) analysis in plasma for selection of sorafenib therapy in patients with metastatic renal cell carcinoma." Ann Oncol **23**(1): 46-52.

BACKGROUND: We investigated cytokines and angiogenic factors (CAFs) in patients with metastatic renal cell carcinoma (mRCC) treated in a randomized phase II clinical trial of sorafenib versus sorafenib+ interferon-alpha (IFN-alpha) that yielded no differences in progression-free survival (PFS). We aimed to link the CAF profile to PFS and select candidate predictive and prognostic markers for further study. METHODS: The concentrations of 52 plasma CAFs were measured pretreatment (n = 69), day 28, and day 56 using multiplex bead arrays and enzyme-linked immunosorbent assay. We investigated the association between baseline levels of CAFs with PFS and posttreatment changes. RESULTS: Unsupervised CAF clustering analysis revealed two distinct mRCC patient groups with elevated proangiogenic or proinflammatory mediators. A six-marker baseline CAF signature [osteopontin, vascular endothelial growth factor (VEGF), carbonic anhydrase 9, collagen IV, VEGF receptor-2, and tumor necrosis factor-related apoptosis-inducing ligand] correlated with PFS benefit (hazard ratio 0.20 versus 2.25, signature negative versus positive, respectively; P = 0.0002). While changes in angiogenic factors were frequently attenuated by the sorafenib+ IFN combination, most key immunomodulatory mediators increased. CONCLUSIONS: Using CAF profiling, we identified two mRCC patient groups, a candidate plasma signature for predicting PFS benefit, and distinct marker changes occurring with each treatment. This platform may provide valuable insights into renal cell carcinoma biology and the molecular consequences of targeted therapies.

# TOPIC: [Kidney Cancer or Renal Cancer] and IL-2

1 [352].	Ahmadzadeh, M. and S. A. Rosenberg (2006). "IL-2 administration increases CD4+ CD25(hi) Foxp3+ regulatory T cells in cancer patients." Blood **107**(6): 2409-2414.

Interleukin-2 (IL-2) is historically known as a T-cell growth factor. Accumulating evidence from knockout mice suggests that IL-2 is crucial for the homeostasis and function of CD4+ CD25+ regulatory T cells in vivo. However, the impact of administered IL-2 in an immune intact host has not been studied in rodents or humans. Here, we studied the impact of IL-2 administration on the frequency and function of human CD4+ CD25(hi) T cells in immune intact patients with melanoma or renal cancer. We found that the frequency of CD4+ CD25(hi) T cells was significantly increased after IL-2 treatment, and these cells expressed phenotypic markers associated with regulatory T cells. In addition, both transcript and protein levels of Foxp3, a transcription factor exclusively expressed on regulatory T cells, were consistently increased in CD4 T cells following IL-2 treatment. Functional analysis of the increased number of CD4+ CD25(hi) T cells revealed that this population exhibited potent suppressive activity in vitro. Collectively, our results demonstrate that administration of high-dose IL-2 increased the frequency of circulating CD4+ CD25(hi) Foxp3+ regulatory T cells. Our findings suggest that selective inhibition of IL-2-mediated enhancement of regulatory T cells may improve the therapeutic effectiveness of IL-2 administration.

2 [371].	Aklilu, M., W. M. Stadler, et al. (2004). "Depletion of normal B cells with rituximab as an adjunct to IL-2 therapy for renal cell carcinoma and melanoma." Ann Oncol **15**(7): 1109-1114.

BACKGROUND: We postulated that in patients with metastatic renal cell carcinoma (RCC) or melanoma, depletion of normal B cells using the anti-CD20 mAb rituximab before treatment with low-dose interleukin (IL)-2 would improve clinical outcome. PATIENTS AND METHODS: Rituximab (375 mg/m(2)) weekly for 4 weeks. IL-2 [11 (million units) daily] s.c., 4 days a week for weeks 5-8, followed by a 2-week rest (weeks 9 and 10). Patients without disease progression continued on IL-2. Disease re-evaluation was performed after rituximab and after every course of IL-2. RESULTS: Fifteen patients with RCC and six with melanoma were enrolled. One patient had a partial response and seven patients had stable disease. Toxicities were similar to those expected with IL-2 alone, and there were no grade 4 events. Circulating B cells were depleted in all patients. The subsequent low-dose IL-2 increased absolute numbers of natural killer cells, activated CD4(+) and activated CD8(+) T cells. Expanded T cells produced interferon-gamma, but not IL-4. Proliferation of peripheral blood lymphocytes to phytohemagglutinin was diminished following rituximab treatment, suggesting that B cells participate in this response in vitro. CONCLUSIONS: Our results suggest that depletion of circulating B cells with rituximab does not increase the response rate, alter the toxicity profile or change the biological activity in response to low-dose IL-2 in patients with RCC or melanoma.

3 [313].	Amato, R. J., R. Malya, et al. (2008). "Phase II study of combination thalidomide/interleukin-2 therapy plus granulocyte macrophage-colony stimulating factor in patients with metastatic renal cell carcinoma." Am J Clin Oncol **31**(3): 237-243.

OBJECTIVE: The early efficacy and safety findings observed with thalidomide plus low-dose Interleukin-2 (IL-2) combination therapy for the treatment of metastatic renal cell carcinoma (MRCC) formed the foundation for this study. Granulocyte macrophage-colony stimulating factor (GM-CSF) is an important cytokine for priming cellular immune responses. This study assessed whether GM-CSF would improve the response rate of MRCC patients to the thalidomide plus IL-2 regimen. METHODS: Thirty-one patients with progressive MRCC without prior treatment were enrolled. They received initial doses of thalidomide 200 mg on day 1 (escalated to 400 mg after the first 48 hours), and fixed doses of IL-2 at 7 mIU/m2 and GM-CSF at 250 microg/m2 by subcutaneous injection on days 1 to 5 in weeks 2 to 5, followed by a 2-week rest. After the initial 7-week course, patients received up to 6 subsequent 6-week courses. RESULTS: Seventeen (55%) patients experienced disease control, including 3 (10%) complete responses, 8 (26%) partial responses, and 6 (19%) cases of stable disease. Disease progression was observed in 14 (45%) patients. Survival ranged from 1 to 30+ months. Toxicities included somnolence, nausea, constipation, rash, flu-like symptoms, fluid retention, hypotension, and neuropathy. CONCLUSION: Thalidomide plus IL-2 in combination with GM-CSF is tolerable and produces durable responses in patients with MRCC. GM-CSF, however, did not produce a response rate superior to that reported in previous studies of combination thalidomide/IL-2 therapy. Further development of the thalidomide plus IL-2 combination therapy will address patients who have received molecular-targeted agents, such as sunitinib and sorafenib, as first- or second-line therapy.

4 [349].	Amato, R. J., M. Morgan, et al. (2006). "Phase I/II study of thalidomide in combination with interleukin-2 in patients with metastatic renal cell carcinoma." Cancer **106**(7): 1498-1506.

BACKGROUND: The purpose of the study was to determine, in a Phase I/II study, the efficacy and safety profile of thalidomide with interleukin-2 (IL-2) in patients with metastatic renal cell carcinoma (MRCC). METHODS: Fifteen patients (8 of whom were previously treated) enrolled in Phase I were treated with escalating doses of oral thalidomide (200-600 mg) and a fixed dose of IL-2 (7 mIU/m(2)) by subcutaneous injection. A course was 6 weeks, with the exception of Course 1, which was 7 weeks. Thirty-seven Phase II patients who had not received prior chemotherapy or immunotherapy for renal cell carcinoma (RCC) received an initial thalidomide dose of 200 mg at Week 0, which was escalated to 400 mg after 48 hours. Subcutaneous IL-2 was administered at the same fixed daily dose used in Phase I. RESULTS: Fifty-one of 52 Phase I/II patients were evaluable. Twenty-seven patients (52%) experienced disease control, including 4 (8%) complete responses, 15 (29%) partial responses, and 8 (15%) cases of stable disease. Disease progression was observed in 24 patients (47%). Survival in the 2 phases ranged from 4 weeks to 45.2+ months. At the time of last follow-up, 2 of 51 patients (4%) remained on maintenance thalidomide therapy and continue to be followed. Three of the 51 patients with CRs (6%) ceased thalidomide therapy at 23-25 months and have maintained their responses to date. One complete responder was lost to follow-up. As of January 2005, 14 of 51 patients (27%) remained alive. Toxicities were mild to moderate, including Grade 1 to 2 somnolence, constipation, neuropathy, rash, flu-like symptoms, fluid retention, hypotension, and hypothyroidism (according to version 2.0 of National Cancer Institute Common Toxicity Criteria). In addition, two patients experienced deep venous thrombosis. CONCLUSIONS: Thalidomide in combination with IL-2 is tolerable and can produce durable, active responses in patients with MRCC. To evaluate the merits of thalidomide as a valuable agent against MRCC and to more fully determine the efficacy of thalidomide/IL-2 combination therapy, the scrutiny of Phase III testing is required. Further development of thalidomide/IL-2 combination therapy will be the focus of this group.

5 [354].	Attia, P., A. V. Maker, et al. (2005). "Inability of a fusion protein of IL-2 and diphtheria toxin (Denileukin Diftitox, DAB389IL-2, ONTAK) to eliminate regulatory T lymphocytes in patients with melanoma." J Immunother **28**(6): 582-592.

Elimination of regulatory T lymphocytes may provide a way to break self-tolerance and unleash the anti-tumor properties of circulating lymphocytes. The use of fusion proteins, which link cytotoxic molecules to receptor targets, provides one approach to this problem. This study examined the ability of a fusion protein of interleukin-2 (IL-2) and diphtheria toxin (Denileukin Diftitox, DAB389IL-2, ONTAK) to eliminate regulatory T lymphocytes based on their expression of high-affinity IL-2 receptors. Thirteen patients (12 with metastatic melanoma, 1 with metastatic renal cell carcinoma) were treated at one of the two Food and Drug Administration-approved doses of Denileukin Diftitox (seven patients at 9 microg/kg, six patients at 18 microg/kg). None of the patients experienced an objective clinical response. Foxp3 expression did not decrease significantly overall, although it did decrease minimally among patients receiving 18 microg/kg (-2.01+/-0.618 copies of Foxp3/10(3) copies of beta-actin; P=0.031). Denileukin Diftitox did not decrease the suppressive ability of CD4CD25 cells as quantified by an in vitro co-culture suppression assay. Furthermore, the increased numbers of lymphocytes in patients resulting from treatment with IL-2 were not susceptible to Denileukin Diftitox. Administration of Denileukin Diftitox does not appear to eliminate regulatory T lymphocytes or cause regression of metastatic melanoma.

6 [294].	Berntsen, A., M. K. Brimnes, et al. (2010). "Increase of circulating CD4+CD25highFoxp3+ regulatory T cells in patients with metastatic renal cell carcinoma during treatment with dendritic cell vaccination and low-dose interleukin-2." J Immunother **33**(4): 425-434.

Regulatory T cells (Treg) play an important role in the maintenance of immune tolerance and may be one of the obstacles of successful tumor immunotherapy. In this study, we analyzed the impact of administration of dendritic cell (DC) vaccination in combination with low-dose interleukin (IL)-2 in patients with metastatic renal cell carcinoma on the frequency of CD4+CD25highFoxp3+ Treg cells in peripheral blood. We found that the treatment increased the frequency of Treg cells more than 7-fold compared with pretreatment levels (P<0.0001). The frequency of Treg cells decreased when patients had been off IL-2 treatment for only 8 days, but remained higher than pretreatment levels. A functional assay showed that isolated Treg cells were capable of inhibiting proliferation of responder cells. Also, in vitro studies showed that coculture of mature DCs, autologous T cells and IL-2 leads to an increase in the number of Treg cells whereas IL-21 does not stimulate the induction of Treg cells. These findings demonstrate that even low doses of IL-2 in combination with DC vaccination are able to expand CD4+CD25+Foxp3+ Treg cells in vivo in metastatic renal cell carcinoma patients. Further, the results indicate that the IL-2-induced effect on Treg cells is reversible and declines shortly after termination of IL-2 treatment. Our data suggest that approaches combining DC-mediated immunotherapy and depletion of Treg cells may be necessary to enhance the ability of vaccination therapy to elicit effective antitumor responses in cancer patients. Also, adjuvant IL-21 administration may lead to immune enhancement without simultaneous induction of Treg cells.

7 [350].	Bleumer, I., E. Oosterwijk, et al. (2006). "A clinical trial with chimeric monoclonal antibody WX-G250 and low dose interleukin-2 pulsing scheme for advanced renal cell carcinoma." J Urol **175**(1): 57-62.

PURPOSE: WX-G250 is a chimeric monoclonal antibody that binds to carbonic anhydrase IX(G250/MN), which is present on greater than 95% of RCCs of the clear cell subtype. The suggested working mechanism of WX-G250 is by ADCC. Because the number of activated ADCC effector cells can be increased by a low dose interleukin-2 pulsing schedule, a multicenter study was initiated to investigate whether WX-G250 combined with LD-IL-2 could lead to an improved clinical outcome in patients with progressive RCC. MATERIALS AND METHODS: A total of 35 patients with progressive clear cell RCC received weekly infusions of WX-G250 for 11 weeks combined with a daily LD-IL-2 regimen. Patients were monitored longitudinally for ADCC capacity. Radiological assessment of metastatic lesions was performed at week 16 and regularly until disease progression. RESULTS: A durable clinical benefit was achieved in 8 of 35 patients (23%), including 3 with a partial response and 5 with stabilization at 24 weeks or greater. Mean survival was 22 months. In general treatment was well tolerated with little toxicity. The number of effector cells increased during treatment but lytic capacity per cell did not increase. ADCC and clinical outcome did not appear to correlate. CONCLUSIONS: WX-G250 combined with LD-IL-2 in patients with metastatic RCC is safe and well tolerated. With a substantial clinical benefit and a median survival of 22 months in patients with metastatic RCC who have progressive disease at study entry combination therapy showed increased overall survival compared to WX-G250 monotherapy. Survival was at least similar to that of currently used cytokine regimens but with a favorable toxicity profile.

8 [271].	Dandamudi, U. B., M. Ghebremichael, et al. (2013). "A phase II study of bevacizumab and high-dose interleukin-2 in patients with metastatic renal cell carcinoma: a Cytokine Working Group (CWG) study." J Immunother **36**(9): 490-495.

Overexpression of vascular endothelial growth factor in renal cell carcinoma (RCC) leads to angiogenesis, tumor progression, and inhibition of immune function. We conducted the first phase II study to estimate the efficacy and safety of bevacizumab with high-dose interleukin-2 (IL-2) therapy in patients with metastatic RCC. Eligible patients had predominantly clear cell metastatic RCC, measurable disease, a Karnofsky Performance Status of >/=80%, and adequate end-organ function. IL-2 (600,000 IU/kg) was infused intravenously every 8 hours (maximum 28 doses) during two 5-day cycles on days 1 and 15 of each 84-day course. Bevacizumab (10 mg/kg) was infused intravenously every 2 weeks beginning 2 weeks before initiating IL-2. Fifty of 51 eligible patients from 8 centers were enrolled. Median progression-free survival (PFS) was 11.2 months (90% confidence interval, 5.7-17.7), and 2-year PFS was 18% (90% confidence interval, 8%-27%). Responses included 4 complete (8%) and 11 partial (22%) responses. Toxicities did not exceed those expected from each agent alone. Combining IL-2 plus bevacizumab is feasible, with a response rate and PFS at least as high as reported previously for the single agents. The regimen did not appear to enhance the rate of durable major responses over that of IL-2 alone.

9 [356].	Donskov, F., M. Middleton, et al. (2005). "Two randomised phase II trials of subcutaneous interleukin-2 and histamine dihydrochloride in patients with metastatic renal cell carcinoma." Br J Cancer **93**(7): 757-762.

Histamine inhibits formation and release of phagocyte-derived reactive oxygen species, and thereby protects natural killer and T cells against oxidative damage. Thus, the addition of histamine may potentially improve the efficacy of interleukin-2 (IL-2). Two randomised phase II trials of IL-2 with or without histamine dihydrochloride (HDC) in patients with metastatic renal cell carcinoma (mRCC) were run in parallel. A total of 41 patients were included in Manchester, UK and 63 in Aarhus, Denmark. The self-administered, outpatient regimen included IL-2 as a fixed dose, 18 MIU s.c. once daily, 5 days per week for 3 weeks followed by 2 weeks rest. Histamine dihydrochloride was added twice daily, 1.0 mg s.c., concomitantly with IL-2. A maximum of four cycles were given. The Danish study showed a statistically significant 1-year survival benefit (76 vs 47%, P = 0.03), a trend towards benefit in both median survival (18.3 vs 11.4 months, P = 0.07), time to PD (4.5 vs 2.2 months, P = 0.13) and clinical benefit (CR + PR + SD) (58 vs 37%, P = 0.10) in favour of IL-2/HDC, whereas the UK study was negative for all end points. Only three patients had grade 4 toxicity; however, two were fatal. A randomised phase III trial is warranted to clarify the potential role of adding histamine to IL-2 in mRCC.

10 [314].	Dudek, A. Z., M. F. Mescher, et al. (2008). "Autologous large multivalent immunogen vaccine in patients with metastatic melanoma and renal cell carcinoma." Am J Clin Oncol **31**(2): 173-181.

OBJECTIVE: To evaluate the safety and activity of large multivalent immunogen (LMI), prepared by immobilizing autologous tumor cell plasma membrane on 5-microm diameter silica beads, in patients with melanoma and renal cell carcinoma (RCC). METHODS: Thirty patients with stage IV metastatic melanoma and 31 patients with stage IV RCC were randomly assigned to 1 of 3 trial arms and received monthly treatment with (1) LMI alone, (2) cyclophosphamide followed 8 days later with LMI, or (3) the same treatment as in arm 2 with IL-2 given for 5 days beginning 1 week after LMI administration. RESULTS: No grade 4 toxicities were observed. For patients with melanoma, median overall survival time for all 30 patients was 20.4 months [95% confidence interval (CI): 8.0-not assessable], and median progression-free survival was 2.8 months (95% CI: 1.9-6.3). For patients with RCC, median overall survival exceeded 46.2 months (95% CI: 30.3-not assessable), and median progression-free survival was 12.2 months (95% CI: 4.6-not assessable). Two patients had a partial response to LMI treatment. CONCLUSIONS: Based on our results that demonstrate the safety and tolerability of LMI vaccine, further development of this therapy is warranted to evaluate its clinical efficacy.

11 [334].	Esteban-Gonzalez, E., J. Carballido, et al. (2007). "Retrospective review in patients with pulmonary metastases of renal cell carcinoma receiving inhaled recombinant interleukin-2." Anticancer Drugs **18**(3): 291-296.

Pulmonary metastases of renal cell carcinoma are associated with poor prognosis. Systemic interleukin-2 is used to treat pulmonary metastases of renal cell carcinoma; however, its toxicity limits its use. The objective of this study was to evaluate the efficacy and safety of inhaled interleukin-2 in pulmonary metastases of renal cell carcinoma patients. The study was designed as a retrospective chart review in pulmonary metastases of renal cell carcinoma patients treated with inhaled interleukin-2. Between 2000 and 2004, 19 centres in Spain and two in Portugal recruited 51 patients. The treatment schedule was as follows: three cycles of 36 MIU interleukin-2 per day for 5 days/week for 12 weeks (with 1 treatment-free week between cycles) in Spain and for 3 weeks (out of each 4 weeks) for 12 weeks in Portugal. Efficacy was assessed by best response following each treatment cycle and at final evaluation. Kaplan-Meier method was used to estimate progression-free survival and overall survival. Safety data were analysed using descriptive statistics, with toxicities expressed in number of weeks, which were reported. Overall objective response rate was 13.7% (95% confidence interval: 5.7-26.3). Median progression-free survival and overall survival were 8.6 (95% confidence interval: 3.45-16.5) and 23 (95% confidence interval: 11.5-34.5) months. The most common toxicities were cough (40% of cycles) and fatigue (7%). The majority of weeks of toxicities were reported to be only grade 1 or 2 in severity. Inhaled interleukin-2 shows efficacy and mild toxicity of pulmonary metastases of renal cell carcinoma patients, and might be considered as an alternative treatment to the systemic administration of this drug in these patients.

12 [305].	Filippetti, M., A. Torsello, et al. (2009). "[IL-2 bronchoscopic istillation and immune cell activation: preliminary results of the BRIIL-2 study for treatment of pulmonary metastasis from renal cancer and melanoma]." Clin Ter **160**(2): 139-143.

BRIIL-2 is a clinical study for evaluation of efficacy and toxicity of third line treatment of pulmonary metastasis from renal cancer and melanoma with flexible bronchoscopic istillation of IL-2. Moreover, we evaluate local (BALT) and peripheral lymphocytic activation during this IL-2 administration. Up today we enrolled two patients with pulmonary metastasis from renal cancer already treated with two lines of molecular therapy, chemotherapy or systemic immunotherapy. Regarding to immunologic stimulation, lymphocytic fraction decreased from 21 to 2% in the first and from 10.5 to 6% in the second patient, indicating lymphocytic enrollment for activation, while TCD4/CD8 ratio is stable. In both patients we also observed a significant increase of HLA-DR in T lymphocytes (CD3) either in BAL or in peripheral blood. No significant major toxicities were observed after broncho-istillation, even if the dose was progressively increased. Thus IL-2 broncho-istillation could represent a valid administration modality to obtain an effective immunologic stimulation either local or systemic.

13 [369].	Galanis, E., P. A. Burch, et al. (2004). "Intratumoral administration of a 1,2-dimyristyloxypropyl-3- dimethylhydroxyethyl ammonium bromide/dioleoylphosphatidylethanolamine formulation of the human interleukin-2 gene in the treatment of metastatic renal cell carcinoma." Cancer **101**(11): 2557-2566.

BACKGROUND: Leuvectin (Vical Inc., San Diego, CA) is a gene transfer product in which a plasmid encoding the human interleukin-2 (IL-2) gene is complexed with the cationic lipid 1,2-dimyristyloxypropyl-3-dimethylhydroxyethyl ammonium bromide/dioleoylphosphatidylethanolamine (DMRIE/DOPE). In the current study, the authors investigated the safety and efficacy of in situ vaccination with Leuvectin in patients with metastatic renal cell carcinoma. METHODS: Thirty-one patients with metastatic renal cell carcinoma were treated with intratumorally administered Leuvectin at doses ranging from 0.75 to 4 mg. These patients subsequently were evaluated for response and for treatment-related toxicity. RESULTS: Treatment was well tolerated: no Grade 3 or 4 toxicities were observed in association with the study agent. Documented side effects included Grade 1 pain at the injection site (20%); mild (i.e., Grade 1 or 2) constitutional symptoms, including malaise/myalgia, low-grade fever, and chills (74%); Grade 1 fatigue (19%); Grade 1 or 2 nausea (10%); and Grade 2 allergy (1 occurrence). Two patients experienced partial responses, which endured for 32 months and 6 years, respectively, and 1 patient currently is experiencing a pathologic complete response, which, to date, has persisted for 50 months; thus, the overall response rate was 10%. In addition, 7 patients (23%) experienced disease stabilization for a median of 8 months (range, 4-48 months). The median duration of survival from the start of Leuvectin treatment was 11 months (range, 2-72 months), with a 1-year survival rate of 48% and a 3-year survival rate of 19%. Laboratory analysis of tumor samples revealed the presence of IL-2 plasmid DNA in six of eight patients posttreatment, increased IL-2 expression in tumor cells in four of eight patients posttreatment, and increased tumor infiltration by CD8-positive lymphocytes in five of eight patients posttreatment. CONCLUSIONS: Immunotherapy with intratumorally administered Leuvectin is safe and can lead to durable objective responses in patients with metastatic renal cell carcinoma.

14 [291].	Garcia, J. A., T. Mekhail, et al. (2011). "Clinical and immunomodulatory effects of bevacizumab and low-dose interleukin-2 in patients with metastatic renal cell carcinoma: results from a phase II trial." BJU Int **107**(4): 562-570.

OBJECTIVE: Low-dose interleukin-2 (IL-2) is a historical treatment for metastatic renal cell carcinoma (mRCC). Increased vascular endothelial growth factor (VEGF) levels inhibit dendritic cell (DC) differentiation and augment production of immunosuppressive regulatory T (Treg) cells. Bevacizumab is an antibody that binds to VEGF, has activity in mRCC and may augment the anti-tumour immune effects of IL-2. To determine the clinical and immunomodulatory effects of this combination, a prospective, phase II trial of bevacizumab plus low-dose IL-2 was conducted. PATIENTS AND METHODS: Patients with untreated mRCC received bevacizumab (10 mg/kg i.v. every 2 weeks) and IL-2 (125,000 units/kg/day subcutaneously from Monday to Friday for 6 consecutive weeks followed by a 2-week rest period). Endpoints included progression-free survival, Response Evaluation Criteria in Solid Tumors-defined objective response rate, immunomodulatory effects and safety. RESULTS: Between January 2005 and September 2007, twenty-six patients with untreated mRCC were enrolled. The median progression-free survival was 9.6 months (95% CI, 4.1-16.9 months) The objective response rate was 15% and an additional 38% of patients had tumour burden reduction of <30%. Grade 3 constitutional adverse events (fatigue, fever/chills) and neutropenia were observed in 42% and 12% of patients, respectively. Peripheral blood CD1c(+) myeloid and CD303(+) plasmacytoid DC increased during treatment as did IL-8 levels and CD4(+) CD25(+) FoxP3(+) Treg cells. No changes in T helper type 1/2-associated cytokines were observed. CONCLUSION: Bevacizumab plus low-dose IL-2 has modest clinical activity in mRCC. Toxicity was largely IL-2 related without enhancement of bevacizumab-related toxicity. Biological data indicate inhibition of VEGF levels and increase of immunosuppressive Treg cells without an effect on DC activation.

15 [379].	Geertsen, P. F., M. E. Gore, et al. (2004). "Safety and efficacy of subcutaneous and continuous intravenous infusion rIL-2 in patients with metastatic renal cell carcinoma." Br J Cancer **90**(6): 1156-1162.

A retrospective analysis was conducted on data from four open-label, nonrandomised, phase II trials of recombinant interleukin-2 (rIL-2) in patients with metastatic renal cell carcinoma to compare the safety and efficacy of administration by subcutaneous (s.c.) and continuous intravenous (c.i.v.) infusion (n=103 s.c. and n=225 c.i.v.). No statistically significant differences were found between the cohorts in terms of overall response rate (s.c.: 13.6% vs c.i.v.: 12.4%, P=0.77), response duration (s.c.: 9.8 months vs c.i.v.: 10.1 months, P=0.99), and overall survival (P=0.08). Compared with c.i.v. administration, more patients in the s.c. cohort experienced stable disease (50.5 vs 29.8%) and fewer underwent disease progression (35.0 vs 43.6%). Subcutaneous administration was associated with a significantly lower incidence of grade 3 or 4 adverse events (46 vs 76%; P<0.001), and fewer s.c. patients required dose reductions because of toxicity (20 vs 82%). At the doses and within the schedules tested, this comparative analysis did not detect any difference in efficacy between s.c. and c.i.v. administration of rIL-2 in terms of overall survival, duration of response and response rate in patients with metastatic renal cell carcinoma. However, s.c. delivery of rIL-2 was associated with improved tolerability.

16 [340].	Gollob, J. A., C. J. Sciambi, et al. (2006). "Phase I trial of sequential low-dose 5-aza-2'-deoxycytidine plus high-dose intravenous bolus interleukin-2 in patients with melanoma or renal cell carcinoma." Clin Cancer Res **12**(15): 4619-4627.

PURPOSE: The silencing of gene expression through DNA methylation contributes to defects in antigen presentation and apoptosis in melanoma and renal cell cancer. To determine how a hypomethylating agent would modulate the toxicity and antitumor activity of immunotherapy, we initiated a phase I trial of 5-aza-2'-deoxycytidine (decitabine) plus high-dose interleukin 2 (IL-2). EXPERIMENTAL DESIGN: Patients received s.c. decitabine daily x 5 days on weeks 1 and 2 of a 12-week cycle. High-dose IL-2, consisting of two cycles of IL-2 600,000 IU/kg i.v. q8 hours x 14 doses separated by a 2-week break, was administered starting on week 3. Decitabine was escalated from 0.1 to 0.25 mg/kg. The hypomethylating activity of decitabine was assessed during cycle 1 by measuring hemoglobin F levels and changes in DNA methylation in peripheral blood mononuclear cells. RESULTS: Twenty-one patients with melanoma or renal cell cancer were enrolled. Decitabine did not alter the tolerability of IL-2 but caused grade 4 neutropenia in most patients. Grade 4 neutropenia lasting more than 7 days was the only dose-limiting toxicity, with a trend toward a higher incidence with increasing decitabine doses. Infection occurred in only one patient despite the high incidence of neutropenia, and granulocyte colony-stimulating factor use in several patients expedited neutrophil recovery. Decitabine augmented hemoglobin F levels and altered DNA methylation and gene expression in peripheral blood mononuclear cells in a dose-independent manner that overlapped with the administration of IL-2. Objective responses occurred in 31% of melanoma patients. CONCLUSIONS: Decitabine can be safely administered with high-dose IL-2 and may enhance the activity of IL-2 in melanoma.

17 [326].	Hutson, T. E. and R. A. Figlin (2007). "Evolving role of novel targeted agents in renal cell carcinoma." Oncology (Williston Park) **21**(10): 1175-1180; discussion 1184, 1187, 1190.

The treatment of metastatic renal cell carcinoma (RCC) has changed dramatically over the past few years. An improved understanding of the biology of RCC has resulted in the development of novel targeted therapeutic agents that have altered the natural history of this disease. In particular, the hypoxia-inducible factor (HIF)/vascular endothelial growth factor (VEGF) pathway and the mammalian target of rapamycin (mTOR) signal transduction pathway have been exploited. Sunitinib malate (Sutent), sorafenib tosylate (Nexavar), bevacizumab (Avastin)/interferon alfa, and temsirolimus (Torisel) have improved clinical outcomes in randomized trials by inhibiting these tumorigenic pathways. Combinations and sequences of these agents are being evaluated. Other novel multitargeted tyrosine kinase inhibitors (pazopanib and axitinib) and mTOR inhibitors (everolimus) are in clinical development. Recently reported and ongoing clinical trials will help further define the role of these agents as therapy for metastatic RCC.

18 [317].	Kamikawa, S., T. Sugimoto, et al. (2008). "Pharmacokinetic study of interleukin-2 following intravenous injection in hemodialysis patients with renal cell carcinoma." Ther Apher Dial **12**(1): 67-71.

The purpose of the present study is to determine the change in blood concentration of interleukin-2 (IL-2) after intravenous injection in hemodialysis patients and to assess its safety. Four hemodialysis patients who underwent nephrectomy due to renal cell carcinoma were treated with IL-2 at a dose of 350 000-700 000 JRU by intravenous injection. Pharmacokinetic parameters were analyzed from the serum IL-2 concentration, which reached its peak just after the end of infusion, followed by biphasic elimination, and was below the detection limit in all patients at 24 h postinfusion. In comparison with patients with normal renal function, the volume of distribution in the serum compartment was almost comparable (3820 +/- 2020 mL). Clearance (50.47 +/- 11.50 mL/min) decreased to 40%, and the half-life of the distribution phase (0.45 +/- 0.19 h) and that of the terminal phase (1.72 +/- 0.20 h) were distinctly longer. The area under the blood concentration-time curve was about two-fold higher than that of non-hemodialysis patients. In all patients, there were no serious adverse reactions. The results of the present study suggest that intravenous IL-2 therapy can be safely performed in hemodialysis patients.

19 [308].	Kaufman, H. L., B. Taback, et al. (2009). "Phase II trial of Modified Vaccinia Ankara (MVA) virus expressing 5T4 and high dose Interleukin-2 (IL-2) in patients with metastatic renal cell carcinoma." J Transl Med **7**: 2.

BACKGROUND: Interleukin-2 (IL-2) induces durable objective responses in a small cohort of patients with metastatic renal cell carcinoma (RCC) but the antigen(s) responsible for tumor rejection are not known. 5T4 is a non-secreted membrane glycoprotein expressed on clear cell and papillary RCCs. A modified vaccinia virus Ankara (MVA) encoding 5T4 was tested in combination with high-dose IL-2 to determine the safety, objective response rate and effect on humoral and cell-mediated immunity. METHODS: 25 patients with metastatic RCC who qualified for IL-2 were eligible and received three immunizations every three weeks followed by IL-2 (600,000 IU/kg) after the second and third vaccinations. Blood was collected for analysis of humoral, effector and regulatory T cell responses. RESULTS: There were no serious vaccine-related adverse events. While no objective responses were observed, three patients (12%) were rendered disease-free after nephrectomy or resection of residual metastatic disease. Twelve patients (48%) had stable disease which was associated with improved median overall survival compared to patients with progressive disease (not reached vs. 28 months, p = 0.0261). All patients developed 5T4-specific antibody responses and 13 patients had an increase in 5T4-specific T cell responses. Although the baseline frequency of Tregs was elevated in all patients, those with stable disease showed a trend toward increased effector CD8+ T cells and a decrease in Tregs. CONCLUSION: Vaccination with MVA-5T4 did not improve objective response rates of IL-2 therapy but did result in stable disease associated with an increase in the ratio of 5T4-specific effector to regulatory T cells in selected patients. TRIAL REGISTRATION NUMBER: ISRCTN83977250.

20 [360].	Kerst, J. M., A. Bex, et al. (2005). "Prolonged low dose IL-2 and thalidomide in progressive metastatic renal cell carcinoma with concurrent radiotherapy to bone and/or soft tissue metastasis: a phase II study." Cancer Immunol Immunother **54**(9): 926-931.

Metastatic renal cell cancer is one of the immuno-sensitive tumors. Apart from the immuno-modulating agents IFNalpha and IL-2, thalidomide has been reported to be effective in this type of cancer. However, bone metastases and bulky metastases, show limited response to immunotherapy, are often site of recurrent disease and are therefore often treated later with radiotherapy. In this phase II study, we evaluated toxicity and efficacy of the combination of continuous low dose (1 mIU/m2) s.c. IL-2 and thalidomide (200 mg once daily) in 22 patients with progressive metastatic renal cell cancer. In addition, 13 soft tissue lesions and two bone metastases in 13 patients were concurrently treated with fractionated radiotherapy. T cell number and activation in blood was measured by immunoflowcytometry. Nearly all patients developed grade 1-2 toxicity consisting of fatigue, sensory neuropathy, constipation and dizziness. Five patients had a grade 3-4 toxic event: four patients with deep venous thrombosis requiring anticoagulant therapy, and one patient who developed radiation myelopathy. On systemic response evaluation ten patients showed ongoing SD with a mean progression free survival of 9 months. One patient showed a PR (at an irradiated site). Regarding local response to irradiation, seven lesions showed a PR for a mean time period of 8.7 months, whereas seven were stable for 6 months. The radiation response of one lesion was not evaluable. Immunoflowcytometry showed an increase in number and activation of lymphocytes (mainly Natural Killer--NK-cells), which was absent or even decreased in irradiated patients. The combination of sc. low dose IL-2, thalidomide and radiotherapy is feasible, but relatively toxic and does not lead to higher responses at non-irradiated sites. The combination of immunotherapy and concurrent radiotherapy is effective at 60% of the relatively large evaluable sites. Progressive myelopathy developed in one patient, possibly due to radiotherapy in combination with thalidomide.

21 [337].	Klatte, T., A. Ittenson, et al. (2006). "Perioperative immunomodulation with interleukin-2 in patients with renal cell carcinoma: results of a controlled phase II trial." Br J Cancer **95**(9): 1167-1173.

We conducted a non-randomised controlled phase II trial to investigate the role of preoperative administration of interleukin-2 (IL-2) in patients with renal cell carcinoma undergoing tumour nephrectomy. A total of 120 consecutive patients were allocated alternately to the two study groups: perioperative immunomodulation with IL-2 (IL-2 group; n=60) and perioperative immunomonitoring without immunomodulation (control group; n=60). Patients from the IL-2 group received four doses of 10 x 10(6) IU m(-2) twice daily subcutaneously a week before operation followed by a daily maintenance dose of 3 x 10(6) IU m(-2) subcutaneously until a day before the operation. Parameters of cellular and humoral immunity (leucocytes, T-cell markers CD3, CD4, and CD8, B-cell marker CD19, monocyte marker CD14, natural killer (NK) cell markers CD16, CD56, and CD57, activation markers CD6, CD25, CD28, and CD69, progenitor cell marker CD34, as well as IL-2, IL-6, IL-10, soluble IL-2 receptor, IL-1 receptor antagonist, transforming growth factor-beta1, and vascular endothelial growth factor) were measured in peripheral venous blood at various intervals. Interleukin-2-related toxicity was WHO grade 1 (24%), 2 (67%), and 3 (9%). In the postoperative period, T-cell markers, activation markers, and NK cell markers decreased, and IL-6 and IL-10 increased. However, all these alterations were significantly less accentuated in patients who had been pretreated with IL-2. Median follow-up was 40 months. Tumour-specific survival in the IL-2 group and the control group was 98 vs 81% after 1 year and 86 vs 73% after 5 years (P=0.04). A similar effect was found for progression-free survival. We conclude that IL-2 can be safely administered in the perioperative period and modulates immunological parameters. However, to validate the survival data, a larger randomised phase III trial is needed.

22 [355].	Koulova, L., Y. Novik, et al. (2005). "A phase 2 study of moderate dose interleukin-2 and granulocyte-macrophage colony-stimulating factor in patients with metastatic or unresectable renal cell carcinoma." J Immunother **28**(6): 576-581.

Interleukin-2 (IL-2) has been shown to produce durable complete remission in patients with renal cell carcinoma (RCC). A phase 2 study was conducted to evaluate the potential therapeutic synergy as well as the toxic side effects of the concurrent administration of IL-2 and granulocyte-macrophage colony-stimulating factor (GM-CSF) in patients with advanced stage disease. Twenty-one patients with unresectable or metastatic RCC having an Eastern Oncology Cooperative Group performance status of 0 or 1 were enrolled. Six patients had received prior immunotherapy with interferon (IFN)-alpha, IFN-gamma, and IL-12, whereas the remaining 15 subjects were previously untreated. Thirteen patients were assigned to a moderate-dose bolus of IL-2 at 72,000 IU/kg every 8 hours on days 1 through 5 and days 15 through 19, whereas 8 patients were given IL-2 as an intravenous continuous infusion at a dose of 5 MU/m2/d on days 1 through 5 and days 15 through 19. Subcutaneous GM-CSF at 125 microg/d on days 1 through 21 was administered concomitantly with IL-2. The median number of IL-2 bolus doses was 23 of a scheduled 28 (85%), whereas with the continuous infusion, 93% of planned IL-2 was given. All patients received 100% of GM-CSF doses. There were no complete or partial responses in this study. Of 13 patients treated in the bolus IL-2 arm, 10 had systemic progression of disease at 4 to 8 weeks, 1 developed metastasis in the brain at 4 weeks, and 2 had stable disease for 4 and 17 months. Among the 8 subjects treated with continuous infusion IL-2, 3 progressed with brain lesions at 3 to 8 weeks and 5 had stable disease at 6+, 7, 8+, 15+, and 17+ months. The median survival for the whole group was 10 months, with a range of 0.5 to 40+ months. There were no regimen-related deaths, and most of the observed toxicities were grade 1 and 2. Serious toxicities (grade 3 and 4) included anemia, atrial fibrillation, oliguria, abnormal liver function, and neurologic events like agitation or confusion. The combination of recombinant IL-2 and GM-CSF administered in the designed schedule and doses was not effective in patients with metastatic RCC and may even interfere with the therapeutic potential of moderate-dose IL-2 and increase its adverse events.

23 [279].	Kunzmann, V., M. Smetak, et al. (2012). "Tumor-promoting versus tumor-antagonizing roles of gammadelta T cells in cancer immunotherapy: results from a prospective phase I/II trial." J Immunother **35**(2): 205-213.

Emerging evidence suggests that nitrogen-containing bisphosphonates have direct and indirect anticancer effects including immunomodulatory effects. Using in vivo targeting of bisphosphonate-reactive gammadelta T cells by adding low-dose interleukin-2 to zoledronic acid, we evaluated the safety, pharmacodynamics, and antitumor activity of this immunotherapy approach in 21 adults with advanced malignancies (renal cell carcinoma [RCC], malignant melanoma, and acute myeloid leukemia). A total of 58 treatment cycles were administered and the median number of treatment cycles was 2.7 (range, 1 to 6). The regimen was well tolerated, with no grade 3 to 4 drug-related adverse events, except for fever. No objective responses were observed in both cohorts of solid tumors (RCC and malignant melanoma), whereas 2 patients with acute myeloid leukemia (25%) achieved objective tumor responses (partial remission). Pharmacodynamic analyses showed significant in vivo activation (interferon-gamma production) and expansion of gammadelta T cells in all evaluable patients. High pretreatment serum vascular endothelial growth factor (VEGF) levels and an unexpected increase in VEGF induced by zoledronic acid plus low-dose interleukin-2 were correlated with the lack of a clinical response. In conclusion, this study indicates that immunotherapy-induced VEGF can limit clinical innate tumor immune responses, especially for angiogenesis-dependent solid tumors. Our data challenge the current cellular immunotherapy paradigms in the treatment of cancer.

24 [341].	Majhail, N. S., L. Wood, et al. (2006). "Adjuvant subcutaneous interleukin-2 in patients with resected renal cell carcinoma: a pilot study." Clin Genitourin Cancer **5**(1): 50-56.

BACKGROUND: A pilot study was conducted to investigate the toxicity and tolerance to low-dose subcutaneous interleukin-2 (IL-2) for patients with resected renal cell carcinoma (RCC) at high risk for recurrent disease (TNM stages III and IV resected distant metastases). PATIENTS AND METHODS: Patients with surgically resected locally advanced (T3-4 or N1-2) or metastatic RCC were randomly assigned to 1 of 4 treatment groups that received different dose levels and schedules of subcutaneous IL-2 as follows: dose level 1, 4 MIU/m2 per day, every other week for 24 weeks (n = 10); dose level 2, 8 MIU/m2 per day, every other week for 24 weeks (n = 9); dose level 3, 4 MIU/m2 per day, weeks 1-4, 9-12, and 17-20 (n = 11); and dose level 4, 8 MIU/m2 per day, weeks 1-4, 9-12, and 17-20 (n = 10). Interleukin-2 was administered in 2 daily doses on days 1-5 of each week indicated. A dose level was considered tolerable if no more than 2 patients experienced grade 3/4 toxicity. RESULTS: Forty-one patients were entered in the study and 40 were evaluable for toxicity. Therapy was well tolerated at all dose levels and schedules, with most patients (98%) experiencing mild-to-moderate constitutional symptoms. Grade 3/4 toxicity was seen in 8 patients (20%). Interleukin-2 dose reductions were required in 7 patients, and no patient discontinued therapy secondary to toxicity. Of 39 patients evaluable for efficacy, 31 have experienced relapse (79%), and 15 have died (38%). Median survival was 1.4 years, and the 3-year disease-free survival rate was 33%. Median overall survival has not been reached; however, the 3-year survival rate was 70%. There was no statistically significant difference between any of the treatment arms with respect to disease-free survival or 3-year survival (P > 0.54 and P >or= 0.09 for all pairwise comparisons), schedules (dose level 1/2 vs. 3/4; P = 0.46 and P = 0.5), or dose of IL-2 administered (dose level 1/3 vs. 2/4; P = 0.99 and P = 0.1). CONCLUSION: Subcutaneous IL-2 was well tolerated for 6 months in patients with surgically resected RCC at high risk of recurrence. Future adjuvant trials in this setting are not likely to include IL-2 in view of the clinical efficacy and favorable toxicity profiles of selected multitargeted kinase inhibitors.

25 [330].	Margolin, K., M. B. Atkins, et al. (2007). "Phase I trial of BAY 50-4798, an interleukin-2-specific agonist in advanced melanoma and renal cancer." Clin Cancer Res **13**(11): 3312-3319.

PURPOSE: BAY 50-4798 is an analogue of interleukin-2 that selectively activates T cells over natural killer cells. This phase I study was designed to determine the maximum tolerated dose (MTD) and safety of BAY 50-4798, screen for tumor response, and assess pharmacokinetics. EXPERIMENTAL DESIGN: Forty-five patients with metastatic melanoma or renal cancer were enrolled, 31 on escalating doses to determine the MTD, with 20 renal cell carcinoma patients treated at MTD to detect antitumor activity. BAY 50-4798 was delivered i.v. every 8 h, days 1 to 5 and 15 to 19, and could be repeated after 9 weeks if tumor was stable or responding. RESULTS: The MTD was defined by and reported in terms of doses received. The doses tested ranged from 1.3 to 26.1 microg/kg, and the MTD was defined as 10.4 microg/kg based on toxicities similar to those of aldesleukin. Two patients achieved partial responses, one with melanoma and one with renal cell carcinoma. Among all 45 patients, 53% and 9% experienced a grade 3 and 4 toxicity, respectively. Among the patients treated at the MTD of 10.4 microg/kg, 71% and 10% experienced a grade 3 and 4 toxicity, respectively. Pharmacokinetics showed dose-dependent peak concentrations (C(max)) and area under the curve with a half-life of approximately 2 h and no evidence of accumulation. Lymphocyte subset analysis confirmed the preferential expansion of T-cell subsets over natural killer cells. CONCLUSIONS: The antitumor activity of BAY 50-4798 in malignancies that respond to high-dose interleukin-2 was low. BAY 50-4798 might provide advantages over aldesleukin in antigen-specific immunotherapies.

26 [357].	Melichar, B., D. Solichova, et al. (2005). "Neopterin in renal cell carcinoma: inhalational administration of interleukin-2 is not accompanied by a rise of urinary neopterin." Luminescence **20**(4-5): 311-314.

Inhalational administration of interleukin-2 (IL-2) is effective in controlling renal cell carcinoma (RCC) lung metastases with minimal toxicity. Neopterin is an indicator of systemic immune activation in metastatic cancer and is increased after systemic IL-2 administration. Urinary neopterin was investigated in 13 patients with metastatic RCC and 18 controls. In seven patients, urinary neopterin was followed before and after treatment with inhalational IL-2. Neopterin was measured by high-performance liquid chromatography and creatinine was determined by Jaffe reaction. Urinary neopterin was significantly increased in patients with metastatic RCC compared to controls (257 +/- 263 micromol/mol creatinine vs. 110 +/- 41 micromol/mol creatinine; Mann-Whitney U-test, p < 0.05). Median survival was significantly longer in patients with urinary neopterin <173 micromol/mol creatinine compared to patients with neopterin > or = 173 micromol/mol creatinine (698 vs. 245 days; log-rank test, p < 0.05). No significant increase was observed after inhalational IL-2 therapy (147 +/- 101 vs. 153 +/- 54 micromol/mol creatinine). We conclude that urinary neopterin is increased in patients with metastatic RCC, and higher neopterin concentrations are indicative of poor prognosis. The absence of an increase in urinary neopterin after inhalational IL-2 therapy is in accord with the lack of significant systemic toxicity.

27 [343].	Mencoboni, M. P., S. Tredici, et al. (2006). "Chemoimmunotherapy with low dose vinorelbine and interleukin-2 in treatment of patients with metastatic renal cell carcinoma." Neoplasma **53**(4): 333-336.

Systemic therapies employed in patients with metastatic renal cell carcinoma (MRCC) include chemotherapy to immunomodulatory cytokines (interleukin 2 [IL-2], interferon alpha [INFalpha]), chemoimmunotherapy, adoptive immune therapy and anti-angiogenic therapy. Despite this range of treatment alternatives, the optimal therapy for MRCC patients is far from being established. Thus, attempts with novel therapeutic approaches implementing new drug combinations are justified. We conducted a phase II evaluation of a combination of vinorelbine and IL-2, both at low doses, in 30 patients with MRCC. The rationale of the combination was to damage the tumor tissue to the extent necessary to make it more immunogenic while, at the same time, to obtain an efficient immune response through the concomitant administration of IL-2. The treatment, given in different dose combinations and administration times, resulted feasible, with no renal, neurological or hematological toxicity. The overall survival of the whole group of patients is higher than that usually observed following treatment with immunotherapies (18.2 versus 13.3 months, respectively). While the limited number of treated patients does not allow advancing conclusions on the effective activity of the adopted protocol, the results observed are encouraging.

28 [378].	Merimsky, O., E. Gez, et al. (2004). "Targeting pulmonary metastases of renal cell carcinoma by inhalation of interleukin-2." Ann Oncol **15**(4): 610-612.

INTRODUCTION: Pulmonary metastases of renal cell carcinoma (RCC) are associated with poor prognosis. Inhalation therapy with interleukin-2 (IL-2) is thus an appealing method for palliation. This multicenter study summarizes the national experience of IL-2 inhalation in patients with lung metastases of RCC. PATIENTS AND METHODS: Forty patients (median, 66.5 years of age) with radiologically documented progressing pulmonary metastases were enrolled. All patients had to be able to comply with inhalation technique, and were not candidates for other treatment options. Twenty-eight patients were systemic treatment-naive. The protocol included three daily inhalations of IL-2 to a total dose of 18 MU. Treatment had to be continued until one of the following occurred: progression; a complete response; a life threatening toxicity; or patient refusal. Response was assessed using the Response Evaluation Criteria in Solid Tumors (RECIST) system. RESULTS: The disease-control rate reached 57.5%, with a partial response rate of 2.5% and a disease stabilization rate of 55%. Median time to progression was 8.7 months. The main side-effects were cough and weakness. CONCLUSIONS: Inhalation of IL-2 for the treatment of pulmonary metastases in RCC is feasible, tolerable and beneficial in controlling progressive disease for considerable periods of time. The definition of response of biological therapy may need to be re-assessed and modified: stable disease should be regarded as a favorable response.

29 [338].	Mirza, N., M. Fishman, et al. (2006). "All-trans-retinoic acid improves differentiation of myeloid cells and immune response in cancer patients." Cancer Res **66**(18): 9299-9307.

Abnormal dendritic cell differentiation and accumulation of immature myeloid suppressor cells (ImC) is one of the major mechanisms of tumor escape. We tested the possibility of pharmacologic regulation of myeloid cell differentiation using all-trans-retinoic acid (ATRA). Eighteen patients with metastatic renal cell carcinoma were treated with ATRA followed by s.c. interleukin 2 (IL-2). Eight healthy individuals comprised a control group. As expected, the cancer patients had substantially elevated levels of ImC. We observed that ATRA dramatically reduced the number of ImC. This effect was observed only in patients with high plasma concentration of ATRA (>150 ng/mL), but not in patients with lower ATRA concentrations (<135 ng/mL). Effects of ATRA on the proportions of different dendritic cell populations were minor. However, ATRA significantly improved myeloid/lymphoid dendritic cell ratio and the ability of patients' mononuclear cells to stimulate allogeneic T cells. This effect was associated with significant improvement of tetanus-toxoid-specific T-cell response. During the IL-2 treatment, the ATRA effect was completely eliminated. To assess the role of IL-2, specimens from 15 patients with metastatic renal cell carcinoma who had been treated with i.v. IL-2 alone were analyzed. In this group also, IL-2 significantly reduced the number and function of dendritic cells as well as T-cell function. These data indicate that ATRA at effective concentrations eliminated ImC, improved myeloid/lymphoid dendritic cell ratio, dendritic cell function, and antigen-specific T-cell response. ATRA treatment did not result in significant toxicity and it could be tested in therapeutic combination with cancer vaccines.

30 [346].	Olencki, T., S. Malhi, et al. (2006). "Phase I trial of thalidomide and interleukin-2 in patients with metastatic renal cell carcinoma." Invest New Drugs **24**(4): 321-326.

BACKGROUND: The treatment of advanced renal cell cancer remains unsatisfactory, therefore new combination regimens such as thalidomide and IL-2 are of interest. A phase I trial of SC IL-2 and oral thalidomide was performed to identify the toxicity, maximum tolerated dose (MTD) and preliminary clinical activity of this regimen. METHODS: 33 patients with advanced/metastatic RCC were enrolled. An established 8-week outpatient schedule of subcutaneously administered IL-2 in escalating doses, days 1-5, for 6 weeks with a 2 week rest was utilized with daily oral thalidomide. Cohorts of 4-6 patients were treated at 4 dose levels. RESULTS: Toxicity was moderate to severe and related to dose level. All patients developed fever, chills and fatigue. 29/33 patients developed < or = Grade 2 desquamation of hands and feet and/or rash. Dose limiting toxicity (DLT) included Grade 3 neutropenia and pulmonary embolus. The maximum tolerated dose (MTD) of IL-2 and thalidomide was 9.0 MIU/m2 s.c. days 1-5, weeks 1 to 6 and 100 mg p.o. daily, respectively. A median of 2 cycles of therapy was administered (range 1-9). 2/33 patients responded (1 CR--prior IL-2 therapy, 1 PR--no prior therapy) with an overall response of 6% (95% CI, 1-20%). One minimal response was converted to a surgical CR (remains disease free at 24 + months). CONCLUSION: Outpatient administration of IL-2 and thalidomide is possible with acceptable toxicity. Further evaluation of this regimen is underway.

31 [348].	Peterson, A. C., H. Harlin, et al. (2006). "A randomized phase II trial of interleukin-2 in combination with four different doses of bryostatin-1 in patients with renal cell carcinoma." Invest New Drugs **24**(2): 141-149.

PURPOSE: Bryostatin-1 is a PKC modulator with direct anti-tumor activity and immunomodulatory properties. We combined different doses of Bryostatin-1 with IL-2 to determine effects on clinical response rate and T cell phenotype in patients with advanced kidney cancer. EXPERIMENTAL DESIGN: IL-2 naive patients were given 11 x 10(6) IU subcutaneously of IL-2 on days 1-4, 8-11, and 15-18 of every 28-day cycle. Twenty four patients were randomized to treatment cohorts of 5, 15 or 25 mcg/m2 of Bryostatin-1 on days 1, 8 and 15, starting in the second cycle. An additional nine, non-randomized patients were given 35 mcg/m2. Lymphocytes were analyzed for number, activation status, and production of IL-2, IL-4 and IFN-gamma. Response evaluation was performed every 3 cycles. RESULTS: Common grade 3 toxicities included fatigue (5), nausea/vomiting (5), myopathy (3), dyspnea (3), and syncope (3). Four patients, in the two highest dose cohorts, demonstrated evidence of tumor shrinkage, although there was only 1 objective PR. The median time to progression was 104 days (95% CI 88-120) and the median survival was 452 days (95% CI = 424-480). There was no significant boosting effect of Bryostatin-1 on lymphocytes. CONCLUSIONS: The addition of Bryostatin-1 to IL-2 was well tolerated, but the overall response rate was low (3.2%), indicating that further studies with this combination are not warranted.

32 [287].	Procopio, G., E. Verzoni, et al. (2011). "Sorafenib with interleukin-2 vs sorafenib alone in metastatic renal cell carcinoma: the ROSORC trial." Br J Cancer **104**(8): 1256-1261.

BACKGROUND: Preclinical investigations support combining sorafenib with IL-2 in the treatment of metastatic renal cell carcinoma (mRCC). METHODS: In this open-label, phase II study, 128 patients with mRCC were randomised to receive oral sorafenib, 400 mg twice daily, plus subcutaneous IL-2, 4.5 million international units (MIU) five times per week for 6 in every 8 weeks, or sorafenib alone. After enrolment of the first 40 patients, IL-2 dose was reduced to improve the tolerability. RESULTS: After a median follow-up of 27 months, median progression-free survival (PFS) was 33 weeks with sorafenib plus IL-2, and 30 weeks with sorafenib alone (P=0.109). For patients receiving the initial higher dose of IL-2, median PFS was 43 weeks vs 31 weeks for those receiving the lower dose. The most common adverse events were asthenia, hand-foot syndrome, hypertension, and diarrhoea. Grade 3-4 adverse events were reported for 38 and 25% of patients receiving combination and single-agent treatment, respectively. CONCLUSION: The combination of sorafenib and IL-2 did not demonstrate improved efficacy vs sorafenib alone. Improvements in PFS appeared greater in patients receiving higher-dose IL-2.

33 [273].	Procopio, G., E. Verzoni, et al. (2013). "Overall survival for sorafenib plus interleukin-2 compared with sorafenib alone in metastatic renal cell carcinoma (mRCC): final results of the ROSORC trial." Ann Oncol **24**(12): 2967-2971.

BACKGROUND: The ROSORC trial, a randomised, phase II trial comparing sorafenib plus interleukin (IL-2) versus sorafenib alone as first-line treatment of metastatic renal cell carcinoma (mRCC) failed to demonstrate differences in progression-free survival (PFS). Updated overall survival (OS) results are reported. PATIENTS AND METHODS: In this study, 128 patients were randomised to receive sorafenib 400 mg twice daily plus subcutaneous IL-2 4.5 million international units (MIU) five times per week for 6 weeks every 8 weeks (arm A) or sorafenib alone (arm B). OS was estimated with the Kaplan-Meier method and compared with the two-sided log-rank test. RESULTS: After a median follow-up of 58 months (interquartile range: 28-63 months), the median OS was 38 and 33 months in arms A and B, respectively (P = 0.667). The 5-year OS was 26.3% [95% confidence interval (CI) 15.9-43.5) and 23.1% (95% CI 13.2-40.5) for the combination- and single-agent arm, respectively. Most of the patients who were refractory to first-line treatment were subsequently treated with different targeted agents; they had a median survival greater than expected. CONCLUSIONS: This outcome suggests a synergistic effect of the subsequent therapies following sorafenib failure. CLINICALTRIALSGOV IDENTIFIER: NCT00609401.

34 [370].	Quan, W., Jr., W. Brick, et al. (2004). "Repeated cycles with 72-hour continuous infusion interleukin-2 in kidney cancer and melanoma." Cancer Biother Radiopharm **19**(3): 350-354.

While high-dose bolus inpatient interleukin-2 is generally given on 8-week cycles, continuous infusion interleukin-2 could potentially allow for more rapidly repeated cycles. Fourteen (14) patients with an Eastern Cooperative Oncology Group (ECOG) performance status (PS) of 0 or 1, having either kidney cancer (6) or melanoma (8), have been treated with continuous infusion (CIV) interleukin-2 (IL-2) 18 MIU/m(2)/24 hours for 72 hours. Cycles were repeated every 3 weeks up to 4 cycles, then every 3-4 weeks for 2 cycles, then every 6-8 weeks, until progression or intolerable toxicity. All patients received famotidine 20 mg intravenously (i.v.) twice per day during the 72-hour infusions. Patient characteristics included a median ECOG performance status of 1; median age = 63 (range: 25-79); most common metastatic sites: lung (9), bone (5), lymph nodes (5), and the liver (3). No patients with metastatic kidney cancer underwent a nephrectomy prior to interleukin-2. Median number of cycles received = 5 (1-9). No patients required Intensive Care Unit (ICU) admission. There have been no treatment-related deaths. Most common toxicities have been rigors, fever, nausea/emesis, and the reversible elevation of creatinine. One complete response and three partial responses (67% response rate; 95% confidence interval: 30%-90%) have been seen in kidney cancer, and two partial responses (25% response rate; 95% confidence interval: 7%-60%) have occurred in melanoma. Median survival has not been reached at >9+ months. Responding sites include the liver, bone, lung, lymph node and subcutaneous sites. Inpatient 72-hour continuous infusion interleukin-2 at this dose and schedule is well tolerated by patients with an ECOG performance status of 0 or 1 and has activity in kidney cancer and melanoma.

35 [306].	Quan, W. D., Jr. and F. M. Quan (2009). "High-dose intensity pulse interleukin-2 with famotidine in metastatic kidney cancer." Cancer Biother Radiopharm **24**(2): 181-183.

Lymphokine-activated killer cell (LAK) activity against tumor cell lines may be induced by intravenous (i.v.) interleukin-2 (IL-2). Daily short infusions (pulses) have been developed to decrease toxicity while maintaining the anticancer activity of this agent against kidney cancer. The anthihistamine, famotidine, may increase IL-2 uptake by the IL-2 receptor on lymphocytes. We have treated 12 patients with metastatic kidney cancer, using pulse IL-2 (18 million IU/M(2) i.v.) over 15-30 minutes, preceded by famotidine (20 mg I.V. daily for 5 days) on an oncology inpatient unit. Cycles were repeated every 3 weeks until disease progression. Patient characteristics were as follows: 9 males with a median age of 66 years (range, 48-74), and median Eastern Cooperative Oncology Group performance status of 1; common metastatic sites included in the lungs 9 and lymph nodes 3. Median number of cycles received was 2 (range, 1-5). The most common toxicities were fever, rigors, and hypomagnesemia. Two (2) patients had partial responses (17% response rate). Responses occurred in the liver (11.5 months) and lung, pleura, and lymph nodes (3 months). Pulse IL-2 with famotidine shows activity in kidney cancer.

36 [327].	Renard, V., L. Staelens, et al. (2007). "Iodine-123-interleukin-2 scintigraphy in metastatic hypernephroma: a pilot study." Q J Nucl Med Mol Imaging **51**(4): 352-356.

AIM: This pilot study reports on the uptake of (123)I-interleukin 2 (IL-2) in metastatic renal cell carcinoma (MRCC) patients and its relationship to prognostic factors of response or failure of MRCC to cytokines treatment. METHODS: Nine consecutive patients with MRCC underwent an (123)I-IL-2 scan (6 male and 3 female; mean age 64 years; range 51-78). Uptake in metastases was related to a summed score of 4 independent factors, predictive of rapid progression under cytokine treatment as defined by Negrier et al. RESULTS: Four patients presented with metastases at one site, 4 at 2 sites and one patient at 3 different sites. Summed scores were: 5 patients had a summed score of 1; 3 a summed score of 2 and 1 patient a summed score of 3. Uptake of (123)I-IL-2 by tumor tissue was found in only 2 patients. Uptake occurred in 1 patient with a summed score of 3 and in 1 with a summed score of 2. CONCLUSION: In this small series of patients with MRCC, (123)I-IL-2 uptake was found in tumors of 2 patients who less likely will benefit from cytokine treatment. Additional studies are needed to assess the relationship between the pretreatment uptake of (123)/I-IL2 in MRCC and the response to IL-2 therapy.

37 [307].	Sabatino, M., S. Kim-Schulze, et al. (2009). "Serum vascular endothelial growth factor and fibronectin predict clinical response to high-dose interleukin-2 therapy." J Clin Oncol **27**(16): 2645-2652.

PURPOSE: High-dose interleukin-2 (IL-2) induces durable therapeutic responses in a small subset of patients with metastatic melanoma and renal cell carcinoma, but simple pretreatment predictors of response have not been identified. PATIENTS AND METHODS: To identify predictive biomarkers of clinical response, sera from patients treated with high-dose IL-2 were collected for analysis using a customized, multiplex antibody-targeted protein array platform that surveyed expression of soluble factors associated with tumor immunobiology. Soluble factors associated with clinical responses were analyzed using a multivariate permutation test, and survival outcomes were determined using Kaplan-Meier and log-rank tests. RESULTS: A training set from 10 patients identified 68 potentially relevant soluble factors that were then tested in an independent validation set of 49 patients. Class comparison revealed a cluster of 11 biomarkers that were associated with therapeutic outcome. Vascular endothelial growth factor (VEGF) and fibronectin were identified as independent predictors of response. In particular, high levels of these proteins were correlated with lack of clinical response and decreased overall survival. CONCLUSION: Serum VEGF and fibronectin are easily measured pretreatment biomarkers that could serve to exclude patients unlikely to respond to IL-2 therapy.

38 [277].	Seung, S. K., B. D. Curti, et al. (2012). "Phase 1 study of stereotactic body radiotherapy and interleukin-2--tumor and immunological responses." Sci Transl Med **4**(137): 137ra174.

Preclinical models suggest that focal high-dose radiation can make tumors more immunogenic. We performed a pilot study of stereotactic body radiation therapy (SBRT) followed by high-dose interleukin-2 (IL-2) to assess safety and tumor response rate and perform exploratory immune monitoring studies. Patients with metastatic melanoma or renal cell carcinoma (RCC) who had received no previous medical therapy for metastatic disease were eligible. Patients received one, two, or three doses of SBRT (20 Gy per fraction) with the last dose administered 3 days before starting IL-2. IL-2 (600,000 IU per kilogram by means of intravenous bolus infusion) was given every 8 hours for a maximum of 14 doses with a second cycle after a 2-week rest. Patients with regressing disease received up to six IL-2 cycles. Twelve patients were included in the intent-to-treat analysis, and 11 completed treatment per the study design. Response Evaluation Criteria in Solid Tumors criteria were used to assess overall response in nonirradiated target lesions. Eight of 12 patients (66.6%) achieved a complete (CR) or partial response (PR) (1 CR and 7 PR). Six of the patients with PR on computed tomography had a CR by positron emission tomography imaging. Five of seven (71.4%) patients with melanoma had a PR or CR, and three of five (60%) with RCC had a PR. Immune monitoring showed a statistically significantly greater frequency of proliferating CD4(+) T cells with an early activated effector memory phenotype (CD3(+)CD4(+)Ki67(+)CD25(+)FoxP3(-)CCR7(-)CD45RA(-)CD27(+)CD28(+/-)) in the peripheral blood of responding patients. SBRT and IL-2 can be administered safely. Because the response rate in patients with melanoma was significantly higher than expected on the basis of historical data, we believe that the combination and investigation of CD4(+) effector memory T cells as a predictor of response warrant further study.

39 [333].	Shaw, D. M., N. B. Connolly, et al. (2007). "A phase II study of a 5T4 oncofoetal antigen tumour-targeted superantigen (ABR-214936) therapy in patients with advanced renal cell carcinoma." Br J Cancer **96**(4): 567-574.

In a phase II study, 43 renal cell carcinoma patients were treated with individualised doses of ABR-214936; a fusion of a Fab recognising the antigen 5T4, and Staphylococcal enterotoxin A. Drug was given intravenously on 4 consecutive days, treatment was repeated 1 month later. Treatment was associated with moderate fever and nausea, but well tolerated. Of 40 evaluable patients, 28 had disease control at 2 months, and at 4 months, one patient showed partial response (PR) and 16 patients stable disease. Median survival, with minimum follow-up of 26 months was 19.7 months with 13 patients alive to date. Stratification by the Motzer's prognostic criteria highlights prolonged survival compared to published expectation. Patients receiving higher drug exposure had greater disease control and lived almost twice as long as expected, whereas the low-exposure patients survived as expected. Sustained interleukin-2 (IL-2) production after a repeated injection appears to be a biomarker for clinical effect, as the induced-IL-2 level on the day 2 of treatment correlated with survival. The high degree of disease control and the prolonged survival suggest that this treatment can be effective. These findings will be used in the trial design for the next generation of drug, with reduced antigenicity and toxicity.

40 [312].	Sheng, X. N., J. L. Li, et al. (2008). "[Clinical study on recombinant human interleukin-2 (Proleukin) in the treatment of metastatic renal cell carcinoma]." Zhonghua Zhong Liu Za Zhi **30**(2): 129-133.

OBJECTIVE: To evaluate the efficacy and safety of subcutaneous injection of recombinant human interleukin-2 (Proleukin) in the treatment of metastatic renal cell carcinoma (RCC). METHODS: Forty-one patients with pathologically confirmed metastatic RCC after radical nephrectomy were enrolled into this study. Two or four consecutive cycles of subcutaneous injection of rhLL-2 were given, with each cycle duration of five weeks consisting of 4 weeks of treatment and one week of rest. The rhLL-2 was injected twice daily subcutaneously at a dose of 9 MIU on D1-D5 during week one, then 9 MIU twice daily on D1-D2 and followed by 9 MIU daily on D3-D5 during week 2-4. Patients were evaluated after the second cycle of treatment. If an objective response or stable disease was observed, the patient would receive another two cycles of treeatment. RESULTS: Of the 41 patients, the overall objective response rate was 17.1% (95% confidence interval, 5.6% to 28.6%) with a complete response (CR) rate of 0.0% and partial response rate (PR) of 17.1%. However, nineteen patients (46.3%) still had a stable disease (SD), and 15 (36.6%) had progressed disease (PD). The disease control rate was 63.4% and the median time to progression (mTTP) was 6 months. The 1-year survival rate was 71.2% with a median overall survival (mOS) rate of 22.5 months. Among 36 PP population, the overall objective response rate was 19.4% (95% confidence interval, 6.5% to 32.3%) with CR rate of 0.0% and PR rate of 19.4%. Sixteen patients(44.4%) had stable disease, and 13 (36.1%) progressed disease. The disease control rate was 63.9%. The 1-year survival rate was 66.7% with a median time to progression of 6 months. The median overall survival (mOS) had not reached yet. The follow-up data showed that the long term survival of the patient who responsed to the IL-2 therapy can be prolonged. Severe toxicity (> or = grade III) was rarely observed. Grade I or II toxicities such as fatigue (100.0%) and fever (82.9%) were frequently observed but reversible. CONCLUSION: Subcutaneous injection of recombinant human interleukin-2 may prolong the survival of patients with a metastatic renal cell carcinoma. This regimen is tolerable with rare severe toxicities.

41 [331].	van der Vliet, H. J., H. B. Koon, et al. (2007). "Effects of the administration of high-dose interleukin-2 on immunoregulatory cell subsets in patients with advanced melanoma and renal cell cancer." Clin Cancer Res **13**(7): 2100-2108.

PURPOSE: High-dose recombinant human interleukin-2 (IL-2) therapy is of clinical benefit in a subset of patients with advanced melanoma and renal cell cancer. Although IL-2 is well known as a T-cell growth factor, its potential in vivo effects on human immunoregulatory cell subsets are largely unexplored. EXPERIMENTAL DESIGN: Here, we studied the effects of high-dose IL-2 therapy on circulating dendritic cell subsets (DC), CD1d-reactive invariant natural killer T cells (iNKT), and CD4(+)CD25(+) regulatory-type T cells. RESULTS: The frequency of both circulating myeloid DC1 and plasmacytoid DC decreased during high-dose IL-2 treatment. Of these, only a significant fraction of myeloid DC expressed CD1d. Although the proportion of Th1-type CD4(-) iNKT increased, similarly to DC subsets, the total frequency of iNKT decreased during high-dose IL-2 treatment. In contrast, the frequency of CD4(+)CD25(+) T cells, including CD4(+)Foxp3(+) T cells, which have been reported to suppress antitumor immune responses, increased during high-dose IL-2 therapy. However, there was little, if any, change of expression of GITR, CD30, or CTLA-4 on CD4(+)CD25(+) T cells in response to IL-2. Functionally, patient CD25(+) T cells at their peak level (immediately after the first cycle of high-dose IL-2) were less suppressive than healthy donor CD25(+) T cells and mostly failed to Th2 polarize iNKT. CONCLUSIONS: Our data show that there are reciprocal quantitative and qualitative alterations of immunoregulatory cell subsets with opposing functions during treatment with high-dose IL-2, some of which may compromise the establishment of effective antitumor immune responses.

42 [353].	Walker, P. R., S. A. Khuder, et al. (2005). "Continuous infusion interleukin-2 and antihistamines in metastatic kidney cancer." Cancer Biother Radiopharm **20**(5): 487-490.

A prior randomized trial suggested a possible survival advantage favoring the combination of histamine and subcutaneous interleukin-2 (IL-2), compared to IL-2 alone in patients with metastatic melanoma. It has been postulated previously that antihistamines may, therefore, actually be antagonistic to IL-2 and thus interfere with its antitumor activity. We have previously shown no such antagonistic effect in patients with melanoma receiving IL-2 and antihistamines when reviewing the known literature. We sought to determine whether there was any negative effect of the combination in patients with metastatic kidney cancer. A PubMed literature search between 1985 and 2005 was done. High-dose continuous (or constant) infusion (CIV) interleukin-2 was used as the reference therapy because of the relatively constant IL-2 levels generated by this approach. Studies in which cimetidine, ranitidine, or famotidine were regularly scheduled and administered concurrently with IL-2 were included. Thirteen studies were identified. A total of 47 patients responded to therapy. Total response rate = 22%; 95%; Confidence Interval: 17%-28%. Eleven complete responses were noted. Complete response rate = 5%; 95% Confidence Interval: 3%-9%. These response rates are consistent with previously noted IL-2 response rates. In this study of CIV IL-2 and antihistamines, this combination appears to be active in metastatic kidney cancer. There appears to be no negative effect of antihistamine on the CIV IL-2 response rate in this disease.

# TOPIC: [Kidney Cancer or Renal Cancer] and Anti-PD-1

1 [384].	Brahmer, J. R., C. G. Drake, et al. (2010). "Phase I study of single-agent anti-programmed death-1 (MDX-1106) in refractory solid tumors: safety, clinical activity, pharmacodynamics, and immunologic correlates." J Clin Oncol **28**(19): 3167-3175.

PURPOSE: Programmed death-1 (PD-1), an inhibitory receptor expressed on activated T cells, may suppress antitumor immunity. This phase I study sought to determine the safety and tolerability of anti-PD-1 blockade in patients with treatment-refractory solid tumors and to preliminarily assess antitumor activity, pharmacodynamics, and immunologic correlates. PATIENTS AND METHODS: Thirty-nine patients with advanced metastatic melanoma, colorectal cancer (CRC), castrate-resistant prostate cancer, non-small-cell lung cancer (NSCLC), or renal cell carcinoma (RCC) received a single intravenous infusion of anti-PD-1 (MDX-1106) in dose-escalating six-patient cohorts at 0.3, 1, 3, or 10 mg/kg, followed by a 15-patient expansion cohort at 10 mg/kg. Patients with evidence of clinical benefit at 3 months were eligible for repeated therapy. RESULTS: Anti-PD-1 was well tolerated: one serious adverse event, inflammatory colitis, was observed in a patient with melanoma who received five doses at 1 mg/kg. One durable complete response (CRC) and two partial responses (PRs; melanoma, RCC) were seen. Two additional patients (melanoma, NSCLC) had significant lesional tumor regressions not meeting PR criteria. The serum half-life of anti-PD-1 was 12 to 20 days. However, pharmacodynamics indicated a sustained mean occupancy of > 70% of PD-1 molecules on circulating T cells > or = 2 months following infusion, regardless of dose. In nine patients examined, tumor cell surface B7-H1 expression appeared to correlate with the likelihood of response to treatment. CONCLUSION: Blocking the PD-1 immune checkpoint with intermittent antibody dosing is well tolerated and associated with evidence of antitumor activity. Exploration of alternative dosing regimens and combinatorial therapies with vaccines, targeted therapies, and/or other checkpoint inhibitors is warranted.

2 [382].	Lipson, E. J., W. H. Sharfman, et al. (2013). "Durable cancer regression off-treatment and effective reinduction therapy with an anti-PD-1 antibody." Clin Cancer Res **19**(2): 462-468.

PURPOSE: Results from the first-in-human phase I trial of the anti-programmed death-1 (PD-1) antibody BMS-936558 in patients with treatment-refractory solid tumors, including safety, tolerability, pharmacodynamics, and immunologic correlates, have been previously reported. Here, we provide long-term follow-up on three patients from that trial who sustained objective tumor regressions off therapy, and test the hypothesis that reinduction therapy for late tumor recurrence can be effective. EXPERIMENTAL DESIGN: Three patients with colorectal cancer, renal cell cancer, and melanoma achieved objective responses on an intermittent dosing regimen of BMS-936558. Following cessation of therapy, patients were followed for more than 3 years. A patient with melanoma who experienced a prolonged partial regression followed by tumor recurrence received reinduction therapy. RESULTS: A patient with colorectal cancer experienced a complete response, which is ongoing after 3 years. A patient with renal cell cancer experienced a partial response lasting 3 years off therapy, which converted to a complete response, which is ongoing at 12 months. A patient with melanoma achieved a partial response that was stable for 16 months off therapy; recurrent disease was successfully treated with reinduction anti-PD-1 therapy. CONCLUSION: These data represent the most prolonged observation to date of patients with solid tumors responding to anti-PD-1 immunotherapy and the first report of successful reinduction therapy following delayed tumor progression. They underscore the potential for immune checkpoint blockade with anti-PD-1 to reset the equilibrium between tumor and the host immune system.

3 [383].	Topalian, S. L., F. S. Hodi, et al. (2012). "Safety, activity, and immune correlates of anti-PD-1 antibody in cancer." N Engl J Med **366**(26): 2443-2454.

BACKGROUND: Blockade of programmed death 1 (PD-1), an inhibitory receptor expressed by T cells, can overcome immune resistance. We assessed the antitumor activity and safety of BMS-936558, an antibody that specifically blocks PD-1. METHODS: We enrolled patients with advanced melanoma, non-small-cell lung cancer, castration-resistant prostate cancer, or renal-cell or colorectal cancer to receive anti-PD-1 antibody at a dose of 0.1 to 10.0 mg per kilogram of body weight every 2 weeks. Response was assessed after each 8-week treatment cycle. Patients received up to 12 cycles until disease progression or a complete response occurred. RESULTS: A total of 296 patients received treatment through February 24, 2012. Grade 3 or 4 drug-related adverse events occurred in 14% of patients; there were three deaths from pulmonary toxicity. No maximum tolerated dose was defined. Adverse events consistent with immune-related causes were observed. Among 236 patients in whom response could be evaluated, objective responses (complete or partial responses) were observed in those with non-small-cell lung cancer, melanoma, or renal-cell cancer. Cumulative response rates (all doses) were 18% among patients with non-small-cell lung cancer (14 of 76 patients), 28% among patients with melanoma (26 of 94 patients), and 27% among patients with renal-cell cancer (9 of 33 patients). Responses were durable; 20 of 31 responses lasted 1 year or more in patients with 1 year or more of follow-up. To assess the role of intratumoral PD-1 ligand (PD-L1) expression in the modulation of the PD-1-PD-L1 pathway, immunohistochemical analysis was performed on pretreatment tumor specimens obtained from 42 patients. Of 17 patients with PD-L1-negative tumors, none had an objective response; 9 of 25 patients (36%) with PD-L1-positive tumors had an objective response (P=0.006). CONCLUSIONS: Anti-PD-1 antibody produced objective responses in approximately one in four to one in five patients with non-small-cell lung cancer, melanoma, or renal-cell cancer; the adverse-event profile does not appear to preclude its use. Preliminary data suggest a relationship between PD-L1 expression on tumor cells and objective response. (Funded by Bristol-Myers Squibb and others; ClinicalTrials.gov number, NCT00730639.).

# TOPIC: [Kidney Cancer or Renal Cancer] and Anti-PD-L1

1 [385].	Brahmer, J. R., S. S. Tykodi, et al. (2012). "Safety and activity of anti-PD-L1 antibody in patients with advanced cancer." N Engl J Med **366**(26): 2455-2465.

BACKGROUND: Programmed death 1 (PD-1) protein, a T-cell coinhibitory receptor, and one of its ligands, PD-L1, play a pivotal role in the ability of tumor cells to evade the host's immune system. Blockade of interactions between PD-1 and PD-L1 enhances immune function in vitro and mediates antitumor activity in preclinical models. METHODS: In this multicenter phase 1 trial, we administered intravenous anti-PD-L1 antibody (at escalating doses ranging from 0.3 to 10 mg per kilogram of body weight) to patients with selected advanced cancers. Anti-PD-L1 antibody was administered every 14 days in 6-week cycles for up to 16 cycles or until the patient had a complete response or confirmed disease progression. RESULTS: As of February 24, 2012, a total of 207 patients--75 with non-small-cell lung cancer, 55 with melanoma, 18 with colorectal cancer, 17 with renal-cell cancer, 17 with ovarian cancer, 14 with pancreatic cancer, 7 with gastric cancer, and 4 with breast cancer--had received anti-PD-L1 antibody. The median duration of therapy was 12 weeks (range, 2 to 111). Grade 3 or 4 toxic effects that investigators considered to be related to treatment occurred in 9% of patients. Among patients with a response that could be evaluated, an objective response (a complete or partial response) was observed in 9 of 52 patients with melanoma, 2 of 17 with renal-cell cancer, 5 of 49 with non-small-cell lung cancer, and 1 of 17 with ovarian cancer. Responses lasted for 1 year or more in 8 of 16 patients with at least 1 year of follow-up. CONCLUSIONS: Antibody-mediated blockade of PD-L1 induced durable tumor regression (objective response rate of 6 to 17%) and prolonged stabilization of disease (rates of 12 to 41% at 24 weeks) in patients with advanced cancers, including non-small-cell lung cancer, melanoma, and renal-cell cancer. (Funded by Bristol-Myers Squibb and others; ClinicalTrials.gov number, NCT00729664.).
