# Supplementary material for: Society for Immunotherapy of Cancer consensus statement on immunotherapy for the treatment of renal cell carcinoma
Source: J Immunother Cancer. 2016 Nov 15;4:81. doi: 10.1186/s40425-016-0180-7 (PMC5109802; doi:10.1186/s40425-016-0180-7)
Supplement: Additional file 4: — Task Force Pre-meeting Survey Questions. (DOCX 34 kb) [file 40425_2016_180_MOESM4_ESM.docx]

| 1. **What best describes your primary role in RCC?** | |
| --- | --- |
| **Answer Options** | **Response Percent** |
| Medical Oncologist | 62.5% |
| Urologist | 12.5% |
| Nurse | 6.3% |
| Patient or Patient Advocate | 12.5% |
| Other (Nurse Practitioner) | 6.3% |
| 1. **Which of the following is the primary focus of your clinical activity?** | |
| **Answer Options** | **Response Percent** |
| Local regional management of RCC | 0.0% |
| Management of patients with advanced RCC | 47.1% |
| All of the above | 35.3% |
| Neither/Not applicable | 11.8% |
| Other (Administrative) | 5.9% |
| 1. **With which of the following do you have clinical experience (select all that apply)?** | |
| **Answer Options** | **Response Percent** |
| VEGF targeted therapy | 88.2% |
| HD IL-2 | 82.4% |
| Nephrectomy | 58.8% |
| Ablative therapy | 41.2% |
| M Tor Therapy | 76.5% |
| Clinical Trials | 88.2% |
| None/Not applicable | 5.9% |
| Other (Patient education and coordination of care) | 11.8% |
| 1. **Which of the following FDA-approved agents have you used for patients with RCC (select all that apply)?** | |
| **Answer Options** | **Response Percent** |
| Sunitinib | 88.2% |
| Pazopanib | 82.4% |
| Axitinib | 88.2% |
| Bevacizumab | 82.4% |
| IFN | 82.4% |
| HD IL-2 | 70.6% |
| Everolimus | 82.4% |
| Temsiroliums | 82.4% |
| None/Not applicable | 11.8% |
| 1. **Which of the following non-FDA-approved agents have you used or recommend for patients with RCC (select all that apply)?** | |
| **Answer Options** | **Response Percent** |
| Anti-PD1 | 82.4% |
| Anti-PDL1 | 58.8% |
| Allogeneic BMT | 29.4% |
| Cabozantinib | 64.7% |
| Dendritic cell vaccine | 47.1% |
| Other RCC vaccine | 52.9% |
| None/Not applicable | 11.8% |
| 1. **What is your current opinion of high-dose Interleukin-2 (IL-2)?** | |
| **Answer Options** | **Response Percent** |
| I do not believe it has a role in the treatment of mRCC | 0.0% |
| I think there is a limited role and will refer patients to centers of excellence when appropriate | 33.3% |
| I think all patients should have a discussion about IL-2 before receiving non-curative TKI therapy | 60.0% |
| I use HD IL-2 as my principal initial therapy for patients with mRCC | 6.7% |
| 1. **What is your current opinion of interferon alpha?** | |
| **Answer Options** | **Response Percent** |
| I do not believe it has a role in the treatment of mRCC; even when I give bevacizumab I do not add interferon | 60.0% |
| I think there is a limited role and will use it only in conjunction with bevacizumab | 20.0% |
| I believe single agent IFN still has a role in the treatment of mRCC | 13.3% |
| I think IFN has no current role, but may be useful in combination with checkpoint blockade in the future. | 6.7% |
| 1. **Rank the patient/tumor characteristics required for use of, or referral for high-dose IL-2? (1 indicating the highest importance).** | |
| **Answer Options** | **Rating Average** |
| Clear cell histology | 1.21 |
| Age (provide maximum age in Q. 9 if relevant) | 4.64 |
| Adequate heart and lung function | 3.57 |
| Lack on CNS metastases | 5.42 |
| Lack of bone metastases | 7.40 |
| Lack of liver metastases | 8.56 |
| Prior nephrectomy | 4.93 |
| No prior TKI use | 7.27 |
| Performance status | 3.71 |
| MSKCC risk group | 7.36 |
| CAIX status | 10.78 |
| Lack of sarcomatoid histology | 9.00 |
| Other | 11.00 |
| 1. **Did you select Age as a relevant ranking factor in Question 8?** | |
| **Answer Options** | **Response Percent** |
| No | 26.7% |
| Yes [Provide maximum age: 60 (18%), 69 (9%), 70 (55%), 75 (18%)] | 73.3% |
| 1. **What is your first line treatment regimen for a 50 y.o. patient, s/p nephrectomy with clear cell RCC, with PS 0, normal organ function and 3 small lung metastases?** | |
| **Answer Options** | **Response Percent** |
| Sunitinib | 6.7% |
| Pazopanib | 0.0% |
| HD-IL-2 | 73.3% |
| Bevacizumab + IFN | 0.0% |
| Watchful waiting | 13.3% |
| Surgical resection to NED | 6.7% |
| 1. **What is the maximum age for which you will give/recommend HD IL-2?** | |
| **Answer Options** | **Response Percent** |
| 75 | 6.7% |
| Physiologically 70 | 66.7% |
| 70 | 0.0% |
| 65 | 26.7% |
| Other (please specify) | 0.0% |
| 1. **Patients with stable disease (<10% tumor shrinkage) 12 weeks following HD IL-2 should:** | |
| **Answer Options** | **Response Percent** |
| Receive a 2nd course of 2 cycles | 80.0% |
| Be observed until further progression, then start another systemic therapy | 13.3% |
| Go immediately on to VEGF targeted therapy | 0.0% |
| Other (observe for a late response if progression, then move on to other systemic therapy) | 6.7% |
| 1. **Patients with response to HD IL-2 therapy lasting at least 6 months whose disease then shows progression should:** | |
| **Answer Options** | **Response Percent** |
| Receive another cycle of HD IL-2 | 13.3% |
| Go on to another therapy | 73.3% |
| Undergo surgical resection of residual disease. | 13.3% |
| 1. **Patients with major response to 2 cycles of HD IL-2 who have residual oligometastatic disease should be managed with:** | |
| **Answer Options** | **Response Percent** |
| Switch to VEGF TKI | 6.7% |
| Ablative therapy of residual disease | 0.0% |
| Another cycle of HD IL-2 | 20.0% |
| Surgical resection of metastatic disease | 73.3% |
| 1. **A 50 yo patient with clear RCC with lung metastases has shown disease progression after sunitinib (12 months) and pazopanib (8 months). His performance status is 0 and he has no residual symptoms. What treatment would you recommend?** | |
| **Answer Options** | **Response Percent** |
| Axitinib | 6.7% |
| Everolimus | 6.7% |
| HD IL-2 | 13.3% |
| Clinical trial with novel targeted agent (cabozantinib) | 6.7% |
| Clinical Trial with anti-PD1 | 66.7% |
| 1. **Under what conditions would you recommend HD-IL-2 to the above patient? (select all that apply):** | |
| **Answer Options** | **Response Percent** |
| 6 months drug holiday following last VEGF targeted therapy | 6.7% |
| Normal cardiac stress test +/- cardiac echo | 46.7% |
| Thorough discussion of risks and potential benefits | 66.7% |
| Never recommend | 33.3% |
| 1. **What is your treatment recommendation for a young patient with ccRCC and lung mets with 2 CNS mets treated with SRS? (select one):** | |
| **Answer Options** | **Response Percent** |
| VEGFR TKI | 46.7% |
| HD IL-2 | 40.0% |
| mTor inhibitor | 0.0% |
| Other (clinical trial) | 13.3% |
| 1. **What tumor features would exclude your recommendation for HD IL-2 therapy (select all that apply):** | |
| **Answer Options** | **Response Percent** |
| Sarcomatoid histology | 40.0% |
| Non-clear cell features | 100.0% |
| Fuhrman grade 4 | 13.3% |
| Extensive granular features | 13.3% |
| No PDL1 expression | 6.7% |
| Other (please specify) | 0.0% |
| 1. **What is your initial treatment recommendation for patients with metastatic papillary RCC involving RP lymph nodes?** | |
| **Answer Options** | **Response Percent** |
| VEGFR TKI | 0.0% |
| MTor inhibitor | 0.0% |
| HD IL-2 | 0.0% |
| Aggressive local regional therapy | 40.0% |
| Observation | 0.0% |
| Clinical trials | 60.0% |
| 1. **What is your initial treatment recommendation for patients with metastatic chromophobe RCC?** | |
| **Answer Options** | **Response Percent** |
| VEGFR TKI | 13.3% |
| MTor inhibitor | 0.0% |
| HD IL-2 | 0.0% |
| Aggressive local regional therapy | 13.3% |
| Observation | 6.7% |
| Clinical Trial | 66.7% |
| 1. **What is your initial treatment recommendation for patients with metastatic sarcomatoid variant RCC?** | |
| **Answer Options** | **Response Percent** |
| VEGFR TKI | 33.3% |
| MTor inhibitor | 6.7% |
| HD IL-2 | 20.0% |
| Cytotoxic chemotherapy | 0.0% |
| Chemotherapy + VEGFR TKI | 26.7% |
| Other | 13.3% |
| 1. **Do you tailor your recommendation for treatment of sarcomatoid RCC based on the percentage of sarcomatoid features?** | |
| **Answer Options** | **Response Percent** |
| No | 60.0% |
| Yes | 40.0% |
| 1. **What is your initial treatment recommendation for patients with Poor prognostic RCC by MSKCC criteria?** | |
| **Answer Options** | **Response Percent** |
| VEGFR TKI | 53.3% |
| Temsirolimus | 20.0% |
| HD IL-2 | 0.0% |
| Clinical trial | 26.7% |
| Hospice/symptomatic care | 0.0% |
| 1. **If faced with an mRCC patient you consider eligible for high-dose IL-2 and also an option of a clinical trial in which the patient was guaranteed to receive a PD-1 based checkpoint inhibitor, all other things being equal, which would you recommend?** | |
| **Answer Options** | **Response Percent** |
| High-dose IL-2 | 6.7% |
| PD-1 based checkpoint inhibitor clinical trial | 93.3% |
| 1. **Which of the following describes your opinion of giving checkpoint inhibitors to patients you consider not eligible for high-dose IL-2?** | |
| **Answer Options** | **Response Percent** |
| Less toxicity | 53.3% |
| More activity in patients with aggressive histology | 20.0% |
| Not proven not to work | 20.0% |
| Can be more readily given after a VEGF inhibitor | 0.0% |
| Other (It’s a reasonable option in a clinical trial) | 6.7% |
| 1. **PD1 blockade is best given in what clinical setting?** | |
| **Answer Options** | **Response Percent** |
| After resistance to VEGFR TKI (s) | 26.7% |
| As initial therapy | 73.3% |
| In the adjuvant setting | 0.0% |
| In the neoadjuvant setting | 0.0% |
| 1. **What patients would you consider for initial checkpoint inhibitor therapy?** | |
| **Answer Options** | **Response Percent** |
| Any patient with metastatic RCC | 60.0% |
| Any patient with clear cell metastatic RCC | 40.0% |
| Patients whose tumors are PDL1 + | 0.0% |
| Would only consider patients after VEGF pathway inhibition | 0.0% |
| Only consider ccRCC patients who have had a cytoreductive nephrectomy. | 0.0% |
| 1. **Which PD1 pathway inhibitor do you consider most promising in mRCC?** | |
| **Answer Options** | **Response Percent** |
| Nivolumab | 40.0% |
| MPDL1-3280A | 0.0% |
| Pembrolizumab | 6.7% |
| Other | 0.0% |
| Don’t know | 53.3% |
| 1. **Which anti-PD1 based combination do you consider most promising in patients with mRCC? (select one):** | |
| **Answer Options** | **Response Percent** |
| With pazopanib | 0.0% |
| With sunitinib | 13.3% |
| With axitinib | 0.0% |
| With bevacizumab | 6.7% |
| With ipilimumab | 53.3% |
| With PEG Intron | 0.0% |
| IL-2 or other cytokine | 6.7% |
| With a vaccine | 6.7% |
| With another immunotherapy | 13.3% |
| Ablative therapy | 0.0% |
| 1. **What is your view about the study of anti-PD1 based treatments in the adjuvant setting in patients with mRCC?** | |
| **Answer Options** | **Response Percent** |
| They are ideal agents for testing in the adjuvant setting | 60.0% |
| PD1 based therapies won’t work in the adjuvant setting because there are no TIL to activate | 0.0% |
| They have exciting potential in the adjuvant setting, although studies will take so long to conduct that they are not relevant for the next decade. | 20.0% |
| Don’t know | 20.0% |
| 1. **The role of PDL1 expression by the tumor in clinical research is best defined by:** | |
| **Answer Options** | **Response Percent** |
| Initial PD1 based therapy should be restricted to patients whose tumors are PDL1+ | 6.7% |
| PDL1 expression should be used as a stratification factor in patients on RCC based clinical trials | 80.0% |
| PDL1+ can only be relied on in a fresh tumor biopsy of a metastasis | 0.0% |
| PDL1+ should not be considered in the context of RCC clinical trials | 13.3% |
| 1. **The study of PD1 based therapy in non-clear cell RCC patients is:** | |
| **Answer Options** | **Response Percent** |
| Not justified by current data and economic environment | 7.1% |
| Worthy of testing particularly in tumor types with high % of PD1+ tumors | 85.7% |
| Only worth testing in patients whose tumors are PDL1+ | 7.1% |
| Only worth testing in combination regimens | 0.0% |
| 1. **The principal arguments in favor of neoadjuvant anti-PD1 based therapy are:** | |
| **Answer Options** | **Response Percent** |
| Restore activity of immune cells before they are removed from tumor microenvironment | 13.3% |
| Good chance to assess efficacy and decide on whether a nephrectomy is indicated or not | 0.0% |
| Good opportunity to study mechanisms of action. | 66.7% |
| Best chance to treat micrometastatic disease | 20.0% |
| 1. **Is there a role for HD IL-2 after failure of an anti-PD1?** | |
| **Answer Options** | **Response Percent** |
| Yes, they work by different mechanisms so still may work | 60.0% |
| Yes, anti-PD1 sticks around for a while and by blocking PDL1 will enhance the efficacy of subsequent IL-2 | 13.3% |
| No, if anti-PD1 doesn’t work then the tumor is not immune responsive. | 0.0% |
| No, many other regimens should be explored first. | 26.7% |
| 1. **When should an anti-PD1 based therapy be stopped in a responding patient?** | |
| **Answer Options** | **Response Percent** |
| Only after a CR | 46.7% |
| After a year | 6.7% |
| After 2 years | 13.3% |
| Never | 0.0% |
| Other (maximal response/toxicity, important to test 1 year and 2 years in clinical trials, unknown, individualized decision) | 33.3% |
| 1. **Is Sunitinib an immunomodulator?** | |
| **Answer Options** | **Response Percent** |
| Yes, It decreases Tregs and MDSCs in the peripheral blood | 60.0% |
| Yes, it blocks Stat 3 function which enhances immune cell function | 6.7% |
| No, relevant changes have not been identified within the tumor microenvironment | 13.3% |
| No, effects are primarily driven by tumor shrinkage. | 20.0% |
| 1. **Which vaccine studies to you think will be positive?** | |
| **Answer Options** | **Response Percent** |
| Imatics | 6.7% |
| AGS-003 | 40.0% |
| Tumor cell fusions | 0.0% |
| All of the above | 0.0% |
| None of the above | 53.3% |

**Note:** These questions were asked prior to the FDA approval of nivolumab for patients with advanced RCC.
